# Synthesis of 4‐Deoxy‐4‐Fluoro‐d‐Sedoheptulose: A Promising New Sugar to Apply the Principle of Metabolic Trapping

**DOI:** 10.1002/chem.202302277

**Published:** 2023-09-28

**Authors:** Lukas Scheibelberger, Toda Stankovic, Marlene Pühringer, Hanspeter Kählig, Theresa Balber, Eva‐Maria Patronas, Evelyn Rampler, Markus Mitterhauser, Arvand Haschemi, Katharina Pallitsch

**Affiliations:** ^1^ Institute of Organic Chemistry University of Vienna Währinger Straße 38 1090 Vienna Austria; ^2^ Vienna Doctoral School in Chemistry (DoSChem) University of Vienna Währinger Straße 42 1090 Vienna Austria; ^3^ Institute of Analytical Chemistry University of Vienna Währinger Straße 38 1090 Vienna Austria; ^4^ Division of Nuclear Medicine Department of Biomedical Imaging and Image-guided Therapy Medical University of Vienna Währinger Gürtel 18–20 1090 Vienna Austria; ^5^ Ludwig Boltzmann Institute Applied Diagnostics Währinger Gürtel 18–20 1090 Vienna Austria; ^6^ Division of Pharmaceutical Technology and Biopharmaceutics Department of Pharmaceutical Sciences University of Vienna, UZAII Josef-Holaubek-Platz 2 1090 Vienna Austria; ^7^ Institute of Inorganic Chemistry University of Vienna Währinger Straße 42 1090 Vienna Austria; ^8^ Department of Laboratory Medicine Medical University of Vienna Währinger Gürtel 18–20 1090 Vienna Austria

**Keywords:** fluorinated carbohydrates, metabolic trapping, pentose phosphate pathway, rare sugars, d-sedoheptulose

## Abstract

Fluorinated carbohydrates are important tools for understanding the deregulation of metabolic fluxes and pathways. Fluorinating specific positions within the sugar scaffold can lead to enhanced metabolic stability and subsequent metabolic trapping in cells. This principle has, however, never been applied to study the metabolism of the rare sugars of the pentose phosphate pathway (PPP). In this study, two fluorinated derivatives of d‐sedoheptulose were designed and synthesized: 4‐deoxy‐4‐fluoro‐d‐sedoheptulose (**4DFS**) and 3‐deoxy‐3‐fluoro‐d‐sedoheptulose (**3DFS**). Both sugars are taken up by human fibroblasts but only **4DFS** is phosphorylated. Fluorination of d‐sedoheptulose at C‐4 effectively halts the enzymatic degradation by transaldolase and transketolase. **4DFS** thus has a high potential as a new PPP imaging probe based on the principle of metabolic trapping. Therefore, the synthesis of potential radiolabeling precursors for **4DFS** for future radiofluorinations with fluorine‐18 is presented.

## Graphical abstract

**Synthesis of 4‐deoxy‐4‐fluorosedoheptulose** (**4DFS**) and 3‐deoxy‐3‐fluorosedoheptulose (**3DFS**) as well as of suitable precursors for the synthesis of their [^18^F]‐analogues is described. First biochemical investigations show that **4DFS** is taken up by human fibroblasts and phosphorylated (giving **4DFS7P**) but not further metabolized by transaldolase or transketolase. Thus, **4DFS** has a high potential as new pentose phosphate pathway imaging probe.

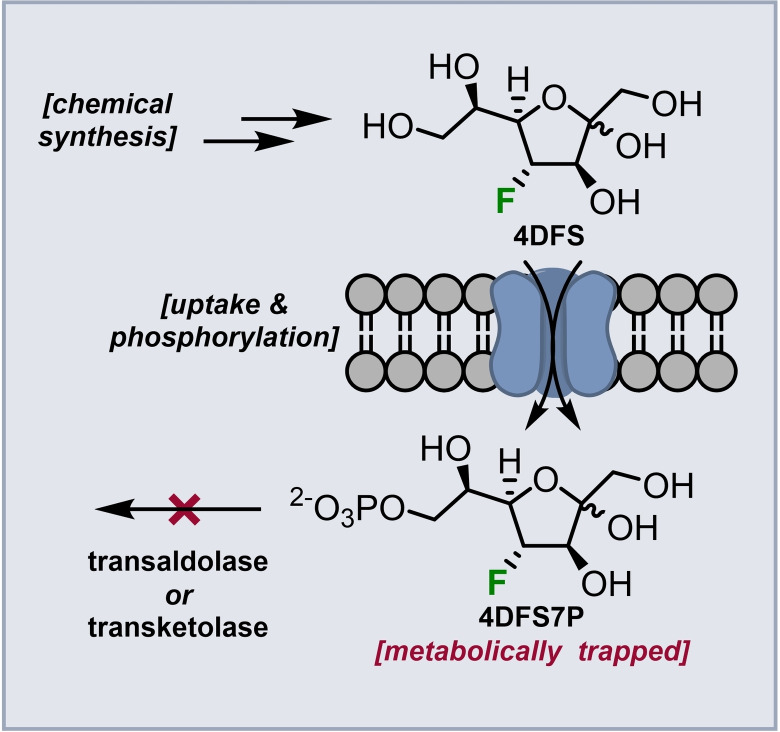

## Introduction

The pentose phosphate pathway (PPP) is a branch of carbohydrate metabolism that shares several common intermediates with glycolysis. It provides cells with the required reducing agents (NADPH) for redox regulation and lipid biosynthesis and is the sole source of ribose‐5‐phosphate (**R5P**) for *de novo* nucleotide biosynthesis.^[1]^ It consists of an irreversible oxidative (oxPPP) phase, and a highly complex network of reversible reactions forming a non‐oxidative (non‐oxPPP) phase. During the non‐oxPPP, carbon scrambling events between monosaccharides of different chain length (C_3_, C_4_, C_5,_ C_6_ and C_7_ sugars)^[2]^ take place to ultimately generate ribose‐5‐phosphate (**R5P**) or revert excessive pentose phosphates back to glycolytic intermediates. Apart from their role during **R5P** formation, these rare sugar phosphates were long considered to be otherwise insignificant metabolic intermediates. The recent discovery of specialized kinases for d‐ribulose, d‐xylulose and d‐sedoheptulose[^3^, ^4^, ^5^, ^6^, ^7^] suggests that these rare sugars can be directly shuffled into primary carbohydrate metabolism. This also raises questions on the extent to which cells consume and rely on these rare sugars^7^ to directly modulate the carbon flux between the non‐oxPPP and glycolysis by influencing the stoichiometry of the involved metabolites.^7^ The respective kinases might even be selectively regulated in areas with elevated oxidative stress or high need for **R5P** during cell proliferation.

This is of particular interest as elevated flux rates towards the PPP and altered PPP enzyme expression levels are often associated with severe pathologies, such as cancer.[^8^, ^9^, ^10^, ^11^, ^12^, ^13^, ^14^, ^15^, ^16^, ^17^, ^18^] Thus, highly sensitive methods are needed to determine flux rates towards the PPP.

**Scheme 1:**
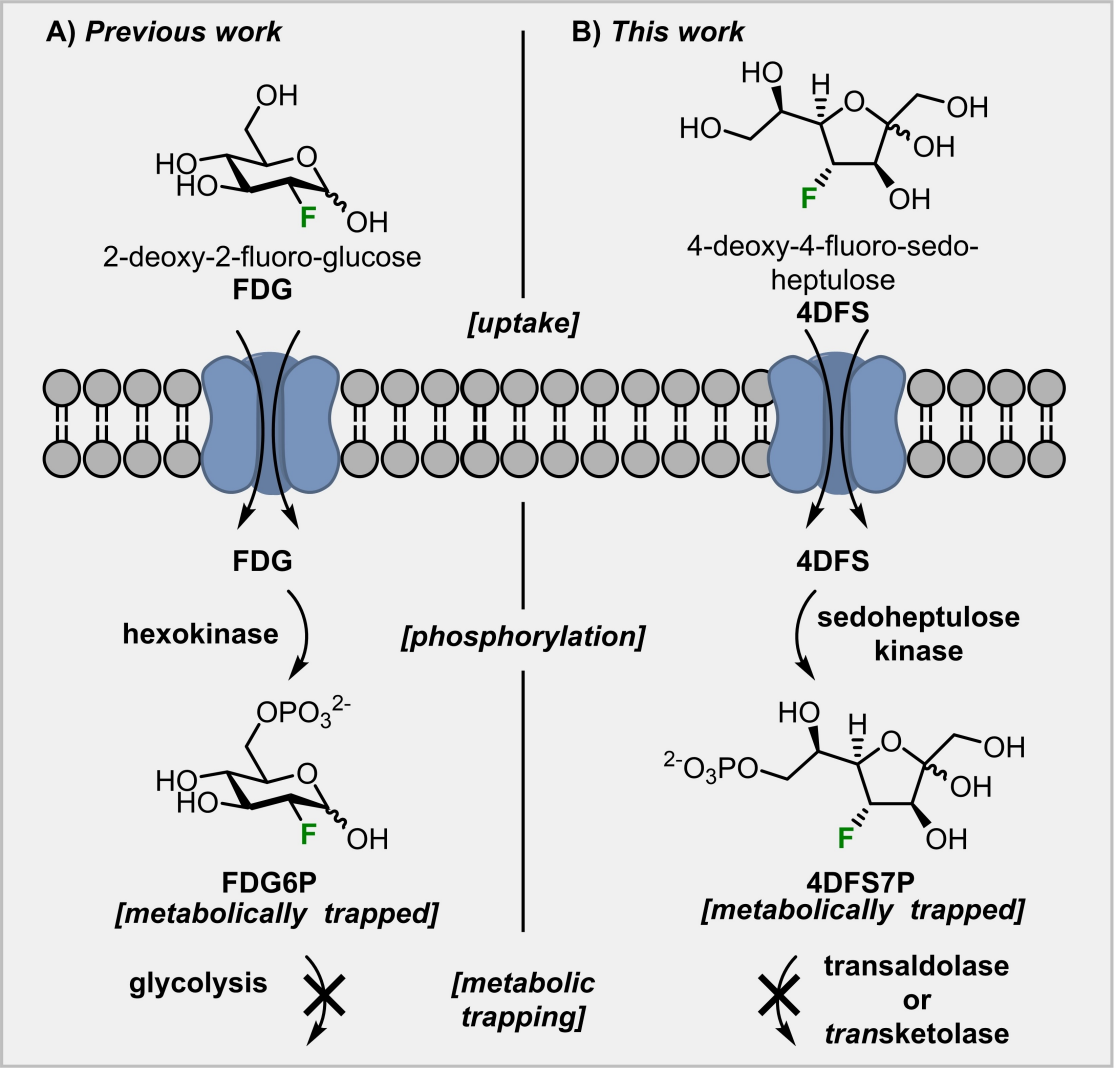
Comparison of previous use of the principle of metabolic trapping and this work.

**Scheme 2:**
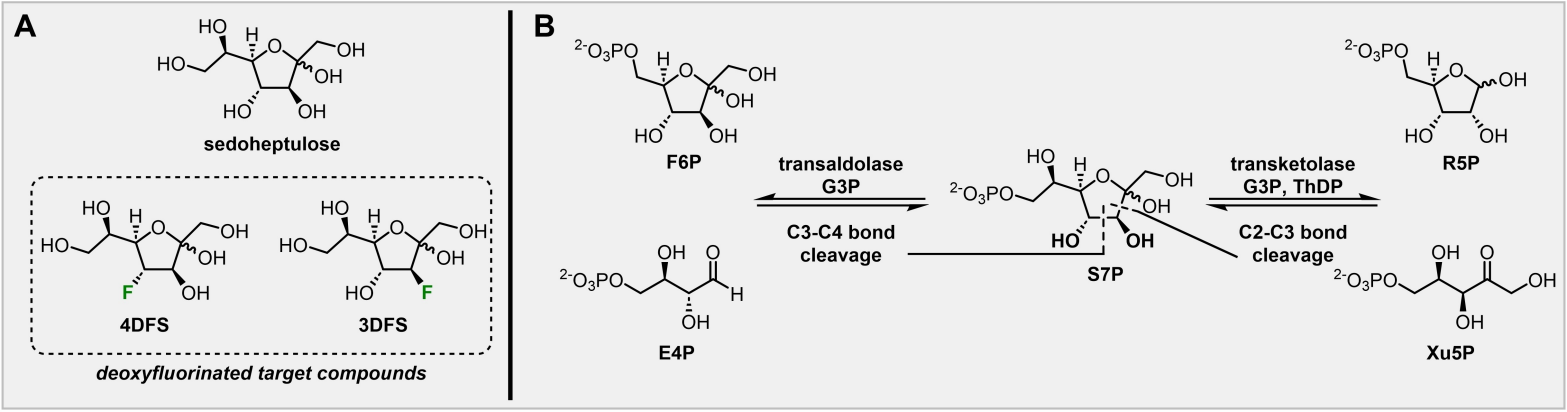
**A**: Structures of d‐sedoheptulose and designed deoxyfluorinated target compounds 4‐deoxy‐4‐fluoro‐d‐sedoheptulose (**4DFS**) and 3‐deoxy‐3‐fluoro‐d‐sedoheptulose (**3DFS**). **B**: Enzymatic transformations of d‐sedoheptulose‐7‐phosphate (**S7P**) during the non‐oxidative PPP; **F6P**=fructose‐6‐phosphate, **E4P**=erythrose‐4‐phosphate, **G3P**=glyceraldehyde‐3‐phosphate, **R5P**=ribose‐5‐phosphate, **Xu5P**=xylulose‐5‐phosphate, **ThDP=**thiamine diphosphate.

Inspired by the mode of action of 2‐deoxy‐2‐[^18^F]fluoro‐glucose ([^18^F]**FDG**),^[19]^ which is the most widely used^[20]^ positron emission tomography (PET) tracer for glucose‐dependent energy consumption imaging, we designed a possible strategy to visualize areas of elevated carbohydrate metabolism via the PPP based on two deoxy‐fluorinated analogs of d‐sedoheptulose (Scheme 2A). Replacing the stable isotope fluorine‐19 with the positron emitting radionuclide fluorine‐18, opens up the possibility to study carbohydrate metabolism in a highly sensitive way *in vivo* by PET imaging.^[21]^ [^18^F]FDG is taken up by cells via glucose transporters, phosphorylated by hexokinase but cannot be broken down by glycolysis (Scheme 1). Hence, [^18^F]FDG‐6‐phosphate (**FDG6P**) accumulates inside cells with a high glucose turnover and allows the visualization of altered metabolic rates of glucose. We want to apply this principle to a different sugar‐phosphate that plays a vital role for the human metabolism: sedoheptulose‐7‐phosphate.

## Results and Discussion

### Design of Target Compounds

Based on the discovery of a specific sedoheptulose kinase (SHPK),^[5]^ we hypothesized that deoxy‐fluorinated d‐sedoheptulose derivatives, such as 4‐deoxy‐4‐fluoro‐d‐sedoheptulose (**4DFS**) and 3‐deoxy‐3‐fluoro‐d‐sedoheptulose (**3DFS**) could be used to identify areas with an upregulation of the non‐oxPPP in a similar way to glucose metabolism imaging with **FDG**. **S7P** can be metabolized by two enzymes: transaldolase (TALDO1) and transketolase (TKT, scheme 2B). While TALDO1 cleaves the C−C bond between carbon 3 and 4 of **S7P** before ultimately oxidizing C‐4 and forming erythrose‐4‐phosphate (**E4P**), TKT removes a C2‐unit of **S7P** by breaking the C−C bond between carbon 2 and 3 and then oxidizes C‐3 to form **R5P**. We assume that **4DFS** and **3DFS**, if accepted by SHPK, would form the respective fluorinated sugar‐phosphates and that the C−F bond would then block the attack by either TALDO1 (in case of **4DFS**) or TKT (in case of **3DFS**). Thus, they should finally be metabolically trapped inside cells. Support for this hypothesis comes from Evdokimov *et al*. who found that 2‐deoxy‐2‐fluoro‐d‐ribose‐5‐phosphate is converted by TKT in a similar way as its natural substrate (**R5P**) to give 4‐deoxy‐4‐fluoro‐d‐sedoheptulose‐7‐phosphate (**4DFS7P**).^[22]^ The latter is, however, not accepted as a substrate by TALDO1 and thereby metabolically trapped *in viv*o. The corresponding fluorine‐18 labeled compounds, [^**18**^ **F**]**4DFS** and [^**18**^ **F**]**3DFS**, could be used as PET tracers to identify areas of elevated carbohydrate metabolism via the PPP and hint at pathology‐related deregulations. Thus, we aimed at the synthesis of **4DFS** and **3DFS** as well as of suitable precursors for the synthesis of their radiolabeled counterparts.

### Synthesis of deoxyfluorinated sugars

We set out to probe our hypothesis by first synthesizing 4‐deoxy‐4‐fluoro‐d‐sedoheptulose (**4DFS**) and 3‐deoxy‐3‐fluoro‐d‐sedoheptulose (**3DFS**). Recent examples of *de novo* synthetic strategies for the synthesis of fluorinated carbohydrate scaffolds led to impressive results regarding the obtained level of stereocontrol.[^23^, ^24^] However, we did not choose a *de novo* synthetic approach for the synthesis of **4DFS** as Yan *et. al* already showed that both *manno* and *allo* configured sugar epoxides can be used for fluorinations. This makes both the desired fluorinated *altro* configured pyranose core structures of **3DFS** and **4DFS** accessible from the same stable and commercially available starting material within 3 steps.^[25]^ Thus, we aimed to combine this epoxide opening approach with the C_1_‐elongation method developed by Waschke *et al*. for synthesizing these heptuloses.^[26]^

**Scheme 3:**
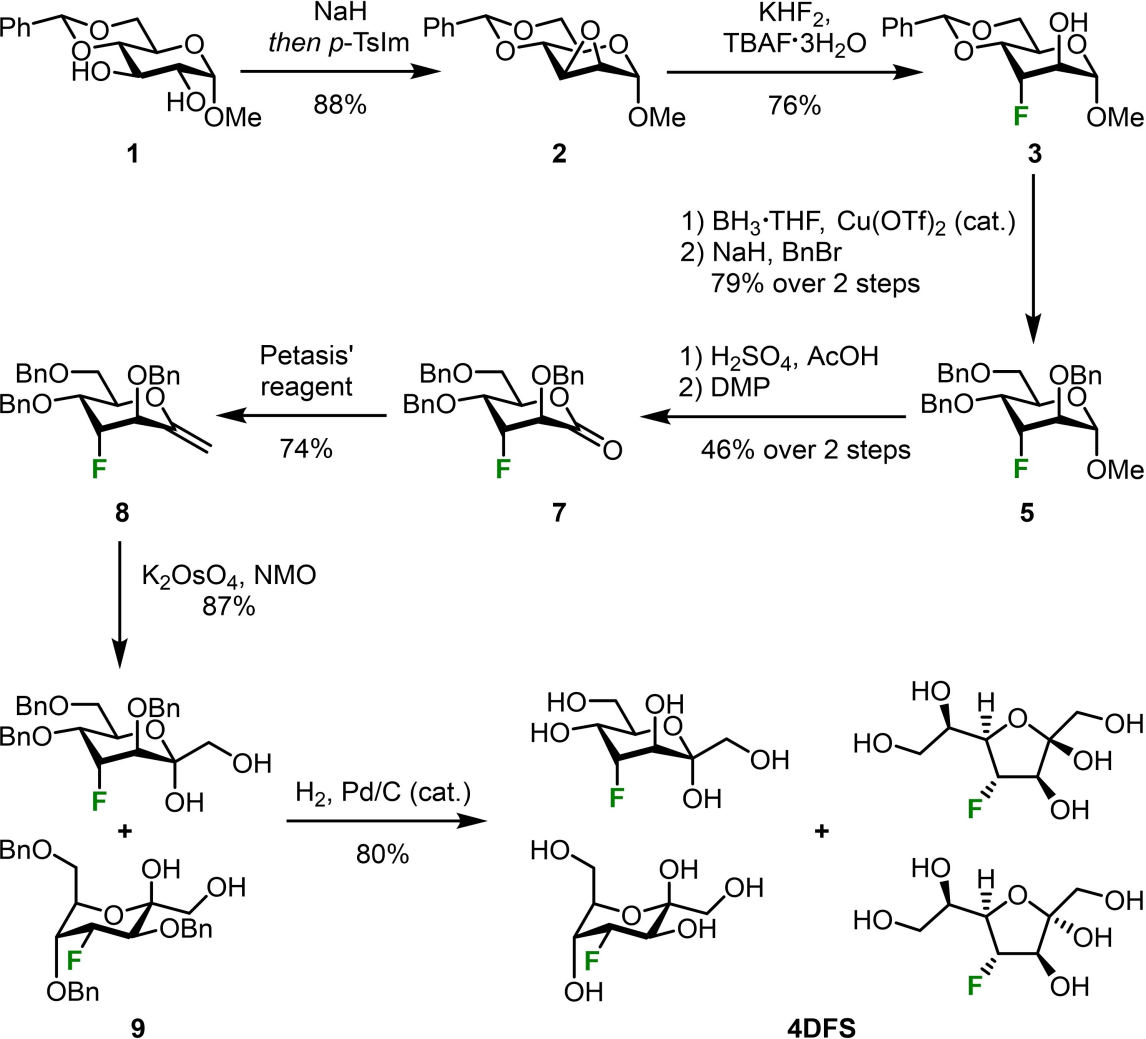
Synthesis of 4‐deoxy‐4‐fluoro‐d‐sedoheptulose (**4DFS**) from methyl 4,6‐*O*‐benzylidene‐α‐d‐*gluco*‐pyranoside (**1**).

Commercially available building block **1** was used as starting point for the synthesis of **4DFS** (scheme 3). The corresponding dialkoxide was generated using sodium hydride and subsequent careful addition of *para*‐toluenesulfonyl imidazole (*p*‐TsIm). This led to selective tosylation at the O‐2 position followed by immediate nucleophilic replacement by the O‐3 alkoxide to give epoxide **2**.[^27^, ^28^] The obtained *manno*‐epoxide **2** was then opened in a trans‐diaxial fashion with *N,N,N,N*‐tetrabutylammonium fluoride trihydrate (TBAF**×**3 H_2_O) and potassium bifluoride on a gram‐scale to give compound **3** as a single regioisomer.^[25]^ Selective reductive opening of the benzylidene acetal at position 6 was achieved by the protocol described by Shie *et al*.^[29]^ and subsequent benzylation of the resulting diol gave fully protected *altro*‐pyranoside **5**. The methyl glycoside was then hydrolyzed under acidic conditions^[30]^ to give the corresponding lactol which could be smoothly oxidized to lactone **7** with Dess‐Martin periodinane (DMP).^[31]^ We used Petasis’ reagent as a C_1_‐elongation method to obtain our desired C_7_‐sugar.[^32^, ^33^] The resulting exocyclic enol ether **8** was subsequently dihydroxylated with *N*‐methylmorpholine‐*N*‐oxide (NMO) in the presence of catalytic amounts of potassium osmate to give heptulose **9** as a mixture of its α‐anomer in ^5^ *C*_2_‐conformation and β‐anomer in ^2^ *C*_5_‐conformation.^[34]^ Final deprotection gave **4DFS** as a mixture of its α‐ and β‐furanose and its α‐ and β‐pyranose in a 1.5 : 4 : 4 : 1 ratio as determined by 2D NMR experiments (HMBC and NOESY).

**Scheme 4:**
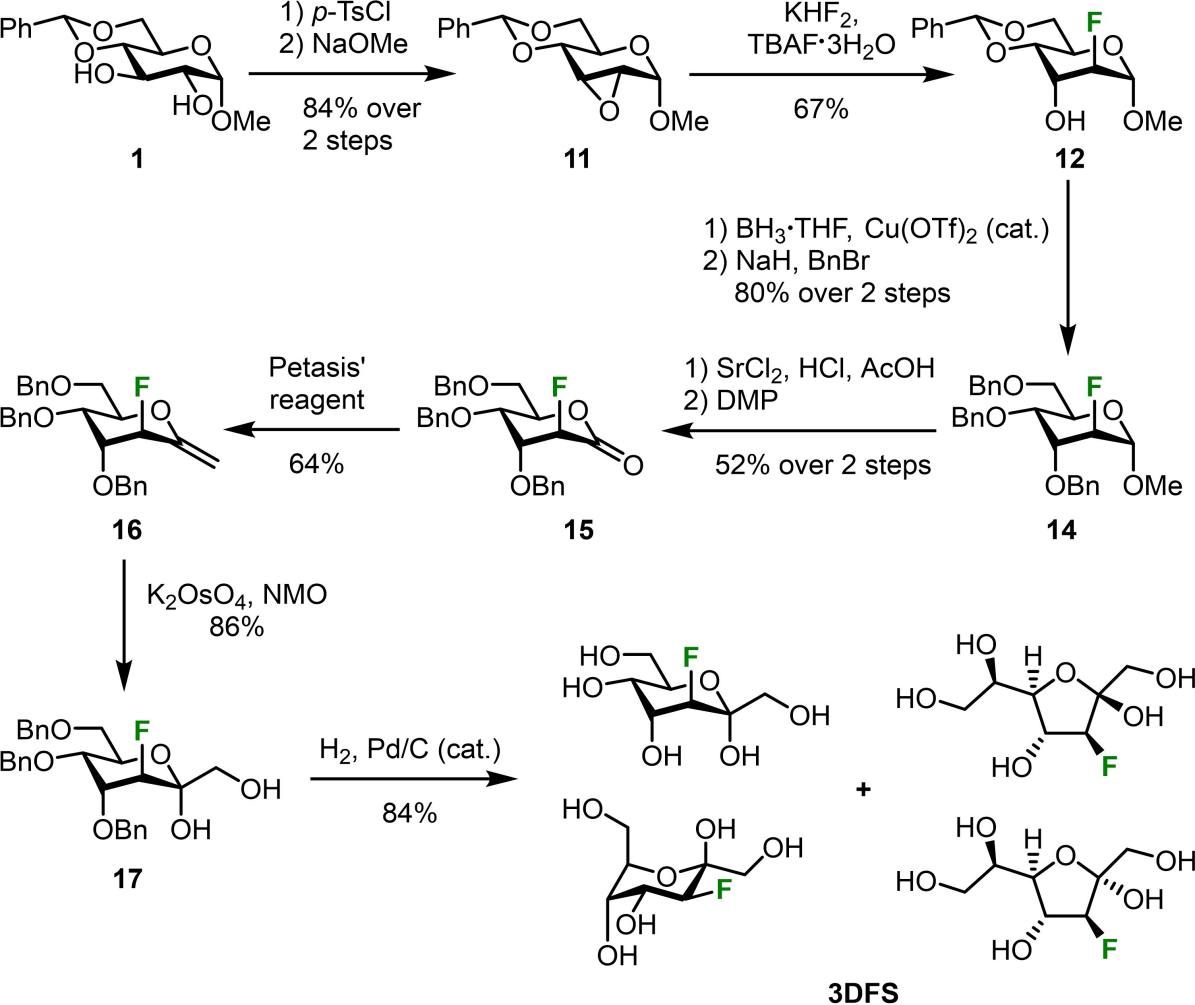
Synthesis of 3‐deoxy‐3‐fluoro‐d‐sedoheptulose (**3DFS**) from methyl 4,6‐*O*‐benzylidene‐α‐d‐*gluco*‐pyranoside (**1**).

3‐Deoxy‐3‐fluoro‐d‐sedoheptulose was obtained in a similar way: Methyl glucoside **1** again served as starting material (Scheme 4). However, this time **1** was di‐tosylated with *para*‐toluenesulfonyl chloride (*p*‐TsCl).^[35]^ Subsequent treatment of intermediate **10** (see Experimental Section) with sodium methoxide^[36]^ led to the formation of the *allo*‐configured epoxide **11** which was again opened in a trans‐diaxial fashion as described above. In this case, the epoxide opening gave a 4 : 1 mixture of the desired fluorinated sugar **12** and undesired *gluco*‐configured regioisomer (methyl 4,6‐*O*‐benzylidene‐3‐deoxy‐3‐fluoro‐α‐d‐*gluco*‐pyranoside). Altroside **12** then underwent the same benzylidene opening and benzylation protocols as described for **4DFS**. Hydrolysis of the methyl glycoside proved to be troublesome in this case and both sulfuric acid and acetic acid led to decomposition of the compound. Strontium chloride in a 1 : 1 mixture of aqueous hydrochloric acid (5 M) and glacial acetic acid^[37]^ finally provided the desired crude lactol which was immediately oxidized with DMP yielding lactone **15**. Methylenation, dihydroxylation and hydrogenolysis of the benzyl groups gave **3DFS** again as a mixture of its α‐ and β‐ furanose and α‐ and β‐pyranose in a 2.4 : 3 : 4 : 1 ratio, respectively.

### First biochemical evaluations

**Figure 1:**
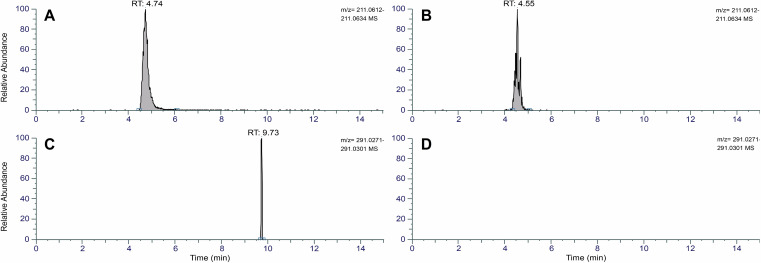
Extracted‐ion chromatograms (*m/z* 211.0623, mass tolerance=5 ppm) of **4DFS** (**A**) and **3DFS** (**B**) uptake assays with human fibroblasts. Extracted‐ion chromatograms (*m/z* 291.0286, mass tolerance=5 ppm) for corresponding sugar phosphates **4DFS7P** (**C**) and **3DFS7P** (not detected, **D**) in the same samples. HILIC‐MS analysis was done with cell lysates, see Experimental Section and Supporting Information for experimental details. RT=retention time.

**Figure 2:**
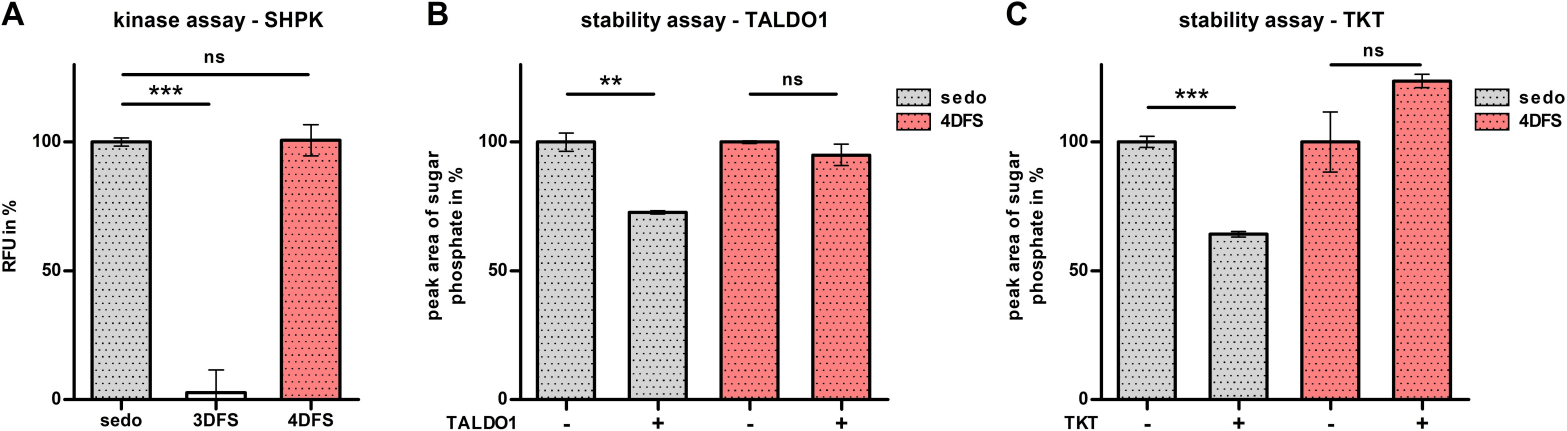
**A**: Fluorescence‐based ADP‐accumulation assay with sedoheptulose kinase (SHPK). The graph represents the change in fluorescence signal arising from ATP consumption due to sugar phosphorylation by SHPK. Positive control (**sedo**) was normalized to 100 %. **B** & **C**: Enzymatic stability assays of **4DFS** in the presence of SHPK and either transaldolase (TALDO1, **B**) or transketolase (TKT, **C**). The shown graph represents the peak area of the respective sugar phosphate after HILIC‐MS analysis compared to the negative (−) control samples (no TALDO1 ***or*** no TKT added) which were normalized to 100 %. Data is presented as the mean±standard error of mean (SEM) and was analyzed with an unpaired two‐samples t‐test: *** *P*<0.001, ** *P*<0.01, ns=not significant. RFU=relative fluorescence units, **sedo**=d‐sedoheptulose, **3DFS**=3‐deoxy‐3‐fluoro‐d‐sedoheptulose, **4DFS**=4‐deoxy‐4‐fluoro‐d‐sedoheptulose.

With **3DFS** and **4DFS** in hand, we wanted to assess whether the fluorinated heptuloses are taken up by cells, then phosphorylated by sedoheptulose kinase (SHPK), and finally whether the fluorine atom halts the enzymatic degradation of the resulting sugar phosphates by TALDO1 and TKT. First uptake studies were conducted with human fibroblasts. The medium of the cells was supplemented with **3DFS**, **4DFS** or water (control) and incubated for 10 min. After thorough washing, lysis of the cells and removal of macromolecules by centrifugation, the supernatant was evaporated and then subjected to HILIC‐MS analysis. **3DFS** and **4DFS** were both shown to be taken up by the cells which was verified by mass spectrometry (Figure 1A & B; HILIC‐MS negative mode: *m/z* 211.0623 [*M*−H]^−^, *R*_T_=4.74 min (**4DFS**) and 4.55 min (**3DFS**)). Both peaks were absent in the control sample (Figure S1, Supporting Information). We then checked for first signs of further metabolism of **4DFS** and **3DFS** using the same samples and indeed could detect the mass of the corresponding sugar phosphate of **4DFS** (Figure 1C, *m/z* 291.0286 [*M*‐H]^−^, *R*_T_=9.73 min) while the respective **3DFS** phosphate was not found (Figure 1D). The exact three‐dimensional structure of SHPK is still unknown, which makes predictions about its substrate promiscuity difficult. Thus, we conducted an ADP‐accumulation‐based kinase assay to study the phosphorylation of our novel fluorinated sugars by SHPK in more detail. As expected from the cell assays, **3DFS** and **4DFS** showed very different results. No ATP consumption was detected during the fluorescence‐based SHPK assay with **3DFS**. While **4DFS** was phosphorylated with no significant difference to the native substrate (d‐sedoheptulose), **3DFS** was not accepted by SHPK at all (Figure 2A), suggesting that the OH‐group at position 3 is crucial for kinase activity and cannot be substituted with fluorine. Since phosphorylation is a prerequisite for the anticipated metabolic trapping mechanism, we continued the biochemical evaluation solely with **4DFS**. Next, we wanted to see if the C−F bond in **4DFS** effectively prevents the enzymatic degradation of the sugar phosphate by TALDO1 and TKT. Thus, *in vitro* stability assays^[38]^ were conducted. For these, **4DFS** or d‐sedoheptulose (control) were enzymatically phosphorylated by SHPK in the presence of either TALDO1 or TKT to test the enzymatic stability of the sugar phosphates. Control experiments clearly show consumption of **S7P** by both enzymes, while no significant **4DFS** phosphate consumption was detected by HILIC‐MS analysis. To our surprise, these experiments clearly demonstrated that phosphorylated **4DFS** is neither a substrate for TALDO1 nor for TKT (Figure 2B and C). This makes **4DFS** a promising candidate for the development of a new sugar‐based imaging probe relying on the principle of metabolic trapping.

### Synthesis of potential radiolabeling precursors for 4DFS

**Scheme 5:**
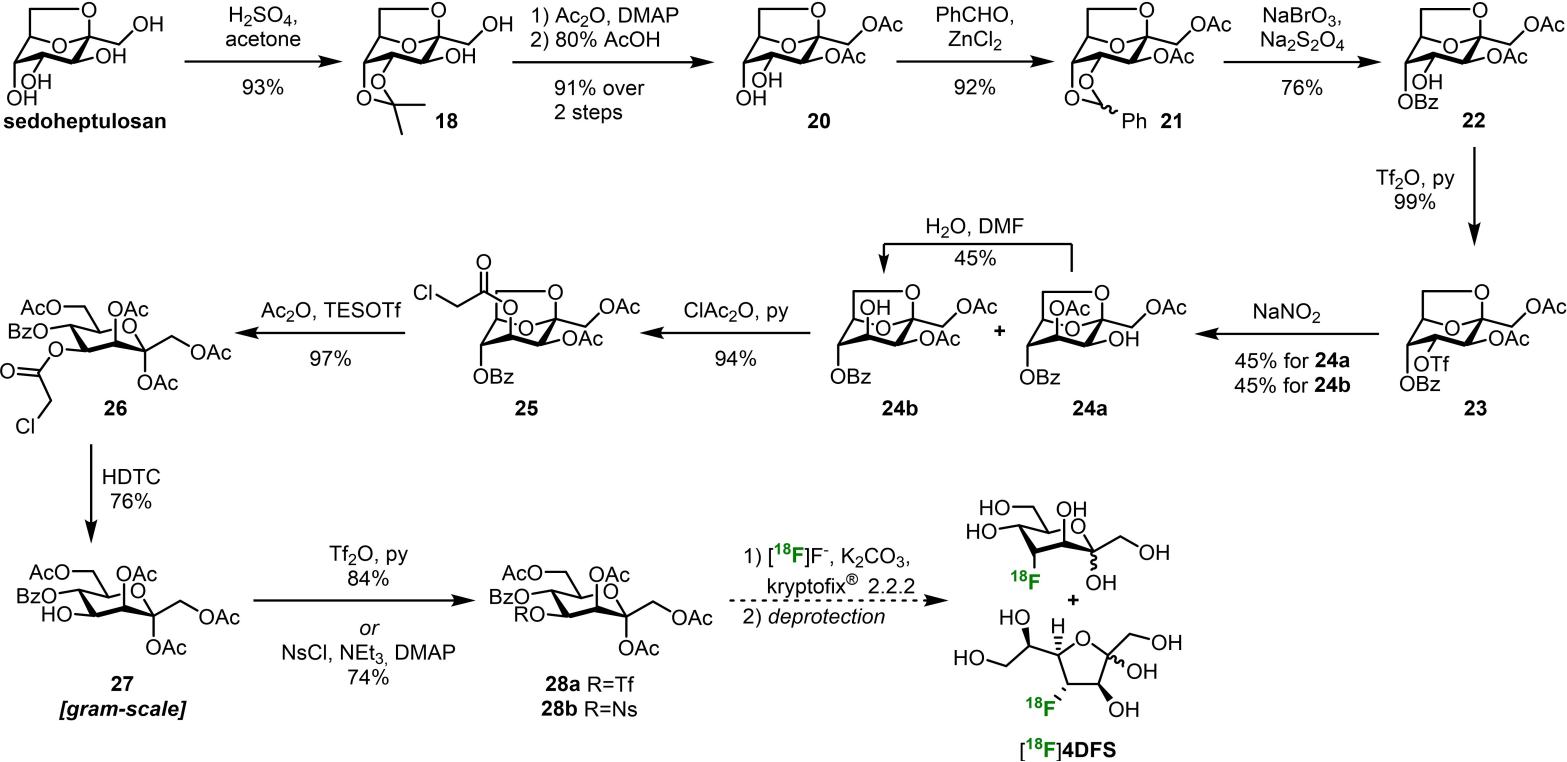
Synthesis of potential radiolabeling precursors **28 a** and **28 b** from sedoheptulosan. Dashed reaction arrows indicate reactions that have not been performed yet.

Prompted by these results we set out to synthesize a radiolabeling precursor for **4DFS** that allows the late‐stage introduction of fluorine‐18 (*t*_1/2_=110 min; see Supporting Information for details on the synthesis of potential radiolabeling precursors for **3DFS**). The synthesis starts with commercially available sedoheptulosan monohydrate which was selectively isopropylidene protected at position 4 and 5 (**18**, Scheme 5).^[39]^ Acetylation^[40]^ of the free hydroxy groups followed by acidic removal of the acetonide yielded diol **20**.^[41]^ Benzylidene protection then yielded **21** ^[42]^ and set the stage for oxidative opening of the acetal with sodium bromate and sodium dithionite to give the key intermediate **22**.^[43]^ For a radiofluorination of the ‐ now unprotected ‐ position 4 with overall retention of configuration we first had to invert this stereocenter. The best results for this transformation were obtained by a Latrell‐Dax‐epimerization.^[44]^ Unfortunately, the reaction always led to acetate migration from O‐3 to O‐4 to give a 1 : 1 mixture of products **24 a** and **24 b**. However, we could take advantage of the equilibrium of this migration by separating the unwanted compound **24 a** from the desired sugar **24 b** and letting **24 a** reach the equilibrium state in wet DMF again. Thereby, we were able to push the equilibrium to the side of **24 b**. Subsequent chloroacetylation at position 4 gave compound **25** with a suitable orthogonal protecting group now in place. Acetolysis of the 2,7‐*anhydro*‐bridge with triethylsilyl triflate (TESOTf) in acetic anhydride yielded heptulose **26**.^[45]^ Selective removal of the chloroacetate with hydrazine dithiocarbonate (HDTC) gave **27**. The excellent yields of all previous transformations allowed us to access this key intermediate on a multigram scale.^[46]^ Installation of various good leaving groups for future radiofluorinations worked smoothly. The reaction with triflic anhydride or nosyl chloride^[47]^ gave the potential radiolabeling precursors **28 a** and **28 b**, respectively, which will be tested as substrates for late‐stage radiofluorinations in the future.

## Conclusions

In summary, the first syntheses of 4‐deoxy‐4‐fluoro‐d‐sedoheptulose (**4DFS**) and 3‐deoxy‐3‐fluoro‐d‐sedoheptulose (**3DFS**) were accomplished. Epoxide opening of a pyranoside with TBAF and KHF_2_ and subsequent C_1_‐elongation with Petasis’ reagent followed by a dihydroxylation of the double bond were key steps for the synthesis of both sugars. **4DFS** and **3DFS** were readily taken up by human fibroblasts as seen by HILIC‐MS analysis but only **4DFS** was phosphorylated by SHPK. Fluorination at position 4 in d‐sedoheptulose effectively prevented the enzymatic degradation of the corresponding sugar phosphate (**4DFS**‐7‐phosphate) as verified by *in vitro* stability assays with SHPK and either TALDO1 or TKT. This makes **4DFS** a promising deoxyfluorinated sugar probe for biomedical imaging relying on the principle of metabolic trapping. Ultimately, we have established a synthetic strategy to access two potential precursors (**28 a** & **28 b**) for future synthetic attempts to access [^18^F]**4DFS** during radiofluorination experiments.

## Experimental Section

### General experimental details

^1^H, ^13^C and ^19^F NMR spectra were recorded on either a Bruker AV III HD 700 (^1^H: 700.40 MHz, ^13^C: 176.12 MHz, ^19^F: 659.03 MHz), AV III 600 (^1^H: 600.25 MHz, ^13^C: 150.93 MHz, ^19^F: 564.803 MHz) or AV NEO 500 (^1^H: 500.32 MHz, ^13^C: 125.81 MHz) spectrometer. Chemical shifts (δ) are given in parts per million (ppm) and were referenced to (residual) solvent signals as follows: ^1^H NMR spectra: CDCl_3_: δ_H_ (CHCl_3_) 7.26, *d_6_*‐DMSO: δ_H_ [(CD_2_H)SO(CD_3_)] 2.50, *d_4_*‐MeOH: δ_H_ (CHD_2_OD) 3.31, and D_2_O: δ_H_ (HDO) 4.79; ^13^C NMR spectra: CDCl_3_ (δ_C_ 77.16), *d_6_*‐DMSO (δ_C_ 39.52), and *d_4_*‐MeOH (δ_C_ 49.00). ^13^C NMR spectra in D_2_O were referenced indirectly to the ^1^H NMR frequency of the sample using the ”xiref”‐command in Bruker Topspin. External CCl_3_F (δ_F_ 0.00) served as reference for ^19^F NMR spectra. Coupling constants (*J*) are reported in Hz. ^13^C spectra were recorded *j*‐modulated. The chemical shift of the two parts of AB‐systems are given separately as unweighted mean value of the single signals. “A” is used to denote the down‐field part and “B” to denote the up‐field part of the AB‐system. High resolution mass spectrometry (HRMS) was conducted on a Bruker maXis UHR‐TOF instrument with electrospray ionization (ESI) in the positive ion mode. Optical rotations were measured on a Schmidt‐Haensch Digital Polarimeter Unipol L 2000 and are given in 10‐^‐1^ deg cm^2^ g^‐1^. Chromatographic separations (MPLC) were carried out on a Biotage Isolera Prime (Biotage, Uppsala, Sweden) flash purification system using Macherey–Nagel silica gel 60 (0.04‐0.063 mm) in self‐packed cartridges. Thin layer chromatography (TLC) was carried out on precoated Merck silica gel 60 F_254_ glass plates or precoated Macherey‐Nagel ALUGRAM Xtra SIL G UV_254_ aluminum plates. Compounds were visualized with UV light (254 nm) and/or by dipping the plate in one of the following solutions, followed by heating with a heat gun: Cerium ammonium molybdate solution (CAM, 46 g (NH_4_)_6_Mo_7_O_24_ **×**4 H_2_O, 2 g Ce(SO_4_)_2_⋅4H_2_O) in 1 L 10 %(w/w) aq. H_2_SO_4_); KMnO_4_ solution (9 g KMnO_4_, 60 g K_2_CO_3_ in 900 mL H_2_O and 15 mL 5 %(w/w) aq. NaOH) or vanillin stain (60 g vanillin in 1 L EtOH and 10 mL conc. H_2_SO_4_) followed by heating. 1‐(*para*‐Toluenesulfonyl)imidazole^[48]^ and Petasis’ reagent^[49]^ were obtained following literature procedures. All other used chemicals and solvents were purchased from commercial sources and used without further purification. Procedures describing the key steps of the synthesis of 4‐deoxy‐4‐fluoro‐d‐sedoheptulose (**4DFS**) and 3‐deoxy‐3‐fluoro‐d‐sedoheptulose (**3DFS**) are described below, followed by details on the biochemical evaluation of these compounds. The full synthetic sequence leading to the target compounds, including all analytical data can be found in the Supporting Information to the manuscript.

Additionally, the synthetic details for the preparation of the four potential radiolabeling precursors (**28 a** and **28 b** for the synthesis of [^18^F]**4DFS**; and **S11** and **S13** for the synthesis of [^18^F]**3DFS**) are reported in the Supporting Information.

### Synthesis of 4‐deoxy‐4‐fluoro‐d–sedoheptulose (4DFS)

**Methyl 4‐*O*‐benzyl‐3‐deoxy‐3‐fluoro‐α‐d–*altro*‐pyranoside (4)** was synthesized following a protocol by Shie *et al*.^*[*29]^ Borane THF complex (1 M in THF, 52.0 mL, 51.99 mmol, 5 equiv.) was slowly added to methyl 4,6‐*O*‐benzylidene‐3‐deoxy‐3‐fluoro‐α‐d‐*altro*‐pyranoside[^27^, ^28^] (**3**, 2.96 g, 10.40 mmol, 1 equiv.) under an argon atmosphere and the reaction mixture was stirred for 10 min at room temperature. Cu(OTf)_2_ (376 mg, 1.04 mmol, 0.1 equiv.) was added and stirring was continued for 1.5 h. The black suspension was then cooled to 0 °C and triethylamine (1.052 g, 1.45 mL, 1.04 mmol, 1 equiv.) was added dropwise followed by methanol (36 mL). The mixture was warmed to room temperature and co‐evaporated thrice with methanol. The crude residue was purified by MPLC (100 g silica gel, 20–100 % EA in *n‐*heptane) to obtain methyl 4‐*O*‐benzyl‐3‐deoxy‐3‐fluoro‐α‐d‐*altro*‐pyranoside (**4**) as a colorless oil (2.95 g, 99 %); *R*_f_=0.34 (*n‐*heptane/EA 1 : 3, UV & vanillin); $[{\alpha ]}_{D}^{20}$ =+106.1 (c=1.0 in CHCl_3_); ^1^H NMR (600 MHz, CDCl_3_) δ=7.38‐7.33 (m, 4H, H^Ar^), 7.33‐7.29 (m, 1H, H^Ar^), 4.86 (dt, ^2^ *J*_3,F_=49.1 Hz, ^3^ *J*_3,2_=^3^ *J*_3,4_=2.9 Hz, 1H, H‐3), 4.73 (d, ^2^ *J*_H,H_=11.7 Hz, 1H, H^Bn^), 4.65 (s, 1H, H‐1), 4.58 (d, ^2^ *J*_H,H_=11.7 Hz, 1H, H^Bn^), 4.09–4.04 (m, 2H, H‐2 & H‐5), 3.84 (ddd, ^3^ *J*_4,F_=27.2 Hz, ^*3*^ *J*_4,5_=9.8 Hz, ^*3*^ *J*_4,3_=2.9 Hz, 1H, H‐4), 3.85–3.82 (m, 2H, H‐6a & H‐6b), 3.40 (s, 3H, OCH_3_), 2.36 (d, ^3^ *J*_OH,2_=7.2 Hz, 1H, OH‐2), 1.97 (dd, ^3^ *J*_OH,6_=6.6 Hz, ^3^ *J*_OH,6_=6.0 Hz, 1H, OH‐6); ^13^C NMR (151 MHz, CDCl_3_) δ=137.71 (C^Ar^), 128.69 (2 CH^Ar^), 128.21 (CH^Ar^), 128.14 (2 CH^Ar^), 101.30 (C‐1), 87.03 (d, ^1^ *J*_3,F_=183.8 Hz, C‐3), 71.54 (CH_2_^Bn^) 70.25 (d, ^2^ *J*_4,F_=16.8 Hz, C‐4), 68.98 (d, ^2^ *J*_2,F_=25.3 Hz, C‐2), 67.26 (d, ^3^ *J*_5,F_=2.9 Hz, C‐5), 62.19 (C‐6), 55.74 (OCH_3_); ^19^F{^1^H} NMR (659 MHz, CDCl_3_) δ=−207.40 (s); ^19^F NMR (659 MHz, CDCl_3_) δ=−207.39 (ddd, ^2^ *J*_F,3_=49.1 Hz, ^3^ *J*_F,4_=27.2 Hz, ^3^ *J*_F,2_=6.5 Hz); HRMS (ESI+): *m/z* calc. for C_14_H_19_FO_5_Na^+^ [M+Na]^+^: 309.1109, found: 309.1115.

**Methyl 2,4,6‐tri‐*O*‐benzyl‐3‐deoxy‐3‐fluoro‐α‐d–*altro*‐pyranoside (5)** Methyl 4‐*O*‐benzyl‐3‐deoxy‐3‐fluoro‐α‐d‐*altro*‐pyranoside (**4**, 2.93 g, 10.22 mmol, 1 equiv.) was dissolved in dry DMF (29.2 mL) under an argon atmosphere and cooled to 0 °C. Sodium hydride (90 % purity, 817 mg, 30.65 mmol, 3 equiv.) was added in small portions and the reaction mixture was stirred for 30 min at 0 °C. Benzyl bromide (5.24 g, 3.6 mL, 30.65 mmol, 3 equiv.) was then added dropwise and the reaction mixture was stirred at room temperature for 16 h. Subsequently, methanol (45 mL) was added at 0 °C, followed by water (45 mL) and EA (45 mL). The biphasic mixture was allowed to come to room temperature, the layers were separated, and the aq. layer was extracted twice with EA. The combined org. layers were washed twice with water and once with brine. The org. layer was dried (MgSO_4_), filtered and evaporated. The crude residue was purified by MPLC (100 g silica gel, 5–31 % EA in *n‐*heptane) yielding methyl 2,4,6‐tri‐*O*‐benzyl‐3‐deoxy‐3‐fluoro‐α‐d‐*altro*‐pyranoside (**5**) as a colorless oil (3.824 g, 80 %); *R*_f_=0.26 (*n‐*heptane/EA 4 : 1, UV & CAM); $[{\alpha ]}_{D}^{20}$ =+47.9 (c=1.0 in CHCl_3_); ^1^H NMR (700 MHz, CDCl_3_) δ=7.36‐7.26 (m, 15H, 15 H^Ar^), 4.79 (dt, ^2^ *J*_3,F_=49.1 Hz, ^3^ *J*_3,2_=3.1 Hz, ^3^ *J*_3,4_=2.7 Hz, 1H, H‐3), 4.74 (s, 1H, H‐1), 4.67 (d, ^2^ *J*_H,H_=12.1 Hz, 1H, H^Bn^), 4.65 (d, ^2^ *J*_H,H_=12.1 Hz, 1H, H^Bn^), 4.61 (d, ^2^ *J*_H,H_=11.6 Hz, 1H, H^Bn^), 4.55 (d, ^2^ *J*_H,H_=11.4 Hz, 1H, H^Bn^), 4.53 (d, ^2^ *J*_H,H_=11.4 Hz, 1H, H^Bn^), 4.51 (d, ^2^ *J*_H,H_=11.6 Hz, 1H, H^Bn^), 4.15 (ddd, ^3^ *J*_5,4_=9.4 Hz, ^3^ *J*_5,6a_=4.0 Hz, ^3^ *J*_5,6b_=2.6 Hz, 1H, H‐5), 3.90 (ddd, ^3^ *J*_4,F_=27.1 Hz, ^3^ *J*_4,5_=9.4 Hz, ^3^ *J*_4,3_=2.7 Hz, 1H, H‐4), 3.78 (dd, ^3^ *J*_2,F_=7.4 Hz, ^3^ *J*_2,3_=3.1 Hz, 1H, H‐2), 3.76 (dd, ^2^ *J*_6a,6b_=10.9 Hz, ^3^ *J*_6a,5_=4.0 Hz, 1H, H‐6a), 3.73 (dd, ^2^ *J*_6b,6a_=10.9 Hz, ^3^ *J*_6b,5_=2.6 Hz, 1H, H‐6b), 3.39 (s, 3H, OCH_3_); ^13^C NMR (176 MHz, CDCl_3_) δ=138.42 (C^Ar^), 137.99 (C^Ar^), 137.57 (C^Ar^), 128.66 (2 CH^Ar^), 128.52 (2 CH^Ar^), 128.46 (2 CH^Ar^), 128.18 (CH^Ar^), 128.09 (2 CH^Ar^), 127.95 (2 CH^Ar^), 127.94 (CH^Ar^), 127.88 (2 CH^Ar^), 127.68 (CH^Ar^), 99.51 (C‐1), 86.56 (d, ^1^ *J*_3,F_=183.1 Hz, C‐3), 75.74 (d, ^2^ *J*_2,F_=24.5 Hz, C‐2), 73.65 (CH_2_^Bn^), 72.94 (CH_2_^Bn^), 71.82 (CH_2_^Bn^), 71.41 (d, ^2^ *J*_4,F_=16.6 Hz, C‐4), 69.34 (C‐6), 67.01 (d, ^3^ *J*_5,F_=3.8 Hz, C‐5), 55.51 (OCH_3_); ^19^F{^1^H} NMR (659 MHz, CDCl_3_) δ=−207.65 (s); ^19^F NMR (659 MHz, CDCl_3_) δ=−207.65 (ddd, ^2^ *J*_F,3_=49.1 Hz, ^3^ *J*_F,4=_27.1 Hz, ^3^ *J*_F,2=_7.8 Hz); HRMS (ESI+): *m/z* calc. for C_28_H_31_FO_5_Na^+^ [M+Na]^+^: 489.2048, found: 489.2051.

**2,4,6‐Tri‐*O*‐benzyl‐3‐deoxy‐3‐fluoro‐d–altrose (6)** was synthesized following a protocol by Matwiejuk *et al*.^[30]^ Methyl 2,4,6‐tri‐*O*‐benzyl‐3‐deoxy‐3‐fluoro‐α‐d‐*altro*‐pyranoside (**5**, 3.804 g, 8.15 mmol, 1 equiv.) was dissolved in acetic acid (81.5 mL) and 1 M aq. H_2_SO_4_ (18.5 mL, 81.53 mmol, 10 equiv.) was added dropwise under stirring. The flask was equipped with a reflux condenser, heated to 100 °C and stirred for 15 h. The reaction mixture was then cooled to room temperature and ice‐cold water (300 mL) was slowly added. The mixture was extracted thrice with CH_2_Cl_2_ and the combined org. layers were washed twice with a sat. aq. NaHCO_3_‐sol. and once with brine. The org. layer was dried (MgSO_4_), filtered and evaporated. The crude residue was purified by MPLC (100 g silica gel, 0–4 % EA in CH_2_Cl_2_) yielding 2,4,6‐tri‐*O*‐benzyl‐3‐deoxy‐3‐fluoro‐d‐altrose (**6**) as a colorless oil and as a mixture of anomers (α:β=1 : 4) (1.77 g, 48 %); *R*_f_=0.14 (*n‐*heptane/EA 4 : 1, UV & CAM); $[{\alpha ]}_{D}^{20}$ =+21.5 (c=1.0 in CHCl_3_); β‐Pyranose: ^1^H NMR (600 MHz, CDCl_3_) δ=7.39‐7.22 (m, 15H, H^Ar^), 5.01 (dt, ^3^ *J*_1,OH_=11.7 Hz, ^3^ *J*_1,2_=^4^ *J*_1,F_=2.0 Hz, 1H, H‐1), 4.74 (ddd, ^2^ *J*_3,F_=48.7 Hz, ^3^ *J*_3,2_=4.0 Hz, ^3^ *J*_3,4_=2.2 Hz, 1H, H‐3), 4.68 (d, ^2^ *J*_H,H_=12.1 Hz, 1H, H^Bn^), 4.64 (d, ^2^ *J*_H,H_=12.1 Hz, 1H, H^Bn^), 4.56 (d, ^2^ *J*_H,H_=12.0 Hz, 1H, H^Bn^), 4.54 (d, ^2^ *J*_H,H_=12.0 Hz, 1H, H^Bn^), 4,47 (s, 2H, CH_2_^Bn^), 3.94 (dd, ^3^ *J*_5,4_=9.5 Hz, ^3^ *J*_5,6_=1.5 Hz 1H, H‐5), 3.86 (ddd, ^3^ *J*_4,F_=29.0 Hz, ^3^ *J*_4,5_=9.5 Hz, ^3^ *J*_4,3_=2.2 Hz, 1H, H‐4), 3.77‐3.70 (m, 3H, H‐2, H‐6a & H‐6b), 3.57 (d, ^3^ *J*_1,OH_=11.7 Hz, 1H, OH); ^13^C NMR (151 MHz, CDCl_3_) δ=138.30 (C^Ar^), 137.71 (C^Ar^), 137.23 (C^Ar^), 128.90 (2 CH^Ar^), 128.62 (CH^Ar^), 128.60 (2 CH^Ar^), 128.48 (2 CH^Ar^), 128.30 (2 CH^Ar^), 128.24 (2 CH^Ar^), 128.12 (CH^Ar^), 128.09 (2 CH^Ar^), 127.75 (CH^Ar^), 91.89 (C‐1), 86.77 (d, ^1^ *J*_3,F_=178.7 Hz, C‐3), 76.05 (d, ^2^ *J*_2,F_=25.9 Hz, C‐2), 74.10 (CH_2_^Bn^), 73.78 (CH_2_^Bn^), 72.37 (d, ^3^ *J*_5,F_=3.6 Hz, C‐5), 72.15 (CH_2_^Bn^), 71.20 (d, ^2^ *J*_4,F_=16.5 Hz, C‐4), 69.15 (C‐6); ^19^F{^1^H} NMR (659 MHz, CDCl_3_) δ=−209.84 (s); ^19^F NMR (659 MHz, CDCl_3_) δ=−209.84 (dd, ^2^ *J*_F,3_=48.7 Hz, ^3^ *J*_F,4_=29.1 Hz); α‐Pyranose: ^1^H NMR (600 MHz, CDCl_3_) δ=7.38‐7.22 (m, 15H, H^Ar^), 5.20 (d, ^3^ *J*_1,OH_=7.9 Hz, 1H, H‐1), 4.84 (ddd, ^2^ *J*_3,F_=49.3 Hz, ^3^ *J*_3,2_=4.0 Hz, ^3^ *J*_3,4_=2.6 Hz, 1H, H‐3), 4.68 (d, ^2^ *J*_H,H_=12.1 Hz, 1H, H^Bn^), 4.64 (d, ^2^ *J*_H,H_=12.1 Hz, 1H, H^Bn^), 4.60 (d, ^2^ *J*_H,H_=11.8 Hz, 1H, H^Bn^), 4.56 (d, ^2^ *J*_H,H_=11.8 Hz, 1H, H^Bn^), 4.54 (d, ^2^ *J*_H,H_=12.1 Hz, 1H, H^Bn^), 4.53 (d, ^2^ *J*_H,H_=12.1 Hz, 1H, H^Bn^), 4.28 (dt, ^3^ *J*_5,4_=8.6 Hz, ^3^ *J*_5,6a_=^3^ *J*_5,6b_=4.2 Hz, 1H, H‐5), 3.94 (ddd, ^3^ *J*_4,F_=27.1 Hz, ^3^ *J*_4,5_=8.6 Hz, ^3^ *J*_4,3_=2.6 Hz, 1H, H‐4), 3.82 (ddd, ^3^ *J*_2,F_=8.0 Hz, ^3^ *J*_2,3_=4.0 Hz, ^3^ *J*_2,1_=0.9 Hz, 1H, H‐2), 3.77‐3.69 (m, 2H, H‐6a & H‐6b), 3.27 (dd, ^3^ *J*_OH,1_=7.9 Hz, *J*_OH,F_=5.2 Hz, 1H, OH); ^13^C NMR (151 MHz, CDCl_3_) δ=138.26 (C^Ar^), 137.68 (C^Ar^), 137.43 (C^Ar^), 128.70 (2 CH^Ar^), 128.62 (CH^Ar^), 128.60 (2 CH^Ar^), 128.50 (2 CH^Ar^), 128.24 (CH^Ar^), 128.17 (2 CH^Ar^), 128.01 (2 CH^Ar^), 127.95 (2 CH^Ar^), 127.77 (CH^Ar^), 93.29 (C‐1), 88.40 (d, ^1^ *J*_3,F_=179.2 Hz, C‐3), 75.94 (d, ^2^ *J*_2,F_=22.5 Hz, C‐2), 73.73 (CH_2_^Bn^), 72.80 (CH_2_^Bn^), 72.20 (CH_2_^Bn^), 71.60 (d, ^2^ *J*_4,F_=16.4 Hz, C‐4), 69.35 (C‐6), 67.80 (d, ^3^ *J*_5,F_=4.4 Hz, C‐5); ^19^F{^1^H} NMR (659 MHz, CDCl_3_) δ=−205.48 (s); ^19^F NMR (659 MHz, CDCl_3_) δ=−205.48 (dddd, ^*2*^ *J*_F,3_=49.3 Hz, ^*3*^ *J*_F,4_=27.3 Hz, ^*3*^ *J*_F,2_=7.0 Hz, *J*_F,OH=_5.2 Hz); HRMS (ESI+): *m/z* calc. for C_27_H_29_FO_5_Na^+^ [M+Na]^+^: 475.1891, found: 475.1881.

### 2,4,6‐Tri‐O‐benzyl‐3‐deoxy‐3‐fluoro‐d–altrono‐1,5‐lactone (7)

2,4,6‐Tri‐*O*‐benzyl‐3‐deoxy‐3‐fluoro‐d‐altrose (**6**, 1.745 g, 3.86 mmol, 1 equiv.) was dissolved in dry CH_2_Cl_2_ (44.8 mL) under argon. Dess–Martin‐Periodinane (DMP, 3.271 g, 7.99 mmol, 2 equiv.) was added and the mixture was stirred for 1 h at room temperature. Then, the reaction was quenched by the addition of a sat. aq. NaHCO_3_‐sol. (160 mL) and a sat. aq. Na_2_S_2_O_3_‐sol. (160 mL) was added. The mixture was extracted thrice with CH_2_Cl_2_, the combined org. layers were dried (MgSO_4_), filtered and evaporated. The crude residue was purified by MPLC (100 g silica gel, 4–40 % EA in *n‐*heptane) yielding 2,4,6‐tri‐*O*‐benzyl‐3‐deoxy‐3‐fluoro‐d‐*altrono*‐1,5‐lactone (**7**) as a colorless oil (1.663 g, 96 %); *R*_f_=0.34 (*n‐*heptane/EA 4 : 1, UV & CAM); $[{\alpha ]}_{D}^{20}$ =−25.2 (c=1.0 in CHCl_3_); ^1^H NMR (600 MHz, CDCl_3_) δ=7.41‐7.26 (m, 15H, H^Ar^), 5.14 (ddd, ^2^ *J*_3,F_=49.8 Hz, ^3^ *J*_3,2_=7.5 Hz, ^3^ *J*_3,4_=3.1 Hz, 1H, H‐3), 5.04 (d, ^2^ *J*_H,H_=11.5 Hz, 1H, H^Bn^), 4.74 (d, ^2^ *J*_H,H_=11.5 Hz, 1H, H^Bn^), 4.74 (d, ^2^ *J*_H,H_=11.7 Hz, 1H, H^Bn^), 4.62 (d, ^2^ *J*_H,H_=11.7 Hz, 1H, H^Bn^), 4.58 (d, ^2^ *J*_H,H_=11.9 Hz, 1H, H^Bn^), 4.58 (m, 1H, H‐5), 4.48 (d, ^2^ *J*_H,H_=11.9 Hz, 1H, H^Bn^), 4.44 (dd, ^3^ *J*_2,F_=14.5 Hz, ^3^ *J*_2,3_=7.5 Hz, 1H, H‐2), 4.17 (ddd, ^3^ *J*_4,F_=14.0 Hz, ^3^ *J*_4,5_=4.2 Hz, ^3^ *J*_4,3_=3.1 Hz, 1H, H‐4), 3.67 (dd, ^2^ *J*_6a,6b_=11.0 Hz, ^3^ *J*_6a,5_=3.8 Hz, 1H, H‐6a), 3.64 (dd, ^2^ *J*_6b,6a_=11.0 Hz, ^3^ *J*_6b,5_=2.7 Hz, 1H, H‐6b); ^13^C NMR (151 MHz, CDCl_3_) δ=168.51 (d, ^3^ *J*_1,F_=10.4 Hz, C‐1), 137.23 (C^Ar^), 137.16 (2 C^Ar^), 128.71 (2 CH^Ar^), 128.67 (2 CH^Ar^), 128.58 (2 CH^Ar^), 128.39 (2 CH^Ar^), 128.33 (CH^Ar^), 128.19 (CH^Ar^), 128.10 (3 CH^Ar^), 127.90 (2 CH^Ar^), 88.84 (d, ^1^ *J*_3,F_=186.5 Hz, C‐3), 78.17 (d, ^3^ *J*_5,F_=6.5 Hz, C‐5), 74.83 (d, ^2^ *J*_2,F_=24.3 Hz, C‐2), 74.05 (CH_2_^Bn^), 73.94 (CH_2_^Bn^), 73.34 (CH_2_^Bn^), 73.30 (d, ^2^ *J*_4,F_=17.3 Hz, C‐4), 68.81 (C‐6); ^19^F{^1^H} NMR (659 MHz, CDCl_3_) δ=−199.38 (s); ^19^F NMR (659 MHz, CDCl_3_) δ=−199.38 (dt, ^2^ *J*_F,3_=49.8 Hz, ^3^ *J*_F,2_=14.5 Hz, ^3^ *J*_F,4_=14.0 Hz); HRMS (ESI+): *m/z* calc. for C_27_H_27_O_5_FNa^+^ [M+Na]^+^: 473.1735, found: 473.1739.

**2,6‐Anhydro‐3,5,7‐tri‐*O*‐benzyl‐1,4‐dideoxy‐4‐fluoro‐d–*altro*‐hept‐1‐enitol (8)** was synthesized following a protocol by Waschke *et al*.^[32]^ 2,4,6‐Tri‐*O*‐benzyl‐3‐deoxy‐3‐fluoro‐d‐*altrono*‐1,5‐lactone (**7**, 1.64 g, 3.64 mmol, 1 equiv.) was dissolved in dry toluene (18.2 mL) under an argon atmosphere and the flask was wrapped in aluminum foil. Bis(cyclopentadienyl)dimethyltitanium (**S2**, 0.5 M in dry toluene, 18.2 mL, 9.10 mmol, 2.5 equiv.) was added dropwise and the flask was equipped with a reflux condenser. The mixture was stirred for 16 h at 70 °C before being cooled to room temperature again. The dark red solution was diluted with *n‐*heptane (60 mL) and stirring was continued for 30 min. The resulting orange suspension was then filtered through a pad of Celite^®^ and the filtrate was evaporated. The crude residue was purified by MPLC (100 g silica gel, 2–20 % EA in *n‐*heptane) yielding 2,6‐anhydro‐3,5,7‐tri‐*O*‐benzyl‐1,4‐dideoxy‐4‐fluoro‐d‐*altro*‐hept‐1‐enitol (**8**) as an orange oil (1.215 g, 74 %); *R*_f_=0.32 (*n‐*heptane/EA 9 : 1, UV & CAM); $[{\alpha ]}_{D}^{20}$ =+48.0 (c 1.0 in CHCl_3_); ^1^H NMR (700 MHz, CDCl_3_) δ=7.36‐7.26 (m, 15H, H^Ar^), 4.95 (s, 1H, H‐1a), 4.91 (ddd, ^2^ *J*_4,F_=50.6 Hz, ^3^ *J*_4,3_=4.6 Hz, ^3^ *J*_4,5_=2.1 Hz, 1H, H‐4), 4.70 (d, ^2^ *J*_H,H_=11.9 Hz, 1H, H^Bn^), 4.69 (d, ^2^ *J*_H,H_=11.9 Hz, 1H, H^Bn^), 4.66 (d, ^2^ *J*_H,H_=11.5 Hz, 1H, H^Bn^), 4.56 (s, 1H, H‐1b), 4.55 (d, ^2^ *J*_H,H_=11.5 Hz, 1H, H^Bn^), 4.54 (d, ^2^ *J*_H,H_=11.6 Hz, 1H, H^Bn^), 4.37 (d, ^2^ *J*_H,H_=11.6 Hz, 1H, H^Bn^), 4.17 (ddd, ^3^ *J*_5,F_=28.4 Hz, ^3^ *J*_5,6_=9.5 Hz, ^3^ *J*_5,4_=2.1 Hz, 1H, H‐5), 4.10 (dd, ^3^ *J*_3,F_=5.7 Hz, ^3^ *J*_3,4_=4.6 Hz, 1H, H‐3), 4.06 (ddd, ^3^ *J*_6,5_=9.5 Hz, ^3^ *J*_6,7a_=3.7 Hz, ^3^ *J*_6,7b_=2.5 Hz, 1H, H‐6), 3.82 (dd, ^2^ *J*_7a,7b_=11.1 Hz, ^3^ *J*_7a,6_=3.7 Hz, 1H, H‐7a), 3.79 (dd, ^2^ *J*_7b,7a_=11.1 Hz, ^3^ *J*_7b,6_=2.5 Hz, 1H); ^13^C NMR (176 MHz, CDCl_3_) δ=153.32 (C‐2), 138.38 (C^Ar^), 137.77 (C^Ar^), 137.71 (C^Ar^), 128.57 (2 CH^Ar^), 128.55 (2 CH^Ar^), 128.48 (2 CH^Ar^), 128.10 (2 CH^Ar^), 128.01 (CH^Ar^), 127.95 (2 CH^Ar^), 127.93 (CH^Ar^), 127.88 (2 CH^Ar^), 127.70 (CH^Ar^), 101.91 (C‐1), 87.02 (d, ^1^ *J*_4,F_=178.1 Hz, C‐4), 76.34 (d, ^3^ *J*_6,F_=3.0 Hz, C‐6), 75.25 (d, ^2^ *J*_3,F_=28.6 Hz, C‐3), 73.67 (CH_2_^Bn^), 72.14 (CH_2_^Bn^), 71.16 (d, ^2^ *J*_5,F_=16.6 Hz, C‐5), 70.06 (CH_2_^Bn^), 69.24 (C‐7); ^19^F{^1^H} NMR (659 MHz, CDCl_3_) δ=−206.16 (s); ^19^F NMR (659 MHz, CDCl_3_) δ=−206.16 (ddd, ^2^ *J*_F,4_=50.6 Hz, ^3^ *J*_F,5_=28.4 Hz, ^3^ *J*_F,3_=5.3 Hz); HRMS (ESI+): *m/z* calc. for C_28_H_29_O_4_FNa^+^ [M+H_2_O+Na]^+^: 471.1942, found: 471.1936.

**3,5,7‐Tri‐*O*‐benzyl‐4‐deoxy‐4‐fluoro‐d–*glycero*‐d–*arabino*‐hept‐2‐ulo‐pyranose (9)** was synthesized following a protocol by Leshch *et al*.^[34]^ 2,6‐Anhydro‐3,5,7‐tri‐*O*‐benzyl‐1,4‐dideoxy‐4‐fluoro‐d‐*altro*‐hept‐1‐enitol (**8**, 1.202 g, 2.68 mmol, 1 equiv.) was dissolved in a 4 : 1 (v/v) mixture of acetone and water (53.6 mL) followed by addition of *N*‐methylmorpholine‐*N*‐oxide (NMO, 628 mg, 5.36 mmol, 2 equiv.) and potassium osmate dihydrate (49 mg, 0.13 mmol, 0.05 equiv.). The reaction mixture was stirred at room temperature for 16 h, diluted with EA and washed with water and brine. The organic layer was dried (MgSO_4_), filtered and evaporated. The crude residue was purified by MPLC (100 g silica gel, 12–84 % EA in *n‐*heptane) yielding 3,5,7‐tri‐*O*‐benzyl‐4‐deoxy‐4‐fluoro‐d‐*glycero*‐α/β‐d‐*arabino*‐hept‐2‐ulopyranose (**9**) as a brown oil (1.126 g, 87 %); *R*_f_=0.28 (*n‐*heptane/EA 1 : 1, UV & CAM); $[{\alpha ]}_{D}^{20}$ =+32.4 (c=1.0 in CHCl_3_); α‐Pyranose ( ^5^ *C*_2_ ): ^1^H NMR (700 MHz, CDCl_3_) δ=7.37‐7.24 (m, 15H, H^Ar^), 4.78 (ddd, ^2^ *J*_4,F_=49.5 Hz, ^3^ *J*_4,3_=3.7 Hz, ^3^ *J*_4,5_=2.5 Hz, 1H, H‐4), 4.65 (d, ^2^ *J*_H,H_=11.9 Hz, 1H, H^Bn^), 4.59 (d, ^2^ *J*_H,H_=11.9 Hz, 1H, H^Bn^), 4.55 (d, ^2^ *J*_H,H_=11.6 Hz, 1H, H^Bn^), 4.53 (d, ^2^ *J*_H,H_=11.6 Hz, 1H, H^Bn^), 4.53 (d, ^2^ *J*_H,H_=11.6 Hz, 1H, H^Bn^), 4.52 (d, ^2^ *J*_H,H_=11.6 Hz, 1H, H^Bn^), 4.25 (ddd, ^3^ *J*_6,5_=9.8 Hz, ^3^ *J*_6,7a_=4.4 Hz, ^3^ *J*_6,7b_=2.1 Hz, 1H, H‐6), 3.90 (ddd, ^3^ *J*_5,F_=30.1 Hz, ^3^ *J*_5,6_=9.8 Hz, ^3^ *J*_5,4_=2.5 Hz, 1H, H‐5), 3.80 (dd, ^3^ *J*_3,F_=5.7 Hz, ^3^ *J*_3,4_=3.7 Hz, 1H, H‐3), 3.80 (d, *J*_OH,F_=6.9 Hz, 1H, OH‐2), 3.78 (dd, ^2^ *J*_7a,7b_=11.1 Hz, ^3^ *J*_7a,6_=4.4 Hz, 1H, H‐7a), 3.71 (dd, ^2^ *J*_7b,7a_=11.1 Hz, ^3^ *J*_7b,6=_2.1 Hz, 1H, H‐7b), 3.69 (dd, ^2^ *J*_1a,1b_=11.1 Hz, ^3^ *J*_1a,OH_=3.5 Hz, 1H, H‐1a), 3.55 (t, ^2^ *J*_1b,1a_=^3^ *J*_1b,OH_=11.1 Hz, 1H, H‐1b), 2.01 (dd, ^3^ *J*_OH,1b_=11.1 Hz, ^3^ *J*_OH,1a_=3.5 Hz, 1H, OH‐1); ^13^C NMR (176 MHz, CDCl_3_) δ=138.39 (C^Ar^), 137.75 (C^Ar^), 137.24 (C^Ar^), 128.77 (2 CH^Ar^), 128.61 (2 CH^Ar^), 128.48 (2 CH^Ar^), 128.45 (CH^Ar^), 128.29 (4 CH^Ar^), 128.13 (CH^Ar^), 128.05 (2 CH^Ar^), 127.76 (CH^Ar^), 96.91 (C‐2), 87.22 (d, ^1^ *J*_4,F_=181.0 Hz, C‐4), 75.35 (d, ^2^ *J*_3,F_=21.5 Hz, C‐3), 73.77 (CH_2_^Bn^), 73.68 (CH_2_^Bn^), 71.99 (CH_2_^Bn^), 70.81 (d, ^2^ *J*_5,F_=16.5 Hz, C‐5), 69.21 (C‐7), 67.85 (d, ^3^ *J*_6,F_=3.4 Hz, C‐6), 65.77 (C‐1); ^19^F{^1^H} NMR (659 MHz, CDCl_3_) δ=−204.33 (s); ^19^F NMR (659 MHz, CDCl_3_) δ= −204.33 (ddt, ^2^ *J*_F,4_=49.5 Hz, ^3^ *J*_F,5_=30.1 Hz, ^3^ *J*_F,3_=*J*_F,OH_=6.9 Hz); β‐Pyranose ( ^2^ *C*_5_ ): ^1^H NMR (700 MHz, CDCl_3_) δ=7.37‐7.24 (m, 15H, H^Ar^), 5.00 (ddd, ^2^ *J*_4,F_=49.3 Hz, ^3^ *J*_4,3_=7.7 Hz, ^3^ *J*_4,5_=3.2 Hz, 1H, H‐4), 4.80 (d, ^2^ *J*_H,H_=11.0 Hz, 1H, H^Bn^), 4.68 (d, ^2^ *J*_H,H_=11.9 Hz, 1H, H^Bn^), 4.66 (d, ^2^ *J*_H,H_=11.0 Hz, 1H, H^Bn^), 4.62 (d, ^2^ *J*_H,H_=11.9 Hz, 1H, H^Bn^), 4.55 (d, ^2^ *J*_H,H_=12.3 Hz, 1H, H^Bn^), 4.50 (d, ^2^ *J*_H,H_=12.3 Hz, 1H, H^Bn^), 4.15 (dq, ^3^ *J*_6,7a_=6.2 Hz, ^3^ *J*_6,5_=^3^ *J*_6,7b_=^4^ *J*_6,F_=4.2 Hz, 1H, H‐6), 4.09 (dd, ^3^ *J*_3,F_=10.1 Hz, ^3^ *J*_3,4_=7.7 Hz, 1H, H‐3), 4.07 (ddd, ^3^ *J*_5,F_=14.9 Hz, ^3^ *J*_5,6_=4.2, ^3^ *J*_5,4_=3.2 Hz, 1H, H‐5), 3.79 (s, 1H, OH‐2), 3.68 (dd, ^2^ *J*_7a,7b_=10.4 Hz, ^3^ *J*_7a,6_=6.2 Hz, 1H, H‐7a), 3.64 (dd, ^2^ *J*_7b,7a_=10.4 Hz, ^3^ *J*_7b,6_=4.2 Hz, 1H, H‐7b), 3.58 (dd, ^2^ *J*_1a,1b_=11.6 Hz, ^3^ *J*_1a,OH_=7.2 Hz, 1H, H‐1a), 3.57 (dd, ^2^ *J*_1b,1a_=11.6 Hz, ^3^ *J*_1b,OH_=6.9 Hz, 1H, H‐1b), 1.96 (t, ^3^ *J*_OH,1a_=7.2 Hz, ^3^ *J*_OH,1b_=6.9 Hz, 1H, OH‐1); ^13^C NMR (176 MHz, CDCl_3_) δ=137.96 (C^Ar^), 137.73 (C^Ar^), 137.43 (C^Ar^), 128.72 (2 CH^Ar^), 128.64 (2 CH^Ar^), 128.57 (2 CH^Ar^), 128.56 (2 CH^Ar^), 128.38 (CH^Ar^), 128.03 (CH^Ar^), 128.02 (2 CH^Ar^), 127.99 (CH^Ar^), 127.93 (2 CH^Ar^), 98.25 (d, ^3^ *J*_2,F_=6.4 Hz, C‐2), 89.76 (d, ^1^ *J*_4,F_=184.6 Hz, C‐4), 74.87 (d, ^4^ *J*_C,F_=2.1 Hz, CH_2_^Bn^), 74.56 (d, ^2^ *J*_3,F_=20.0 Hz, C‐3), 74.41 (d, ^3^ *J*_6,F_=5.8 Hz, C‐6), 74.17 (d, ^2^ *J*_5,F_=15.8 Hz, C‐5), 73.59 (CH_2_^Bn^), 72.75 (d, ^4^ *J*_C,F_=1.8 Hz, CH_2_^Bn^), 70.29 (C‐7), 65.32 (d, ^4^ *J*_1,F_=3.4 Hz, C‐1); ^19^F{^1^H} NMR (659 MHz, CDCl_3_) δ=−205.36 (s); ^19^F NMR (659 MHz, CDCl_3_) δ=−205.36 (dddd, ^2^ *J*_F,4_=49.3 Hz, ^3^ *J*_F,5_=14.2 Hz, ^3^ *J*_F,3_=10.4, ^4^ *J*_F,6_=4.2 Hz); HRMS (ESI+): *m/z* calc. for C_28_H_31_O_6_FNa^+^ [M+Na]^+^: 505.1997, found: 505.1998.

### 4‐Deoxy‐4‐fluoro‐d–sedoheptulose (4DFS)

3,5,7‐Tri‐*O*‐benzyl‐4‐deoxy‐4‐fluoro‐d‐*glycero*‐α/β‐d‐*arabino*‐hept‐2‐ulopyranose (**9**, 170 mg, 0.35 mmol, 1 equiv.) was dissolved in methanol (5 mL) and Pd/C (10 %, 60 mg, 0.04 mmol, 0.1 equiv.) was added. The black suspension was degassed (3 freezing/thawing cycles with liquid N_2_ under vacuum) and subsequently stirred at room temperature for 24 h (completion of the reaction was verified by absence of aromatic proton signals in *d_4_*‐methanol after drying a small portion of the reaction mixture in high vacuum). The mixture was filtered, evaporated, and purified by reversed‐phase chromatography (C_18_‐modified silica gel, H_2_O) yielding 4‐deoxy‐4‐fluoro‐d‐sedoheptulose (**4DFS**) as a colorless oil and as a mixture of α‐ and β‐furanoses and α‐ and β‐pyranoses in a 1.5 :4 : 4 : 1 ratio (60 mg, 80 %); *R*_f_=0.7 (1‐BuOH/acetone/H_2_O 5 : 4 : 1, CAM); $[{\alpha ]}_{D}^{20}$ =+23.4 (c=1.0 in H_2_O); β‐Furanose: ^1^H NMR (700 MHz, D_2_O) δ=5.17 (dt, ^2^ *J*_4,F_=56.4 Hz, ^3^ *J*_4,3_=6.1 Hz, ^3^ *J*_4,5_=5.1 Hz, 1H, H‐4), 4.42 (dd, ^3^ *J*_3,F_=22.7 Hz, ^3^ *J*_3,4_=6.1 Hz, 1H, H‐3), 4.00 (ddd, ^3^ *J*_5,F_=21.4 Hz, ^3^ *J*_5,6_=7.8 Hz, ^3^ *J*_5,4_=5.1 Hz, 1H, H‐5), 3.82 (ddd, ^3^ *J*_6,5_=7.8 Hz, ^3^ *J*_6,7b_=6.2 Hz, ^3^ *J*_6,7a_=3.3 Hz, 1H, H‐6), 3.76 (dd, ^2^ *J*_7a,7b_=11.9 Hz, ^3^ *J*_7a,6_=3.3 Hz, 1H, H‐7a), 3.62 (dd, ^2^ *J*_7b,7a_=11.9 Hz, ^3^ *J*_7b,6_=6.2 Hz, 1H, H‐7b), 3.59 (dd, ^2^ *J*_1a,1b_=12.1 Hz, ^5^ *J*_1a,F=_0.7 Hz, 1H, H‐1a), 3.55 (dd, ^2^ *J*_1b,1a_=12.1 Hz, ^5^ *J*_1b,F=_1.7 Hz, 1H, H‐1b); ^13^C NMR (176 MHz, D_2_O) δ=105.30 (d, ^3^ *J*_2,F_=10.0 Hz, C‐2), 101.15 (d, ^1^ *J*_4,F_=182.28 Hz, C‐4), 81.07 (d, ^2^ *J*_5,F_=24.4 Hz, C‐5), 77.11 (d, ^2^ *J*_3,F_=22.3 Hz, C‐3), 74.99 (d, ^3^ *J*_6,F_=3.6 Hz, C‐6), 65.04 (C‐1), 64.94 (C‐7); ^19^F{^1^H} NMR (659 MHz, D_2_O) δ=−192.63 (s); ^19^F NMR (659 MHz, D_2_O) δ=−192.63 (dt, ^2^ *J*_F,4_=56.4 Hz, ^3^ *J*_F,3_=^3^ *J*_F,5_=21.4 Hz); α‐Pyranose ( ^5^ *C*_2_ ): ^1^H NMR (700 MHz, D_2_O) δ=4.83 (dt, ^2^ *J*_4,F_=48.9 Hz, ^3^ *J*_4,3_=^3^ *J*_4,5_=3.2 Hz, 1H, H‐4), 4.10 (ddd, ^3^ *J*_6,5_=10.5 Hz, ^3^ *J*_6,7b_=5.8 Hz, ^3^ *J*_6,7a_=2.4 Hz, 1H, H‐6), 4.06 (dd, ^3^ *J*_3,F_=6.0 Hz, ^3^ *J*_3,4_=3.2 Hz, 1H, H‐3), 3.88 (ddd, ^3^ *J*_5,F_=31.1 Hz, ^3^ *J*_5,6_=10.5 Hz, ^3^ *J*_5,4_=3.2 Hz, 1H, H‐5), 3.87 (dd, ^2^ *J*_7a,7b_=12.2 Hz, ^3^ *J*_7a,6_=2.4 Hz, 1H, H‐7a), 3.75 (dd, ^2^ *J*_7b,7a_=12.2 Hz, ^3^ *J*_7b,6_=5.8 Hz, 1H, H‐7b), 3.70 (d, ^2^ *J*_1a,1b_=11.7 Hz, 1H, H‐1a), 3.53 (d, ^2^ *J*_1b,1a_=11.7 Hz, 1H, H‐1b); ^13^C NMR (176 MHz, D_2_O) δ=99.27 (C‐2), 93.43 (d, ^1^ *J*_4,F_=179.6 Hz, C‐4), 71.20 (d, ^3^ *J*_6,F_=2.4 Hz, C‐6), 70.00 (d, ^2^ *J*_3,F_=23.4 Hz, C‐3), 67.31 (C‐1), 65.67 (d, ^2^ *J*_5,F_=17.5 Hz, C‐5), 63.76 (C‐7); ^19^F{^1^H} NMR (659 MHz, D_2_O) δ=−202.12 (s); ^19^F NMR (659 MHz, D_2_O) δ=−202.12 (ddd, ^2^ *J*_F,4_=48.9 Hz, ^3^ *J*_F,5_=31.1 Hz, ^3^ *J*_F,3_=6.0 Hz); α‐Furanose: ^1^H NMR (700 MHz, D_2_O) δ=5.03 (ddd, ^2^ *J*_4,F_=52.9 Hz, ^3^ *J*_4,5_=3.8 Hz, ^3^ *J*_4,3_=2.2 Hz, 1H, H‐4), 4.33 (dd, ^3^ *J*_3,F_=16.2 Hz, ^3^ *J*_3,4_=2.2 Hz, 1H, H‐3), 4.24 (ddd, ^3^ *J*_5,F_=24.0 Hz, ^3^ *J*_5,6_=6.7 Hz, ^3^ *J*_5,4_=3.8 Hz, 1H, H‐5), 3.80 (td, ^3^ *J*_6,5_=^3^ *J*_6,7b_=6.7 Hz, ^3^ *J*_6,7a_=3.6 Hz, 1H, H‐6), 3.74 (ddd, ^2^ *J*_7a,7b_=12.0 Hz, ^3^ *J*_7a,6_=3.6 Hz, ^5^ *J*_7a,F_=0.4 Hz, 1H, H‐7a), 3.72 (d, ^2^ *J*_1a,1b_=12.0 Hz, 1H, H‐1a), 3.66 (d, ^2^ *J*_1b,1a_=12.0 Hz, 1H, H‐1b), 3.62 (dd, ^2^ *J*_7b,7a_=12.0 Hz, ^3^ *J*_7b,6_=6.7 Hz, 1H, H‐7b); ^13^C NMR (176 MHz, D_2_O) δ=108.28 (d, ^3^ *J*_2,F_=4.2 Hz, C‐2), 100.47 (d, ^1^ *J*_4,F_=183.0 Hz, C‐4), 84.08 (d, ^2^ *J*_5,F_=25.7 Hz, C‐5), 81.32 (d, ^2^ *J*_3,F_=23.1 Hz, C‐3), 73.73 (d, ^3^ *J*_6,F_=5.5 Hz, C‐6), 65.06 (C‐1), 65.02 (C‐7); ^19^F{^1^H} NMR (659 MHz, D_2_O) δ=−182.45 (s); ^19^F NMR (659 MHz, D_2_O) δ=−182.45 (ddd, ^2^ *J*_F,4_=52.9 Hz, ^3^ *J*_F,5_=24.0 Hz, ^3^ *J*_F,3=_16.2 Hz); β‐Pyranose ( ^2^ *C*_5_ ): ^1^H NMR (700 MHz, D_2_O) δ=4.93 (ddd, ^2^ *J*_4,F_=48.9 Hz, ^3^ *J*_4,3_=8.3 Hz, ^3^ *J*_4,5_=3.5 Hz, 1H, H‐4), 4.27 (dt, ^3^ *J*_5,F_=13.8 Hz, ^3^ *J*_5,4_=^3^ *J*_5,6_=3.5 Hz, 1H, H‐5), 4.14 (dd, ^3^ *J*_3,F_=10.9 Hz, ^3^ *J*_3,4_=8.3 Hz, 1H, H‐3), 3.99 (dddd, ^3^ *J*_6,7a_=6.3 Hz, ^3^ *J*_6,7b_=6.0 Hz, ^4^ *J*_6,F_=4.3 Hz, ^3^ *J*_6,5_=3.5 Hz, 1H, H‐6), 3.80 (dd, ^2^ *J*_7a,7b_=12.0 Hz, ^3^ *J*_7a,6_=6.3 Hz, 1H, H‐7a), 3.77 (dd, ^2^ *J*_7b,7a_=12.0 Hz, ^3^ *J*_7b,6_=6.0 Hz, 1H, H‐7b), 3.71 (dd, ^2^ *J*_1a,1b_=11.9 Hz, ^5^ *J*_1a,F_=1.0 Hz, 1H, H‐1a), 3.57 (dd, ^2^ *J*_1b,1a_=11.9 Hz, ^5^ *J*_1b,F_=1.9 Hz, 1H, H‐1b); ^13^C NMR (176 MHz, D_2_O) δ=101.58 (d, ^3^ *J*_2,F_=6.5 Hz, C‐2), 92.94 (d, ^1^ *J*_4,F_=178.3 Hz, C‐4), 80.52 (d, ^3^ *J*_6,F_=5.6 Hz, C‐6), 69.03 (d, ^2^ *J*_5,F_=16.6 Hz, C‐5), 68.64 (d, ^2^ *J*_3,F_=20.5 Hz, C‐3), 65.81 (d, ^5^ *J*_1,F_=3.4 Hz, C‐1), 64.74 (C‐7); ^19^F{^1^H} NMR (659 MHz, D_2_O) δ=−205.02 (s); ^19^F NMR (659 MHz, D_2_O) δ=−205.02 (dtd, ^2^ *J*_F,4_=48.9 Hz), ^3^ *J*_F,5_ =13.8 Hz, ^3^ *J*_F,3_=10.9 Hz, ^4^ *J*_F,6_=4.3 Hz); HRMS (ESI+): *m/z* calc. for C_7_H_13_O_6_FNa^+^ [M+Na]^+^: 235.0588, found: 235.0593.

### Synthesis of 3‐deoxy‐3‐fluoro‐d–sedoheptulose (3DFS)

**Methyl 4‐*O*‐benzyl‐2‐deoxy‐2‐fluoro‐α‐d–*altro*‐pyranoside (13 a) & methyl 6‐*O*‐benzyl‐2‐deoxy‐2‐fluoro‐α‐d–*altro*‐pyranoside (13 b)** were synthesized following a protocol by Shie *et al*.^[29]^ Methyl 4,6‐*O*‐benzyliden‐2‐deoxy‐2‐fluoro‐α‐d‐*altro*‐pyranoside[^25^, ^35^] (**12**, 151 mg, 0.53 mmol, 1 equiv.) was dissolved in a borane THF complex solution (1 M in THF, 2.66 mL, 0.04 mmol, 5 equiv.) under argon atmosphere at 0 °C. The resulting mixture was stirred for 10 min before Cu(OTf)_2_ (9.6 mg, 0.03 mmol, 0.05 equiv.) was added. After 2 h the reaction mixture was cooled to 0 °C and triethylamine (0.08 mL), followed by MeOH (0.95 mL) was added. The solvents were removed *in vacuo* and the residue was co‐evaporated with methanol. Purification via MPLC (25 g silica gel, 12–100 % EA in *n‐*heptane) gave the desired product as a clear oily mixture of methyl 4‐*O*‐benzyl‐2‐deoxy‐2‐fluoro‐α‐d‐*altro*‐pyranoside (**13 a**) and methyl 6‐*O*‐benzyl‐2‐deoxy‐2‐fluoro‐α‐d‐*altro‐*pyranoside (**13 b**) in a 1.35 : 1 ratio (141 mg, 93 %); *R*_f_=0.24 (*n‐*heptane/EA 1 : 1, UV & CAM); $[{\alpha ]}_{D}^{20}$ =+105.2 (c=1.0 in CHCl_3_); **13 b**: ^1^H NMR (700 MHz, CDCl_3_) δ=7.38‐7‐28 (m, 5H, H^Ar^), 4.87 (d, ^3^ *J*_1,F_=9.0 Hz, 1H, H‐1), 4.66 (ddd, ^3^ *J*_2,F_=44.5 Hz, ^3^ *J*_2,3_=3.5 Hz, ^3^ *J*_2,1_=1.6 Hz, 1H, H‐2), 4.63 (s, 2H, H^Bn^), 4.12 (dddd, ^3^ *J*_3,F_=9.8 Hz, ^3^ *J*_3,OH_=8.9 Hz, ^3^ *J*_3,4_=6.5 Hz, ^3^ *J*_3,2_=3.5 Hz, 1H, H‐3), 3.86 (ddd, ^3^ *J*_5,4_=8.4 Hz, ^3^ *J*_5,6b_=5.8 Hz, ^3^ *J*_5,6a_=2.8 Hz, 1H, H‐5), 3.85 (dd, ^2^ *J*_*6a,6b*_=11.5 Hz, ^3^ *J*_6a,5_ *=*2.8 Hz, 1H, H‐6a), 3.78 (dddd, ^3^ *J*_4,5_=8.4 Hz, ^3^ *J*_4,OH_=8.2 Hz, ^3^ *J*_4,3_=6.5 Hz, ^4^ *J*_4,F_=2.3 Hz, 1H, H‐4), 3.77 (dd, ^2^ *J*_*6b,6a*_=11.5 Hz, ^3^ *J*_6b,5_ *=*5.6 Hz, 1H, H‐6b), 3.47 (s, OCH_3_), 3.00 (d, ^3^ *J*_OH,3_=8.9 Hz, 1H, OH‐3), 2.68 (d, ^3^ *J*_OH,4_=8.2 Hz, 1H, OH‐4); ^13^C NMR (176 MHz, CDCl_3_) δ=137.94 (C^Ar^), 128.42 (2 CH^Ar^), 128.03 (CH^Ar^), 127.63 (2 CH^Ar^), 98.13 (d, ^3^ *J*_1,F_=31.0 Hz, C‐1), 86.18 (d, ^2^ *J*_2,F_=175.1 Hz, C‐2), 73.68 (CH_2_^Bn^), 71.38 (d, ^3^ *J*_4,F_=1.2 Hz, C‐4), 70.29 (C‐6), 67.93 (d, ^3^ *J*_3,F_=27.3 Hz, C‐3), 67.30 (C‐5), 55.76 (OCH_3_); ^19^F {^1^H} NMR (659 MHz, CDCl_3_) δ=−196.55 (s); ^19^F NMR (659 MHz, CDCl_3_) δ=−196.55 (m); **13 a**: ^1^H NMR (700 MHz, CDCl_3_) δ=7.38‐7.28 (m, 5H, H^Ar^), 4.82 (d, ^3^ *J*_1,F_=10.4 Hz, 1H, H‐1), 4.70 (d, ^2^ *J*_H,H_=11.4 Hz, 1H, H^Bn^), 4.66 (ddd, ^3^ *J*_2,F_=44.5 Hz, ^3^ *J*_2,3_=3.5 Hz, ^3^ *J*_2,1_=1.6 Hz, 1H, H‐2), 4.58 (d, ^2^ *J*_H.H_=11.4 Hz, 1H, H^Bn^), 4.28 (dddd, ^3^ *J*_3,F_=9.8 Hz, ^3^ *J*_3,OH_=6.5 Hz,^3^ *J*_3,4_=3.5 Hz, ^3^ *J*_3,2_=3.5 Hz, 1H, H‐3), 3.95 (ddd, ^3^ *J*_4,5_=9.9 Hz, ^3^ *J*_4,3_=3.5 Hz, ^4^ *J*_4,F_=3.5 Hz, 1H, H‐4), 3.89 (ddd, ^2^ *J*_6a,6b_=11.7 Hz, ^3^ *J*_6a,OH_=5.2 Hz, ^3^ *J*_6a,5_=2.9 Hz, 1H, H‐6a), 3.81 (ddd, ^2^ *J*_6b,6a_=11.7 Hz, ^3^ *J*_6b,OH_=7.9 Hz, ^3^ *J*_6b,5_=4.3 Hz, 1H, H‐6b), 3.81 (ddd, ^3^ *J*_5,4_=9.9 Hz, ^3^ *J*_5,6b_=4.3 Hz, ^3^ *J*_5,6a_=2.9 Hz, 1H, H‐5), 3.44 (s, OCH_3_), 2.77 (d, ^3^ *J*_OH,3_=6.5 Hz, 1H, OH‐3), 1.87 (dd, ^3^ *J*_OH,6b_=7.9 Hz, ^3^ *J*_OH,6a_=5.2 Hz, 1H, OH‐6); ^13^C NMR (176 MHz, CDCl_3_) δ=137.33 (C^Ar^), 128.61 (2 CH^Ar^), 128.18 (CH^Ar^), 127.72 (2 CH^Ar^), 98.49 (d, ^3^ *J*_1,F_=32.0 Hz, C‐1), 87.08 (d, ^2^ *J*_2,F_=172.8 Hz, C‐2), 71.44 (CH_2_^Bn^), 66.28 (C‐4), 65.56 (d, ^3^ *J*_3,F_=27.7 Hz, C‐3), 65.20 (C‐5), 62.28 (C‐6), 55.79 (OCH_3_); ^19^F {^1^H} NMR (700 MHz, CDCl_3_) δ=−195.55 (s); ^19^F NMR (700 MHz, CDCl_3_) δ=−195.55 (m); HRMS (+ESI): *m/z* calc. for C_14_H_19_O_5_FNa^+^ [M+Na]^+^: 309.1109, found: 309.1112.

### Methyl 3,4,6‐tri‐O‐benzyl‐2‐deoxy‐2‐fluoro‐α‐d–altro‐pyranoside (14)

Sodium hydride (35.6 mg, 1.49 mmol, 4 equiv.) was suspended in dry THF (1.6 mL) under argon atmosphere and cooled to 0 °C. Stirring was continued for 10 min after addition of a solution of the methyl 4‐*O*‐benzyl‐2‐deoxy‐2‐fluoro‐α‐d‐*altro*‐pyranoside and methyl 6‐*O*‐benzyl‐2‐deoxy‐2‐fluoro‐α‐d‐*altro*‐pyranoside mixture (**13 a** & **13 b**, 106.3 mg, 0.37 mmol, 1 equiv.) in THF (2.5 mL). Then, benzyl bromide (187 mg, 0.13 mL, 1.49 mmol, 3 equiv.) was added and the resulting mixture was stirred for 10 min at 0 °C followed by 15 h at room temperature. H_2_O (10 mL) was added, the two layers were separated, and the aqueous layer was extracted with EA (3×10 mL). The crude residue was purified via MPLC (25 g silica gel, 12–100 % EA in *n‐*heptane) to yield methyl 3,4,6‐tri‐*O*‐benzyl‐2‐deoxy‐2‐fluoro‐α‐d‐*altro*‐pyranoside as a colorless oil (**14**, 148 mg, 86 %); *R*_f_=0.78 (*n‐*heptane/EA 1 : 1, UV & CAM); $[{\alpha ]}_{D}^{20}$ =+85.7 (c=1.0 in CHCl_3_); ^1^H NMR (600 MHz, CDCl_3_) δ=7.35‐7.21 (m, 15 H, H^Ar^), 4.71 (d, ^2^ *J*_1,F_=13.3 Hz, 1H, H‐1), 4.71 (d, ^2^ *J*_H,H_=12.4 Hz, 1H, H^Bn^), 4.67 (ddd, ^2^ *J*_2,F_=45.7 Hz, ^3^ *J*_2,3_=4.3 Hz, ^3^ *J*_2,1_=1.3 Hz, 1H, H‐2), 4.65 (d, ^2^ *J*_H,H_=12.4 Hz, 1H, H^Bn^), 4.63 (d, ^2^ *J*_H,H_=12.3 Hz, 1H, H^Bn^), 4.53 (d, ^2^ *J*_H,H_=12.3 Hz, 1H, H^Bn^), 4.52 (d, ^2^ *J*_H,H_=11.8 Hz, 1H, H^Bn^), 4.46 (d, ^2^ *J*_H,H_=11.8 Hz, 1H, H^Bn^), 4.25 (ddd, ^3^ *J*_5,4_ *=*8.5 Hz, ^3^ *J*_5,6a_=4.2 Hz, ^3^ *J*_5,6b_ *=*2.8 Hz, 1H, H‐5), 3.95 (ddd, ^3^ *J*_3,F_ *=*8.2 Hz, ^3^ *J*_3,2_=4.3 Hz, ^3^ *J*_3,4_ *=*3.2 Hz, 1H, H‐3), 3.86 (ddd, ^3^ *J*_4,5_=8.5 Hz, ^3^ *J*_4,3_=3.2 Hz, ^4^ *J*_4,F_=3.1 Hz, 1H, H‐4), 3.73 (dd, ^2^ *J*_*6a,6b*_=10.8 Hz, ^3^ *J*_6a,5_ *=*4.2 Hz, 1H, H‐6a), 3.70 (dd, ^2^ *J*_6b,6a_=10.8 Hz, ^3^ *J*_6b,5_=2.8 Hz, 1H, H‐6b), 3.43 (s, OCH_3_); ^13^C NMR (151 MHz, CDCl_3_) δ=138.18 (C^Ar^), 138.06 (C^Ar^), 138.04 (C^Ar^), 128.34 (2 CH^Ar^), 128.31 (2 CH^Ar^), 128.30 (2 CH^Ar^), 127.79 (2 CH^Ar^), 127.78 (2 CH^Ar^), 127.70 (3 CH^Ar^), 127.65 (CH^Ar^), 127.53 (CH^Ar^), 99.34 (d, ^3^ *J*_1,F=_33.02 Hz, C‐1), 87.89 (d, ^*2*^ *J*_2,F=_173.8 Hz, C‐2), 73.50 (CH_2_^Bn^), 72.75 (d, ^*3*^ *J*_3,F=_25.17 Hz, C‐3), 72.65 (CH_2_^Bn^), 72.56 (C‐4), 71.83 (CH_2_^Bn^), 69.36 (C‐6), 67.97 (C‐5), 55.61 (OCH_3_); ^19^F {^1^H} NMR (659 MHz, CDCl_3_) δ=−194.69 (s); ^19^F NMR (659 MHz, CDCl_3_) δ=−194.69 (dddd, ^2^ *J*_F,2_=45.7 Hz, ^3^ *J*_F,1_=13.3 Hz, ^3^ *J*_F,3_=8.2 Hz, ^4^ *J*_F,4_=3.1 Hz); HRMS (+ESI): *m/z* calc. For C_28_H_31_O_5_Fna^+^ [M+Na]^+^: 489.2048, found: 489.2045.

**3,4,6‐Tri‐*O*‐benzyl‐2‐deoxy‐2‐fluoro‐α‐d–*altrono*‐1,5‐lactone (15)** was synthesized following a protocol by Shi *et al*.^[37]^ Methyl 3,4,6‐tri‐*O*‐benzyl‐2‐deoxy‐2‐fluoro‐α‐d‐*altro*‐pyranoside (**14**, 100 mg, 0.214 mmol, 1 equiv.) was dissolved in glacial acetic acid (1.2 mL) and heated to 70 °C. After the addition of 5 M aqueous HCl (0.2 mL) and strontium chloride hexahydrate (5.7 mg, 0.011 mmol, 0.1 equiv.), stirring was continued at 70 °C for 3 h. The reaction mixture was diluted with water (10 mL), extracted with CH_2_Cl_2_ (3×15 mL) and washed with a sat. aqueous NaHCO_3_‐sol. (3×15 mL). The combined organic layers were dried (MgSO_4_), and the solvent was concentrated *in vacuo (*25 °C). The crude residue was cooled to 0 °C, DMP (145.08 mg, 0.34 mmol, 1.5 equiv.) was added, and stirring was continued at room temperature for 3 h. The reaction mixture was diluted with CH_2_Cl_2_ (10 mL) and washed thrice with a 1 : 1 (v/v) mixture of sat. aqueous NaHCO_3_/sat. aqueous Na_2_S_2_O_3_‐sol. (15 mL). After drying (MgSO_4_), the solvent was evaporated and the crude residue was purified by MPLC (25 g silica gel, 5–50 % EA in *n‐*heptane) to yield 3,4,6‐tri‐*O*‐benzyl‐2‐deoxy‐2‐fluoro‐α‐d‐*altrono*‐1,5‐lactone as a colorless oil (**15**, 50 mg, 52 %); *R*_f_=0.70 (*n‐*heptane/EA 1 : 1, UV & CAM); $[{\alpha ]}_{D}^{20}$ =−54.2 (*c=*1.0 in CHCl_3_); ^1^H NMR (700 MHz, CDCl_3_) δ=7.42‐7.16 (m, 15H, H^Ar^), 5.28 (dd, ^2^ *J*_2,F_=48.2 Hz, ^3^ *J*_2,3_=9.0 Hz, 1H, H‐2), 4.74 (d, ^2^ *J*_H,H_=12.0 Hz, 2H, H^Bn^), 4.64 (d, ^2^ *J*_H,H_=12.0 Hz, 1H, H^Bn^), 4.58 (d, ^2^ *J*_H,H_=11.9 Hz, 1H, H^Bn^), 4.58 (ddd, ^3^ *J*_5,4_ *=*5.9 Hz, ^3^ *J*_5,6a_=4.6 Hz, ^3^ *J*_5,6b_ *=*2.7 Hz 1H, H‐5), 4.51 (d, ^2^ *J*_H,H_=11.9 Hz, 1H, H^Bn^), 4.40 (d, ^2^ *J*_H,H_=12.0 Hz, 1H, H^Bn^), 4.34 (ddd, ^3^ *J*_3,F_=12.0 Hz, ^3^ *J*_3,2_=9.0 Hz, ^3^ *J*_3,4_=3.1 Hz, 1H, H‐3), 3.96 (ddd, ^3^ *J*_4,5_=5.9 Hz, ^3^ *J*_4,3_=3.1 Hz, ^4^ *J*_4,F_=3.1 Hz, 1H, H‐4), 3.58 (dd, ^2^ *J*_*6a,6b*_=10.8 Hz, ^3^ *J*_6a,5_ *=*4.6 Hz, 1H, H‐6a), 3.52 (dd, ^2^ *J*_6b,6a_=10.8 Hz, ^3^ *J*_6b,5_=2.7 Hz, 1H, H‐6b); ^13^C NMR (176 MHz, CDCl_3_) δ=166.61 (d, ^2^ *J*_1,F_=20.0 Hz, C‐1), 137.39 (C^Ar^), 137.16 (C^Ar^), 136.92 (C^Ar^), 128.55 (2 CH^Ar^), 128.54 (2 CH^Ar^), 128.50 (2 CH^Ar^), 128.11 (CH^Ar^), 128.04 (2 CH^Ar^), 127.95 (2 CH^Ar^), 127.92 (2 CH^Ar^), 127.69 (2 CH^Ar^), 87.52 (d, ^1^ *J*_2,F=_189.8 Hz, C‐2), 79.10 (C‐5), 75.13 (d, *J*_3,F=_18.8 Hz, C‐3), 74.63 (d, *J*_4,F=_8.71 Hz, C‐4), 73.76 (CH_2_^Bn^), 73.12 (CH_2_^Bn^), 73.04 (CH_2_^Bn^), 68.97 (C‐6); ^19^F {^1^H} NMR (659 MHz, CDCl_3_) δ=−201.85 (s); ^19^F NMR (659 MHz, CDCl_3_) δ=−201.85 (ddd, ^2^ *J*_F,2_=48.2 Hz, ^3^ *J*_F,3_=12.0 Hz, ^4^ *J*_F,4_=3.1 Hz); HRMS (+ESI): *m/z* calc. for C_27_H_27_O_5_FNa^+^ [M+Na]^+^: 473.1735, found: 473.1732.

**2,6‐Anhydro‐4,5,7‐tri‐*O*‐benzyl‐1,3‐dideoxy‐3‐fluoro‐d–*altro*‐hept‐1‐enitol (16)** was synthesized following a protocol by Waschke *et al*.^[32]^ A 0.5 M solution of Petasis’ reagent (**S2**, 3.9 mL, 2.2 equiv.) in dry toluene was added under argon atmosphere in the dark to 3,4,6‐tri‐*O*‐benzyl‐2‐deoxy‐2‐fluoro‐α‐d‐*altrono*‐1,5‐lactone (**15**, 400 mg, 0.89 mmol, 1 equiv.) dissolved in dry toluene (1 mL). The mixture was stirred at 65 °C for 16 h before being cooled to room temperature. The solution was diluted with *n‐*heptane (20 mL) and stirring was continued for 30 min. The resulting orange suspension was then filtered over Celite^®^, the filtrate evaporated, and the crude residue was purified by MPLC (100 g silica gel, 10–30 % Et_2_O in *n‐*heptane+0.5 % Et_3_N) yielding 2,6‐anhydro‐4,5,7‐tri‐*O*‐benzyl‐1,3‐dideoxy‐3‐fluoro‐d‐*altro*‐hept‐1‐enitol as a light yellow oil (**16**, 255.5 mg, 64 %); *R*_f_=0.62 (*n‐*heptane/Et_2_O 5 : 1, UV & CAM); $[{\alpha ]}_{D}^{20}$ =+45.2 (*c=*0.67 in CHCl_3_); ^1^H NMR (600 MHz, CDCl_3_) δ=7.42–7.16 (m, 15H, H^Ar^), 4.91 (dd, ^2^ *J*_3,F_=48.5 Hz, ^3^ *J*_3,4_=5.1 Hz, 1H, H‐3), 4.86 (d, ^2^ *J*_1a,1b_=5.0 Hz, 1H, H‐1a), 4.72 (d, ^2^ *J*_H,H_=12.3 Hz, 1H, H^Bn^), 4.65 (d, ^2^ *J*_H,H_=12.3 Hz, 1H, H^Bn^), 4.63 (d, ^2^ *J*_H,H_=12.3 Hz, 1H, H^Bn^), 4.61 (d, ^2^ *J*_1b,1a_=5.0 Hz, 1H, H‐1b), 4.57 (d, ^2^ *J*_H,H_=11.6 Hz, 1H, H^Bn^), 4.54 (d, ^2^ *J*_H,H_=12.3 Hz, 1H, H^Bn^), 4.53 (d, ^2^ *J*_H,H_=11.6 Hz, 1H, H^Bn^), 4.21 (ddd, ^3^ *J*_6,5_ *=*8.0 Hz, ^3^ *J*_6,7a_=4.0 Hz, ^3^ *J*_6,7b_ *=*3.0 Hz 1H, H‐6), 4.04 (ddd, ^3^ *J*_5,6_=8.0 Hz, ^3^ *J*_5,4_=3.0 Hz, ^4^ *J*_5,F_=3.0 Hz, 1H, H‐5), 3.96 (ddd, ^3^ *J*_4,F_=8.1 Hz, ^3^ *J*_4,3_=5.1 Hz, ^3^ *J*_4,5_=3.0 Hz, 1H, H‐4), 3.76 (dd, ^2^ *J*_*7a,7b*_=10.9 Hz, ^3^ *J*_7a,6_ *=*4.0 Hz, 1H, H‐7a), 3.71 (dd, ^2^ *J*_7b,7a_=10.9 Hz, ^3^ *J*_7b,6_=3.0 Hz, 1H, H‐7b); ^13^C NMR (151 MHz, CDCl_3_) δ=153.50 (d, ^2^ *J*_2,F_=15.0 Hz, C‐2), 138.11 (C^Ar^), 137.89 (C^Ar^), 137.80 (C^Ar^), 128.41 (2 CH^Ar^), 128.40 (2 CH^Ar^), 128.33 (2 CH^Ar^), 127.91 (2 CH^Ar^), 127.85 (CH^Ar^), 127.83 (CH^Ar^), 127.78 (2 CH^Ar^), 127.73 (2 CH^Ar^), 127.60 (CH^Ar^), 100.65 (d, ^3^ *J*_1,F=_8.9 Hz, C‐1), 87.72 (d, ^1^ *J*_3,F=_176.5 Hz, C‐3), 76.06 (C‐6), 73.52 (CH_2_^Bn^), 73.04 (d, ^*2*^ *J*_4,F=_21.9 Hz, C‐4), 72.94 (CH_2_^Bn^), 72.45 (C‐5), 72.23 (CH_2_^Bn^), 68.98 (C‐7); ^19^F {^1^H} NMR (659 MHz, CDCl_3_) δ=−180.53 (s); ^19^F NMR (659 MHz, CDCl_3_) δ=−180.53 (d, ^2^ *J*_F,3_=48.5 Hz); HRMS (+ESI): *m/z* calc. for C_28_H_29_O_4_FNa^+^ [M+Na]^+^: 471.1942, found: 471.1949.

**4,5,7‐Tri‐*O*‐benzyl‐3‐deoxy‐3‐fluoro‐d–*glycero*‐d–*arabino*‐hept‐2‐ulopyranose (17)** was synthesized following a protocol by Leshch et al.^[34]^ 2,6‐Anhydro‐4,5,7‐tri‐*O*‐benzyl‐1,3‐dideoxy‐3‐fluoro‐d‐*altro*‐hept‐1‐enitol (**16**, 240 mg, 0.54 mmol, 1 equiv.) was dissolved in a 4 : 1 (v/v) mixture of acetone/water (2.6 mL), and the reaction mixture was stirred at rt for 18 h after the addition of potassium osmate dihydrate (9.9 mg, 0.03 mmol, 0.05 equiv.) and NMO (125.4 mg, 1.07 mmol, 2 equiv.). After completion, EA (20 mL) was added, and the mixture was washed with water (2×15 mL). The organic layer was dried (MgSO_4_), filtered, and evaporated *in vacuo*. The crude residue was purified by MPLC (10 g silica gel, 12–100 % EA in *n‐*heptane) yielding 4,5,7‐tri‐O‐benzyl‐3‐deoxy‐3‐fluoro‐d‐*glycero*‐d‐*arabino*‐hept‐2‐ulopyranose as an anomeric mixture in a 1:0.1 ratio of its α‐ and β‐pyranose form (β‐pyranose form only interpretable in ^19^F NMR) as a colorless oil (**17**, 224 mg, 86 %); *R*_f_=0.28 (*n‐*heptane/EA 1 : 1, CAM); $[{\alpha ]}_{D}^{20}$ =+41.2 (c=1.0 in CHCl_3_); α‐Pyranose: ^1^H NMR (600 MHz, CDCl_3_) δ=7.46‐7.18 (m, 15H, H^Ar^), 5.38 (s, 1H, OH‐2), 4.86 (d, ^2^ *J*_H,H_=11.8 Hz, 1H, H^Bn^), 4.66 (dd, ^2^ *J*_3,F_=45.9 Hz, ^3^ *J*_3,4_=3.8 Hz, 1H, H‐3), 4.67 (d, ^2^ *J*_H,H_=10.5 Hz, 1H, H^Bn^), 4.65 (d, ^2^ *J*_H,H_=10.5 Hz, 1H, H^Bn^), 4.54 (d, ^2^ *J*_H,H_=11.8 Hz, 1H, H^Bn^), 4.53 (s, 2H, H^Bn^), 4.23 (dd, ^3^ *J*_5,4_=3.4 Hz, ^3^ *J*_5,6_=9.7 Hz, 1H, H‐5), 4.18 (ddd, ^3^ *J*_4,3_=3.8 Hz, ^3^ *J*_4,5_=3.4 Hz, ^3^ *J*_4,F_=7.0 Hz 1H, H‐4), 3.94 (ddd, ^3^ *J*_6,5_ *=*9.7 Hz, ^3^ *J*_6,7b_ *=*9.9 Hz, ^3^ *J*_6,7a_=3.2 Hz, 1H, H‐6), 3.84 (dd, ^2^ *J*_*7a,7b*_=11.0 Hz, ^3^ *J*_7a,6_ *=*3.2 Hz, 1H, H‐7a), 3.72 (dd, ^2^ *J*_7b,7a_=11.0 Hz, ^3^ *J*_7b,6_=9.9 Hz, 1H, H‐7b), 3.69 (d, ^2^ *J*_1a,1b_=10.8 Hz, 1H, H‐1a), 3.54 (dd, ^2^ *J*_1b,1a_=10.7 Hz, ^3^ *J*_1b,OH_=10.5 Hz, 1H, H‐1b), 1.93 (d, ^3^ *J*_OH,1b_=10.5 Hz, 1H, OH‐1); ^13^C NMR (151 MHz, CDCl_3_) δ=138.27 (C^Ar^), 137.70 (C^Ar^), 136.57 (C^Ar^), 128.72 (2 CH^Ar^), 128.51 (CH^Ar^), 128.49 (2 CH^Ar^), 128.30 (2 CH^Ar^), 128.14 (2 CH^Ar^), 127.96 (CH^Ar^), 127.90 (2 CH^Ar^), 127.83 (2 CH^Ar^), 127.55 (CH^Ar^), 96.10 (d, ^2^ *J*_2,F=_22.14 Hz, C‐2), 84.27 (d, ^1^ *J*_3,F=_183.87 Hz, C‐3), 74.73 (CH_2_^Bn^), 73.55 (CH_2_^Bn^), 73.43 (d, ^*2*^ *J*_4,F=_26.0 Hz, C‐4), 72.51 (CH_2_^Bn^), 72.09 (C‐6), 68.86 (C‐7), 68.29 (C‐5), 64.29 (d, ^3^ *J*_1,F=_4.0 Hz, C‐1); ^19^F {^1^H} NMR (659 MHz, CDCl_3_) δ=−195.53 (s); ^19^F NMR (659 MHz, CDCl_3_) δ=−195.53 (d, ^2^ *J*_F,3_=45.9 Hz); β‐Pyranose: ^19^F {^1^H} NMR (659 MHz, CDCl_3_) δ=−207.00 (s); ^19^F NMR (659 MHz, CDCl_3_) δ=−207.00 (m); HRMS (+ESI): *m/z* calc. for C_28_H_31_O_6_FNa^+^ [M+Na]^+^: 505.1997, found: 505.1993.

### 3‐Deoxy‐3‐fluoro‐d–sedoheptulose (3DFS)

4,5,7‐Tri‐*O*‐benzyl‐3‐deoxy‐3‐fluoro‐d‐*glycero*‐d‐*arabino*‐hept‐2‐ulopyra‐nose (**17**, 175 mg, 0.36 mmol, 1 equiv.) was dissolved in methanol (5 mL) and Pd/C (10 % Pd, 85 mg, 0.057 mmol, 0.14 equiv.) was added. The reaction mixture was degassed (3 freezing/thawing cycles with liquid N_2_ under vacuum) and stirred at room temperature under 1 atm H_2_ pressure for 24 h. The catalyst was removed by filtration over Celite^®^ and the solvent was removed *in vacuo*. The crude residue was purified by reversed‐phase chromatography (C_18_‐modified silica gel, MeCN/H_2_O 1 : 3) yielding 3‐deoxy‐3‐fluoro‐d‐sedoheptulose as a colorless oil as a mixture of its α‐ and β‐furanoses and α‐ and β‐pyranoses in a 2.4 : 3 : 4 : 1 ratio (**3DFS**, 64 mg, 84 %); *R*_f_=0.72 (1‐BuOH/acetone/H_2_O 6 : 3 : 1, CAM); $[{\alpha ]}_{D}^{20}$ =+26.1 (c=1.0 in H_2_O); β‐Furanose: ^1^H NMR (700 MHz, D_2_O) δ=4.97 (dd, ^2^ *J*_3,F_=52.8 Hz, ^3^ *J*_3,4_=6.5 Hz, 1H, H‐3), 4.62 (ddd, ^3^ *J*_4,F_=19.2 Hz, ^3^ *J*_4,3_=6.5 Hz, ^3^ *J*_4,5_=6.5 Hz, 1H, H‐4), 3.87 (dddd, ^3^ *J*_6,5_=6.5 Hz, ^3^ *J*_6,7b_=6.5 Hz, ^3^ *J*_6,7a_=3.1 Hz, ^5^ *J*_6,F_=1.1 Hz, 1H, H‐6), 3.82 (dd, ^3^ *J*_5,6_=6.5 Hz, ^3^ *J*_5,4_=6.5 Hz, 1H, H‐5), 3.78 (dd, ^2^ *J*_7a,7b_=12.0 Hz, ^3^ *J*_7a,6_=3.1 Hz, 1H, H‐7a), 3.66 (dd, ^2^ *J*_1a,1b_=12.2 Hz, ^4^ *J*_1a,F=_1.0 Hz, 1H, H‐1a), 3.64 (dd, ^2^ *J*_7b,7a_=12.0 Hz, ^3^ *J*_7b,6_=6.5 Hz, 1H, H‐7b), 3.62 (d, ^2^ *J*_1b,1a_=12.2 Hz, 1H, H‐1b); ^13^C NMR (176 MHz, D_2_O) δ=103.46 (d, ^2^ *J*_2,F_=17.6 Hz, C‐2), 97.95 (d, ^1^ *J*_3,F_=194.0 Hz, C‐3), 82.62 (d, ^3^ *J*_5,F_=9.6 Hz, C‐5), 76.65 (d, ^2^ *J*_4,F_=22.3 Hz, C‐4), 75.16 (C‐6), 65.48 (C‐1), 64.89 (C‐7); ^19^F {^1^H} NMR (659 MHz, CDCl_3_) δ=−204.94 (s); ^19^F NMR (659 MHz, CDCl_3_) δ=−204.94 (dddd, ^2^ *J*_F,3_=52.8 Hz, ^3^ *J*_F,4_=19.2 Hz, ^5^ *J*_F,6_=1.1 Hz, ^4^ *J*_F,1a_=1.0 Hz); α‐Furanose: ^1^H NMR (700 MHz, D_2_O) δ=4.89 (dd, ^2^ *J*_3,F_=50.8 Hz, ^3^ *J*_3,4_=1.8 Hz, 1H, H‐3), 4.43 (ddd, ^3^ *J*_4,F_=23.8 Hz, ^3^ *J*_4,5_=5.2 Hz, ^3^ *J*_4,3_=1.8 Hz, 1H, H‐4), 4.10 (dd, ^3^ *J*_5,6_=5.2 Hz, ^3^ *J*_5,4_=5.2 Hz, 1H, H‐5), 3.89 (ddd, ^3^ *J*_6,7b_=6.9 Hz, ^3^ *J*_6,5_=5.2, ^3^ *J*_6,7a_=3.7 Hz, 1H, H‐6), 3.77 (dd, ^2^ *J*_1a,1b_=12.2 Hz, ^4^ *J*_1a,F_=2.6 Hz, 1H, H‐1a), 3.75 (dd, ^2^ *J*_7a,7b_=12.0 Hz, ^3^ *J*_7a,6_=3.7 Hz, 1H, H‐7a), 3.68 (dd, ^2^ *J*_1b,1a_=12.0 Hz, ^4^ *J*_1b,F_=3.5 Hz, 1H, H‐1b), 3.63 (dd, ^2^ *J*_7b,7a_=12.2 Hz, ^4^ *J*_7b,6_=6.9 Hz, 1H, H‐7b); ^13^C NMR (176 MHz, D_2_O) δ=107.12 (d, ^2^ *J*_2,F_=26.5 Hz, C‐2), 102.77 (d, ^1^ *J*_3,F_=184.4 Hz, C‐3), 86.07 (d, ^3^ *J*_5,F_=3.4 Hz, C‐5), 77.54 (d, ^2^ *J*_4,F_=27.2 Hz, C‐4), 73.91 (C‐6), 64.95 (C‐7), 64.65 (d, ^3^ *J*_1,F_=3.4 Hz, C‐1); ^19^F {^1^H} NMR (659 MHz, CDCl_3_) δ=−193.07 (s); ^19^F NMR (659 MHz, CDCl_3_) δ=−193.07 (dddd, ^2^ *J*_F,3_=50.8 Hz, ^3^ *J*_F,4_=23.8 Hz, ^4^ *J*_F,1b_=3.5 Hz, ^4^ *J*_F,1a_=2.6 Hz); β‐Pyranose ( ^2^ *C*_5_ ): ^1^H NMR (700 MHz, D_2_O) δ=4.79 (dd, ^2^ *J*_3,F_=49.8 Hz, ^3^ *J*_3,4_=9.3 Hz, 1H, H‐3), 4.31 (ddd, ^3^ *J*_4,F_=11.7 Hz, ^3^ *J*_4,3_=9.3 Hz, ^3^ *J*_4,5_=3.6 Hz, 1H, H‐4), 4.16 (ddd, ^3^ *J*_5,4_=3.6 Hz, ^4^ *J*_5,F_=3.6 Hz, ^3^ *J*_5,6_=2.5 Hz, 1H, H‐5), 4.02 (dddd, ^3^ *J*_6,7a_=7.2 Hz, ^3^ *J*_6,7b_=6.3 Hz, ^3^ *J*_6,5_=2.5, ^5^ *J*_6,F_=1.1, 1H, H‐6), 3.84 (dd, ^2^ *J*_7a,7b_=12.0 Hz, ^3^ *J*_7a,6_=7.2 Hz, 1H, H‐7a), 3.82 (dd, ^2^ *J*_7b,7a_=12.0 Hz, ^4^ *J*_7b,6_=6.3 Hz, 1H, H‐7b), 3.71 (dd, ^2^ *J*_1a,1b_=12.0 Hz, ^4^ *J*_1a,F_=1.7 Hz, 1H, H‐1a), 3.61 (dd, ^2^ *J*_1b,1a_=12.0 Hz, ^4^ *J*_1b,F_=1.1 Hz, 1H, H‐1b); ^13^C NMR (176 MHz, D_2_O) δ=99.86 (d, ^2^ *J*_2,F_=18.5 Hz, C‐2), 90.76 (d, ^1^ *J*_3,F_=182.2 Hz, C‐3), 81.76 (C‐6), 71.86 (d, ^3^ *J*_5,F_=7.5 Hz, C‐5), 68.46 (d, ^2^ *J*_4,F_=19.0 Hz, C‐4), 66.12 (C‐1), 64.82 (C‐7); ^19^F {^1^H} NMR (659 MHz, CDCl_3_) δ=−209.96 (s); ^19^F NMR (659 MHz, CDCl_3_) δ=−209.96 (m); α‐Pyranose ( ^5^ *C*_2_ ): ^1^H NMR (700 MHz, D_2_O) δ=4.74 (dd, ^2^ *J*_3,F_=44.9 Hz, ^3^ *J*_3,4_=3.8 Hz, 1H, H‐3), 4.28 (ddd, ^3^ *J*_4,F_=6.4 Hz, ^3^ *J*_4,3_=3.8 Hz, ^3^ *J*_4,5_=2.5 Hz, 1H, H‐4), 4.08 (ddd, ^3^ *J*_6,5_=10.4 Hz, ^3^ *J*_6,7b_=5.4 Hz, ^3^ *J*_6,7a_=2.4, 1H, H‐6), 3.88 (dd, ^2^ *J*_7a,7b_=12.3 Hz, ^3^ *J*_7a,6_=2.4 Hz, 1H, H‐7a), 3.84 (ddd, ^3^ *J*_5,6_=10.4 Hz, ^4^ *J*_5,F_=3.5 Hz, ^3^ *J*_5,4_=3.5 Hz, 1H, H‐5), 3.80 (dd, ^2^ *J*_7b,7a_=12.3 Hz, ^4^ *J*_7b,6_=5.4 Hz, 1H, H‐7b), 3.72 (dd, ^2^ *J*_1a,1b_=11.9 Hz, ^4^ *J*_1a,F_=2.5 Hz, 1H, H‐1a), 3.53 (dd, ^2^ *J*_1b,1a_=11.9 Hz, ^4^ *J*_1b,F_=3.9 Hz, 1H, H‐1b); ^13^C NMR (176 MHz, D_2_O) δ=98.65 (d, ^2^ *J*_2,F_=24.2 Hz, C‐2), 89.60 (d, ^1^ *J*_3,F_=176.2 Hz, C‐3), 71.28 (C‐6), 70.37 (d, ^2^ *J*_4,F_=27.8 Hz, C‐4), 66.36 (^3^ *J*_1,F_=5.0 Hz, C‐1), 66.22 (d, ^3^ *J*_5,F_=1.3 Hz, C‐5), 63.62 (C‐7); ^19^F {^1^H} NMR (659 MHz, CDCl_3_) δ=−197.98 (s); ^19^F NMR (659 MHz, CDCl_3_) δ=−197.98 (m); HRMS (+ESI): *m/z* calc. for C_7_H_13_O_6_FNa^+^ [M+Na]^+^: 235.0588, found: 235.0586.

### Biochemical assays

#### HILIC‐MS analysis

All solvents were of MS‐grade. Water and MeCN were purchased from Honeywell (Seelze, Germany). Concentrated formic acid was obtained from VWR (Radnor, Pennsylvania, USA) and ammonium bicarbonate as well as ammonium hydroxide from Sigma Aldrich (St. Louis, Missouri, USA). Hydrophilic interaction liquid chromatography (HILIC) was performed using an Atlantis Premier BEH Z‐HILIC Column (2.1 mm×150 mm; 1.7 μm) (Waters, Milford, MA, USA) on a Vanquish Horizon HPLC system (Thermo Fisher Scientific, Waltham, MA, USA). Mobile phase A consisted of 15 mM ammonium bicarbonate in water (pH=9.00), and mobile phase B of 15 mM ammonium bicarbonate in a 9 : 1 mixture (v/v) of MeCN & H_2_O (pH=9.00). The separation was achieved using a 15 min gradient as follows: the start conditions were 100 % B with a gradient to 85 % B at 2 min, followed by an isocratic elution at 85 % until 7 min, from 7–10 min a ramp to 40 % B was applied, and then an isocratic elution at 40 % B from 10–11 min followed. The system was re‐equilibrated using 100 % B from 11–15 min. The column temperature was 30 °C and the flow rate 250 μL/min throughout the chromatographic run. Mass spectrometry analysis was performed on Thermo Scientific™ Orbitrap ID‐X™ Tribrid™ mass spectrometer with an electrospray ionization (ESI) in the negative ion mode. ESI source parameters were set as follows: spray voltage 2900 V, sheath gas 40, auxiliary gas 8, ion transfer tube temperature 275 °C. An MS full scan was conducted in a range of 200–600 *m/z* at a resolution of 60 000. Chromatographic data was analyzed with FreeStyle^TM^.

#### Cellular uptake assays

An aqueous solution of sugar (**3DFS** or **4DFS**, 0.1 M) was added to adherent human dermal fibroblasts cultured in DMEM medium containing 10 % FBS to a final concentration of 1 mM for the respective sugar. No fluorinated sugar was added to the control sample. Cells were incubated at 37 °C for 10 min, then the culture medium was removed, and cells were thoroughly washed (3×5 mL water). The cells were shock‐frozen with liquid nitrogen in the plates and stored at −80 °C. Directly before HILIC‐MS analysis, the cells were taken from the freezer and instantly lysed by the addition of an 8 : 2 (v/v) mixture of methanol and water (300 μL), scraped off the plates and transferred to an Eppendorf tube. The suspension was centrifuged (18 000 g, 20 min, 12 °C) to clear from precipitates. The supernatant was then collected and evaporated at 45 °C with a SpeedVac^TM^. The remaining small molecule extract was dissolved in a mixture of MeCN/H_2_O (1 : 1 (v/v), 160 μL) and subjected to HILIC‐MS‐analysis.

#### Kinase assays

Recombinant sedoheptulose kinase (SHPK) was expressed in *E.coli* and purified as previously described.^[5]^ ADP‐Quest^TM^ Assay kit was obtained from DisoverX and used according to the manufacturer‘s instructions. In short: 20 μL SHPK (0.009 μg/μL in assay buffer), 10 μL sugar solution (d‐sedoheptulose, **3DFS** or **4DFS**; 10 mM in water), 20 μL reagent **A**, 40 μL reagent **B** and 10 μL ATP solution (2 mM in 10 mM HEPES pH 7.6) were mixed in a 96 well plate. Fluorescence was measured on a BioTek Synergy 2 plate reader (excitation wavelength: 530 nm, emission wavelength: 590 nm) for 10 min (signal read‐out every 2 min). All experiments were performed in triplicates. RFU mean of negative control (sedoheptulose with no ATP added) was subtracted from all other values for analysis. The shown graph (Figure 2A) represents the change in signal from starting‐ to endpoint compared to the positive control (**sedo**) which was normalized to 100 %. Data analysis was done with GraphPad Prism.

#### Stability assays^[38]^

Human transaldolase (TALDO1) was purchased from ProSpecBio (Rehovot, Israel) and used as received. Transaldolase stability assays contained the following components in water: 1 mM sugar (**sedo** or **4DFS**), 1 mM ATP, 10 mM HEPES pH 7.6, 20 mM KCl, 10 mM MgCl_2_, 1 mM **G3P**, 0.045 μg SHPK & 1 μg TALDO1 in a final volume of 25 μL. The reactions were incubated at 30 °C for 1.5 h, then diluted 1 : 100 with MeCN/water 1 : 1 (v/v) and subjected to HILIC‐MS analysis. The shown graph represents the peak area of the respective sugar phosphate compared to the negative control (no TALDO1 added) which was normalized to 100 % (Figure 2B). All experiments were performed in triplicates. Statistical analysis was done with GraphPad Prism^TM^.

Human transketolase (TKT) was purchased from ProSpecBio (Rehovot, Israel) and used as received. Transketolase stability assays contained the following components in water: 1 mM sugar (**sedo** or **4DFS**), 1 mM ATP, 10 mM HEPES pH 7.6, 20 mM KCl, 10 mM MgCl_2_, 1 mM **G3P**, 0.5 mM ThPP, 0.045 μg SHPK & 1 μg TKT in a final volume of 25 μL. The reactions were incubated at 30 °C for 0.5 h, then diluted 1 : 100 with MeCN/water 1 : 1 (v/v) and subjected to HILIC‐MS analysis. The shown graph represents the peak area of the respective sugar phosphate compared to the negative control (no TKT added) which was normalized to 100 % (Figure 2C). All experiments were performed in triplicates. Statistical analysis was done with GraphPad Prism^TM^.

## Supporting Information

All experimental procedures, NMR spectra and chromatograms can be found in the Supporting Information. The authors have cited additional references within the Supporting Information.^[50]^

## Author Contributions

L.S., T.S. & K.P. conceived the synthetic routes and wrote the original draft of the manuscript. L.S. & T.S. synthesized all compounds. L.S., T.S. & A.H. conducted biochemical experiments and assays. M.P. performed HILIC‐MS analysis. E.R. supervised HILIC‐MS analysis. H.K. performed NMR analysis of final carbohydrates. K.P., M.M. & A.H. conceived the project. K.P. & A.H. supervised the project. K.P. acquired funding for the project. L.S., T.S., M.P., H.K., T.B., E.P., M.M., A.H. & K.P. reviewed and edited the manuscript.

## Conflict of interest

The authors declare no conflict of interest.

## Supporting information

As a service to our authors and readers, this journal provides supporting information supplied by the authors. Such materials are peer reviewed and may be re‐organized for online delivery, but are not copy‐edited or typeset. Technical support issues arising from supporting information (other than missing files) should be addressed to the authors.

Supporting Information
