# Supplementary material for: Synthesis of 4‐Deoxy‐4‐Fluoro‐d‐Sedoheptulose: A Promising New Sugar to Apply the Principle of Metabolic Trapping
Source: Chemistry. 2023 Sep 28;29(62):e202302277. doi: 10.1002/chem.202302277 (PMC10946558; doi:10.1002/chem.202302277)
Supplement: Supplementary file 1 — Supporting Information [file CHEM-29-0-s001.pdf]

Chemistry–A European Journal

Supporting Information

Synthesis of 4-Deoxy-4-Fluoro-D-Sedoheptulose: A Promising New Sugar to Apply the Principle of Metabolic Trapping

Lukas Scheibelberger, Toda Stankovic, Marlene Pühringer, Hanspeter Kählig, Theresa Balber, Eva-Maria Patronas, Evelyn Rampler, Markus Mitterhauser, Arvand Haschemi, and Katharina Pallitsch*

Table of Contents

1 General Experimental Details 1
1.1 Synthetic Chemistry 2
2 Biochemical assays 33
2.1 HILIC-MS 33
2.2 Uptake assay 33
2.3 Kinase assay 33
2.4 Stability assays 34
3 Appendix 35
3.1 Chromatograms 35
3.2 NMR spectra 44
4 References 106

1 General Experimental Details

All used chemicals and solvents were purchased from commercial sources and used without further purification. ^{1}H, ^{13}C and ^{19}F NMR spectra were recorded on either a Bruker AV III HD 700 (^{1}H: 700.40 MHz, ^{13}C: 176.12 MHz, ^{19}F: 659.03 MHz), AV III 600 (^{1}H: 600.25 MHz, ^{13}C: 150.93 MHz, ^{19}F: 564.803 MHz) or AV NEO 500 (^{1}H: 500.32 MHz, ^{13}C: 125.81 MHz) spectrometer. Chemical shifts (δ) are given in parts per million (ppm) and were referenced to (residual) solvent signals as follows: ^{1}H NMR spectra: $CDCl_3$: δ_H ($CHCl_3$) 7.26, *d*$_6$-DMSO: δ_H [(CD_2H)SO(CD_3)] 2.50, *d*$_4$-MeOH: δ_H (CHD_2OD) 3.31, and D_2O: δ_H (HDO) 4.79; ^{13}C NMR spectra: $CDCl_3$ (δ_C 77.16), *d*$_6$-DMSO (δ_C 39.52), and *d*$_4$-MeOH (δ_C 49.00). ^{13}C NMR spectra in D_2O were referenced indirectly to the ^{1}H NMR frequency of the sample using the "xiref"-command in Bruker Topspin. External CCl_3F (δ_F 0.00) served as reference for ^{19}F NMR spectra. Coupling constants (*J*) are reported in Hz. ^{13}C spectra were recorded *j*-modulated. The chemical shift of the two parts of AB-systems are given separately as unweighted mean value of the single signals. "A" is used to denote the down-field part and "B" to denote the high-field part of the AB-system.

High resolution mass spectrometry (HRMS) was conducted on a Bruker maXis UHR-TOF instrument with electrospray ionization (ESI) in the positive ion mode. Optical rotations were measured on a Schmidt-Haensch Digital Polarimeter Unipol L 2000 and are given in 10^{-1} deg cm^2 g^{-1}.
Chromatographic separations (MPLC) were carried out on a Biotage Isolera Prime (Biotage, Uppsala, Sweden) flash purification system using Macherey-Nagel silica gel 60 (0.04–0.063 mm) in self-packed cartridges. Thin layer chromatography (TLC) was carried out on precoated Merck silica gel 60 F_{254} glass plates or precoated Macherey-Nagel ALUGRAM Xtra SIL G UV254 aluminum plates. Compounds were visualized with UV light (254 nm) and/or by dipping the plate in one of the following solutions, followed by heating with a heat gun: Cerium ammonium molybdate solution (CAM, 46 g $(NH_4)_6Mo_7O_{24}$×4 H_2O, 2 g $Ce(SO_4)_2$×4H_2O) in 1 L 10%(w/w) aq. H_2SO_4); $KMnO_4$

solution (9 g $KMnO_4$, 60 g K_2CO_3 in 900 mL H_2O and 15 mL 5%(w/w) aq. NaOH) or vanillin stain (60 g vanillin in 1000 mL EtOH & 10 mL conc. H_2SO_4) followed by heating.

1.1 Synthetic Chemistry

1.1.1 Synthesis of 4-deoxy-4-fluoro-D-sedoheptulose (4DFS)

1.1.1.1 1-(*para*-Toluenesulfonyl)imidazole[1] (S1)

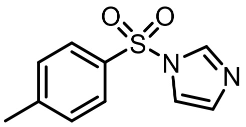

S1
$C_{10}H_{10}N_2O_2S$
222.26 g/mol

para-Toluenesulfonylchloride (3 g, 15.74 mmol, 1 eq) was dissolved in dry dichloromethane (DCM, 11 mL) and added dropwise to a solution of imidazole (2.46 g, 36.19 mmol, 2.3 eq) in dry DCM (11 mL) at 0°C under argon atmosphere. The reaction mixture was stirred at room temperature for 2.5 h after the addition was complete. The suspension was filtered through a pad of silica and eluted with a 1:1 mixture of ethyl acetate (EA) and cyclohexane (38 mL). The filtrate was concentrated and treated with EA (1.8 mL) and cyclohexane (18 mL). Precipitation was induced by sonication, followed by storage of the mixture at 4°C over 2 days. Filtration yielded 1-(*para*-toluenesulfonyl)imidazole (**S1**) as a white solid (3 g, 86%); R_f = 0.4 (*n*-heptane/EA 1:1, UV); ^{1}H NMR (600 MHz, $CDCl_3$): δ = 8.00 (s, 1H, H^{imid}), 7.82 (d, $^3J_{H,H}$ = 8.4 Hz, 2H, H^{Ar}), 7.35 (d, $^3J_{H,H}$ = 8.1 Hz, 2H, H^{Ar}), 7.28 (t, J = 1.4 Hz, 1H, H^{imid}), 7.07 (bs, 1H, H^{imid}), 2.43 (s, 1H, CH_3); ^{13}C NMR (151 MHz, $CDCl_3$): δ = 146.45 (C^{Ar}), 136.78 (CH^{imid}), 135.10 (C^{Ar}), 131.56 (CH^{imid}), 130.56 (2 CH^{Ar}), 127.50 (2 CH^{Ar}), 117.55 (CH^{imid}), 21.85 (CH_3); HRMS (ESI+): *m/z* calc. for $C_{10}H_{11}N_2O_2S^+$ $[M+H]^+$: 223.0536, found: 223.0541.

1.1.1.2 Methyl 2,3-anhydro-4,6-*O*-benzylidene-α-D-*manno*-pyranoside[2,3] (2)

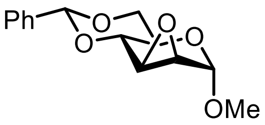

2
$C_{14}H_{16}O_5$
264.28 g/mol

Methyl 4,6-*O*-benzylidene-α-D-*gluco*-pyranoside (**1**, 97% purity, 5.00 g, 17.18 mmol. 1 equiv.) was dissolved in dry DMF (100 mL) under an argon atmosphere. A suspension of sodium hydride (90% purity, 1.05 g, 39.52 mmol, 2.3 equiv.) in dry DMF (200 mL) was added dropwise and the reaction mixture was stirred for 45 min at room temperature. 1-(*para*-toluenesulfonyl)imidazole (**S1**, 4.01 g, 18.04 mmol, 1.05 equiv.), dissolved in dry DMF (140 mL), was added dropwise over 2 h via a syringe pump. The reaction mixture was stirred for 1 h at room temperature after the addition was complete and then poured into ice-cold H_2O (850 mL) while stirring. The white precipitate was collected by filtration, washed with cold H_2O and dried under high vacuum. The mother liquor was combined with the filtrate of the wash and extracted thrice with EA. The combined organic layers were washed twice with water and once with brine, dried ($MgSO_4$), filtered and evaporated to give a white solid. The combined crude solids were purified by recrystallization from methanol to yield methyl 2,3-anhydro-4,6-*O*-benzylidene-α-D-*manno*-pyranoside (3.284 g) as colourless needles. The mother liquor of the recrystallization was evaporated and the remaining solid was recrystallized again from methanol yielding another portion of **2** (combined yield: 4.01 g, 88%); R_f = 0.29 (*n*-heptane/EA 9:1, UV & CAM); $[\alpha]_D^{20}$ = +100.2 (c = 1.0 in $CHCl_3$) (lit.,[4] 100 (*c* = 1.0 in $CHCl_3$)); ^{1}H NMR (700

MHz, $CDCl_3$) δ = 7.51-7.49 (m, 2H, H^{Ar}), 7.41-7.37 (m, 3H, H^{Ar}), 5.57 (s, 1H, H^{Bn}), 4.90 (s, 1H, H-1), 4.29-4.24 (m, 1H, H-6a), 3.75-3.67 (m, 3H, H-4, H-5 & H-6b), 3.48 (d, $^{3}J_{3,2}$ = 3.7 Hz, 1H, H-3), 3.47 (s, 3H, OCH_3), 3.17 (d, $^{3}J_{2,3}$ = 3.6 Hz, 1H, H-2); ^{13}C NMR (176 MHz, $CDCl_3$) δ = 137.21 (C^{Ar}), 129.45 (CH^{Ar}), 128.54 (2 CH^{Ar}), 126.34 (2 CH^{Ar}), 102.61 (CH^{Bn}), 97.05 (C-1), 75.05 (C-4), 69.59 (C-6), 61.83 (C-5), 55.94 (OCH_3), 54.00 (C-3), 50.72 (C-2); HRMS (ESI+): *m/z* calc. for $C_{14}H_{16}O_5Na^+$ $[M+Na]^+$: 287.0890, found: 287.0889.

1.1.1.3 Methyl 4,6-*O*-benzylidene-3-deoxy-3-fluoro-α-D-*altro*-pyranoside[5] (3)

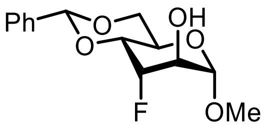

3
$C_{14}H_{17}FO_5$
284.28 g/mol

N,N,N,N-Tetrabutylammonium fluoride trihydrate (23.313 g, 73.83 mmol, 8 equiv.) and KHF_2 (2.886 g, 36.95 mmol, 4 equiv.) were added to 2,3-anhydro-4,6-*O*-benzylidene-α-D-*manno*-pyranoside (**2**, 2.441 g, 9.24 mmol, 1 equiv.) in a PFA-flask. The flask was flushed with argon, sealed with a septum and an argon balloon was inserted. The reaction mixture was stirred for 3 h at 130°C (reagents began to melt at 70°C). The resulting brown liquid was cooled to room temperature, diluted with EA (80 mL) and water (20 mL) and neutralized by addition of sat. aq. $NaHCO_3$-solution (140 mL). The layers were separated and the aqueous layer was extracted thrice with EA. The combined organic layers were washed with water and brine, dried ($MgSO_4$), filtered and evaporated. The residue was purified by MPLC (50 g silica gel, 5-40% EA in *n*-heptane) yielding methyl 4,6-*O*-benzylidene-3-deoxy-3-fluoro-α-D-*altro*-pyranoside (**3**) as a white solid (1.99 g, 76%); R_f = 0.1 (*n*-heptane/EA 4:1, UV & CAM); $[\alpha]_D^{20}$ = +110.7 (c = 1.0 in $CHCl_3$) (lit.,[5] 108.3 (*c* = 1.0 in $CHCl_3$)); ^{1}H NMR (600 MHz, $CDCl_3$) δ = 7.52-7.49 (m, 2H, H^{Ar}), 7.39-7.34 (m, 3H, H^{Ar}), 5.61 (s, 1H, H^{Bn}), 4.86 (dt, $^{2}J_{3,F}$ = 49.8 Hz, $^{3}J_{3,2}$ = 3.2 Hz, $^{3}J_{3,4}$ = 2.6 Hz, 1H, H-3), 4.67 (s, 1H, H-1), 4.36 (dd, $^{2}J_{6a,6b}$ = 10.1 Hz, $^{3}J_{6a,5}$ = 5.3 Hz, 1H, H-6a), 4.31 (td, $^{3}J_{5,4}$ = $^{3}J_{5,6b}$ = 10.1 Hz, $^{3}J_{5,6a}$ = 5.3 Hz, 1H, H-5), 4.15 (td, $^{3}J_{2,OH}$ = $^{3}J_{2,F}$ = 6.2 Hz, $^{3}J_{2,3}$ = 3.2 Hz, 1H, H-2), 3.99 (ddd, $^{3}J_{4,F}$ = 30.2 Hz, $^{3}J_{4,5}$ = 9.7, $^{3}J_{4,3}$ = 2.6 Hz, 1H, H-4), 3.80 (td, $^{2}J_{6b,6a}$ = $^{3}J_{6b,5}$ = 10.1 Hz, $^{5}J_{6b,F}$ =1.0 Hz, 1H, H-6b), 3.44 (s, 3H, OCH_3), 1.96 (d, $^{3}J_{OH,2}$ = 6.2 Hz, 1H, OH); ^{13}C NMR (151 MHz, $CDCl_3$) δ = 137.25 (C^{Ar}), 129.41 (CH^{Ar}), 128.50 (2 CH^{Ar}), 126.45 (2 CH^{Ar}), 102.76 (CH^{Bn}), 101.58 (C-1), 87.10 (d, $^{1}J_{3,F}$ = 185.6 Hz, C-3), 74.99 (d, $^{2}J_{4,F}$ = 16.6 Hz, C-4), 69.36 (C-6), 69.29 (d, $^{2}J_{2,F}$ = 26.5 Hz, C-2), 58.53 (d, $^{3}J_{5,F}$ = 3.1 Hz, C-5), 55.82 (OCH_3); $^{19}F\{^{1}H\}$ NMR (659 MHz, $CDCl_3$) δ = −205.71 (s); ^{19}F NMR (659 MHz, $CDCl_3$) δ = −205.71 (ddd, $^{2}J_{F,3}$ = 49.8 Hz, $^{3}J_{F,4}$ = 30.2 Hz, $^{3}J_{F,2}$ = 6.2 Hz); HRMS (ESI+): *m/z* calc. for $C_{14}H_{17}FO_5Na^+$ $[M+Na]^+$: 307.0952, found: 307.0964.

1.1.1.4 Methyl 4-*O*-benzyl-3-deoxy-3-fluoro-α-D-*altro*-pyranoside (4)

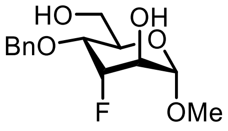

4
$C_{14}H_{19}FO_5$
286.30 g/mol

Compound **4** was synthesized following a protocol by Shie *et al.*[6] Borane THF complex (1 M in THF, 52.0 mL, 51.99 mmol, 5 equiv.) was slowly added to methyl 4,6-*O*-benzylidene-3-deoxy-3-fluoro-α-D-*altro*-pyranoside (**3**, 2.96 g, 10.40 mmol, 1 equiv.) under an argon atmosphere and the reaction mixture was stirred for 10 min at room temperature. $Cu(OTf)_2$ (376 mg, 1.04 mmol, 0.1 equiv.) was added

and stirring was continued for 1.5 h. The black suspension was then cooled to 0°C and triethylamine (1.052 g, 1.45 mL, 1.04 mmol, 1 equiv.) was added dropwise followed by methanol (36 mL). The mixture was warmed to room temperature and co-evaporated thrice with methanol. The crude residue was purified by MPLC (100 g silica gel, 20-100% EA in *n*-heptane) to obtain methyl 4-*O*-benzyl-3-deoxy-3-fluoro-α-D-*altro*-pyranoside (**4**) as a colourless oil (2.95 g, 99%); R_f = 0.34 (*n*-heptane/EA 1:3, UV & vanillin); $[\alpha]_D^{20}$ = +106.1 (c = 1.0 in $CHCl_3$); ^1H NMR (600 MHz, $CDCl_3$) δ = 7.38-7.33 (m, 4H, H^{Ar}), 7.33-7.29 (m, 1H, H^{Ar}), 4.86 (dt, $^2J_{3,F}$ = 49.1 Hz, $^3J_{3,2}$ = $^3J_{3,4}$ = 2.9 Hz, 1H, H-3), 4.73 (d, $^2J_{H,H}$ = 11.7 Hz, 1H, H^{Bn}), 4.65 (s, 1H, H-1), 4.58 (d, $^2J_{H,H}$ = 11.7 Hz, 1H, H^{Bn}), 4.09-4.04 (m, 2H, H-2 & H-5), 3.84 (ddd, $^3J_{4,F}$ = 27.2 Hz, $^3J_{4,5}$ = 9.8 Hz, $^3J_{4,3}$ = 2.9 Hz, 1H, H-4), 3.85-3.82 (m, 2H, H-6a & H-6b), 3.40 (s, 3H, OCH_3), 2.36 (d, $^3J_{OH,2}$ = 7.2 Hz, 1H, OH-2), 1.97 (dd, $^3J_{OH,6}$ = 6.6 Hz, $^3J_{OH,6}$ = 6.0 Hz, 1H, OH-6); ^{13}C NMR (151 MHz, $CDCl_3$) δ = 137.71 (C^{Ar}), 128.69 (2 CH^{Ar}), 128.21 (CH^{Ar}), 128.14 (2 CH^{Ar}), 101.30 (C-1), 87.03 (d, $^1J_{3,F}$ = 183.8 Hz, C-3), 71.54 (CH_2^{Bn}) 70.25 (d, $^2J_{4,F}$ = 16.8 Hz, C-4), 68.98 (d, $^2J_{2,F}$ = 25.3 Hz, C-2), 67.26 (d, $^3J_{5,F}$ = 2.9 Hz, C-5), 62.19 (C-6), 55.74 (OCH_3); $^{19}F\{^1H\}$ NMR (659 MHz, $CDCl_3$) δ = −207.40 (s); ^{19}F NMR (659 MHz, $CDCl_3$) δ = −207.39 (ddd, $^2J_{F,3}$ = 49.1 Hz, $^3J_{F,4}$ = 27.2 Hz, $^3J_{F,2}$ = 6.5 Hz); HRMS (ESI+): *m/z* calc. for $C_{14}H_{19}FO_5Na^+$ $[M+Na]^+$: 309.1109, found: 309.1115.

1.1.1.5 Methyl 2,4,6-tri-*O*-benzyl-3-deoxy-3-fluoro-α-D-*altro*-pyranoside (5)

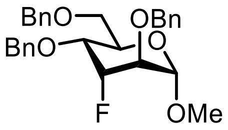

5

$C_{28}H_{31}FO_5$

466.55 g/mol

Methyl 4-*O*-benzyl-3-deoxy-3-fluoro-α-D-*altro*-pyranoside (**4**, 2.93 g, 10.22 mmol, 1 equiv.) was dissolved in dry DMF (29.2 mL) under an argon atmosphere and cooled to 0°C. Sodium hydride (90% purity, 817 mg, 30.65 mmol, 3 equiv.) was added in small portions and the reaction mixture was stirred for 30 min at 0°C. Benzyl bromide (5.24 g, 3.6 mL, 30.65 mmol, 3 equiv.) was then added dropwise and the reaction mixture was stirred at room temperature for 16 h. Subsequently, methanol (45 mL) was added at 0°C, followed by water (45 mL) and EA (45 mL). The biphasic mixture was allowed to come to room temperature, the layers were separated and the aq. layer was extracted twice with EA. The combined org. layers were washed twice with water and once with brine. The org. layer was dried ($MgSO_4$), filtered and evaporated. The crude residue was purified by MPLC (100 g silica gel, 5-31% EA in *n*-heptane) yielding methyl 2,4,6-tri-*O*-benzyl-3-deoxy-3-fluoro-α-D-*altro*-pyranoside (**5**) as a colourless oil (3.824 g, 80%); R_f = 0.26 (*n*-heptane/EA 4:1, UV & CAM); $[\alpha]_D^{20}$ = + 47.9 (c = 1.0 in $CHCl_3$); ^1H NMR (700 MHz, $CDCl_3$) δ = 7.36-7.26 (m, 15H, 15 H^{Ar}), 4.79 (dt, $^2J_{3,F}$ = 49.1 Hz, $^3J_{3,2}$ = 3.1 Hz, $^3J_{3,4}$ = 2.7 Hz, 1H, H-3), 4.74 (s, 1H, H-1), 4.67 (d, $^2J_{H,H}$ = 12.1 Hz, 1H, H^{Bn}), 4.65 (d, $^2J_{H,H}$ = 12.1 Hz, 1H, H^{Bn}), 4.61 (d, $^2J_{H,H}$ = 11.6 Hz, 1H, H^{Bn}), 4.55 (d, $^2J_{H,H}$ = 11.4 Hz, 1H, H^{Bn}), 4.53 (d, $^2J_{H,H}$ = 11.4 Hz, 1H, H^{Bn}), 4.51 (d, $^2J_{H,H}$ = 11.6 Hz, 1H, H^{Bn}), 4.15 (ddd, $^3J_{5,4}$ = 9.4 Hz, $^3J_{5,6a}$ = 4.0 Hz, $^3J_{5,6b}$ = 2.6 Hz, 1H, H-5), 3.90 (ddd, $^3J_{4,F}$ = 27.1 Hz, $^3J_{4,5}$ = 9.4 Hz, $^3J_{4,3}$ = 2.7 Hz, 1H, H-4), 3.78 (dd, $^3J_{2,F}$ = 7.4 Hz, $^3J_{2,3}$ = 3.1 Hz, 1H, H-2), 3.76 (dd, $^2J_{6a,6b}$ = 10.9 Hz, $^3J_{6a,5}$ = 4.0 Hz, 1H, H-6a), 3.73 (dd, $^2J_{6b,6a}$ = 10.9 Hz, $^3J_{6b,5}$ = 2.6 Hz, 1H, H-6b), 3.39 (s, 3H, OCH_3); ^{13}C NMR (176 MHz, $CDCl_3$) δ = 138.42 (C^{Ar}), 137.99 (C^{Ar}), 137.57 (C^{Ar}), 128.66 (2 CH^{Ar}), 128.52 (2 CH^{Ar}), 128.46 (2 CH^{Ar}), 128.18 (CH^{Ar}), 128.09 (2

CH^{Ar}), 127.95 (2 CH^{Ar}), 127.94 (CH^{Ar}), 127.88 (2 CH^{Ar}), 127.68 (CH^{Ar}), 99.51 (C-1), 86.56 (d, $^{1}J_{3,F}$ = 183.1 Hz, C-3), 75.74 (d, $^{2}J_{2,F}$ = 24.5 Hz, C-2), 73.65 (CH$_2^{Bn}$), 72.94 (CH$_2^{Bn}$), 71.82 (CH$_2^{Bn}$), 71.41 (d, $^{2}J_{4,F}$ = 16.6 Hz, C-4), 69.34 (C-6), 67.01 (d, $^{3}J_{5,F}$ = 3.8 Hz, C-5), 55.51 (OCH$_3$); ^{19}F{^{1}H} NMR (659 MHz, CDCl$_3$) δ = −207.65 (s); ^{19}F NMR (659 MHz, CDCl$_3$) δ = −207.65 (ddd, $^{2}J_{F,3}$ = 49.1 Hz, $^{3}J_{F,4}$ = 27.1 Hz, $^{3}J_{F,2}$ = 7.8 Hz); HRMS (ESI+): *m/z* calc. for $C_{28}H_{31}FO_5Na^+$ [M+Na]$^+$: 489.2048, found: 489.2051.

1.1.1.6 2,4,6-Tri-*O*-benzyl-3-deoxy-3-fluoro-D-altrose (6)

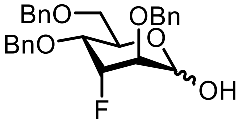

6

$C_{27}H_{29}FO_5$

452.52 g/mol

Compound **6** was synthesized following a protocol by Matwiejuk *et al.*[7] Methyl 2,4,6-tri-*O*-benzyl-3-deoxy-3-fluoro-α-D-*altro*-pyranoside (**5**, 3.804 g, 8.15 mmol, 1 equiv.) was dissolved in acetic acid (81.5 mL) and 1 M aq. H_2SO_4 (18.5 mL, 81.53 mmol, 10 equiv.) was added dropwise under stirring. The flask was equipped with a reflux condenser, heated to 100°C and stirred for 15 h. The reaction mixture was then cooled to room temperature and ice-cold water (300 mL) was slowly added. The mixture was extracted thrice with DCM and the combined org. layers were washed twice with a sat. aq. NaHCO$_3$-sol. and once with brine. The org. layer was dried (MgSO$_4$), filtered and evaporated. The crude residue was purified by MPLC (100 g silica gel, 0-4% EA in DCM) yielding 2,4,6-tri-*O*-benzyl-3-deoxy-3-fluoro-D-altrose (**6**) as a colourless oil and as a mixture of anomers (α:β = 1:4) (1.77 g, 48%); R_f = 0.14 (*n*-heptane/EA 4:1, UV & CAM); $[\alpha]_D^{20}$ = +21.5 (c = 1.0 in CHCl$_3$); β-Pyranose: ^{1}H NMR (600 MHz, CDCl$_3$) δ = 7.39-7.22 (m, 15H, H^{Ar}), 5.01 (dt, $^{3}J_{1,OH}$ = 11.7 Hz, $^{3}J_{1,2}$ = $^{4}J_{1,F}$ = 2.0 Hz, 1H, H-1), 4.74 (ddd, $^{2}J_{3,F}$ = 48.7 Hz, $^{3}J_{3,2}$ = 4.0 Hz, $^{3}J_{3,4}$ = 2.2 Hz, 1H, H-3), 4.68 (d, $^{2}J_{H,H}$ = 12.1 Hz, 1H, H^{Bn}), 4.64 (d, $^{2}J_{H,H}$ = 12.1 Hz, 1H, H^{Bn}), 4.56 (d, $^{2}J_{H,H}$ = 12.0 Hz, 1H, H^{Bn}), 4.54 (d, $^{2}J_{H,H}$ = 12.0 Hz, 1H, H^{Bn}), 4,47 (s, 2H, CH$_2^{Bn}$), 3.94 (dd, $^{3}J_{5,4}$ = 9.5 Hz, $^{3}J_{5,6}$ = 1.5 Hz 1H, H-5), 3.86 (ddd, $^{3}J_{4,F}$ = 29.0 Hz, $^{3}J_{4,5}$ = 9.5 Hz, $^{3}J_{4,3}$ = 2.2 Hz, 1H, H-4), 3.77-3.70 (m, 3H, H-2, H-6a & H-6b), 3.57 (d, $^{3}J_{1,OH}$ = 11.7 Hz, 1H, OH); ^{13}C NMR (151 MHz, CDCl$_3$) δ = 138.30 (C^{Ar}), 137.71 (C^{Ar}), 137.23 (C^{Ar}), 128.90 (2 CH^{Ar}), 128.62 (CH^{Ar}), 128.60 (2 CH^{Ar}), 128.48 (2 CH^{Ar}), 128.30 (2 CH^{Ar}), 128.24 (2 CH^{Ar}), 128.12 (CH^{Ar}), 128.09 (2 CH^{Ar}), 127.75 (CH^{Ar}), 91.89 (C-1), 86.77 (d, $^{1}J_{3,F}$ = 178.7 Hz, C-3), 76.05 (d, $^{2}J_{2,F}$ = 25.9 Hz, C-2), 74.10 (CH$_2^{Bn}$), 73.78 (CH$_2^{Bn}$), 72.37 (d, $^{3}J_{5,F}$ = 3.6 Hz, C-5), 72.15 (CH$_2^{Bn}$), 71.20 (d, $^{2}J_{4,F}$ = 16.5 Hz, C-4), 69.15 (C-6); ^{19}F{^{1}H} NMR (659 MHz, CDCl$_3$) δ = −209.84 (s); ^{19}F NMR (659 MHz, CDCl$_3$) δ = −209.84 (dd, $^{2}J_{F,3}$ = 48.7 Hz, $^{3}J_{F,4}$ = 29.1 Hz); α-Pyranose: ^{1}H NMR (600 MHz, CDCl$_3$) δ = 7.38-7.22 (m, 15H, H^{Ar}), 5.20 (d, $^{3}J_{1,OH}$ = 7.9 Hz, 1H, H-1), 4.84 (ddd, $^{2}J_{3,F}$ = 49.3 Hz, $^{3}J_{3,2}$ = 4.0 Hz, $^{3}J_{3,4}$ = 2.6 Hz, 1H, H-3), 4.68 (d, $^{2}J_{H,H}$ = 12.1 Hz, 1H, H^{Bn}), 4.64 (d, $^{2}J_{H,H}$ = 12.1 Hz, 1H, H^{Bn}), 4.60 (d, $^{2}J_{H,H}$ = 11.8 Hz, 1H, H^{Bn}), 4.56 (d, $^{2}J_{H,H}$ = 11.8 Hz, 1H, H^{Bn}), 4.54 (d, $^{2}J_{H,H}$ = 12.1 Hz, 1H, H^{Bn}), 4.53 (d, $^{2}J_{H,H}$ = 12.1 Hz, 1H, H^{Bn}), 4.28 (dt, $^{3}J_{5,4}$ = 8.6 Hz, $^{3}J_{5,6a}$ = $^{3}J_{5,6b}$ = 4.2 Hz, 1H, H-5), 3.94 (ddd, $^{3}J_{4,F}$ = 27.1 Hz, $^{3}J_{4,5}$ = 8.6 Hz, $^{3}J_{4,3}$ = 2.6 Hz, 1H, H-4), 3.82 (ddd, $^{3}J_{2,F}$ = 8.0 Hz, $^{3}J_{2,3}$ = 4.0 Hz, $^{3}J_{2,1}$ = 0.9 Hz, 1H, H-2), 3.77-3.69 (m, 2H, H-6a & H-6b), 3.27 (dd, $^{3}J_{OH,1}$ = 7.9 Hz, $J_{OH,F}$ = 5.2 Hz, 1H, OH); ^{13}C NMR (151 MHz, CDCl$_3$) δ = 138.26 (C^{Ar}), 137.68 (C^{Ar}), 137.43 (C^{Ar}), 128.70 (2 CH^{Ar}), 128.62 (CH^{Ar}), 128.60 (2 CH^{Ar}), 128.50 (2 CH^{Ar}), 128.24 (CH^{Ar}), 128.17 (2 CH^{Ar}), 128.01 (2 CH^{Ar}), 127.95 (2 CH^{Ar}), 127.77 (CH^{Ar}), 93.29 (C-1), 88.40

(d, $^{1}J_{3,F}$ = 179.2 Hz, C-3), 75.94 (d, $^{2}J_{2,F}$ = 22.5 Hz, C-2), 73.73 (CH_2^{Bn}), 72.80 (CH_2^{Bn}), 72.20 (CH_2^{Bn}), 71.60 (d, $^{2}J_{4,F}$ = 16.4 Hz, C-4), 69.35 (C-6), 67.80 (d, $^{3}J_{5,F}$ = 4.4 Hz, C-5); $^{19}F\{^{1}H\}$ NMR (659 MHz, $CDCl_3$) δ = −205.48 (s); ^{19}F NMR (659 MHz, $CDCl_3$) δ = −205.48 (dddd, $^{2}J_{F,3}$ = 49.3 Hz, $^{3}J_{F,4}$ = 27.3 Hz, $^{3}J_{F,2}$ = 7.0 Hz, $J_{F,OH}$ = 5.2 Hz); HRMS (ESI+): *m/z* calc. for $C_{27}H_{29}FO_5Na^+$ $[M+Na]^+$: 475.1891, found: 475.1881.

1.1.1.7 2,4,6-Tri-*O*-benzyl-3-deoxy-3-fluoro-D-*altrono*-1,5-lactone (7)

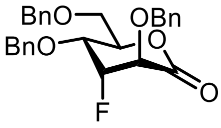

7
$C_{27}H_{27}FO_5$
450.51 g/mol

2,4,6-Tri-*O*-benzyl-3-deoxy-3-fluoro-D-altrose (**6**, 1.745 g, 3.86 mmol, 1 equiv.) was dissolved in dry DCM (44.8 mL) under argon. Dess-Martin-Periodinane (DMP, 3.271 g, 7.99 mmol, 2 equiv.) was added and the mixture was stirred for 1 h at room temperature. Then, the reaction was quenched by the addition of a sat. aq. $NaHCO_3$-sol. (160 mL) and a sat. aq. $Na_2S_2O_3$-sol. (160 mL) was added. The mixture was extracted thrice with DCM, the combined org. layers were dried ($MgSO_4$), filtered and evaporated. The crude residue was purified by MPLC (100 g silica gel, 4-40% EA in *n*-heptane) yielding 2,4,6-tri-*O*-benzyl-3-deoxy-3-fluoro-D-*altrono*-1,5-lactone (**7**) as a colourless oil (1.663 g, 96%); R_f = 0.34 (*n*-heptane/EA 4:1, UV & CAM); $[\alpha]_D^{20}$ = –25.2 (c = 1.0 in $CHCl_3$); ^{1}H NMR (600 MHz, $CDCl_3$) δ = 7.41-7.26 (m, 15H, H^{Ar}), 5.14 (ddd, $^{2}J_{3,F}$ = 49.8 Hz, $^{3}J_{3,2}$ = 7.5 Hz, $^{3}J_{3,4}$ = 3.1 Hz, 1H, H-3), 5.04 (d, $^{2}J_{H,H}$ = 11.5 Hz, 1H, H^{Bn}), 4.74 (d, $^{2}J_{H,H}$ = 11.5 Hz, 1H, H^{Bn}), 4.74 (d, $^{2}J_{H,H}$ = 11.7 Hz, 1H, H^{Bn}), 4.62 (d, $^{2}J_{H,H}$ = 11.7 Hz, 1H, H^{Bn}), 4.58 (d, $^{2}J_{H,H}$ = 11.9 Hz, 1H, H^{Bn}), 4.58 (m, 1H, H-5), 4.48 (d, $^{2}J_{H,H}$ = 11.9 Hz, 1H, H^{Bn}), 4.44 (dd, $^{3}J_{2,F}$ = 14.5 Hz, $^{3}J_{2,3}$ = 7.5 Hz, 1H, H-2), 4.17 (ddd, $^{3}J_{4,F}$ = 14.0 Hz, $^{3}J_{4,5}$ = 4.2 Hz, $^{3}J_{4,3}$ = 3.1 Hz, 1H, H-4), 3.67 (dd, $^{2}J_{6a,6b}$ = 11.0 Hz, $^{3}J_{6a,5}$ = 3.8 Hz, 1H, H-6a), 3.64 (dd, $^{2}J_{6b,6a}$ = 11.0 Hz, $^{3}J_{6b,5}$ = 2.7 Hz, 1H, H-6b); ^{13}C NMR (151 MHz, $CDCl_3$) δ = 168.51 (d, $^{3}J_{1,F}$ = 10.4 Hz, C-1), 137.23 (C^{Ar}), 137.16 (2 C^{Ar}), 128.71 (2 CH^{Ar}), 128.67 (2 CH^{Ar}), 128.58 (2 CH^{Ar}), 128.39 (2 CH^{Ar}), 128.33 (CH^{Ar}), 128.19 (CH^{Ar}), 128.10 (3 CH^{Ar}), 127.90 (2 CH^{Ar}), 88.84 (d, $^{1}J_{3,F}$ = 186.5 Hz, C-3), 78.17 (d, $^{3}J_{5,F}$ = 6.5 Hz, C-5), 74.83 (d, $^{2}J_{2,F}$ = 24.3 Hz, C-2), 74.05 (CH_2^{Bn}), 73.94 (CH_2^{Bn}), 73.34 (CH_2^{Bn}), 73.30 (d, $^{2}J_{4,F}$ = 17.3 Hz, C-4), 68.81 (C-6); $^{19}F\{^{1}H\}$ NMR (659 MHz, $CDCl_3$) δ = −199.38 (s); ^{19}F NMR (659 MHz, $CDCl_3$) δ = −199.38 (dt, $^{2}J_{F,3}$ = 49.8 Hz, $^{3}J_{F,2}$ = 14.5 Hz, $^{3}J_{F,4}$ = 14.0 Hz); HRMS (ESI+): *m/z* calc. for $C_{27}H_{27}O_5FNa^+$ $[M+Na]^+$: 473.1735, found: 473.1739.

1.1.1.8 Bis(cyclopentadienyl)dimethyltitanium/Petasis' reagent[8,9] (S2)

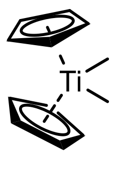

S2
$C_{12}H_{16}Ti$
208.13 g/mol

This reaction is light sensitive and was carried out in the dark. Bis(cyclopentadienyl)titanium dichloride (3.5 g, 14.06 mmol, 1 equiv.) was suspended in dry Et_2O (70.3 mL) in a flame-dried flask under an argon atmosphere and cooled to 10°C. Methyl lithium (1.6 M in Et_2O, 21.1 mL, 33.74 mmol, 2.4 equiv.) was added dropwise and the reaction mixture was stirred for 15 min at room temperature. The reaction was quenched by the dropwise addition of ice-cold H_2O (30 mL) at 0°C. The layers were separated and the aqueous layer was extracted twice with Et_2O. The combined organic layers were dried ($MgSO_4$), filtered, and evaporated (25°C) yielding bis(cyclopentadienyl)dimethyltitanium (**S2**) as an orange solid (2.9 g, 99%); The

product was stored as a 0.5 M solution in dry toluene under argon at -20°C; ^{1}H NMR (600 MHz, $CDCl_3$) δ = 6.06 (s, 10H, Cp-CH), −0.15 (s, 6H, CH_3); ^{13}C NMR (151 MHz, $CDCl_3$) δ = 113.23 (10 CH^{Cp}), 45.61 (2 CH_3).

1.1.1.9 2,6-Anhydro-3,5,7-tri-*O*-benzyl-1,4-dideoxy-4-fluoro-D-*altro*-hept-1-enitol (8)

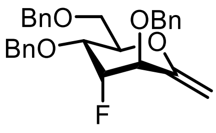

8
$C_{28}H_{29}FO_4$
448.53 g/mol

Compound **8** was synthesized following a protocol by Waschke *et al.*[10] 2,4,6-Tri-*O*-benzyl-3-deoxy-3-fluoro-D-*altrono*-1,5-lactone (**7**, 1.64 g, 3.64 mmol, 1 equiv.) was dissolved in dry toluene (18.2 mL) under an argon atmosphere and the flask was wrapped in aluminum foil. Bis(cyclopentadienyl)dimethyltitanium (**S2**, 0.5 M in dry toluene, 18.2 mL, 9.10 mmol, 2.5 equiv.) was added dropwise and the flask was equipped with a reflux condenser. The mixture was stirred for 16 h at 70°C before being cooled to room temperature again. The dark red solution was diluted with *n*-heptane (60 mL) and stirring was continued for 30 min. The resulting orange suspension was then filtered through a pad of Celite® and the filtrate was evaporated. The crude residue was purified by MPLC (100 g silica gel, 2-20% EA in *n*-heptane) yielding 2,6-anhydro-3,5,7-tri-*O*-benzyl-1,4-dideoxy-4-fluoro-D-*altro*-hept-1-enitol (**8**) as an orange oil (1.215 g, 74%); R_f = 0.32 (*n*-heptane/EA 9:1, UV & CAM); $[\alpha]_D^{20}$ = +48.0 (c 1.0 in $CHCl_3$); ^{1}H NMR (700 MHz, $CDCl_3$) δ = 7.36-7.26 (m, 15H, H^{Ar}), 4.95 (s, 1H, H-1a), 4.91 (ddd, $^{2}J_{4,F}$ = 50.6 Hz, $^{3}J_{4,3}$ = 4.6 Hz, $^{3}J_{4,5}$ = 2.1 Hz, 1H, H-4), 4.70 (d, $^{2}J_{H,H}$ = 11.9 Hz, 1H, H^{Bn}), 4.69 (d, $^{2}J_{H,H}$ = 11.9 Hz, 1H, H^{Bn}), 4.66 (d, $^{2}J_{H,H}$ = 11.5 Hz, 1H, H^{Bn}), 4.56 (s, 1H, H-1b), 4.55 (d, $^{2}J_{H,H}$ = 11.5 Hz, 1H, H^{Bn}), 4.54 (d, $^{2}J_{H,H}$ = 11.6 Hz, 1H, H^{Bn}), 4.37 (d, $^{2}J_{H,H}$ = 11.6 Hz, 1H, H^{Bn}), 4.17 (ddd, $^{3}J_{5,F}$ = 28.4 Hz, $^{3}J_{5,6}$ = 9.5 Hz, $^{3}J_{5,4}$ = 2.1 Hz, 1H, H-5), 4.10 (dd, $^{3}J_{3,F}$ = 5.7 Hz, $^{3}J_{3,4}$ = 4.6 Hz, 1H, H-3), 4.06 (ddd, $^{3}J_{6,5}$ = 9.5 Hz, $^{3}J_{6,7a}$ = 3.7 Hz, $^{3}J_{6,7b}$ = 2.5 Hz, 1H, H-6), 3.82 (dd, $^{2}J_{7a,7b}$ = 11.1 Hz, $^{3}J_{7a,6}$ = 3.7 Hz, 1H, H-7a), 3.79 (dd, $^{2}J_{7b,7a}$ = 11.1 Hz, $^{3}J_{7b,6}$ = 2.5 Hz, 1H); ^{13}C NMR (176 MHz, $CDCl_3$) δ = 153.32 (C-2), 138.38 (C^{Ar}), 137.77 (C^{Ar}), 137.71 (C^{Ar}), 128.57 (2 CH^{Ar}), 128.55 (2 CH^{Ar}), 128.48 (2 CH^{Ar}), 128.10 (2 CH^{Ar}), 128.01 (CH^{Ar}), 127.95 (2 CH^{Ar}), 127.93 (CH^{Ar}), 127.88 (2 CH^{Ar}), 127.70 (CH^{Ar}), 101.91 (C-1), 87.02 (d, $^{1}J_{4,F}$ = 178.1 Hz, C-4), 76.34 (d, $^{3}J_{6,F}$ = 3.0 Hz, C-6), 75.25 (d, $^{2}J_{3,F}$ = 28.6 Hz, C-3), 73.67 (CH_2^{Bn}), 72.14 (CH_2^{Bn}), 71.16 (d, $^{2}J_{5,F}$ = 16.6 Hz, C-5), 70.06 (CH_2^{Bn}), 69.24 (C-7); $^{19}F\{^{1}H\}$ NMR (659 MHz, $CDCl_3$) δ = −206.16 (s); ^{19}F NMR (659 MHz, $CDCl_3$) δ = −206.16 (ddd, $^{2}J_{F,4}$ = 50.6 Hz, $^{3}J_{F,5}$ = 28.4 Hz, $^{3}J_{F,3}$ = 5.3 Hz); HRMS (ESI+): *m/z* calc. for $C_{28}H_{29}O_4FNa^+$ $[M+H_2O+Na]^+$: 471.1942, found: 471.1936.

1.1.1.10 3,5,7-Tri-*O*-benzyl-4-deoxy-4-fluoro-D-*glycero*-D-*arabino*-hept-2-ulopyranose (9)

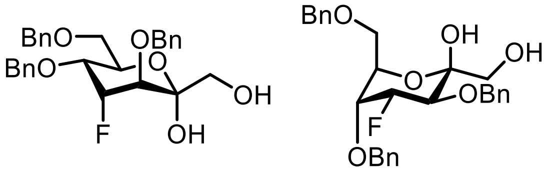

9

$C_{28}H_{31}FO_6$

482.55 g/mol

Compound **9** was synthesized following a protocol by Leshch *et al.*[11] 2,6-Anhydro-3,5,7-tri-*O*-benzyl-1,4-dideoxy-4-fluoro-D-*altro*-hept-1-enitol (**8**, 1.202 g, 2.68 mmol, 1 equiv.) was dissolved in a 4:1 (v/v) mixture of acetone and water (53.6 mL) followed by addition of *N*-methylmorpholine-*N*-oxide (NMO, 628 mg, 5.36 mmol, 2 equiv.) and potassium osmate dihydrate (49 mg, 0.13 mmol, 0.05 equiv.). The reaction mixture was stirred at room temperature for 16 h, diluted with EA and washed with water and brine. The organic layer was dried ($MgSO_4$), filtered and evaporated. The crude residue was purified by MPLC (100 g silica gel, 12-84% EA in *n*-heptane) yielding 3,5,7-tri-*O*-benzyl-4-deoxy-4-fluoro-D-*glycero*-α/β-D-*arabino*-hept-2-ulopyranose (**9**) as a brown oil (1.126 g, 87%); R_f = 0.28 (*n*-heptane/EA 1:1, UV & CAM); $[\alpha]_D^{20}$ = +32.4 (c = 1.0 in $CHCl_3$); <u>α-Pyranose ($^{5}C_2$)</u>: ^{1}H NMR (700 MHz, $CDCl_3$) δ = 7.37-7.24 (m, 15H, H^{Ar}), 4.78 (ddd, $^{2}J_{4,F}$ = 49.5 Hz, $^{3}J_{4,3}$ = 3.7 Hz, $^{3}J_{4,5}$ = 2.5 Hz, 1H, H-4), 4.65 (d, $^{2}J_{H,H}$ = 11.9 Hz, 1H, H^{Bn}), 4.59 (d, $^{2}J_{H,H}$ = 11.9 Hz, 1H, H^{Bn}), 4.55 (d, $^{2}J_{H,H}$ = 11.6 Hz, 1H, H^{Bn}), 4.53 (d, $^{2}J_{H,H}$ = 11.6 Hz, 1H, H^{Bn}), 4.53 (d, $^{2}J_{H,H}$ = 11.6 Hz, 1H, H^{Bn}), 4.52 (d, $^{2}J_{H,H}$ = 11.6 Hz, 1H, H^{Bn}), 4.25 (ddd, $^{3}J_{6,5}$ = 9.8 Hz, $^{3}J_{6,7a}$ = 4.4 Hz, $^{3}J_{6,7b}$ = 2.1 Hz, 1H, H-6), 3.90 (ddd, $^{3}J_{5,F}$ = 30.1 Hz, $^{3}J_{5,6}$ = 9.8 Hz, $^{3}J_{5,4}$ = 2.5 Hz, 1H, H-5), 3.80 (dd, $^{3}J_{3,F}$ = 5.7 Hz, $^{3}J_{3,4}$ = 3.7 Hz, 1H, H-3), 3.80 (d, $J_{OH,F}$ = 6.9 Hz, 1H, OH-2), 3.78 (dd, $^{2}J_{7a,7b}$ = 11.1 Hz, $^{3}J_{7a,6}$ = 4.4 Hz, 1H, H-7a), 3.71 (dd, $^{2}J_{7b,7a}$ = 11.1 Hz, $^{3}J_{7b,6}$ = 2.1 Hz, 1H, H-7b), 3.69 (dd, $^{2}J_{1a,1b}$ = 11.1 Hz, $^{3}J_{1a,OH}$ = 3.5 Hz, 1H, H-1a), 3.55 (t, $^{2}J_{1b,1a}$ = $^{3}J_{1b,OH}$ = 11.1 Hz, 1H, H-1b), 2.01 (dd, $^{3}J_{OH,1b}$ = 11.1 Hz, $^{3}J_{OH,1a}$ = 3.5 Hz, 1H, OH-1); ^{13}C NMR (176 MHz, $CDCl_3$) δ = 138.39 (C^{Ar}), 137.75 (C^{Ar}), 137.24 (C^{Ar}), 128.77 (2 CH^{Ar}), 128.61 (2 CH^{Ar}), 128.48 (2 CH^{Ar}), 128.45 (CH^{Ar}), 128.29 (4 CH^{Ar}), 128.13 (CH^{Ar}), 128.05 (2 CH^{Ar}), 127.76 (CH^{Ar}), 96.91 (C-2), 87.22 (d, $^{1}J_{4,F}$ = 181.0 Hz, C-4), 75.35 (d, $^{2}J_{3,F}$ = 21.5 Hz, C-3), 73.77 (CH_2^{Bn}), 73.68 (CH_2^{Bn}), 71.99 (CH_2^{Bn}), 70.81 (d, $^{2}J_{5,F}$ = 16.5 Hz, C-5), 69.21 (C-7), 67.85 (d, $^{3}J_{6,F}$ = 3.4 Hz, C-6), 65.77 (C-1); ^{19}F{^{1}H} NMR (659 MHz, $CDCl_3$) δ = −204.33 (s); ^{19}F NMR (659 MHz, $CDCl_3$) δ= −204.33 (ddt, $^{2}J_{F,4}$ = 49.5 Hz, $^{3}J_{F,5}$ = 30.1 Hz, $^{3}J_{F,3}$ = $J_{F,OH}$ = 6.9 Hz); <u>β-Pyranose ($^{2}C_5$)</u>: ^{1}H NMR (700 MHz, $CDCl_3$) δ = 7.37-7.24 (m, 15H, H^{Ar}), 5.00 (ddd, $^{2}J_{4,F}$ = 49.3 Hz, $^{3}J_{4,3}$ = 7.7 Hz, $^{3}J_{4,5}$ = 3.2 Hz, 1H, H-4), 4.80 (d, $^{2}J_{H,H}$ = 11.0 Hz, 1H, H^{Bn}), 4.68 (d, $^{2}J_{H,H}$ = 11.9 Hz, 1H, H^{Bn}), 4.66 (d, $^{2}J_{H,H}$ = 11.0 Hz, 1H, H^{Bn}), 4.62 (d, $^{2}J_{H,H}$ = 11.9 Hz, 1H, H^{Bn}), 4.55 (d, $^{2}J_{H,H}$ = 12.3 Hz, 1H, H^{Bn}), 4.50 (d, $^{2}J_{H,H}$ = 12.3 Hz, 1H, H^{Bn}), 4.15 (dq, $^{3}J_{6,7a}$ = 6.2 Hz, $^{3}J_{6,5}$ = $^{3}J_{6,7b}$ = $^{4}J_{6,F}$ = 4.2 Hz, 1H, H-6), 4.09 (dd, $^{3}J_{3,F}$ = 10.1 Hz, $^{3}J_{3,4}$ = 7.7 Hz, 1H, H-3), 4.07 (ddd, $^{3}J_{5,F}$ = 14.9 Hz, $^{3}J_{5,6}$ = 4.2, $^{3}J_{5,4}$ = 3.2 Hz, 1H, H-5), 3.79 (s, 1H, OH-2), 3.68 (dd, $^{2}J_{7a,7b}$ = 10.4 Hz, $^{3}J_{7a,6}$ = 6.2 Hz, 1H, H-7a), 3.64 (dd, $^{2}J_{7b,7a}$ = 10.4 Hz, $^{3}J_{7b,6}$ = 4.2 Hz, 1H, H-7b), 3.58 (dd, $^{2}J_{1a,1b}$ = 11.6 Hz, $^{3}J_{1a,OH}$ = 7.2 Hz, 1H, H-1a), 3.57 (dd, $^{2}J_{1b,1a}$ = 11.6 Hz, $^{3}J_{1b,OH}$ = 6.9 Hz, 1H, H-1b), 1.96 (t, $^{3}J_{OH,1a}$ = 7.2 Hz, $^{3}J_{OH,1b}$ = 6.9 Hz, 1H, OH-1); ^{13}C NMR (176 MHz, $CDCl_3$) δ = 137.96 (C^{Ar}), 137.73 (C^{Ar}), 137.43 (C^{Ar}), 128.72 (2 CH^{Ar}), 128.64 (2 CH^{Ar}), 128.57 (2 CH^{Ar}), 128.56 (2 CH^{Ar}), 128.38 (CH^{Ar}), 128.03 (CH^{Ar}), 128.02 (2 CH^{Ar}), 127.99 (CH^{Ar}), 127.93 (2

CH^{Ar}), 98.25 (d, $^{3}J_{2,F}$ = 6.4 Hz, C-2), 89.76 (d, $^{1}J_{4,F}$ = 184.6 Hz, C-4), 74.87 (d, $^{4}J_{C,F}$ = 2.1 Hz, CH$_2^{Bn}$), 74.56 (d, $^{2}J_{3,F}$ = 20.0 Hz, C-3), 74.41 (d, $^{3}J_{6,F}$ = 5.8 Hz, C-6), 74.17 (d, $^{2}J_{5,F}$ = 15.8 Hz, C-5), 73.59 (CH$_2^{Bn}$), 72.75 (d, $^{4}J_{C,F}$ = 1.8 Hz, CH$_2^{Bn}$), 70.29 (C-7), 65.32 (d, $^{4}J_{1,F}$ = 3.4 Hz, C-1); ^{19}F{^{1}H} NMR (659 MHz, CDCl$_3$) δ = −205.36 (s); ^{19}F NMR (659 MHz, CDCl$_3$) δ = −205.36 (dddd, $^{2}J_{F,4}$ = 49.3 Hz, $^{3}J_{F,5}$ = 14.2 Hz, $^{3}J_{F,3}$ = 10.4, $^{4}J_{F,6}$ = 4.2 Hz); HRMS (ESI+): *m/z* calc. for $C_{28}H_{31}O_6FNa^+$ [M+Na]$^+$: 505.1997, found: 505.1998.

1.1.1.11 4-Deoxy-4-fluoro-D-sedoheptulose/ 4-Deoxy-4-fluoro-D-*glycero*-D-*arabino*-hept-2-ulopyranose & 4-deoxy-4-fluoro-D-*glycero*-D-*arabino*-hept-2-ulofuranose (4DFS)

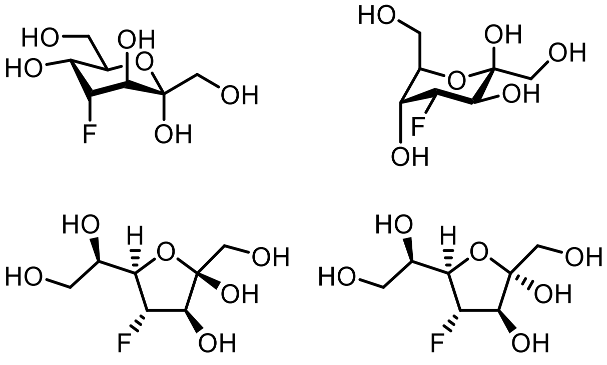

4DFS
$C_7H_{13}FO_6$
212.17 g/mol

3,5,7-Tri-*O*-benzyl-4-deoxy-4-fluoro-D-*glycero*-α/β-D-*arabino*-hept-2-ulopyranose (**9**, 170 mg, 0.35 mmol, 1 equiv.) was dissolved in methanol (5 mL) and Pd/C (10%, 60 mg, 0.04 mmol, 0.1 equiv.) was added. The black suspension was degassed (3 freezing/thawing cycles with liquid N_2 under vacuum) and subsequently stirred at room temperature for 24 h (completion of the reaction was verified by absence of aromatic proton signals in *d$_4$*-methanol after drying a small portion of the reaction mixture in high vacuum). The mixture was filtered, evaporated, and purified by reversed-phase chromatography (C$_{18}$-modified silica gel, H_2O) yielding 4-deoxy-4-fluoro-D-sedoheptulose (**4DFS**) as a colourless oil and as a mixture of α- and β-furanoses and α- and β-pyranoses in a 1.5:4:4:1 ratio (60 mg, 80%); R_f = 0.7 (1-BuOH/acetone/H_2O 5:4:1, CAM); $[\alpha]_D^{20}$ = +23.4 (c = 1.0 in H_2O); β-Furanose: ^{1}H NMR (700 MHz, D_2O) δ = 5.17 (dt, $^{2}J_{4,F}$ = 56.4 Hz, $^{3}J_{4,3}$ = 6.1 Hz, $^{3}J_{4,5}$ = 5.1 Hz, 1H, H-4), 4.42 (dd, $^{3}J_{3,F}$ = 22.7 Hz, $^{3}J_{3,4}$ = 6.1 Hz, 1H, H-3), 4.00 (ddd, $^{3}J_{5,F}$ = 21.4 Hz, $^{3}J_{5,6}$ = 7.8 Hz, $^{3}J_{5,4}$ = 5.1 Hz, 1H, H-5), 3.82 (ddd, $^{3}J_{6,5}$ = 7.8 Hz, $^{3}J_{6,7b}$ = 6.2 Hz, $^{3}J_{6,7a}$ = 3.3 Hz, 1H, H-6), 3.76 (dd, $^{2}J_{7a,7b}$ = 11.9 Hz, $^{3}J_{7a,6}$ = 3.3 Hz, 1H, H-7a), 3.62 (dd, $^{2}J_{7b,7a}$ = 11.9 Hz, $^{3}J_{7b,6}$ = 6.2 Hz, 1H, H-7b), 3.59 (dd, $^{2}J_{1a,1b}$ = 12.1 Hz, $^{5}J_{1a,F}$ = 0.7 Hz, 1H, H-1a), 3.55 (dd, $^{2}J_{1b,1a}$ = 12.1 Hz, $^{5}J_{1b,F}$ = 1.7 Hz, 1H, H-1b); ^{13}C NMR (176 MHz, D_2O) δ = 105.30 (d, $^{3}J_{2,F}$ = 10.0 Hz, C-2), 101.15 (d, $^{1}J_{4,F}$ = 182.28 Hz, C-4), 81.07 (d, $^{2}J_{5,F}$ = 24.4 Hz, C-5), 77.11 (d, $^{2}J_{3,F}$ = 22.3 Hz, C-3), 74.99 (d, $^{3}J_{6,F}$ = 3.6 Hz, C-6), 65.04 (C-1), 64.94 (C-7); ^{19}F{^{1}H} NMR (659 MHz, D_2O) δ = −192.63 (s); ^{19}F NMR (659 MHz, D_2O) δ = −192.63 (dt, $^{2}J_{F,4}$ = 56.4 Hz, $^{3}J_{F,3}$ = $^{3}J_{F,5}$ = 21.4 Hz); α-Pyranose ($^{5}C_2$): ^{1}H NMR (700 MHz, D_2O) δ = 4.83 (dt, $^{2}J_{4,F}$ = 48.9 Hz, $^{3}J_{4,3}$ = $^{3}J_{4,5}$ = 3.2 Hz, 1H, H-4), 4.10 (ddd, $^{3}J_{6,5}$ = 10.5 Hz, $^{3}J_{6,7b}$ = 5.8 Hz, $^{3}J_{6,7a}$ = 2.4 Hz, 1H, H-6), 4.06 (dd, $^{3}J_{3,F}$ = 6.0 Hz, $^{3}J_{3,4}$ = 3.2 Hz, 1H, H-3), 3.88 (ddd, $^{3}J_{5,F}$ = 31.1 Hz, $^{3}J_{5,6}$ = 10.5 Hz, $^{3}J_{5,4}$ = 3.2 Hz, 1H, H-5), 3.87 (dd, $^{2}J_{7a,7b}$ = 12.2 Hz, $^{3}J_{7a,6}$ = 2.4 Hz, 1H, H-7a), 3.75 (dd, $^{2}J_{7b,7a}$ = 12.2 Hz, $^{3}J_{7b,6}$ = 5.8 Hz, 1H, H-7b), 3.70 (d, $^{2}J_{1a,1b}$ = 11.7 Hz, 1H, H-1a), 3.53 (d, $^{2}J_{1b,1a}$ = 11.7

Hz, 1H, H-1b); ^{13}C NMR (176 MHz, D_2O) δ = 99.27 (C-2), 93.43 (d, $^{1}J_{4,F}$ = 179.6 Hz, C-4), 71.20 (d, $^{3}J_{6,F}$ = 2.4 Hz, C-6), 70.00 (d, $^{2}J_{3,F}$ = 23.4 Hz, C-3), 67.31 (C-1), 65.67 (d, $^{2}J_{5,F}$ = 17.5 Hz, C-5), 63.76 (C-7); ^{19}F{^{1}H} NMR (659 MHz, D_2O) δ = −202.12 (s); ^{19}F NMR (659 MHz, D_2O) δ = −202.12 (ddd, $^{2}J_{F,4}$ = 48.9 Hz, $^{3}J_{F,5}$ = 31.1 Hz, $^{3}J_{F,3}$ = 6.0 Hz); α-Furanose: ^{1}H NMR (700 MHz, D_2O) δ = 5.03 (ddd, $^{2}J_{4,F}$ = 52.9 Hz, $^{3}J_{4,5}$ = 3.8 Hz, $^{3}J_{4,3}$ = 2.2 Hz, 1H, H-4), 4.33 (dd, $^{3}J_{3,F}$ = 16.2 Hz, $^{3}J_{3,4}$ = 2.2 Hz, 1H, H-3), 4.24 (ddd, $^{3}J_{5,F}$ = 24.0 Hz, $^{3}J_{5,6}$ = 6.7 Hz, $^{3}J_{5,4}$ = 3.8 Hz, 1H, H-5), 3.80 (td, $^{3}J_{6,5}$ = $^{3}J_{6,7b}$ = 6.7 Hz, $^{3}J_{6,7a}$ = 3.6 Hz, 1H, H-6), 3.74 (ddd, $^{2}J_{7a,7b}$ = 12.0 Hz, $^{3}J_{7a,6}$ = 3.6 Hz, $^{5}J_{7a,F}$ = 0.4 Hz, 1H, H-7a), 3.72 (d, $^{2}J_{1a,1b}$ = 12.0 Hz, 1H, H-1a), 3.66 (d, $^{2}J_{1b,1a}$ = 12.0 Hz, 1H, H-1b), 3.62 (dd, $^{2}J_{7b,7a}$ = 12.0 Hz, $^{3}J_{7b,6}$ = 6.7 Hz, 1H, H-7b); ^{13}C NMR (176 MHz, D_2O) δ = 108.28 (d, $^{3}J_{2,F}$ = 4.2 Hz, C-2), 100.47 (d, $^{1}J_{4,F}$ = 183.0 Hz, C-4), 84.08 (d, $^{2}J_{5,F}$ = 25.7 Hz, C-5), 81.32 (d, $^{2}J_{3,F}$ = 23.1 Hz, C-3), 73.73 (d, $^{3}J_{6,F}$ = 5.5 Hz, C-6), 65.06 (C-1), 65.02 (C-7); ^{19}F{^{1}H} NMR (659 MHz, D_2O) δ = −182.45 (s); ^{19}F NMR (659 MHz, D_2O) δ = −182.45 (ddd, $^{2}J_{F,4}$ = 52.9 Hz, $^{3}J_{F,5}$ = 24.0 Hz, $^{3}J_{F,3}$ = 16.2 Hz); β-Pyranose ($^{2}C_{5}$): ^{1}H NMR (700 MHz, D_2O) δ = 4.93 (ddd, $^{2}J_{4,F}$ = 48.9 Hz, $^{3}J_{4,3}$ = 8.3 Hz, $^{3}J_{4,5}$ = 3.5 Hz, 1H, H-4), 4.27 (dt, $^{3}J_{5,F}$ = 13.8 Hz, $^{3}J_{5,4}$ = $^{3}J_{5,6}$ = 3.5 Hz, 1H, H-5), 4.14 (dd, $^{3}J_{3,F}$ = 10.9 Hz, $^{3}J_{3,4}$ = 8.3 Hz, 1H, H-3), 3.99 (dddd, $^{3}J_{6,7a}$ = 6.3 Hz, $^{3}J_{6,7b}$ = 6.0 Hz, $^{4}J_{6,F}$ = 4.3 Hz, $^{3}J_{6,5}$ = 3.5 Hz, 1H, H-6), 3.80 (dd, $^{2}J_{7a,7b}$ = 12.0 Hz, $^{3}J_{7a,6}$ = 6.3 Hz, 1H, H-7a), 3.77 (dd, $^{2}J_{7b,7a}$ = 12.0 Hz, $^{3}J_{7b,6}$ = 6.0 Hz, 1H, H-7b), 3.71 (dd, $^{2}J_{1a,1b}$ = 11.9 Hz, $^{5}J_{1a,F}$ = 1.0 Hz, 1H, H-1a), 3.57 (dd, $^{2}J_{1b,1a}$ = 11.9 Hz, $^{5}J_{1b,F}$ = 1.9 Hz, 1H, H-1b); ^{13}C NMR (176 MHz, D_2O) δ = 101.58 (d, $^{3}J_{2,F}$ = 6.5 Hz, C-2), 92.94 (d, $^{1}J_{4,F}$ = 178.3 Hz, C-4), 80.52 (d, $^{3}J_{6,F}$ = 5.6 Hz, C-6), 69.03 (d, $^{2}J_{5,F}$ = 16.6 Hz, C-5), 68.64 (d, $^{2}J_{3,F}$ = 20.5 Hz, C-3), 65.81 (d, $^{5}J_{1,F}$ = 3.4 Hz, C-1), 64.74 (C-7); ^{19}F{^{1}H} NMR (659 MHz, D_2O) δ = −205.02 (s); ^{19}F NMR (659 MHz, D_2O) δ = −205.02 (dtd, $^{2}J_{F,4}$ = 48.9 Hz), $^{3}J_{F,5}$ =13.8 Hz, $^{3}J_{F,3}$ = 10.9 Hz, $^{4}J_{F,6}$ = 4.3 Hz); HRMS (ESI+): *m/z* calc. for $C_7H_{13}O_6FNa^+$ $[M+Na]^+$: 235.0588, found: 235.0593.

1.1.2 Synthesis of 3-deoxy-3-fluoro-D-sedoheptulose (3DFS)

1.1.2.1 Methyl 4,6-*O*-benzylidene-2,3-di-*O*-*para*-toluenesulfonyl-α-D-*gluco*-pyranoside[12] (10)

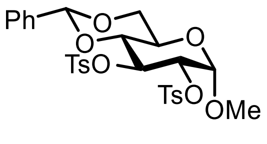

10

$C_{28}H_{30}O_{10}S_2$

590.66 g/mol

para-Toluenesulfonyl chloride (6.75 g, 35.43 mmol, 5 equiv.) was added to a solution of methyl 4,6-*O*-benzylidene-α-D-*gluco*-pyranoside (**1**, 2.0 g, 7.02 mmol, 1 equiv.) in pyridine (18 mL). The resulting solution was stirred at 50°C for 16 h. After the solvent was removed *in vacuo*, the remaining pyridine was co-evaporated with toluene (2 × 15 mL). The residue was dissolved in water and extracted with DCM (3 × 15 mL). The product precipitated upon addition of ethanol, to yield **10** as a colourless solid (3.93 g, 94%); $[\alpha]_D^{20}$ = +18.6 (c = 1.0 in $CHCl_3$) (lit.,[12] 14.9 (*c* = 1.0 in $CHCl_3$)); ^{1}H NMR (600 MHz, $CDCl_3$) δ = 7.81 (d, *J* = 8.3 Hz, 2H, H^{Ar}), 7.62 (d, *J* = 8.3 Hz, 2H, H^{Ar}), 7.41-7.29 (m, 5H, H^{Ar}), 7.25 (dd, *J* = 8.3 Hz, *J* = 1.5 Hz, 2H, H^{Ar}), 6.93 (dd, *J* = 8.2 Hz, *J* = 0.5 Hz, 2H, H^{Ar}), 5.28 (s, 1H, H-7), 5.09 (dd, $^{3}J_{3,4}$ = 9.6 Hz, $^{3}J_{3,2}$ = 9.6 Hz, 1H, H-3), 5.04 (d, $^{3}J_{1,2}$ = 3.6 Hz, 1H, H-1), 4.42 (dd, $^{3}J_{2,3}$ = 9.6 Hz, $^{3}J_{2,1}$ = 3.6 Hz, 1H, H-2), 4.25 (dd, $^{2}J_{6a,6b}$ = 10.5 Hz, $^{3}J_{6a,5}$ = 4.9 Hz, 1H, H-6a), 3.85 (ddd, $^{3}J_{5,4}$ = 10.0 Hz, $^{3}J_{5,6b}$ = 10.2 Hz, $^{3}J_{5,6a}$ = 4.9 Hz, 1H, H-5),

3.66 (dd, $^{2}J_{6b,6a}$ = 10.5 Hz, $^{3}J_{6b,5}$ = 10.2 Hz, 1H, H-6b), 3.50 (dd, $^{3}J_{4,3}$ = 9.6 Hz, $^{3}J_{4,5}$ = 9.6 Hz , 1H, H-4), 3.41 (s, 3H, OCH_3), 2.45 (s, 3H, CH_3^{Tos}), 2.25 (s, 3H, CH_3^{Tos}); ^{13}C NMR (151 MHz, $CDCl_3$) δ = 145.46 (CH^{Ar}), 144.37 (CH^{Ar}), 136.60 (CH^{Ar}), 134.07 (CH^{Ar}), 132.56 (C^{Ar}), 129.94 (2 C^{Ar}), 129.33 (2 CH^{Ar}), 129.26 (C^{Ar}), 128.66 (2 CH^{Ar}), 128.26 (2 C^{Ar}), 128.19 (2 CH^{Ar}), 126.44 (2 CH^{Ar}), 102.06 (C-7), 98.67 (C-1), 79.17 (C-4), 76.51 (C-3), 75.95 (C-2), 68.79 (C-6), 62.51 (C-5), 56.11 (OCH_3), 21.89 (CH_3^{Tos}), 21.80 (CH_3^{Tos}); HRMS (+ESI): *m/z* calc. for $C_{28}H_{31}O_{10}S_2H^+$ $[M+H]^+$: 591.1353, found: 591.1348.

1.1.2.2 Methyl 2,3-anhydro-4,6-*O*-benzylidene-α-D-*allo*-pyranoside[12] (11)

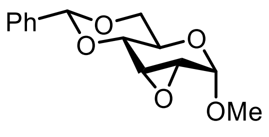

11
$C_{14}H_{16}O_5$
264.28 g/mol

Methyl 4,6-*O*-benzylidene-2,3-di-*O*-*para*-toluenesulfonyl-α-D-*gluco*-pyranoside (**10**, 5.52 g, 9.35 mmol, 1 equiv.) in DCM (74 mL) was added slowly to a freshly prepared solution of NaOMe (2.3 M in methanol, 26 mL) and the resulting mixture was stirred under argon for 19 h. After cooling the solution to 0°C, water was added and the aqueous layer was extracted with DCM (3 × 50 mL). The combined organic layers were dried ($MgSO_4$), and the solvent was evaporated to yield a white solid. The pure product **11** was obtained by recrystallization from DCM and *n*-heptane as colourless needles (2.27 g, 91%); R_f = 0.67 (*n*-heptane/EA 1:1, UV & CAM); $[\alpha]_D^{20}$ = +116.6 (c = 1.0 in $CHCl_3$) (lit.,[12] 136.7 (*c* = 1.0 in $CHCl_3$)); ^{1}H NMR (700 MHz, $CDCl_3$) δ = 7.50 (dd, *J* = 7.7 Hz, *J* = 1.7 Hz, 2H, H^{Ar}), 7.40-7.32 (m, 3H, H^{Ar}), 5.57 (s, 1H, H-7), 4.89 (d,$^{3}J_{1,2}$ = 2.9 Hz, 1H, H-1), 4.25 (dd, $^{2}J_{6a,6b}$ = 10.3 Hz, $^{3}J_{6a,5}$ = 5.1 Hz, 1H, H-6a), 4.09 (ddd, $^{3}J_{5,6b}$ = 10.3 Hz, $^{3}J_{5,4}$ = 9.6 Hz, $^{3}J_{5,6a}$ = 5.1 Hz, 1H, H-5), 3.96 (dd, $^{3}J_{4,5}$ = 9.6 Hz, $^{3}J_{4,3}$ = 1.2 Hz, 1H, H-4), 3.69 (dd, $^{2}J_{6b,6a}$ = 10.3 Hz, $^{3}J_{6b,5}$ = 10.3 Hz, 1H, H-6b), 3.53 (dd, $^{3}J_{3,2}$ = 4.3 Hz, $^{3}J_{3,4}$ = 1.2 Hz, 1H, H-3), 3.50 (dd, $^{3}J_{2,3}$ = 4.3 Hz, $^{3}J_{2,1}$ = 2.9 Hz, 1H, H-2), 3.48 (s, s, 3H, OCH_3); ^{13}C NMR (176 MHz, $CDCl_3$) δ = 137.11 (CH^{Ar}), 129.26 (C^{Ar}), 128.34 (2 C^{Ar}), 126.31 (2 C^{Ar}), 102.79 (C-7), 95.31 (C-1), 77.88 (C-4), 68.91 (C-6), 60.03 (C-5), 55.90 (OCH_3), 53.13 (C-3), 50.72 (C-2); HRMS (+ESI): *m/z* calc. for $C_{14}H_{16}O_5Na^+$ $[M+Na]^+$: 287.0890, found: 287.0888.

1.1.2.3 Methyl 4,6-*O*-benzylidene-2-deoxy-2-fluoro-α-D-*altro*-pyranoside[5] (12)

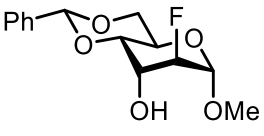

12
$C_{14}H_{17}FO_5$
284.28 g/mol

Methyl 2,3-anhydro-4,6-*O*-benzylidene-α-D-*allo*-pyranoside (**11**, 1.98 g, 7.49 mmol, 1 equiv.), *N,N,N,N*-tetrabutylammonium fluoride trihydrate (19.17 g, 59.92 mmol, 8 equiv.) and KHF_2 (2.34 g, 29.96 mmol, 4 equiv.) were stirred at 115°C under Ar atmosphere in a teflon flask. After 8 h, the reaction mixture was cooled to rt and diluted with EA (100 mL). A sat. aqueous $NaHCO_3$-sol. (400 mL) was added and the layers were separated. The aqueous layer was extracted with EA (2 × 50 mL) the combined organic layers were washed with brine (2 × 50 mL) and dried ($MgSO_4$). The solvent was removed under reduced pressure and the resulting crude residue was purified by MPLC (100 g silica gel, 12-100% EA in *n*-heptane) to yield **12** as a colourless oil (1.43 g, 67%); R_f = 0.79 (*n*-heptane/EA 1:1, UV & CAM); $[\alpha]_D^{20}$ = +134.6 (*c* = 1.0 in $CHCl_3$) (lit.,[5] 142.2 (*c* =

1.0 in $CHCl_3$)); ^{1}H NMR (700 MHz, $CDCl_3$) δ = 7.50-7.35 (m, 5H, H^{Ar}), 5.66 (s, 1H, H-7), 4.84 (d, $^3J_{1,F}$ = 10.4 Hz, 1H, H-1), 4.69 (ddd, $^2J_{2,F}$ = 43.6 Hz, $^3J_{2,3}$ = 3.2 Hz, $^3J_{2,1}$ = 1.0 Hz, 1H, H-2), 4.36 (dd, $^2J_{6a,6b}$ = 10.3 Hz, $^3J_{6a,5}$ = 5.2 Hz, 1H, H-6a), 4.29-4.33 (m, 1H, H-3), 4.24 (ddd, $^3J_{5,4}$ = 10.0 Hz, $^3J_{5,6b}$ = 10.2 Hz, $^3J_{5,6a}$ = 5.2 Hz, 1H, H-5), 3.92 (ddd, $^3J_{4,5}$ = 9.8 Hz , $^3J_{4,3}$ = 3.1 Hz, $^4J_{4,F}$ = 3.1 Hz, 1H, H-4), 3.84 (dd, $^2J_{6b,6a}$ = 10.3 Hz, $^3J_{6b,5}$ = 10.2 Hz, 1H, H-6b), 3.48 (s, OCH_3), 2.62 (dd, $^3J_{OH,3}$ = 5.5 Hz, $^4J_{OH,F}$ = 1.3 Hz, 1H, H-OH); ^{13}C NMR (176 MHz, $CDCl_3$) δ = 137.06 (CH^{Ar}), 129.27 (C^{Ar}), 128.34 (2 CH^{Ar}), 126.21 (2 CH^{Ar}), 102.30 (C-7), 98.96 (d, $^3J_{1,F}$ = 33.2 Hz, C-1), 87.61 d, $^2J_{2,F}$ = 174.0 Hz, C-2), 76.06 (d, $^4J_{4,F}$ = 1.1 Hz, C-4), 69.07 (C-6), 66.50 (d, $^3J_{3,F}$ = 27.6 Hz, C-3), 58.10 (C-5), 55.97 (OCH_3); ^{19}F {^{1}H} NMR (659 MHz, $CDCl_3$) δ = –194.10 (s); ^{19}F NMR (659 MHz, $CDCl_3$) δ = –194.10 (m); HRMS (+ESI): *m/z* calc. for $C_{14}H_{17}O_5FNa^+$ $[M+Na]^+$: 307.0953, found: 307.0953.

1.1.2.4 Methyl 4-*O*-benzyl-2-deoxy-2-fluoro-α-D-*altro*-pyranoside (13a) & methyl 6-*O*-benzyl-2-deoxy-2-fluoro-α-D-*altro*-pyranoside (13b)

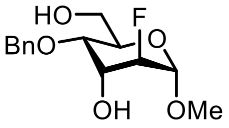

13a
$C_{14}H_{19}FO_5$
286.30 g/mol

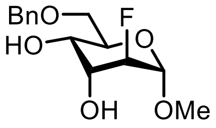

13b
$C_{14}H_{19}FO_5$
286.30 g/mol

Compounds **13a** and **13b** were synthesized following a protocol by Shie *et al.*[6] Methyl 4,6-*O*-benzyliden-2-deoxy-2-fluoro-α-D-*altro*-pyranoside (**12**, 151 mg, 0.53 mmol, 1 equiv.) was dissolved in a borane THF complex solution (1 M in THF, 2.66 mL, 0.04 mmol, 5 equiv.) under argon atmosphere at 0°C. The resulting mixture was stirred for 10 minutes before $Cu(OTf)_2$ (9.6 mg, 0.03 mmol, 0.05 equiv.) was added. After 2 h the reaction mixture was cooled to 0°C and triethylamine (0.08 mL), followed by MeOH (0.95 mL) was added. The solvents were removed *in vacuo* and the residue was co-evaporated with methanol. Purification *via* MPLC (25 g silica gel, 12-100% EA in *n*-heptane) gave the desired product as a clear oily mixture of methyl 4-*O*-benzyl-2-deoxy-2-fluoro-α-D-*altro*-pyranoside (**13a**) and methyl 6-*O*-benzyl-2-deoxy-2-fluoro-α-D-*altro*-pyranoside (**13b**) in a 1.35 : 1 ratio (141 mg, 93%)[6]; R_f = 0.24 (*n*-heptane/EA 1:1, UV & CAM); $[\alpha]_D^{20}$ = +105.2 (c = 1.0 in $CHCl_3$); **13b**: ^{1}H NMR (700 MHz, $CDCl_3$) δ = 7.38-7-28 (m, 5H, H^{Ar}), 4.87 (d, $^3J_{1,F}$ = 9.0 Hz, 1H, H-1), 4.66 (ddd, $^3J_{2,F}$ = 44.5 Hz, $^3J_{2,3}$ = 3.5 Hz, $^3J_{2,1}$ = 1.6 Hz, 1H, H-2), 4.63 (s, 2H, H^{Bn}), 4.12 (dddd, $^3J_{3,F}$ = 9.8 Hz, $^3J_{3,OH}$ = 8.9 Hz, $^3J_{3,4}$ = 6.5 Hz, $^3J_{3,2}$ = 3.5 Hz, 1H, H-3), 3.86 (ddd, $^3J_{5,4}$ = 8.4 Hz, $^3J_{5,6b}$ = 5.8 Hz, $^3J_{5,6a}$ = 2.8 Hz, 1H, H-5), 3.85 (dd, $^2J_{6a,6b}$ = 11.5 Hz, $^3J_{6a,5}$ = 2.8 Hz, 1H, H-6a), 3.78 (dddd, $^3J_{4,5}$ = 8.4 Hz, $^3J_{4,OH}$ = 8.2 Hz, $^3J_{4,3}$ = 6.5 Hz, $^4J_{4,F}$ = 2.3 Hz, 1H, H-4), 3.77 (dd, $^2J_{6b,6a}$ = 11.5 Hz, $^3J_{6b,5}$ = 5.6 Hz, 1H, H-6b), 3.47 (s, OCH_3), 3.00 (d, $^3J_{OH,3}$ = 8.9 Hz, 1H, OH-3), 2.68 (d, $^3J_{OH,4}$ = 8.2 Hz, 1H, OH-4); ^{13}C NMR (176 MHz, $CDCl_3$) δ = 137.94 (C^{Ar}), 128.42 (2 CH^{Ar}), 128.03 (CH^{Ar}), 127.63 (2 CH^{Ar}), 98.13 (d, $^3J_{1,F}$ = 31.0 Hz, C-1), 86.18 (d, $^2J_{2,F}$ = 175.1 Hz, C-2), 73.68 (CH_2^{Bn}), 71.38 (d, $^3J_{4,F}$ = 1.2 Hz, C-4), 70.29 (C-6), 67.93 (d, $^3J_{3,F}$ = 27.3 Hz, C-3), 67.30 (C-5), 55.76 (OCH_3); ^{19}F {^{1}H} NMR (659 MHz, $CDCl_3$) δ = – 196.55 (s); ^{19}F NMR (659 MHz, $CDCl_3$) δ = – 196.55 (m); **13a**: ^{1}H NMR (700 MHz, $CDCl_3$) δ = 7.38-7.28 (m, 5H, H^{Ar}), 4.82 (d, $^3J_{1,F}$ = 10.4 Hz, 1H, H-1), 4.70 (d, $^2J_{H,H}$ = 11.4 Hz, 1H, H^{Bn}), 4.66 (ddd, $^3J_{2,F}$ = 44.5 Hz, $^3J_{2,3}$ = 3.5 Hz, $^3J_{2,1}$ = 1.6 Hz, 1H, H-2), 4.58 (d, $^2J_{H.H}$ = 11.4 Hz, 1H, H^{Bn}), 4.28 (dddd, $^3J_{3,F}$ = 9.8 Hz, $^3J_{3,OH}$

= 6.5 Hz,$^3J_{3,4}$ = 3.5 Hz, $^3J_{3,2}$ = 3.5 Hz, 1H, H-3), 3.95 (ddd, $^3J_{4,5}$ = 9.9 Hz, $^3J_{4,3}$ = 3.5 Hz, $^4J_{4,F}$ = 3.5 Hz, 1H, H-4), 3.89 (ddd, $^2J_{6a,6b}$ = 11.7 Hz, $^3J_{6a,OH}$ = 5.2 Hz, $^3J_{6a,5}$ = 2.9 Hz, 1H, H-6a), 3.81 (ddd, $^2J_{6b,6a}$ = 11.7 Hz, $^3J_{6b,OH}$ = 7.9 Hz, $^3J_{6b,5}$ = 4.3 Hz, 1H, H-6b), 3.81 (ddd, $^3J_{5,4}$ = 9.9 Hz, $^3J_{5,6b}$ = 4.3 Hz, $^3J_{5,6a}$ = 2.9 Hz, 1H, H-5), 3.44 (s, OCH_3), 2.77 (d, $^3J_{OH,3}$ = 6.5 Hz, 1H, OH-3), 1.87 (dd, $^3J_{OH,6b}$ = 7.9 Hz, $^3J_{OH,6a}$ = 5.2 Hz, 1H, OH-6); ^{13}C NMR (176 MHz, $CDCl_3$) δ = 137.33 (C^{Ar}), 128.61 (2 CH^{Ar}), 128.18 (CH^{Ar}), 127.72 (2 CH^{Ar}), 98.49 (d, $^3J_{1,F}$ = 32.0 Hz, C-1), 87.08 (d, $^2J_{2,F}$ = 172.8 Hz, C-2), 71.44 (CH_2^{Bn}), 66.28 (C-4), 65.56 (d, $^3J_{3,F}$ = 27.7 Hz, C-3), 65.20 (C-5), 62.28 (C-6), 55.79 (OCH_3); ^{19}F {^{1}H} NMR (700 MHz, $CDCl_3$) δ = –195.55 (s); ^{19}F NMR (700 MHz, $CDCl_3$) δ = –195.55 (m); HRMS (+ESI): *m/z* calc. for $C_{14}H_{19}O_5FNa^+$ $[M+Na]^+$: 309.1109, found: 309.1112.

1.1.2.5 Methyl 3,4,6-tri-*O*-benzyl-2-deoxy-2-fluoro-α-D-*altro*-pyranoside (14)

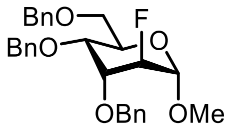

14
$C_{28}H_{31}FO_5$
466.55 g/mol

Sodium hydride (35.6 mg, 1.49 mmol, 4 equiv.) was suspended in dry THF (1.6 mL) under argon atmosphere and cooled to 0°C. Stirring was continued for 10 min after addition of a solution of the methyl 4-*O*-benzyl-2-deoxy-2-fluoro-α-D-*altro*-pyranoside and methyl 6-*O*-benzyl-2-deoxy-2-fluoro-α-D-*altro*-pyranoside mixture (**13a** & **13b**, 106.3 mg, 0.37 mmol, 1 equiv.) in THF (2.5 mL),. Then, benzyl bromide (187 mg, 0.13 mL, 1.49 mmol, 3 equiv.) was added and the resulting mixture was stirred for 10 minutes at 0°C followed by 15 h at room temperature. H_2O (10 mL) was added, the two layers were separated, and the aqueous layer was extracted with EA (3 × 10 mL). The crude residue was purified *via* MPLC (25 g silica gel, 12-100% EA in *n*-heptane) to yield methyl 3,4,6-tri-*O*-benzyl-2-deoxy-2-fluoro-α-D-*altro*-pyranoside as a colourless oil (**14**, 148 mg, 86%); R_f = 0.78 (*n*-heptane/EA 1:1, UV & CAM); $[\alpha]_D^{20}$ = +85.7 (c = 1.0 in $CHCl_3$); ^{1}H NMR (600 MHz, $CDCl_3$) δ = 7.35-7.21 (m, 15 H, H^{Ar}), 4.71 (d, $^2J_{1,F}$ = 13.3 Hz, 1H, H-1), 4.71 (d, $^2J_{H,H}$ = 12.4 Hz, 1H, H^{Bn}), 4.67 (ddd, $^2J_{2,F}$ = 45.7 Hz, $^3J_{2,3}$ = 4.3 Hz, $^3J_{2,1}$ = 1.3 Hz, 1H, H-2), 4.65 (d, $^2J_{H,H}$ = 12.4 Hz, 1H, H^{Bn}), 4.63 (d, $^2J_{H,H}$ = 12.3 Hz, 1H, H^{Bn}), 4.53 (d, $^2J_{H,H}$ = 12.3 Hz, 1H, H^{Bn}), 4.52 (d, $^2J_{H,H}$ = 11.8 Hz, 1H, H^{Bn}), 4.46 (d, $^2J_{H,H}$ = 11.8 Hz, 1H, H^{Bn}), 4.25 (ddd, $^3J_{5,4}$ = 8.5 Hz, $^3J_{5,6a}$ = 4.2 Hz, $^3J_{5,6b}$ = 2.8 Hz, 1H, H-5), 3.95 (ddd, $^3J_{3,F}$ = 8.2 Hz, $^3J_{3,2}$ = 4.3 Hz, $^3J_{3,4}$ = 3.2 Hz, 1H, H-3), 3.86 (ddd, $^3J_{4,5}$ = 8.5 Hz, $^3J_{4,3}$ = 3.2 Hz, $^4J_{4,F}$ = 3.1 Hz, 1H, H-4), 3.73 (dd, $^2J_{6a,6b}$ = 10.8 Hz, $^3J_{6a,5}$ = 4.2 Hz, 1H, H-6a), 3.70 (dd, $^2J_{6b,6a}$ = 10.8 Hz, $^3J_{6b,5}$ = 2.8 Hz, 1H, H-6b), 3.43 (s, OCH_3); ^{13}C NMR (151 MHz, $CDCl_3$) δ = 138.18 (C^{Ar}), 138.06 (C^{Ar}), 138.04 (C^{Ar}), 128.34 (2 CH^{Ar}), 128.31 (2 CH^{Ar}), 128.30 (2 CH^{Ar}), 127.79 (2 CH^{Ar}), 127.78 (2 CH^{Ar}), 127.70 (3 CH^{Ar}), 127.65 (CH^{Ar}), 127.53 (CH^{Ar}), 99.34 (d, $^3J_{1,F}$ = 33.02 Hz, C-1), 87.89 (d, $^2J_{2,F}$ = 173.8 Hz, C-2), 73.50 (CH_2^{Bn}), 72.75 (d, $^3J_{3,F}$ = 25.17 Hz, C-3), 72.65 (CH_2^{Bn}), 72.56 (C-4), 71.83 (CH_2^{Bn}), 69.36 (C-6), 67.97 (C-5), 55.61 (OCH_3); ^{19}F {^{1}H} NMR (659 MHz, $CDCl_3$) δ = – 194.69 (s); ^{19}F NMR (659 MHz, $CDCl_3$) δ = – 194.69 (dddd, $^2J_{F,2}$ = 45.7 Hz, $^3J_{F,1}$ = 13.3 Hz, $^3J_{F,3}$ = 8.2 Hz, $^4J_{F,4}$ = 3.1 Hz); HRMS (+ESI): *m/z* calc. For $C_{28}H_{31}O_5Fna^+$ $[M+Na]^+$: 489.2048, found: 489.2045.

1.1.2.6 3,4,6-Tri-*O*-benzyl-2-deoxy-2-fluoro-α-D-*altrono*-1,5-lactone (15)

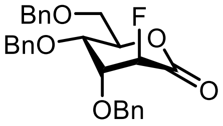

15
$C_{27}H_{27}FO_5$
450.51 g/mol

Compound **15** was synthesized following a protocol by Shi *et al.*[13] Methyl 3,4,6-tri-*O*-benzyl-2-deoxy-2-fluoro-α-D-*altro*-pyranoside (**14**, 100 mg, 0.214 mmol, 1 equiv.) was dissolved in glacial acetic acid (1.2 mL) and heated to 70°C. After the addition of 5 M aqueous HCl (0.2 mL) and strontium chloride hexahydrate (5.7 mg, 0.011 mmol, 0.1 equiv.), stirring was continued at 70°C for 3 h. The reaction mixture was diluted with water (10 mL), extracted with DCM (3 × 15 mL) and washed with a sat. aqueous $NaHCO_3$-sol. (3 × 15 mL). The combined organic layers were dried ($MgSO_4$), and the solvent was concentrated *in vacuo (*25°C). The crude residue was cooled to 0°C, DMP (145.08 mg, 0.34 mmol, 1.5 equiv.) was added, and stirring was continued at room temperature for 3 h. The reaction mixture was diluted with DCM (10 mL) and washed thrice with a 1:1 (v/v) mixture of sat. aqueous $NaHCO_3$/sat. aqueous $Na_2S_2O_3$-sol. (15 mL). After drying ($MgSO_4$), the solvent was evaporated and the crude residue was purified by MPLC (25 g silica gel, 5-50% EA in *n*-heptane) to yield 3,4,6-tri-*O*-benzyl-2-deoxy-2-fluoro-α-D-*altrono*-1,5-lactone as a colourless oil (**15**, 50 mg, 52%); R_f = 0.70 (*n*-heptane/EA 1:1, UV & CAM); $[\alpha]_D^{20}$ = – 54.2 (*c* = 1.0 in $CHCl_3$); ^{1}H NMR (700 MHz, $CDCl_3$) δ = 7.42-7.16 (m, 15H, H^{Ar}), 5.28 (dd, $^2J_{2,F}$ = 48.2 Hz, $^3J_{2,3}$ = 9.0 Hz, 1H, H-2), 4.74 (d, $^2J_{H,H}$ = 12.0 Hz, 2H, H^{Bn}), 4.64 (d, $^2J_{H,H}$ = 12.0 Hz, 1H, H^{Bn}), 4.58 (d, $^2J_{H,H}$ = 11.9 Hz, 1H, H^{Bn}), 4.58 (ddd, $^3J_{5,4}$ = 5.9 Hz, $^3J_{5,6a}$ = 4.6 Hz, $^3J_{5,6b}$ = 2.7 Hz 1H, H-5), 4.51 (d, $^2J_{H,H}$ = 11.9 Hz, 1H, H^{Bn}), 4.40 (d, $^2J_{H,H}$ = 12.0 Hz, 1H, H^{Bn}), 4.34 (ddd, $^3J_{3,F}$ = 12.0 Hz, $^3J_{3,2}$ = 9.0 Hz, $^3J_{3,4}$ = 3.1 Hz, 1H, H-3), 3.96 (ddd, $^3J_{4,5}$ = 5.9 Hz, $^3J_{4,3}$ = 3.1 Hz, $^4J_{4,F}$ = 3.1 Hz, 1H, H-4), 3.58 (dd, $^2J_{6a,6b}$ = 10.8 Hz, $^3J_{6a,5}$ = 4.6 Hz, 1H, H-6a), 3.52 (dd, $^2J_{6b,6a}$ = 10.8 Hz, $^3J_{6b,5}$ = 2.7 Hz, 1H, H-6b); ^{13}C NMR (176 MHz, $CDCl_3$) δ = 166.61 (d, $^2J_{1,F}$ = 20.0 Hz, C-1), 137.39 (C^{Ar}), 137.16 (C^{Ar}), 136.92 (C^{Ar}), 128.55 (2 CH^{Ar}), 128.54 (2 CH^{Ar}), 128.50 (2 CH^{Ar}), 128.11 (CH^{Ar}), 128.04 (2 CH^{Ar}), 127.95 (2 CH^{Ar}), 127.92 (2 CH^{Ar}), 127.69 (2 CH^{Ar}), 87.52 (d, $^1J_{2,F}$ = 189.8 Hz, C-2), 79.10 (C-5), 75.13 (d, $J_{3,F}$ = 18.8 Hz, C-3), 74.63 (d, $J_{4,F}$ = 8.71 Hz, C-4), 73.76 (CH_2^{Bn}), 73.12 (CH_2^{Bn}), 73.04 (CH_2^{Bn}), 68.97 (C-6); ^{19}F {^{1}H} NMR (659 MHz, $CDCl_3$) δ = – 201.85 (s); ^{19}F NMR (659 MHz, $CDCl_3$) δ = – 201.85 (ddd, $^2J_{F,2}$ = 48.2 Hz, $^3J_{F,3}$ = 12.0 Hz, $^4J_{F,4}$ = 3.1 Hz); HRMS (+ESI): *m/z* calc. for $C_{27}H_{27}O_5FNa^+$ $[M+Na]^+$: 473.1735, found: 473.1732.

1.1.2.7 2,6-Anhydro-4,5,7-tri-*O*-benzyl-1,3-dideoxy-3-fluoro-D-*altro*-hept-1-enitol (16)

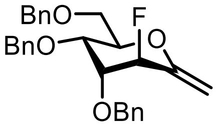

16
$C_{28}H_{29}FO_4$
448.53 g/mol

Compound **16** was synthesized following a protocol by Waschke *et al.*[10] A 0.5 M solution of Petasis' reagent (**S2**, 3.9 mL, 2.2 equiv.) in dry toluene was added under argon atmosphere in the dark to 3,4,6-tri-*O*-benzyl-2-deoxy-2-fluoro-α-D-*altrono*-1,5-lactone (**15**, 400 mg, 0.89 mmol, 1 equiv.) dissolved in dry toluene (1 mL). The mixture was stirred at 65°C for 16 h before being cooled to room temperature. The solution was diluted with *n*-heptane (20 mL) and stirring was continued for 30 min. The resulting orange suspension was then filtered over Celite®, the filtrate evaporated, and the crude residue was purified by MPLC (100 g silica gel,

10-30% Et_2O in *n*-heptane + 0.5% Et_3N) yielding 2,6-anhydro-4,5,7-tri-*O*-benzyl-1,3-dideoxy-3-fluoro-D-*altro*-hept-1-enitol as a light yellow oil (**16**, 255.5 mg, 64%); R_f = 0.62 (*n*-heptane/Et_2O 5:1, UV & CAM); $[\alpha]_D^{20}$ = +45.2 (*c* = 0.67 in $CHCl_3$); ^1H NMR (600 MHz, $CDCl_3$) δ = 7.42-7.16 (m, 15H, H^{Ar}), 4.91 (dd, $^2J_{3,F}$ = 48.5 Hz, $^3J_{3,4}$ = 5.1 Hz, 1H, H-3), 4.86 (d, $^2J_{1a,1b}$ = 5.0 Hz, 1H, H-1a), 4.72 (d, $^2J_{H,H}$ = 12.3 Hz, 1H, H^{Bn}), 4.65 (d, $^2J_{H,H}$ = 12.3 Hz, 1H, H^{Bn}), 4.63 (d, $^2J_{H,H}$ = 12.3 Hz, 1H, H^{Bn}), 4.61 (d, $^2J_{1b,1a}$ = 5.0 Hz, 1H, H-1b), 4.57 (d, $^2J_{H,H}$ = 11.6 Hz, 1H, H^{Bn}), 4.54 (d, $^2J_{H,H}$ = 12.3 Hz, 1H, H^{Bn}), 4.53 (d, $^2J_{H,H}$ = 11.6 Hz, 1H, H^{Bn}), 4.21 (ddd, $^3J_{6,5}$ = 8.0 Hz, $^3J_{6,7a}$ = 4.0 Hz, $^3J_{6,7b}$ = 3.0 Hz 1H, H-6), 4.04 (ddd, $^3J_{5,6}$ = 8.0 Hz, $^3J_{5,4}$ = 3.0 Hz, $^4J_{5,F}$ = 3.0 Hz, 1H, H-5), 3.96 (ddd, $^3J_{4,F}$ = 8.1 Hz, $^3J_{4,3}$ = 5.1 Hz, $^3J_{4,5}$ = 3.0 Hz, 1H, H-4), 3.76 (dd, $^2J_{7a,7b}$ = 10.9 Hz, $^3J_{7a,6}$ = 4.0 Hz, 1H, H-7a), 3.71 (dd, $^2J_{7b,7a}$ = 10.9 Hz, $^3J_{7b,6}$ = 3.0 Hz, 1H, H-7b); ^{13}C NMR (151 MHz, $CDCl_3$) δ = 153.50 (d, $^2J_{2,F}$ = 15.0 Hz, C-2), 138.11 (C^{Ar}), 137.89 (C^{Ar}), 137.80 (C^{Ar}), 128.41 (2 CH^{Ar}), 128.40 (2 CH^{Ar}), 128.33 (2 CH^{Ar}), 127.91 (2 CH^{Ar}), 127.85 (CH^{Ar}), 127.83 (CH^{Ar}), 127.78 (2 CH^{Ar}), 127.73 (2 CH^{Ar}), 127.60 (CH^{Ar}), 100.65 (d, $^3J_{1,F}$ = 8.9 Hz, C-1), 87.72 (d, $^1J_{3,F}$ = 176.5 Hz, C-3), 76.06 (C-6), 73.52 (CH_2^{Bn}), 73.04 (d, $^2J_{4,F}$ = 21.9 Hz, C-4), 72.94 (CH_2^{Bn}), 72.45 (C-5), 72.23 (CH_2^{Bn}), 68.98 (C-7); ^{19}F {^1H} NMR (659 MHz, $CDCl_3$) δ = – 180.53 (s); ^{19}F NMR (659 MHz, $CDCl_3$) δ = – 180.53 (d, $^2J_{F,3}$ = 48.5 Hz); HRMS (+ESI): *m/z* calc. for $C_{28}H_{29}O_4FNa^+$ $[M+Na]^+$: 471.1942, found: 471.1949.

1.1.2.8 4,5,7-Tri-*O*-benzyl-3-deoxy-3-fluoro-D-*glycero*-D-*arabino*-hept-2-ulopyranose (17)

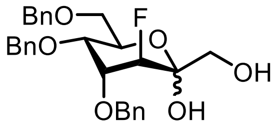

17
$C_{28}H_{31}FO_6$
482.55 g/mol

Compound **17** was synthesized following a protocol by Leshch *et al.*[11] 2,6-Anhydro-4,5,7-tri-*O*-benzyl-1,3-dideoxy-3-fluoro-D-*altro*-hept-1-enitol (**16**, 240 mg, 0.54 mmol, 1 equiv.) was dissolved in a 4:1 (v/v) mixture of acetone/water (2.6 mL), and the reaction mixture was stirred at rt for 18 h after the addition of potassium osmate dihydrate (9.9 mg, 0.03 mmol, 0.05 equiv.) and NMO (125.4 mg, 1.07 mmol, 2 equiv.). After completion, EA (20 mL) was added, and the mixture was washed with water (2 × 15 mL). The organic layer was dried ($MgSO_4$), filtered, and evaporated *in vacuo.* The crude residue was purified by MPLC (10 g silica gel, 12-100% EA in *n*-heptane) yielding 4,5,7-tri-O-benzyl-3-deoxy-3-fluoro-D-*glycero*-D-*arabino*-hept-2-ulopyranose as an anomeric mixture in a 1:0.1 ratio of its α- and β-pyranose form (β-pyranose form only interpretable in ^{19}F NMR) as a colourless oil (**17**, 224 mg, 86%); R_f = 0.28 (*n*-heptane/EA 1:1, CAM); $[\alpha]_D^{20}$ = +41.2 (c = 1.0 in $CHCl_3$); α-Pyranose: ^1H NMR (600 MHz, $CDCl_3$) δ = 7.46-7.18 (m, 15H, H^{Ar}), 5.38 (s, 1H, OH-2), 4.86 (d, $^2J_{H,H}$ = 11.8 Hz, 1H, H^{Bn}), 4.66 (dd, $^2J_{3,F}$ = 45.9 Hz, $^3J_{3,4}$ = 3.8 Hz, 1H, H-3), 4.67 (d, $^2J_{H,H}$ = 10.5 Hz, 1H, H^{Bn}), 4.65 (d, $^2J_{H,H}$ = 10.5 Hz, 1H, H^{Bn}), 4.54 (d, $^2J_{H,H}$ = 11.8 Hz, 1H, H^{Bn}), 4.53 (s, 2H, H^{Bn}), 4.23 (dd, $^3J_{5,4}$ = 3.4 Hz, $^3J_{5,6}$ = 9.7 Hz, 1H, H-5), 4.18 (ddd, $^3J_{4,3}$ = 3.8 Hz, $^3J_{4,5}$ = 3.4 Hz, $^3J_{4,F}$ = 7.0 Hz 1H, H-4), 3.94 (ddd, $^3J_{6,5}$ = 9.7 Hz, $^3J_{6,7b}$ = 9.9 Hz, $^3J_{6,7a}$ = 3.2 Hz, 1H, H-6), 3.84 (dd, $^2J_{7a,7b}$ = 11.0 Hz, $^3J_{7a,6}$ = 3.2 Hz, 1H, H-7a), 3.72 (dd, $^2J_{7b,7a}$ = 11.0 Hz, $^3J_{7b,6}$ = 9.9 Hz, 1H, H-7b), 3.69 (d, $^2J_{1a,1b}$ = 10.8 Hz, 1H, H-1a), 3.54 (dd, $^2J_{1b,1a}$ = 10.7 Hz, $^3J_{1b,OH}$ = 10.5 Hz, 1H, H-1b), 1.93 (d, $^3J_{OH,1b}$ = 10.5 Hz, 1H, OH-1); ^{13}C NMR (151 MHz, $CDCl_3$) δ = 138.27

(C^{Ar}), 137.70 (C^{Ar}), 136.57 (C^{Ar}), 128.72 (2 CH^{Ar}), 128.51 (CH^{Ar}), 128.49 (2 CH^{Ar}), 128.30 (2 CH^{Ar}), 128.14 (2 CH^{Ar}), 127.96 (CH^{Ar}), 127.90 (2 CH^{Ar}), 127.83 (2 CH^{Ar}), 127.55 (CH^{Ar}), 96.10 (d, $^2J_{2,F}$ = 22.14 Hz, C-2), 84.27 (d, $^1J_{3,F}$ = 183.87 Hz, C-3), 74.73 (CH_2^{Bn}), 73.55 (CH_2^{Bn}), 73.43 (d, $^2J_{4,F}$ = 26.0 Hz, C-4), 72.51 (CH_2^{Bn}), 72.09 (C-6), 68.86 (C-7), 68.29 (C-5), 64.29 (d, $^3J_{1,F}$ = 4.0 Hz, C-1); ^{19}F {^{1}H} NMR (659 MHz, $CDCl_3$) δ = – 195.53 (s); ^{19}F NMR (659 MHz, $CDCl_3$) δ = – 195.53 (d, $^2J_{F,3}$ = 45.9 Hz); β-Pyranose: ^{19}F {^{1}H} NMR (659 MHz, $CDCl_3$) δ – 207.00 (s); ^{19}F NMR (659 MHz, $CDCl_3$) δ – 207.00 (m); HRMS (+ESI): *m/z* calc. for $C_{28}H_{31}O_6FNa^+$ $[M+Na]^+$: 505.1997, found: 505.1993.

1.1.2.9 3-Deoxy-3-fluoro-D-sedoheptulose/ 3-Deoxy-3-fluoro-D-*glycero*-D-*arabino*-hept-2-ulopyranose & 3-deoxy-3-fluoro-D-*glycero*-D-*arabino*-hept-2-ulofuranose (3DFS)

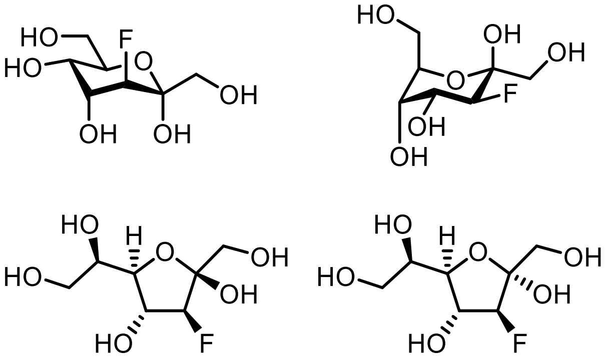

3DFS
$C_7H_{13}FO_6$
212.17 g/mol

4,5,7-Tri-*O*-benzyl-3-deoxy-3-fluoro-D-*glycero*-D-*arabino*-hept-2-ulopyranose (**17**, 175 mg, 0.36 mmol, 1 equiv.) was dissolved in methanol (5 mL) and Pd/C (10% Pd, 85 mg, 0.057 mmol, 0.14 equiv.) was added. The reaction mixture was degassed (3 freezing/thawing cycles with liquid N_2 under vacuum) and stirred at room temperature under 1 atm H_2 pressure for 24 h. The catalyst was removed by filtration over Celite® and the solvent was removed *in vacuo*. The crude residue was purified by reversed-phase chromatography (C_{18}-modified silica gel, MeCN/H_2O 1:3) yielding 3-deoxy-3-fluoro-D-sedoheptulose as a colourless oil as a mixture of its α- and β-furanoses and α- and β-pyranoses in a 2.4:3:4:1 ratio (**3DFS**, 64 mg, 84%); R_f = 0.72 (1-BuOH/acetone/H_2O 6:3:1, CAM); $[\alpha]_D^{20}$ = +26.1 (c = 1.0 in H_2O); β-Furanose: ^{1}H NMR (700 MHz, D_2O) δ = 4.97 (dd, $^2J_{3,F}$ = 52.8 Hz, $^3J_{3,4}$ = 6.5 Hz, 1H, H-3), 4.62 (ddd, $^3J_{4,F}$ = 19.2 Hz, $^3J_{4,3}$ = 6.5 Hz, $^3J_{4,5}$ = 6.5 Hz, 1H, H-4), 3.87 (dddd, $^3J_{6,5}$ = 6.5 Hz, $^3J_{6,7b}$ = 6.5 Hz, $^3J_{6,7a}$ = 3.1 Hz, $^5J_{6,F}$ = 1.1 Hz, 1H, H-6), 3.82 (dd, $^3J_{5,6}$ = 6.5 Hz, $^3J_{5,4}$ = 6.5 Hz, 1H, H-5), 3.78 (dd, $^2J_{7a,7b}$ = 12.0 Hz, $^3J_{7a,6}$ = 3.1 Hz, 1H, H-7a), 3.66 (dd, $^2J_{1a,1b}$ = 12.2 Hz, $^4J_{1a,F}$ = 1.0 Hz, 1H, H-1a), 3.64 (dd, $^2J_{7b,7a}$ = 12.0 Hz, $^3J_{7b,6}$ = 6.5 Hz, 1H, H-7b), 3.62 (d, $^2J_{1b,1a}$ = 12.2 Hz, 1H, H-1b); ^{13}C NMR (176 MHz, D_2O) δ = 103.46 (d, $^2J_{2,F}$ = 17.6 Hz, C-2), 97.95 (d, $^1J_{3,F}$ = 194.0 Hz, C-3), 82.62 (d, $^3J_{5,F}$ = 9.6 Hz, C-5), 76.65 (d, $^2J_{4,F}$ = 22.3 Hz, C-4), 75.16 (C-6), 65.48 (C-1), 64.89 (C-7); ^{19}F {^{1}H} NMR (659 MHz, $CDCl_3$) δ = – 204.94 (s); ^{19}F NMR (659 MHz, $CDCl_3$) δ = – 204.94 (dddd, $^2J_{F,3}$ = 52.8 Hz, $^3J_{F,4}$ = 19.2 Hz, $^5J_{F,6}$ = 1.1 Hz, $^4J_{F,1a}$ = 1.0 Hz); α-Furanose: ^{1}H NMR (700 MHz, D_2O) δ = 4.89 (dd, $^2J_{3,F}$ = 50.8 Hz, $^3J_{3,4}$ = 1.8 Hz, 1H, H-3), 4.43 (ddd, $^3J_{4,F}$ = 23.8 Hz, $^3J_{4,5}$ = 5.2 Hz, $^3J_{4,3}$ = 1.8 Hz, 1H, H-4), 4.10 (dd, $^3J_{5,6}$ = 5.2 Hz, $^3J_{5,4}$ = 5.2 Hz, 1H, H-5), 3.89 (ddd, $^3J_{6,7b}$ = 6.9 Hz, $^3J_{6,5}$ = 5.2, $^3J_{6,7a}$ = 3.7 Hz, 1H, H-6), 3.77 (dd, $^2J_{1a,1b}$ = 12.2 Hz, $^4J_{1a,F}$ = 2.6 Hz, 1H, H-1a), 3.75 (dd, $^2J_{7a,7b}$ = 12.0 Hz, $^3J_{7a,6}$ = 3.7 Hz, 1H, H-7a),

3.68 (dd, $^{2}J_{1b,1a}$ = 12.0 Hz, $^{4}J_{1b,F}$ = 3.5 Hz, 1H, H-1b), 3.63 (dd, $^{2}J_{7b,7a}$ = 12.2 Hz, $^{4}J_{7b,6}$ = 6.9 Hz, 1H, H-7b); ^{13}C NMR (176 MHz, D_2O) δ = 107.12 (d, $^{2}J_{2,F}$ = 26.5 Hz, C-2), 102.77 (d, $^{1}J_{3,F}$ = 184.4 Hz, C-3), 86.07 (d, $^{3}J_{5,F}$ = 3.4 Hz, C-5), 77.54 (d, $^{2}J_{4,F}$ = 27.2 Hz, C-4), 73.91 (C-6), 64.95 (C-7), 64.65 (d, $^{3}J_{1,F}$ = 3.4 Hz, C-1); ^{19}F {^{1}H} NMR (659 MHz, $CDCl_3$) δ = – 193.07 (s); ^{19}F NMR (659 MHz, $CDCl_3$) δ = – 193.07 (dddd, $^{2}J_{F,3}$ = 50.8 Hz, $^{3}J_{F,4}$ = 23.8 Hz, $^{4}J_{F,1b}$ = 3.5 Hz, $^{4}J_{F,1a}$ = 2.6 Hz); β-Pyranose ($^{2}C_{5}$): ^{1}H NMR (700 MHz, D_2O) δ = 4.79 (dd, $^{2}J_{3,F}$ = 49.8 Hz, $^{3}J_{3,4}$ = 9.3 Hz, 1H, H-3), 4.31 (ddd, $^{3}J_{4,F}$ = 11.7 Hz, $^{3}J_{4,3}$ = 9.3 Hz, $^{3}J_{4,5}$ = 3.6 Hz, 1H, H-4), 4.16 (ddd, $^{3}J_{5,4}$ = 3.6 Hz, $^{4}J_{5,F}$ = 3.6 Hz, $^{3}J_{5,6}$ = 2.5 Hz, 1H, H-5), 4.02 (dddd, $^{3}J_{6,7a}$ = 7.2 Hz, $^{3}J_{6,7b}$ = 6.3 Hz, $^{3}J_{6,5}$ = 2.5, $^{5}J_{6,F}$ = 1.1, 1H, H-6), 3.84 (dd, $^{2}J_{7a,7b}$ = 12.0 Hz, $^{3}J_{7a,6}$ = 7.2 Hz, 1H, H-7a), 3.82 (dd, $^{2}J_{7b,7a}$ = 12.0 Hz, $^{4}J_{7b,6}$ = 6.3 Hz, 1H, H-7b), 3.71 (dd, $^{2}J_{1a,1b}$ = 12.0 Hz, $^{4}J_{1a,F}$ = 1.7 Hz, 1H, H-1a), 3.61 (dd, $^{2}J_{1b,1a}$ = 12.0 Hz, $^{4}J_{1b,F}$ = 1.1 Hz, 1H, H-1b); ^{13}C NMR (176 MHz, D_2O) δ = 99.86 (d, $^{2}J_{2,F}$ = 18.5 Hz, C-2), 90.76 (d, $^{1}J_{3,F}$ = 182.2 Hz, C-3), 81.76 (C-6), 71.86 (d, $^{3}J_{5,F}$ = 7.5 Hz, C-5), 68.46 (d, $^{2}J_{4,F}$ = 19.0 Hz, C-4), 66.12 (C-1), 64.82 (C-7); ^{19}F {^{1}H} NMR (659 MHz, $CDCl_3$) δ = – 209.96 (s); ^{19}F NMR (659 MHz, $CDCl_3$) δ = – 209.96 (m); α-Pyranose ($^{5}C_{2}$): ^{1}H NMR (700 MHz, D_2O) δ = 4.74 (dd, $^{2}J_{3,F}$ = 44.9 Hz, $^{3}J_{3,4}$ = 3.8 Hz, 1H, H-3), 4.28 (ddd, $^{3}J_{4,F}$ = 6.4 Hz, $^{3}J_{4,3}$ = 3.8 Hz, $^{3}J_{4,5}$ = 2.5 Hz, 1H, H-4), 4.08 (ddd, $^{3}J_{6,5}$ = 10.4 Hz, $^{3}J_{6,7b}$ = 5.4 Hz, $^{3}J_{6,7a}$ = 2.4, 1H, H-6), 3.88 (dd, $^{2}J_{7a,7b}$ = 12.3 Hz, $^{3}J_{7a,6}$ = 2.4 Hz, 1H, H-7a), 3.84 (ddd, $^{3}J_{5,6}$ = 10.4 Hz, $^{4}J_{5,F}$ = 3.5 Hz, $^{3}J_{5,4}$ = 3.5 Hz, 1H, H-5), 3.80 (dd, $^{2}J_{7b,7a}$ = 12.3 Hz, $^{4}J_{7b,6}$ = 5.4 Hz, 1H, H-7b), 3.72 (dd, $^{2}J_{1a,1b}$ = 11.9 Hz, $^{4}J_{1a,F}$ = 2.5 Hz, 1H, H-1a), 3.53 (dd, $^{2}J_{1b,1a}$ = 11.9 Hz, $^{4}J_{1b,F}$ = 3.9 Hz, 1H, H-1b); ^{13}C NMR (176 MHz, D_2O) δ = 98.65 (d, $^{2}J_{2,F}$ = 24.2 Hz, C-2), 89.60 (d, $^{1}J_{3,F}$ = 176.2 Hz, C-3), 71.28 (C-6), 70.37 (d, $^{2}J_{4,F}$ = 27.8 Hz, C-4), 66.36 ($^{3}J_{1,F}$ = 5.0 Hz, C-1), 66.22 (d, $^{3}J_{5,F}$ = 1.3 Hz, C-5), 63.62 (C-7); ^{19}F {^{1}H} NMR (659 MHz, $CDCl_3$) δ = – 197.98 (s); ^{19}F NMR (659 MHz, $CDCl_3$) δ = – 197.98 (m); HRMS (+ESI): *m/z* calc. for $C_7H_{13}O_6FNa^+$ $[M+Na]^+$: 235.0588, found: 235.0586.

1.1.3 Synthesis of a potential radiolabelling precursor for 4DFS

1.1.3.1 2,7-Anhydro-4,5-*O*-isopropylidene-D-*glycero*-β-D-*arabino*-hept-2-ulopyranose[14] (18)

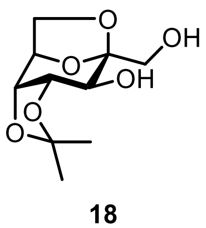

18
$C_{10}H_{16}O_6$
232.23 g/mol

Sedoheptulosan monohydrate (11.293 g, 58.77 mmol, 1 equiv.) and molecular sieves (2.8 g, 3 Å) were suspended in dry acetone (117 mL) and conc. H_2SO_4 (2.1 g, 1.15 mL, 21.45 mmol, 0.37 equiv.). The suspension was stirred under an argon atmosphere at room temperature for 3 h. Over the course of the reaction, the suspension of colourless crystals of sedoheptulosan monohydrate turned into a white, cloudy suspension. The suspension was filtered and washed twice with cold acetone yielding 2,7-anhydro-4,5-*O*-isopropylidene-D-*glycero*-β-D-*arabino*-hept-2-ulopyranose (**18**) as a white solid which was directly used for the next step (11.605 g, 93%); R_f = 0.25 (EA, CAM); $[\alpha]_D^{20}$ = –126.4 (c = 1.0 in H_2O) (lit.,[14] –124 (*c* = 0.88 in H_2O)); ^{1}H NMR (700 MHz, d_6-DMSO) δ = 5.13 (d, $^{3}J_{OH,3}$ = 6.7 Hz, 1H, OH-3), 4.72 (dd, $^{3}J_{OH,1a}$ = 7.0 Hz, $^{3}J_{OH,1b}$ = 5.7 Hz, 1H, OH-1), 4.71 (d, $^{3}J_{6,7b}$ = 5.3 Hz, 1H, H-6), 4.19 (dd, $^{3}J_{5,4}$ = 6.2 Hz, $^{3}J_{5,6}$ = 1.1 Hz, 1H, H-5), 3.99 (t, $^{3}J_{4,5}$ = 6.2 Hz, $^{3}J_{4,3}$ = 6.0 Hz, 1H, H-4), 3.76 (d, $^{2}J_{7a,7b}$ = 7.5 Hz, 1H, H-7a), 3.68 (dd, $^{2}J_{7b,7a}$ = 7.5

Hz, $^{3}J_{7b,6}$ = 5.3 Hz, 1H, H-7b), 3.59 (dd, $^{2}J_{1a,1b}$ = 11.9 Hz, $^{3}J_{1a,OH}$ = 7.0 Hz, 1H, H-1a), 3.53 (dd, $^{3}J_{3,OH}$ = 6.7 Hz, $^{3}J_{3,4}$ =6.0 Hz, 1H, H-3), 3.34 (dd, $^{2}J_{1b,1a}$ = 11.9 Hz, $^{3}J_{1b,OH}$ = 5.7 Hz, 1H, H-1b), 1.39 (s, 3H, CH_3^{isopr}), 1.27 (s, 3H, CH_3^{isopr}); ^{13}C NMR (176 MHz, d_6-DMSO) δ = 109.13 (C^{isopr}), 107.76 (C-2), 78.95 (C-4), 75.86 (C-5), 73.65 (C-6), 71.88 (C-3), 66.64 (C-7), 60.59 (C-1), 27.81 (CH_3^{isopr}), 26.18 (CH_3^{isopr}); HRMS (ESI+): *m/z* calc. for $C_{10}H_{16}O_6Na^+$ [M+Na]$^+$: 255.0839, found: 255.0840.

1.1.3.2 1,3-Di-*O*-acetyl-2,7-anhydro-4,5-*O*-isopropylidene-D-*glycero*-β-D-*arabino*-hept-2-ulopyranose[15] (19)

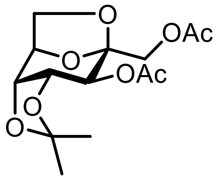

19
$C_{14}H_{20}O_8$
316.31 g/mol

2,7-Anhydro-4,5-*O*-isopropyliden-D-*glycero*-β-D-*arabino*-hept-2-ulopyranose (**18**, 16.312 g, 70.24 mmol, 1 equiv.) was dissolved in dry pyridine (140.5 mL) under an argon atmosphere and cooled to 0°C. Acetic anhydride (28.683 g, 26.6 mL, 281 mmol, 4 equiv.) was then added dropwise followed by 4-(dimethylamino)pyridine (429 mg, 3.51 mmol, 0.05 equiv.). The reaction mixture was stirred at room temperature for 2 h and the solution was then co-evaporated thrice with toluene. The residue was dissolved in EA, washed twice with water and once with brine. The organic layer was dried ($MgSO_4$), filtered and evaporated yielding 1,3-di-*O*-acetyl-2,7-anhydro-4,5-*O*-isopropylidene-D-*glycero*-β-D-*arabino*-hept-2-ulopyranose (**19**) as a white solid which was used for the next reaction without further purification (21.45 g, 97%); R_f = 0.24 (*n*-heptane/EA 2:1, CAM); $[\alpha]_D^{20}$ = –133.5 (c 1.0 in $CHCl_3$) (lit.,[15] –138.2 (*c* = 1.0 in $CHCl_3$)); ^{1}H NMR (700 MHz, $CDCl_3$) δ = 5.13 (d, $^{3}J_{3,4}$ = 5.8 Hz, 1H, H-3), 4.89 (d, $^{3}J_{6,7a}$ = 5.1 Hz, 1H, H-6), 4.40 (d, $^{2}J_{1a,1b}$ = 12.2 Hz, 1H, H-1a), 4.27 (t, $^{3}J_{4,3}$ = $^{3}J_{4,5}$ = 5.8 Hz, 1H, H-4), 4.22 (dd, $^{3}J_{5,4}$ = 5.8 Hz, $^{3}J_{5,6}$ = 1.3 Hz, 1H, H-5), 3.97 (d, $^{2}J_{1b,1a}$ = 12.2 Hz, 1H, H-1b), 3.95 (dd, $^{2}J_{7a,7b}$ = 7.9 Hz, $^{3}J_{7a,6}$ = 5.1 Hz, 1H, H-7a), 3.91 (d, $^{2}J_{7b,7a}$ = 7.9 Hz, 1H, H-7b), 2.12 (s, 3H, CH_3^{Ac-3}), 2.08 (s, 3H, CH_3^{Ac-1}), 1.59 (s, 3H, CH_3^{isopr}), 1.37 (s, 3H, CH_3^{isopr}); ^{13}C NMR (176 MHz, $CDCl_3$) δ = 170.43 (C^{Ac-1}), 169.89 (C^{Ac-3}), 111.52 (C^{isopr}), 105.56 (C-2), 76.90 (C-4), 76.49 (C-5), 75.12 (C-6), 73.43 (C-3), 67.89 (C-7), 61.83 (C-1), 27.80 (CH_3^{Ac-3}), 26.39 (CH_3^{Ac-1}), 21.08 (CH_3^{isopr}), 20.84 (CH_3^{isopr}); HRMS (ESI+): *m/z* calc. for $C_{14}H_{20}O_8Na^+$ [M+Na]$^+$: 339.1050, found: 339.1052.

1.1.3.3 1,3-Di-*O*-acetyl-2,7-anhydro-D-*glycero*-β-D-*arabino*-hept-2-ulopyranose[16] (20)

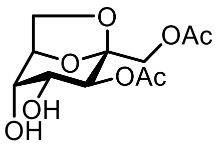

20
$C_{11}H_{16}O_8$
276.24 g/mol

Water (106 mL) was added to a solution 1,3-di-*O*-acetyl-2,7-anhydro-4,5-*O*-isopropylidene-D-*glycero*-β-D-*arabino*-hept-2-ulopyranose (**19**, 21.45 g, 67.81 mmol, 1 equiv.) in glacial acetic acid (424 mL). The reaction mixture was stirred for 2 h at 80°C and the solution was then cooled to room temperature, followed by co-evaporation with toluene (3 times). The crude residue was recrystallized from ethanol yielding 1,3-di-*O*-acetyl-2,7-anhydro-D-*glycero*-β-D-*arabino*-hept-2-ulopyranose (**20**) as a white solid (17.686 g, 94%); R_f = 0.24 (EA, CAM); $[\alpha]_D^{20}$ = –94.7 (c = 1.0 in $CHCl_3$) (lit.,[16] –113.2 (*c* = 1.56 in $CHCl_3$)); ^{1}H NMR (600 MHz, $CDCl_3$) δ = 4.97 (d, $^{3}J_{3,4}$ = 8.3 Hz, 1H, H-3), 4.77 (dd, $^{3}J_{6,7a}$ = 5.4 Hz, $^{3}J_{6,5}$ = 2.0 Hz, 1H, H-6), 4.53

(d, $^2J_{1a,1b}$ = 12.1 Hz, 1H, H-1a), 4.00 (d, $^2J_{1b,1a}$ = 12.1 Hz, 1H, H-1b), 3.96 (td, $^3J_{5,4}$ = $^3J_{5,OH}$ = 4.8 Hz, $^3J_{5,6}$ = 2.0 Hz, 1H, H-5), 3.93 (dd, $^2J_{7a,7b}$ = 8.1 Hz, $^3J_{7a,6}$ = 5.4 Hz, 1H, H-7a), 3.88 (ddd, $^3J_{4,3}$ = 8.3 Hz, $^3J_{4,OH}$ = 6.3 Hz, $^3J_{4,5}$ = 4.8 Hz, 1H, H-4), 3.84 (d, , $^2J_{7b,7a}$ = 8.1 Hz, 1H, H-7b), 3.25 (d, $^3J_{OH,4}$ = 6.3 Hz, 1H, OH-4), 2.77 (d, $^3J_{OH,5}$ = 4.8 Hz, 1H, OH-5), 2.16 (s, 3H, CH_3^{Ac-3}), 2.09 (s, 3H, CH_3^{Ac-1}); ^{13}C NMR (151 MHz, $CDCl_3$) δ = 172.32 (C^{Ac-3}), 170.32 (C^{Ac-1}), 105.50 (C-2), 77.57 (C-6), 74.75 (C-3), 70.53 (C-5), 69.99 (C-4), 66.58 (C-7), 61.70 (C-1), 21.08 (CH_3^{Ac-3}), 20.84 (CH_3^{Ac-3}); HRMS (ESI+): *m/z* calc. for $C_{11}H_{16}O_8Na^+$ $[M+Na]^+$: 299.0737, found: 299.0737.

1.1.3.4 1,3-Di-*O*-acetyl-2,7-anhydro-4,5-*O*-benzylidene-D-*glycero*-β-D-*arabino*-hept-2-ulopyranose[17] (21)

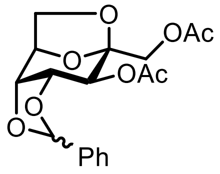

21
$C_{18}H_{20}O_8$
364.35 g/mol

1,3-Di-*O*-acetyl-2,7-anhydro-D-*glycero*-β-D-*arabino*-hept-2-ulopyranose (**20**, 17.686 g, 64.05 mmol, 1 equiv.) was suspended in benzaldehyde (101 mL) under argon. $ZnCl_2$ (10.47 g, 76.86 mmol, 1.2 equiv.), was melted with a Bunsen burner and the resulting solid grinded in a mortar before addition to the reaction mixture. The reaction mixture was stirred at room temperature for 4 h, then diluted with DCM and washed with water and brine. The organic layer was dried ($MgSO_4$), filtered and evaporated. Remaining benzaldehyde was removed under reduced pressure at 80°C for 4 h on a rotary evaporator to yield 1,3-di-*O*-acetyl-2,7-anhydro-4,5-*O*-benzylidene-D-*glycero*-β-D-*arabino*-hept-2-ulopyranose (**21**) as a 1:1 mixture of exo- and endo-stereoisomers as a yellow oil of sufficient purity for the next step (21.12 g, 92%); $[\alpha]_D^{20}$ = –119.8 (c = 1.0 in $CHCl_3$); Exo: R_f = 0.13 (*n*-heptane/EA 2:1, UV & CAM); ^1H NMR (600 MHz, $CDCl_3$) δ = 7.62 (m, 1H, H^{Ar}), 7.50-7.46 (m, 2H, H^{Ar}), 7.39-7.37 (m, 2H, H^{Ar}), 6.23 (s, 1H, H^{Bn}), 5.30 (d, $^3J_{3,4}$ = 6.0 Hz, 1H, H-3), 4.96 (d, $^3J_{6,7a}$ = 4.9 Hz, 1H, H-6), 4.60 (t, $^3J_{4,3}$ = $^3J_{4,5}$ = 6.0 Hz, 1H, H-4), 4.46 (d, $^2J_{1a,1b}$ = 12.1 Hz, 1H, H-1a), 4.26 (dd, $^3J_{5,4}$ = 6.0 Hz, $^3J_{5,6}$ = 1.3 Hz, 1H, H-5), 4.01 (d, $^2J_{1b,1a}$ = 12.1 Hz, 1H, H-1b), 3.95 (dd, $^2J_{7a,7b}$ = 7.9 Hz, $^3J_{7a,6}$ = 4.9 Hz, 1H, H-7a), 3.92 (dd, $^2J_{7b,7a}$ = 7.9 Hz, $^3J_{7b,6}$ = 0.9 Hz, 1H, H-7b), 2.15 (s, 3H, CH_3^{Ac-3}), 2.12 (s, 3H, CH_3^{Ac-1}); ^{13}C NMR (151 MHz, $CDCl_3$) δ = 170.32 (C^{Ac-1}), 169.77 (C^{Ac-3}), 137.39 (C^{Ar}), 133.76 (CH^{Ar}), 128.47 (2 CH^{Ar}), 126.36 (2 CH^{Ar}), 105.19 (C-2), 104.08 (CH^{Bn}), 77.87 (C-4), 76.39 (C-5), 75.60 (C-6), 70.97 (C-3), 67.42 (C-7), 61.69 (C-1), 20.91 (CH_3^{Ac-3}), 20.74 (CH_3^{Ac-1}); Endo: R_f = 0.24 (*n*-heptane/EA 2:1, UV & CAM); ^1H NMR (600 MHz, $CDCl_3$) δ = 7.64-7.61 (m, 1H, H^{Ar}), 7.58-7.55 (m, 2H, H^{Ar}), 7.42-7.39 (m, 2H, H^{Ar}), 5.93 (s, 1H, H^{Bn}), 5.19 (d, $^3J_{3,4}$ = 5.1 Hz, 1H, H-3), 5.02-5.00 (m, 1H, H-6), 4.41 (dd, $^3J_{4,5}$ = 6.8 Hz, $^3J_{4,3}$ = 5.1 Hz, 1H, H-4), 4.39 (d, $^2J_{1a,1b}$ = 12.1 Hz, 1H, H-1a), 4.30 (dd, $^3J_{5,4}$ = 6.8 Hz, $^3J_{5,6}$ = 1.2 Hz, 1H, H-5), 4.02 (d, $^2J_{1b,1a}$ = 12.1 Hz, 1H, H-1b), 3.99-3.98 (m, 2H, H-7a & H-7b), 2.12 (s, 3H, CH_3^{Ac-3}), 2.07 (s, 3H, CH_3^{Ac-1}); ^{13}C NMR (151 MHz, $CDCl_3$) δ = 170.28 (C^{Ac-1}), 169.65 (C^{Ac-3}), 136.31 (C^{Ar}), 129.71 (CH^{Ar}), 128.50 (2 CH^{Ar}), 126.92 (2 CH^{Ar}), 105.50 (CH^{Bn}), 105.47 (C-2), 78.24 (C-5), 76.74 (C-4), 74.86 (C-6), 73.25 (C-3), 67.78 (C-7), 61.75 (C-1), 20.92 (CH_3^{Ac-3}), 20.69 (CH_3^{Ac-1}); HRMS (ESI+): *m/z* calc. for $C_{18}H_{20}O_8Na^+$ $[M+Na]^+$: 387.1050, found: 387.1050.

1.1.3.5 1,3-Di-*O*-acetyl-2,7-anhydro-5-*O*-benzoyl-D-*glycero*-β-D-*arabino*-hept-2-ulopyranose (22)

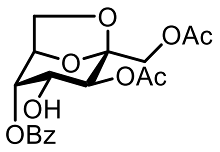

22
$C_{18}H_{20}O_9$
380.35 g/mol

Compound **22** was synthesized following a protocol by Adinolfi *et al.*[18] Sodium bromate (26.2 g, 173.73 mmol, 3 equiv.), dissolved in water (386 mL), was added to 1,3-di-*O*-acetyl-2,7-anhydro-4,5-*O*-benzylidene-D-*glycero*-β-D-*arabino*-hept-2-ulopyranose (**21**, 21.1 g, 57.91 mmol, 1 equiv.) dissolved in EA (772 mL) and sodium dithionite (85% purity, 29.66 g, 144.78 mmol, 2.5 equiv.), dissolved in water (772 mL), was added dropwise to the biphasic system over 10 min. The reaction was stirred at room temperature for 35 min before it was diluted with EA. The layers were separated and the aqueous layer was extracted twice with EA. The combined organic layers were washed with sat. aq. $Na_2S_2O_3$-solution, water and brine, dried ($MgSO_4$), filtered and evaporated. The crude residue was recrystallized from MeOH yielding 1,3-di-*O*-acetyl-2,7-anhydro-5-*O*-benzoyl-D-*glycero*-β-D-*arabino*-hept-2-ulopyranose (**22**) as a white solid (16.68 g, 76%); R_f = 0.18 (*n*-heptane/EA 1:1, UV & CAM); $[\alpha]_D^{20}$ = –177.9 (c = 1.0 in $CHCl_3$); ^1H NMR (600 MHz, $CDCl_3$) δ = 8.10 (d, $^3J_{H,H}$ = 8.4 Hz, 2H, H^{Ar}), 7.63 (t, $^3J_{H,H}$ = 7.4 Hz, 1H, H^{Ar}), 7.50 (t, $^3J_{H,H}$ = 7.8 Hz, 2H, H^{Ar}), 5.35 (dd, $^3J_{5,4}$ = 4.8 Hz, $^3J_{5,6}$ = 2.6 Hz, 1H, H-5), 5.22 (d, $^3J_{3,4}$ = 9.0 Hz, 1H, H-3), 4.93 (dd, $^3J_{6,7a\ or\ 7b}$ = 5.6 Hz, $^3J_{6,5}$ = 2.6 Hz, 1H, H-6), 4.55 (d, $^2J_{1a,1b}$ = 12.1 Hz, 1H, H-1a), 4.16 (m, 1H, H-4), 4.05 (d, $^2J_{1b,1a}$ = 12.1 Hz, 1H, H-1a), 3.98-3.94 (m, 2H, H-7a & H-7b), 2.28 (s, 1H, OH), 2.17 (s, 3H, CH_3^{Ac-3}), 2.11 (s, 3H, CH_3^{Ac-1}); ^{13}C NMR (151 MHz, $CDCl_3$) δ = 171.29 (C^{Ac-3}), 170.34 (C^{Ac-3}), 166.30 (C^{Bz}), 133.88 (CH^{Ar}), 130.10 (2 CH^{Ar}), 129.30 (C^{Ar}), 128.76 (2 CH^{Ar}), 106.01 (C-2), 76.15 (C-6), 74.12 (C-3), 72.88 (C-5), 68.64 (C-4), 66.95 (C-7), 61.60 (C-1), 21.09 (CH_3^{Ac-3}), 20.84 (CH_3^{Ac-1}); HRMS (ESI+): *m/z* calc. for $C_{18}H_{20}O_9Na^+$ $[M+Na]^+$: 403.1000, found: 403.1003.

1.1.3.6 1,3-Di-*O*-acetyl-2,7-anhydro-5-*O*-benzoyl-4-*O*-trifluoromethanesulfonyl-D-*glycero*-β-D-*arabino*-hept-2-ulopyranose (23)

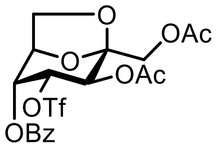

23
$C_{19}H_{19}F_3O_{11}S$
512.41 g/mol

Pyridine (17.677 g, 18 mL, 223.5 mmol, 8.5 equiv.) and trifluoromethanesulfonic anhydride (14.836 g, 8.8 mL, 52.58 mmol, 2 equiv.) were added dropwise to a solution of 1,3-di-*O*-acetyl-2,7-anhydro-5-*O*-benzoyl-D-*glycero*-β-D-*arabino*-hept-2-ulopyranose (**22**, 10 g, 26.29 mmol, 1 equiv.) in dry DCM (131 mL) under an argon atmosphere at 0°C The resulting mixture was stirred at 0°C for 1 h, then diluted with DCM (200 mL) and quenched by the dropwise addition of ice-cold 1 M aq. HCl (125 mL). The layers were separated and the aqueous layer was extracted twice with DCM. The combined organic layers were washed with cold water, cold sat. aq. $NaHCO_3$-solution and cold brine, dried ($MgSO_4$), filtered and evaporated. The crude residue was dried under high vacuum yielding 1,3-di-*O*-acetyl-2,7-anhydro-5-*O*-benzoyl-4-*O*-trifluoromethanesulfonyl-D-*glycero*-β-D-*arabino*-hept-2-ulopyranose (**23**) as a white foam which was used without further purification (13.35 g, 99%); R_f = 0.66 (*n*-heptane/EA 1:1, UV & CAM); $[\alpha]_D^{20}$ = –143.7 (c = 1.0 in $CHCl_3$); ^1H NMR (700 MHz, $CDCl_3$) δ = 8.11 (d, $^3J_{H,H}$ = 8.3 Hz, 2H, H^{Ar}), 7.64 (t, $^3J_{H,H}$ = 7.5 Hz, 1H, H^{Ar}), 7.51 (t, $^3J_{H,H}$ = 7.9 Hz, 2H, H^{Ar}), 5.58 (d, $^3J_{3,4}$ = 9.0 Hz, 1H, H-3), 5.58 (dd,

$^{3}J_{5,4}$ = 4.7 Hz, $^{3}J_{5,6}$ = 2.5 Hz, 1H, H-5), 5.18 (dd, $^{3}J_{4,3}$ = 9.0 Hz, $^{3}J_{4,5}$ = 4.7 Hz, 1H, H-4), 5.00 (dd, $^{3}J_{6,7b}$ = 5.2 Hz, $^{3}J_{6,5}$ = 2.2 Hz, 1H, H-6), 4.56 (d, $^{2}J_{1a,1b}$ = 12.3 Hz, 1H, H-1a), 4.04 (dd, $^{2}J_{7a,7b}$ = 8.5 Hz, $^{3}J_{7a,6}$ = 0.7 Hz, 1H, H-7a), 4.02 (d, $^{2}J_{1b,1a}$ = 12.3 Hz, 1H, H-1b), 4.00 (dd, $^{2}J_{7b,7a}$ = 8.5 Hz, $^{3}J_{7b,6}$ = 5.2 Hz, 1H, H-7b), 2.16 (s, 3H, CH_3^{Ac-3}), 2.11 (s, 3H, CH_3^{Ac-1}); ^{13}C NMR (176 MHz, $CDCl_3$) δ = 170.23 (C^{Ac-1}), 169.42 (C^{Ac-3}), 165.71 (C^{Bz}), 134.16 (CH^{Ar}), 130.25 (2 CH^{Ar}), 128.84 (2 CH^{Ar}), 128.60 (C^{Ar}), 118.39 (q, $^{1}J_{C,F}$ = 319.6 Hz, C^{Tf}), 106.52 (C-2), 81.31 (C-4), 76.20 (C-6), 70.77 (C-5), 70.13 (C-3), 66.88 (C-7), 61.16 (C-1), 20.79 (CH_3^{Ac-1}), 20.60 (CH_3^{Ac-3}). ^{19}F NMR (659 MHz, $CDCl_3$) δ = -75.25 (s); HRMS (ESI+): *m/z* calc. for $C_{19}H_{19}F_3O_{11}SNa^+$ [M+Na]$^+$: 535.0492, found: 535.0490, *m/z* calc. for $C_{18}H_{19}O_8^+$ [M-OTf]$^+$: 363.1074, found: 363.1076.

1.1.3.7 1,4-Di-*O*-acetyl-2,7-anhydro-5-*O*-benzoyl-D-*glycero*-β-D-*lyxo*-hept-2-ulopyranose (24a) & 1,3-di-*O*-acetyl-2,7-anhydro-5-*O*-benzoyl-D-*glycero*-β-D-*lyxo*-hept-2-ulopyranose (24b)

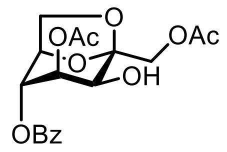

24a
$C_{18}H_{20}O_9$
380.35 g/mol

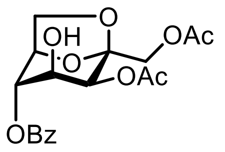

24b
$C_{18}H_{20}O_9$
380.35 g/mol

Crude 1,3-di-*O*-acetyl-2,7-anhydro-5-*O*-benzoyl-4-*O*-trifluoromethanesulfonyl-D-*glycero*-β-D-*arabino*-hept-2-ulopyranose (**23**, 13.35 g, 26.29 mmol, 1 equiv.) was dissolved in dry DMF (263 mL) under an argon atmosphere and sodium nitrite (18.14 g, 262.92 mmol, 10 equiv.) was added. The reaction mixture was stirred at 40°C for 72 h, diluted with EA and washed twice with water. The combined aqueous layers were extracted thrice with EA. Then the combined organic layers were washed with brine, dried ($MgSO_4$), filtered and evaporated. The crude residue was purified in portions of 2 g each by MPLC (340 g silica gel, 12-46% EA in toluene) yielding 1,3-di-*O*-acetyl-2,7-anhydro-5-*O*-benzoyl-D-*glycero*-β-D-*lyxo*-hept-2-ulopyranose (**24b**, 4.51 g, 45%) and 1,4-di-*O*-acetyl-2,7-anhydro-5-*O*-benzoyl-D-*glycero*-β-D-*lyxo*-hept-2-ulopyranose (**24a**, 4.54 g, 45%) as a white foam each; **24b**: R_f = 0.21 (*n*-heptane/EA 1:1, UV & CAM); $[\alpha]_D^{20}$ = –114.9 (c = 1.0 in $CHCl_3$); ^{1}H NMR (600 MHz, $CDCl_3$) δ = 8.09 (d, $^{3}J_{H,H}$ = 7.2 Hz, 2H, H^{Ar}), 7.61 (t, $^{3}J_{H,H}$ = 7.4 Hz, 1H, H^{Ar}), 7.48 (t, $^{3}J_{H,H}$ = 7.8 Hz, 2H, H^{Ar}), 5.21 (t, $^{3}J_{5,4}$ = $^{3}J_{5,6}$ = 1.4 Hz, 1H, H-5), 5.17 (d, $^{3}J_{3,4}$ = 5.2 Hz, 1H, H-3), 4.85 (d, $^{3}J_{6,7b}$ = 5.3 Hz, 1H, H-6), 4.54 (d, $^{2}J_{1a,1b}$ = 12.1 Hz, 1H, H-1a), 4.44 (d, $^{2}J_{7a,7b}$ = 7.5 Hz, 1H, H-7a), 4.33 (tt, $^{3}J_{4,3}$ = $^{3}J_{4,OH}$ = 5.2 Hz, $^{3}J_{4,5}$ = $^{4}J_{4,6}$ = 1.4 Hz, 1H, H-4), 4.09 (d, $^{2}J_{1b,1a}$ = 12.1 Hz, 1H, H-1b), 3.95 (dd, $^{2}J_{7b,7a}$ = 7.5 Hz, $^{3}J_{7b,6}$ = 5.6 Hz, 1H, H-7b), 2.75 (d, $^{3}J_{OH,4}$ = 5.2 Hz, 1H, OH), 2.18 (s, 3H, CH_3^{Ac-3}), 2.11 (s, 3H, CH_3^{Ac-1}); ^{13}C NMR (151 MHz, $CDCl_3$) δ = 170.37 (C^{Ac-1}), 169.58 (C^{Ac-1}), 165.66 (C^{Bz}), 133.75 (CH^{Ar}), 130.13 (2 CH^{Ar}), 129.39 (C^{Ar}), 128.66 (2 CH^{Ar}), 105.82 (C-2), 76.26 (C-6), 74.21 (C-5), 68.75 (C-4), 68.35 (C-3), 66.70 (C-7), 62.05 (C-1), 20.92 ($CH_3^{Ac-1\text{ or }3}$), 20.87 ($CH_3^{Ac-1\text{ or }3}$). HRMS (ESI+): *m/z* calc. for $C_{18}H_{20}O_9Na^+$ [M+Na]$^+$: 403.1000, found: 403.1005; **24a**: R_f = 0.16 (*n*-heptane/EA 1:1, UV & CAM); $[\alpha]_D^{20}$ = –118.9 (c = 1.0 in $CHCl_3$); ^{1}H NMR (600 MHz, $CDCl_3$) δ = 8.07 (d, $^{3}J_{H,H}$ = 8.3 Hz, 2H, H^{Ar}), 7.61 (t, $^{3}J_{H,H}$ = 7.4 Hz, 1H, H^{Ar}), 7.47 (t, $^{3}J_{H,H}$ = 7.8 Hz, 2H, H^{Ar}), 5.37 (dt, $^{3}J_{4,3}$ = 6.0 Hz, $^{3}J_{4,5}$ = $^{4}J_{4,6}$ =1.7 Hz, 1H, H-4), 5.06 (t, $^{3}J_{5,4}$ = $^{3}J_{5,6}$ = 1.7 Hz, 1H, H-5), 4.79 (d, $^{3}J_{6,7b}$ = 5.3 Hz, 1H, H-6), 4.49 (d, $^{2}J_{1a,1b}$ = 12.1 Hz, 1H, H-1a), 4.34 (d, $^{2}J_{1b,1a}$ = 12.1 Hz, 1H, H-1b), 4.21 (dd, $^{2}J_{7a,7b}$ = 7.8 Hz, $^{3}J_{7a,6}$ = 0.7 Hz, 1H, H-7a),

4.11 (dd, $^3J_{3,OH}$ = 10.2 Hz, $^3J_{3,4}$ = 6.0 Hz, 1H, H-3), 3.97 (dd, $^2J_{7b,7a}$ = 7.8 Hz, $^3J_{7b,6}$ = 5.3 Hz, 1H, H-7b), 2.45 (d, $^3J_{OH,3}$ = 10.2 Hz, 1H, OH), 2.20 (s, 3H, CH_3^{Ac-4}), 2.15 (s, 3H, CH_3^{Ac-1}); ^{13}C NMR (151 MHz, $CDCl_3$) δ = 170.70 (C^{Ac-1}), 170.03 (C^{Ac-4}), 165.47 (C^{Bz}), 133.82 (CH^{Ar}), 130.11 (2 CH^{Ar}), 129.24 (C^{Ar}), 128.67 (2 CH^{Ar}), 106.27 (C-2), 75.37 (C-6), 72.76 (C-5), 69.90 (C-4), 66.54 (C-7), 65.94 (C-3), 62.37 (C-1), 21.13 ($CH_3^{Ac-1\text{ or }4}$), 20.99 ($CH_3^{Ac-1\text{ or }4}$); HRMS (ESI+): *m/z* calc. for $C_{18}H_{20}O_9Na^+$ $[M+Na]^+$: 403.1000, found: 403.1002.

1.1.3.8 1,3-Di-*O*-acetyl-2,7-anhydro-5-*O*-benzoyl-D-*glycero*-β-D-*lyxo*-hept-2-ulopyranose (24b) from 24a

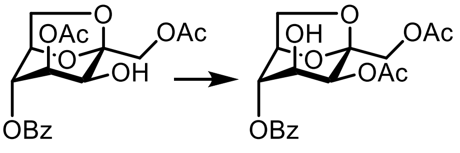

24a $C_{18}H_{20}O_9$ 380.35 g/mol

24b $C_{18}H_{20}O_9$ 380.35 g/mol

1,4-Di-*O*-acetyl-2,7-anhydro-5-*O*-benzoyl-D-*glycero*-β-D-*lyxo*-hept-2-ulopyranose (**24a**, 5.933 g, 15.60 mmol, 1 equiv.) was dissolved in dry DMF (156 mL) and water (2.81 g, 166 mmol, 10 equiv.) was added. The solution was stirred at 40°C for 72 h. After NMR indicated a 1:1 ratio of starting material and product, the solution was co-evaporated thrice with *n*-heptane. The crude residue was purified in portions of 2 g by MPLC (340 g silica gel, 12-46% EA in toluene) yielding 1,3-di-*O*-acetyl-2,7-anhydro-5-*O*-benzoyl-D-*glycero*-β-D-*lyxo*-hept-2-ulopyranose as a white foam (**24b**, 2.65 g, 45%) and 1,4-di-*O*-acetyl-2,7-anhydro-5-*O*-benzoyl-D-*glycero*-β-D-*lyxo*-hept-2-ulopyranose as a white foam (24a, 2.69 g, 45%); Analytical data for **24a** and **24b** were identical as in section 1.1.3.7.

1.1.3.9 1,3-Di-*O*-acetyl-2,7-anhydro-5-*O*-benzoyl-4-*O*-chloroacetyl-D-*glycero*-β-D-*lyxo*-hept-2-ulopyranose (25)

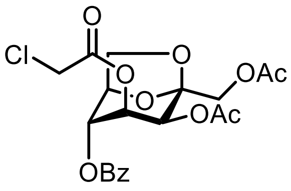

25 $C_{20}H_{21}ClO_{10}$ 456.83 g/mol

Pyridine (3.550 g, 3.62 mL, 44.89 mmol, 4 equiv.) followed by chloroacetic anhydride (3.837 g, 22.44 mmol, 2 equiv.), dissolved in dry DCM (37 mL) were added dropwise to 1,3-di-*O*-acetyl-2,7-anhydro-5-*O*-benzoyl-D-*glycero*-β-D-*arabino*-hept-2-ulopyranose (**24b**, 4.268 g, 11.22 mmol, 1 equiv.) dissolved in dry DCM (75 mL) under an argon atmosphere at 0°C. The reaction mixture was stirred at 0°C for 2.5 h, then water (75 mL) was added at 0°C and stirring was continued for 5 min. The layers were separated and the aqueous layer was extracted twice with DCM. The combined organic layers were washed with 1 M HCl, water and brine, dried ($MgSO_4$), filtered and evaporated to yield 1,3-di-*O*-acetyl-2,7-anhydro-5-*O*-benzoyl-4-*O*-chloroacetyl-D-*glycero*-β-D-*lyxo*-hept-2-ulopyranose (**25**) as a white foam which was used without further purification for the next step (4.843 g, 94%); R_f = 0.37 (*n*-heptane/EA 1:1, UV & CAM); $[\alpha]_D^{20}$ = –149.8 (c = 1.0 in $CHCl_3$); ^{1}H NMR (600 MHz, $CDCl_3$) δ = 8.10 (d, $^3J_{H,H}$ = 7.3 Hz, 2H, H^{Ar}), 7.63 (t, $^3J_{H,H}$ = 7.4 Hz, 1H, H^{Ar}), 7.49 (t, $^3J_{H,H}$ = 7.8 Hz, 2H, H^{Ar}), 5.54 (dt, $^3J_{4,3}$ = 5.4 Hz, $^3J_{4,5}$ = $^4J_{4,6}$ = 1.5 Hz, 1H, H-4), 5.36 (d, $^3J_{3,4}$ = 5.4 Hz, 1H, H-3), 5.13 (t, , $^3J_{5,4}$ = $^3J_{5,6}$ = 1.5 Hz, 1H, H-5), 4.86 (d, $^3J_{6,7b}$ = 5.5 Hz, 1H, H-6), 4.54 (d, $^2J_{1a,1b}$ = 12.1 Hz, 1H, H-1a), 4.39 (d, $^2J_{7a,7b}$ = 7.8 Hz, 1H, H-7a),

4.17 (s, 2H, CH_2^{ClAc}), 4.05 (d, $^2J_{1b,1a}$ = 12.1 Hz, 1H, H-1b), 4.03 (dd, $^2J_{7b,7a}$ = 7.8 Hz, $^3J_{7b,6}$ = 5.5 Hz, 1H, H-7b), 2.10 (s, 3H, CH_3^{Ac-1}), 2.04 (s, 3H, CH_3^{Ac-1}); ^{13}C NMR (151 MHz, $CDCl_3$) δ = 170.28 (C^{Ac-1}), 169.53 (C^{Ac-3}), 166.38 (C^{ClAc}), 165.47 (C^{Bz}), 134.03 (CH^{Ar}), 130.23 (2 CH^{Ar}), 128.88 (C^{Ar}), 128.74 (2 CH^{Ar}), 105.50 (C-2), 75.96 (C-6), 72.59 (C-5), 70.07 (C-4), 66.58 (C-7), 65.91 (C-3), 61.96 (C-1), 40.66 (CH_2^{ClAc}), 20.84 (CH_3^{Ac-1}), 20.63 (CH_3^{Ac-3}); HRMS (ESI+): *m/z* calc. for $C_{20}H_{21}ClO_{10}Na^+$ $[M+Na]^+$: 479.0715, found: 479.0725.

1.1.3.10 1,2,3,7-Tetra-*O*-acetyl-5-*O*-benzoyl-4-*O*-chloroacetyl-D-*glycero*-α-D-*lyxo*-hept-2-ulopyranose (26)

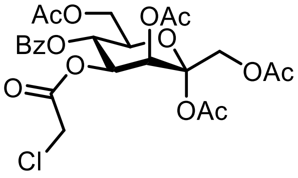

26
$C_{24}H_{27}ClO_{13}$
558.92 g/mol

Compound **26** was synthesized following a protocol by Zottola *et al.*[19] 1,3-Di-*O*-acetyl-2,7-anhydro-5-*O*-benzoyl-4-*O*-chloroacetyl-D-*glycero*-β-D-*lyxo*-hept-2-ulopyranose (**25**, 4.746 g, 10.39 mmol, 1 equiv.) was dissolved in acetic anhydride (104 mL) under an argon atmosphere and cooled to 0°C. Then triethylsilyltrifluoromethanesulfonate (2.746 g, 2.35 mL, 19.39 mmol, 1 equiv.) was added dropwise and the reaction mixture was stirred at 0°C for 1 h. After NMR indicated completion of the reaction, it was quenched by the slow addition of a sat. aq. $NaHCO_3$-sol. (2 L) at 0°C. The mixture was diluted with DCM and the layers were separated. The aqueous layer was extracted twice with DCM. The combined organic layers were washed with sat. aq. $NaHCO_3$-sol. and brine, dried ($MgSO_4$), filtered and evaporated. The crude residue was purified by MPLC (100 g silica gel, 12-63% EA in *n*-heptane) yielding 1,2,3,7-tetra-*O*-acetyl-5-*O*-benzoyl-4-*O*-chloroacetyl-D-*glycero*-α-D-*lyxo*-hept-2-ulopyranose (**26**) as a white foam (5.643 g, 97%); R_f = 0.36 (*n*-heptane/EA 1:1, UV & CAM); $[\alpha]_D^{20}$ = +23.4 (c = 1.0 in $CHCl_3$); ^{1}H NMR (700 MHz, $CDCl_3$) δ = 7.99 (d, $^3J_{H,H}$ = 8.1 Hz, 2H, H^{Ar}), 7.60 (t, $^3J_{H,H}$ = 7.4 Hz, 1H, H^{Ar}), 7.46 (t, $^3J_{H,H}$ = 7.8 Hz, 2H, H^{Ar}), 5.59 (dd, $^3J_{4,5}$ = 10.5 Hz, $^3J_{4,3}$ = 2.6 Hz, 1H, H-4), 5.57 (t, $^3J_{5,4}$ = $^3J_{5,6}$ = 10.5 Hz, 1H, H-5), 5.52 (d, $^3J_{3,4}$ = 2.6 Hz, 1H, H-3), 4.88 (d, $^2J_{1a,1b}$ = 12.3 Hz, 1H, H-1a), 4.49 (d, $^2J_{1b,1a}$ = 12.3 Hz, 1H, H-1b), 4.29 (dd, $^2J_{7a,7b}$ = 12.3 Hz, $^3J_{7a,6}$ = 5.7 Hz, 1H, H-7a), 4.21 (dd, $^2J_{7b,7a}$ = 12.3 Hz, $^3J_{7b,6}$ = 3.0 Hz, 1H, H-7b), 4.10 (ddd, $^3J_{6,5}$ = 9.7 Hz, $^3J_{6,7a}$ = 5.7 Hz, $^3J_{6,7b}$ = 3.0 Hz, 1H, H-6), 3.90 (d, $^2J_{H,H}$ = 15.2 Hz, 1H, H^{ClAc}), 3.87 (d, $^2J_{H,H}$ = 15.2 Hz, 1H, H^{ClAc}), 2.21 (s, 3H, CH_3^{Ac-2}), 2.19 (s, 3H, $CH_3^{Ac-1\ or\ 3}$), 2.04 (s, 3H, $CH_3^{Ac-1\ or\ 3}$), 2.02 (s, 3H, CH_3^{Ac-7}); ^{13}C NMR (176 MHz, $CDCl_3$) δ = 170.74 (C^{Ac-7}), 169.97 ($C^{Ac-1\ or\ 3}$), 169.90 ($C^{Ac-1\ or\ 3}$), 167.69 (C^{Ac-2}), 166.73 (C^{ClAc}), 165.47 (C^{Bz}), 134.01 (CH^{Ar}), 130.02 (2 CH^{Ar}), 128.80 (2 CH^{Ar}), 128.72 (C^{Ar}), 102.05 (C-2), 71.23 (C-4), 71.03 (C-6), 67.12 (C-3), 66.06 (C-5), 62.42 (C-7), 60.15 (C-1), 40.46 (CH_2^{ClAc}), 22.04 (CH_3^{Ac-2}), 20.86 ($CH_3^{Ac-1,3\ or\ 7}$), 20.76 ($CH_3^{Ac-1,3\ or\ 7}$), 20.70 ($CH_3^{Ac-1,3\ or\ 7}$); HRMS (ESI+): *m/z* calc. for $C_{24}H_{27}ClO_{13}Na^+$ $[M+Na]^+$: 581.1032, found: 581.1017.

1.1.3.11 1,2,3,7-Tetra-*O*-acetyl-5-*O*-benzoyl-D-*glycero*-α-D-*lyxo*-hept-2-ulopyranose (27)

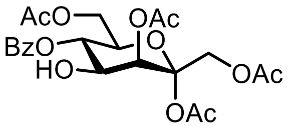

27
$C_{22}H_{26}O_{12}$
482.44 g/mol

Compound **27** was synthesized following a protocol by Van Boeckel *et al.*[20] Hydrazine hydrate (1.7 g, 1.65 mL, 33.96 mmol, 1 equiv.) was dissolved in a 2:1 (v/v) mixture of ethanol and water (68 mL) and cooled to 0°C. Then, *N,N*-diisopropyl-*N*-ethylamine (4.39 g, 5.93 mL, 33.96 mmol, 1 equiv.) was slowly added followed by carbon disulfide (2.59 g, 2.05 mL, 33.96 mmol, 1 equiv.) in dioxane (13.6 mL). The mixture was stirred at 0°C for 10 min and immediately used in the following reaction.
1,2,3,7-Tetra-*O*-acetyl-5-*O*-benzoyl-4-*O*-chloroacetyl-D-*glycero*-α-D-*lyxo*-hept-2-ulo-pyranose (**26**, 5.643 g, 10.10 mmol, 1 equiv.) was dissolved in a 3:1 (v/v) mixture of 2,6-lutidine and acetic acid (101 mL) and cooled to 0°C. The freshly prepared hydrazine dithiocarbonate solution (0.375 M, 81 mL, 30.29 mmol, 3 equiv.) was added dropwise and the reaction stirred at 0°C for 20 min before a sat. aq. $NaHCO_3$-sol. (250 mL) was added. The mixture was extracted thrice with DCM. The combined organic layers were washed with a sat. aq. $NaHCO_3$-sol., 0.5 M HCl, a sat. aq. $NaHCO_3$-sol. and brine. The organic layer was dried ($MgSO_4$), filtered and evaporated. The crude residue was purified by MPLC (340 g silica gel, 12-83% EA in *n*-heptane) yielding 1,2,3,7-tetra-*O*-acetyl-5-*O*-benzoyl-D-*glycero*-α-D-*lyxo*-hept-2-ulopyranose (**27**) as a white solid (3.685 g, 76%); R_f = 0.23 (*n*-heptane/EA 1:1, UV & CAM); $[\alpha]_D^{20}$ = +44.0 (c = 1.0 in $CHCl_3$); ^1H NMR (600 MHz, $CDCl_3$) δ = 8.05 (d, $^3J_{H,H}$ = 7.3 Hz, 2H, H^{Ar}), 7.61 (t, $^3J_{H,H}$ = 7.4 Hz, 1H, H^{Ar}), 7.47 (t, $^3J_{H,H}$ = 7.8 Hz, 2H, H^{Ar}), 5.53 (d, $^3J_{3,4}$ = 3.6 Hz, 1H, H-3), 5.31 (t, $^3J_{5,4}$ = $^3J_{5,6}$ = 10.0 Hz, 1H, H-5), 4.82 (d, $^2J_{1a,1b}$ = 12.2 Hz, 1H, H-1a), 4.46 (d, $^2J_{1b,1a}$ = 12.2 Hz, 1H, H-1b), 4.31 (ddd, $^3J_{4,5}$ = 10.0 Hz, $^3J_{4,OH}$ = 5.3 Hz, $^3J_{4,3}$ = 3.6 Hz, 1H, H-4), 4.29-4.25 (m, 2H, H-7a & H-7b), 4.07 (ddd, $^3J_{6,5}$ = 10.0 Hz, $^3J_{6,7a}$ = 4.7 Hz, $^3J_{6,7b}$ = 3.8 Hz 1H, H-6), 2.48 (d, $^3J_{OH,4}$ = 5.3 Hz, 1H, OH), 2.20 (s, 3H, CH_3^{Ac-3}), 2.16 (s, 3H, CH_3^{Ac-2}), 2.06 (s, 3H, CH_3^{Ac-1}), 2.03 (s, 3H, CH_3^{Ac-7}); ^{13}C NMR (151 MHz, $CDCl_3$) δ = 170.85 (C^{Ac-7}), 170.31 (C^{Ac-3}), 170.08 (C^{Ac-1}), 167.78 (C^{Ac-2}), 166.92 (C^{Bz}), 133.97 (CH^{Ar}), 130.08 (2 CH^{Ar}), 129.03 (C^{Ar}), 128.74 (2 CH^{Ar}), 102.31 (C-2), 70.77 (C-6), 69.86 (C-5), 69.36 (C-3 or C-4), 69.35 (C-3 or C-4), 62.65 (C-7), 60.54 (C-1), 21.99 (CH_3^{Ac-2}), 20.96 ($CH_3^{Ac-1,3\ or\ 7}$), 20.82 ($CH_3^{Ac-1,3\ or\ 7}$), 20.75 ($CH_3^{Ac-1,3\ or\ 7}$); HRMS (ESI+): *m/z* calc. for $C_{22}H_{26}O_{12}Na^+$ $[M+Na]^+$: 505.1316, found: 505.1297.

1.1.3.12 1,2,3,7-Tetra-*O*-acetyl-5-*O*-benzoyl-4-O-trifluoromethanesulfonyl-D-*glycero*-α-D-*lyxo*-hept-2-ulopyranose (28a)

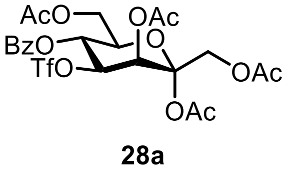

28a
$C_{23}H_{25}F_3O_{14}S$
614.49 g/mol

1,2,3,7-Tetra-*O*-acetyl-5-*O*-benzoyl-D-*glycero*-α-D-*lyxo*-hept-2-ulopyranose (**27**, 300 mg, 0.62 mmol, 1 equiv.) was dissolved in dry DCM (3.1 mL) under an argon atmosphere and cooled to 0°C. Dry pyridine (418 mg, 427 µL, 5.29 mmol, 8.5 equiv.) and trifluoromethanesulfonic anhydride (351 mg, 209 µL, 1.24 mmol, 2 equiv.) were added dropwise, the reaction mixture was stirred at 0°C for 1 h before addition of DCM (3 mL). Then an ice-cold 1 M aq. HCl (5 mL) solution was added dropwise, the layers were separated and the

aqueous layer was extracted twice with DCM. The combined organic layers were washed with cold water, cold sat. aq. $NaHCO_3$-solution and cold brine. The organic layer was dried ($MgSO_4$), filtered and evaporated (25°C). The crude residue was purified by MPLC (25 g silica gel, 8-66% EA in *n*-heptane) to give a colourless oil which was co-evaporated thrice with Et_2O to yield 1,2,3,7-tetra-*O*-acetyl-5-*O*-benzoyl-4-*O*-trifluoromethanesulfonyl-D-*glycero*-α-D-*lyxo*-hept-2-ulopyranose (**28a**) as a white foam (320 mg, 84%); R_f = 0.37 (*n*-heptane/EA 1:1, CAM); $[\alpha]_D^{20}$ = +0.62 (c = 1.0 in $CHCl_3$); ^{1}H NMR (600 MHz, $CDCl_3$) δ = 8.06 (d, $^3J_{H,H}$ = 8.1 Hz, 2H, H^{Ar}), 7.62 (t, $^3J_{H,H}$ = 7.5 Hz, 1H, H^{Ar}), 7.47 (t, $^3J_{H,H}$ = 7.9 Hz, 2H, H^{Ar}), 5.74 (d, $^3J_{3,4}$ = 3.5 Hz, 1H, H-3), 5.69 (t, $^3J_{5,4}$ = $^3J_{5,6}$ = 10.1 Hz, 1H, H-5), 5.35 (dd, $^3J_{4,5}$ = 10.1 Hz, $^3J_{4,3}$ = 3.5 Hz, 1H, H-4), 4.86 (d, $^2J_{1a,1b}$ = 12.4 Hz, 1H, H-1a), 4.45 (d, $^2J_{1b,1a}$ = 12.4 Hz, 1H, H-1b), 4.28 (dd, $^2J_{7a,7b}$ = 12.3 Hz, $^3J_{7a,6}$ = 5.5 Hz, 1H, H-7a), 4.20 (dd, $^2J_{7b,7a}$ = 12.3 Hz, $^3J_{7b,6}$ = 3.2 Hz, 1H, H-7b), 4.08 (ddd, $^3J_{6,5}$ = 10.1 Hz, $^3J_{6,7a}$ = 5.5 Hz, $^3J_{6,7b}$ = 3.2 Hz, 1H, H-6), 2.22 (s, 3H, CH_3^{Ac-3}), 2.22 (s, 3H, CH_3^{Ac-2}), 2.07 (s, 3H, CH_3^{Ac-1}), 2.00 (s, 3H, CH_3^{Ac-7}); ^{13}C NMR (151 MHz, $CDCl_3$) δ = 170.64 (C^{Ac-7}), 169.96 (C^{Ac-1}), 168.84 (C^{Ac-3}), 167.43 (C^{Ac-2}), 165.11 (C^{Bz}), 134.20 (CH^{Ar}), 130.16 (2 CH^{Ar}), 128.78 (2 CH^{Ar}), 128.25 (C^{Ar}), 118.31 (q, $^1J_{C,F}$ = 319.6 Hz, C^{Tf}), 102.31 (C-2), 82.17 (C-4), 70.96 (C-6), 67.33 (C-3), 65.89 (C-5), 62.20 (C-7), 59.88 (C-1), 22.02 (CH_3^{Ac-2}), 20.71 ($CH_3^{Ac-1,3\text{ or }7}$), 20.69 ($CH_3^{Ac-1,3\text{ or }7}$), 20.60 ($CH_3^{Ac-1,3\text{ or }7}$); ^{19}F NMR (659 MHz, $CDCl_3$) δ = −75.30 (s); HRMS (ESI+): *m/z* calc. for $C_{23}H_{25}F_3O_{14}SNa^+$ [M+Na]$^+$: 637.0809, found: 637.0794.

1.1.3.13 1,2,3,7-Tetra-*O*-acetyl-5-*O*-benzoyl-4-*O*-4-nitrobenzenesulfonyl-D-*glycero*-α-D-*lxyo*-hept-2-ulopyranose (28b)

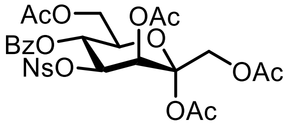

28b

$C_{28}H_{29}NO_{16}S$

667.59 g/Mol

Compound **28b** was synthesized following a protocol by Chiotellis *et al.*[21] 1,2,3,7-Tetra-*O*-acetyl-5-*O*-benzoyl-D-*glycero*-α-D-*lyxo*-hept-2-ulopyranose (**27**, 100 mg, 0.21 mmol, 1 equiv.) was dissolved in dry DCM (2 mL) under an argon atmosphere and cooled to 0°C. DMAP (3 mg, 0.02 mmol, 0.1 equiv.) was added followed by triethylamine (59 mg, 81 µL, 0.58 mmol, 2.8 equiv.) and 4-nitrobenzenesulfonyl chloride (92 mg, 0.41 mmol, 2 equiv.), dissolved in dry DCM (1 mL). The ice bath was removed and stirring was continued for 2 h at room temperature. The reaction mixture was then cooled to 0°C again before dropwise addition of a 5% aq. $NaHCO_3$-solution (3 mL). The layers were separated and the aqueous layer was extracted twice with DCM. The combined organic layers were dried ($MgSO_4$), filtered and evaporated. The crude residue was purified by MPLC (25 g silica gel, 12-95% EA in *n*-heptane) yielding 1,2,3,7-tetra-*O*-acetyl-5-*O*-benzoyl-4-*O*-4-nitrobenzenesulfonyl-D-*glycero*-α-D-*lyxo*-hept-2-ulopyranose as a white solid (**28b**, 102 mg, 74%); R_f = 0.33 (*n*-heptane/EA 1:1, UV & CAM); $[\alpha]_D^{20}$ = –32.5 (c = 1.0 in $CHCl_3$); ^{1}H NMR (600 MHz, $CDCl_3$) δ = 8.01 (d, $^3J_{H,H}$ = 8.8 Hz, 2H, H^{Ar}), 7.90 (d, $^2J_{H,H}$ = 8.8 Hz, 2H, H^{Ar}), 7.78 (d, $^3J_{H,H}$ = 7.2 Hz, 2H, H^{Ar}), 7.56 (t, $^3J_{H,H}$ = 7.5 Hz, 1H, H^{Ar}), 7.37 (t, $^3J_{H,H}$ = 7.8 Hz, 2H, H^{Ar}), 5.72 (d, $^3J_{3,4}$ = 3.5 Hz, 1H, H-3), 5.53 (t, $^3J_{5,4}$ = $^3J_{5,6}$ = 10.1 Hz, 1H, H-5), 5.23 (dd, $^3J_{4,5}$ = 10.1 Hz, $^3J_{4,3}$ = 3.5 Hz, 1H, H-4), 4.84 (d, $^2J_{1a,1b}$ = 12.4 Hz, 1H, H-1a), 4.44 (d, $^2J_{1b,1a}$ = 12.4 Hz, 1H, H-1b), 4.21 (dd, $^2J_{7a,7b}$ = 12.3 Hz, $^3J_{7a,6}$ = 5.6 Hz, 1H, H-7a), 4.14 (dd, $^2J_{7b,7a}$ = 12.3 Hz, $^3J_{7b,6}$

= 3.1 Hz, 1H, H-7b), 4.05 (ddd, $^3J_{6,5}$ = 9.8 Hz, $^3J_{6,7a}$ = 5.6 Hz, $^3J_{6,7b}$ = 3.1 Hz, 1H, H-6), 2.24 (s, 3H, $CH_3^{Ac\text{-}2}$), 2.17 (s, 3H, $CH_3^{Ac\text{-}3}$), 2.07 (s, 3H, $CH_3^{Ac\text{-}1}$), 1.97 (s, 3H, $CH_3^{Ac\text{-}7}$); ^{13}C NMR (151 MHz, $CDCl_3$) δ = 170.64 ($C^{Ac\text{-}7}$), 170.01 ($C^{Ac\text{-}1}$), 168.89 ($C^{Ac\text{-}3}$), 167.56 ($C^{Ac\text{-}2}$), 164.74 (C^{Bz}), 150.49 (C^{Ar}), 141.75 (C^{Ar}), 134.30 (CH^{Ar}), 129.82 (2 CH^{Ar}), 128.94 (2 CH^{Ar}), 128.64 (2 CH^{Ar}), 128.17 (C^{Ar}), 124.34 (2 CH^{Ar}), 102.35 (C-2), 77.58 (C-4), 70.81 (C-6), 67.93 (C-3), 65.82 (C-5), 62.22 (C-7), 59.99 (C-1), 22.11 ($CH_3^{Ac\text{-}2}$), 20.74 ($CH_3^{Ac\text{-}1,3 \text{ or } 7}$), 20.71 ($CH_3^{Ac\text{-}1,3 \text{ or } 7}$), 20.68 ($CH_3^{Ac\text{-}1,3 \text{ or } 7}$); HRMS (ESI+): *m/z* calc. for $C_{28}H_{29}NO_{16}SNa^+$ $[M+Na]^+$: 690.1099, found: 690.1097.

1.1.4 Synthesis of a potential radiolabelling precursor for 3DFS

1.1.4.1 2,7-Anhydro-4,5-*O*-isopropylidene-1-*O*-trityl-D-*glycero*-β-D-*arabino*-hept-2-ulopyranose[15] (S3)

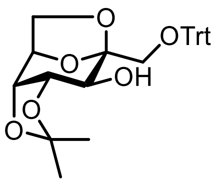

S3
$C_{29}H_{30}O_6$
474.55 g/mol

2,7-Anhydro-4,5-*O*-isopropylidene-D-*glycero*-β-D-*arabino*-hept-2-ulopyranose (**18**, 600 mg, 2.58 mmol, 1 equiv.) was dissolved in pyridine (3 mL). Trityl chloride (1.29 g, 4.65 mmol, 1.8 equiv.) was added, the reaction mixture was heated to 70°C and stirred for 2 h. The reaction mixture was cooled to room temperature and stirring was continued for 18 h. Then the solvent was removed *in vacuo* and the residue was co-evaporated with toluene (10 mL). The remaining viscous liquid was taken up in EA (20 mL) and washed with water (3 × 20 mL). The organic layer was dried ($MgSO_4$) and evaporated under reduced pressure. The crude residue was purified by MPLC (100 g silica gel, 12-100% EA in *n*-heptane) to give a colourless solid (**S3**, 1.04 g, 85%); R_f = 0.52 (*n*-heptane/EA 1:1, CAM); $[\alpha]_D^{20}$ = – 60.89 (c = 1.0 in $CHCl_3$) (lit.,[15] – 44.5 (*c* = 1.2 in $CHCl_3$)); ^{1}H NMR (600 MHz, d_6-DMSO) δ = 7.43 – 7.24 (m, 15H, H^{Ar}), 5.27 (d, $^3J_{OH3,3}$ = 7.3 Hz, 1H, OH-3), 4.82 (d, $^3J_{6,7b}$ = 5.4 Hz, 1H, H-6), 4.24 (dd, $^3J_{5,4}$ = 6.0 Hz, $^3J_{5,6}$ = 0.9 Hz, 1H, H-5), 4.03 (dd, $^3J_{4,3}$ = 5.9 Hz, $^3J_{4,5}$ = 6.0 Hz, 1H, H-4), 3.81 (d, $^2J_{7a,7b}$ = 7.5 Hz, 1H, H-7a), 3.73 (dd, $^2J_{7b,7a}$ = 7.5 Hz, $^3J_{7b,6}$ = 5.4 Hz, 1H, H-7b), 3.68 (dd, $^3J_{3,OH3}$ = 7.3 Hz, $^3J_{3,4}$ = 5.9 Hz, 1H, H-3), 3.15 (d, $^2J_{1a,1b}$ = 9.5 Hz, 1H, H-1a), 3.00 (d, $^2J_{1b,1a}$ = 9.5 Hz, 1H, H-1b), 1.47 (s, 3H, CH_3^{isopr}), 1.30 (s, 3H, CH_3^{isopr}); ^{13}C NMR (151 MHz, d_6-DMSO) δ = 143.58 (3 × C^{Trt}), 128.27 (6 × CH^{Trt}), 127.80 (6 × CH^{Trt}), 126.98 (3 × CH^{Trt}), 109.35 (C^{isopr}), 107.26 (C-2), 85.68 (C^{Trt}), 79.13 (C-4), 75.83 (C-5), 73.80 (C-6), 72.63 (C-3), 66.61 (C-7), 62.30 (C-1), 27.85 (CH_3^{isopr}), 26.31 (CH_3^{isopr}); HRMS (+ESI): *m/z* calc. for $C_{29}H_{30}O_6Na^+$ $[M+Na]^+$: 497.1935, found: 497.1922.

1.1.4.2 2,7-Anhydro-4,5-*O*-isopropylidene-1-*O*-trityl-D-*glycero*-β-D-*ribo*-2,3-heptodiulopyranose (S4)

2,7-Anhydro-4,5-*O*-isopropyliden-1-*O*-trityl-D-*glycero*-β-D-*arabino*-hept-2-ulopyranose (**S3**, 500 mg, 1.05 mmol, 1 equiv.) was dissolved in DCM (8.5 mL) and cooled to 0°C. DMP (668 mg, 1.57 mmol, 1.5 equiv.) was added and stirring was continued at room temperature for 3 h. The reaction mixture was diluted with DCM (15 mL) and washed thrice with a 1:1 (v/v) mixture of sat. aqueous $NaHCO_3$ and sat. aqueous $Na_2S_2O_3$-sol.

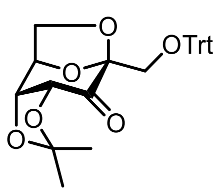

S4

$C_{29}H_{28}O_6$
472.54 g/mol

(15 mL). After drying ($MgSO_4$), the solvent was evaporated and the crude residue was purified by MPLC (50 g silica gel, 24-100% EA in *n*-heptane) to yield 2,7-anhydro-4,5-*O*-isopropylidene-1-*O*-trityl-D-*glycero*-β-D-*xylulo*-hept-2-ulopyranose (**S4**) as a colourless solid (397 mg, 80%); R_f = 0.63 (*n*-heptane/EA 1:1, CAM); $[\alpha]_D^{20}$ = – 62.19 (c = 1.0 in $CHCl_3$), (lit.,[15] – 68.0 (c = 1.0 in $CHCl_3$)); ^{1}H NMR (600 MHz, $CDCl_3$) δ = 7.50- 7.21 (m, 15H, H^{Ar}), 5.01 (d, $^3J_{6,7a}$ = 4.8 Hz, 1H, H-6), 4.63 (d, $^3J_{4,5}$ = 6.3 Hz, 1H, H-4), 4.58 (dd, $^3J_{5,4}$ = 6.3 Hz, $^3J_{5,6}$ = 1.3 Hz, 1H, H-5), 4.15 (dd, $^2J_{7a,7b}$ = 7.8 Hz, $^3J_{7a,6}$ = 4.8 Hz, 1H, H-7a), 4.06 (d, $^2J_{7b,7a}$ = 7.8 Hz, 1H, H-7b), 3.52 (d, $^2J_{1a,1b}$ = 11.0 Hz, 1H, H-1a), 3.52 (d, $^2J_{1b,1a}$ = 11.0 Hz, 1H, H-1b), 1.43 (s, 3H, $CH_3{}^{isopr}$), 1.41 (s, 3H, $CH_3{}^{isopr}$); ^{13}C NMR (151 MHz, $CDCl_3$) δ = 196.79 (C-3), 143.59 (3 × C^{Trt}), 128.78 (6 × CH^{Trt}), 127.82 (6 × CH^{Trt}), 127.03 (3 × CH^{Trt}), 111.61 (C^{isopr}), 105.16 (C-2), 86.72 (C^{Trt}), 79.34 (C-5), 75.83 (C-4), 74.75 (C-6), 66.65 (C-7), 59.74 (C-1), 27.16 ($CH_3{}^{isopr}$), 26.17 ($CH_3{}^{isopr}$); HRMS (+ESI): *m/z* calc. for $C_{29}H_{28}O_6Na^+$ $[M+Na]^+$: 495.1778, found: 495.1762.

1.1.4.3 2,7-Anhydro-4,5-*O*-isopropylidene-1-*O*-trityl-D-*glycero*-β-D-*ribo*-hept-2-ulopyranose[15] (S5)

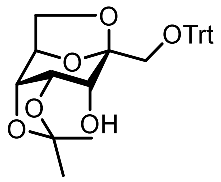

S5

$C_{29}H_{30}O_6$
474.55 g/mol

2,7-Anhydro-4,5-*O*-isopropylidene-1-*O*-trityl-D-*glycero*-β-D-*xylulo*-hept-2-ulopyranose (**S4**, 760 mg, 1.61 mmol, 1 equiv.) was dissolved in EtOH (70 mL) and cooled to 0°C. $NaBH_4$ (730 mg, 19.30 mmol, 12 equiv.) and water (5.08 mL) were added and the mixture was allowed to warm up to room temperature. After stirring for 18 h, EtOH was removed *in vacuo*, the remaining liquid was diluted with water (20 mL) and extracted with EA (3 × 20 mL). The combined organic layers were dried ($MgSO_4$) and the solvent was evaporated under reduced pressure. The crude residue was purified by MPLC (50 g silica gel, 12-100% EA in *n*-heptane) to yield 2,7-anhydro-4,5-*O*-isopropylidene-1-*O*-trityl-D-*glycero*-β-D-*ribo*-hept-2-ulopyranose (**S5**) as a colourless solid (700 mg, 91%); R_f = 0.63 (*n*-heptane/EA 1:1, CAM); $[\alpha]_D^{20}$ = – 44.23 (c = 0.76 in $CHCl_3$), (lit.,[15] – 50.2 (c = 0.85 in $CHCl_3$)); ^{1}H NMR (600 MHz, d_6-DMSO) δ = 7.43-7.24 (m, 15H, H^{Ar}), 4.77 (d, $^3J_{6,7b}$ = 5.0 Hz, 1H, H-6), 4.35 (d, $^3J_{OH,3}$ = 5.2 Hz, 1H, OH-3), 4.23 (dd, $^3J_{4,3}$ = 11.8 Hz, $^3J_{4,5}$ = 6.0 Hz, 1H, H-4), 4.12 (dd, $^3J_{5,4}$ = 6.0 Hz, $^3J_{5,6}$ = 1.1 Hz, 1H, H-5), 3.89 (d, $^2J_{7a,7b}$ = 7.5 Hz, 1H, H-7a), 3.70 (dd, $^2J_{7b,7a}$ = 7.5 Hz, $^3J_{7b,6}$ = 5.0 Hz, 1H, H-7b), 3.65 (dd, $^3J_{3,4}$ = 11.8 Hz, $^3J_{3,OH3}$ = 5.2 Hz, 1H, H-3), 3.20 (d, $^2J_{1a,1b}$ = 9.4 Hz, 1H, H-1a), 3.04 (d, $^2J_{1b,1a}$ = 9.4 Hz, 1H, H-1b), 1.42 (s, 3H, $CH_3{}^{isopr}$), 1.29 (s, 3H, $CH_3{}^{isopr}$); ^{13}C NMR (151 MHz, d_6-DMSO) δ = 143.63 (3 × C^{Trt}), 128.30 (6 × CH^{Trt}), 127.80 (6 × CH^{Trt}), 126.97 (3 × CH^{Trt}), 109.44 (C^{isopr}), 107.08 (C-2), 85.63 (C^{Trt}), 73.13 (C-5), 72.77 (C-6), 70.49 (C-4), 66.12 (C-7), 64.38 (C-3), 63.50 (C-1), 25.95 ($CH_3{}^{isopr}$), 25.73 ($CH_3{}^{isopr}$); HRMS (+ESI): *m/z* calc. for $C_{29}H_{30}O_6Na^+$ $[M+Na]^+$: 497.1935, found: 497.1925.

1.1.4.4 2,7-Anhydro-3-*O*-benzyl-4,5-*O*-isopropylidene-1-*O*-trityl-D-*glycero*-β-D-*ribo*-hept-2-ulopyranose (S6)

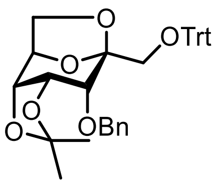

S6
$C_{36}H_{36}O_6$
564.68 g/mol

Sodium hydride (101 mg, 4.21 mmol, 4 equiv.) was suspended in dry THF (3 mL) under argon atmosphere and cooled to 0°C. A solution of the 2,7-anhydro-4,5-*O*-isopropylidene-1-*O*-trityl-D-*glycero*-β-D-*ribo*-hept-2-ulopyranose (**S5**, 500 mg, 1.05 mmol, 1 equiv.) in dry THF (4 mL), was added and the resulting mixture was stirred for 10 min. Then, benzyl bromide (270 mg, 0.19 mL, 1.58 mmol, 1.5 equiv) was added at 0°C and the reaction mixture was stirred for 10 minutes at 0°C followed by 15 h at room temperature. H_2O (10 mL) was added, the two layers were separated, and the aqueous layer was extracted with EA (3 × 15 mL). The crude residue was purified *via* MPLC (50 g silica gel, 12-100% EA in *n*-heptane) to yield 2,7-anhydro-3-*O*-benzyl-4,5-*O*-isopropylidene-1-*O*-trityl-D-*glycero*-β-D-*ribo*-hept-2-ulopyranose (**S6**) as a colourless oil (550 mg, 93%); R_f = 0.67 (*n*-heptane/EA 1:1, CAM); $[\alpha]_D^{20}$ = –83.4 (c = 1.0 in $CHCl_3$); ^1H NMR (600 MHz, d_6-DMSO) δ = 7.41-6.87 (m, 20H, H^{Ar}), 4.78 (d, $^3J_{6,7b}$ = 5.0 Hz, 1H, H-6), 4.73 (d, $^2J_{H,H}$ = 10.8 Hz, 1H, H^{Bn}), 4.47 (dd, $^3J_{4,3}$ = 6.2 Hz, $^3J_{4,5}$ = 6.2 Hz, 1H, H-4), 4.25 (d, $^2J_{H,H}$ = 10.8 Hz, 1H, H^{Bn}), 4.14 (dd, $^3J_{5,4}$ = 6.2 Hz, $^3J_{6,5}$ = 1.46 Hz, 1H, H-5), 3.95 (d, $^2J_{7a,7b}$ = 7.5 Hz, 1H, H-7a), 3.71 (dd, $^2J_{7b,7a}$ = 7.5 Hz, $^3J_{7b,6}$ = 5.0 Hz, 1H, H-7b), 3.70 (d, $^3J_{3,4}$ = 6.2 Hz, 1H, H-3), 3.18 (d, $^2J_{1a,1b}$ = 9.4 Hz, 1H, H-1a), 3.15 (d, $^2J_{1b,1a}$ = 9.4 Hz, 1H, H-1b), 128 (s, 3H, CH_3^{isopr}), 1.28 (s, 3H, CH_3^{isopr}); ^{13}C NMR (151 MHz, d_6-DMSO) δ = 143.45 (3 × C^{Trt}), 138.00 (C^{Ar}), 128.24 (6 × CH^{Trt}), 127.88 (2 × CH^{Ar}), 127.86 (6 × CH^{Trt}), 127.63 (2 × CH^{Ar}), 127.18 (C^{Ar}), 127.02 (3 × CH^{Trt}), 109.73 (C^{isopr}), 106.74 (C-2), 85.84 (C^{Trt}), 74.00 (C-8), 73.02 (C-3), 72.81 (C-5), 72.79 (C-6), 71.08 (C-4), 66.01 (C-7), 63.77 (C-1), 26.17 (CH_3^{isopr}), 25.59 (CH_3^{isopr}); HRMS (+ESI): *m/z* calc. for $C_{36}H_{36}O_6Na^+$ $[M+Na]^+$: 587.2400, found: 587.2404.

1.1.4.5 2,7-Anhydro-3-*O*-benzyl-D-*glycero*-β-D-*ribo*-hept-2-ulopyranose (S7)

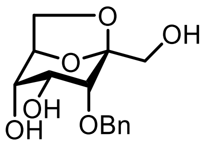

S7
$C_{14}H_{18}O_6$
282.29 g/mol

Compound **S7** was synthesized following a protocol by Köll *et al.*[16] 2,7-Anhydro-3-*O*-benzyl-4,5-*O*-isopropyliden-1-*O*-trityl-D-*glycero*-β-D-*ribo*-hept-2-ulopyranose (**S6**, 966 mg, 1.71 mmol, 1eqiv.) was dissolved in glacial acetic acid (10.6 mL) and water (2.6 mL) was added. The reaction mixture was heated to 80°C for 5 h before removing the solvents *in vacuo*. The residue was co-evaporated with toluene (3 × 15 mL) and the crude residue was purified *via* MPLC (50 g silica gel, 24-100% EA in *n*-heptane) to yield 2,7-anhydro-3-*O*-benzyl-D-*glycero*-β-D-*ribo*-hept-2-ulopyranose (**S7**) as a colourless oil (459 mg, 95%); R_f = 0.45 (EA, CAM); $[\alpha]_D^{20}$ = –60.7 (*c* = 1.0 in $CHCl_3$); ^1H NMR (600 MHz, d_6-DMSO) δ = 7.38-7.26 (m, 5H, H^{Ar}), 4.92 (dd, $^3J_{OH,1a}$ = 6.7 Hz, $^3J_{OH,1b}$ = 5.4 Hz, 1H, OH-3), 4.81 (d, $^2J_{H,H}$ = 11.0 Hz, 1H, H^{Bn}), 4.56 (d, $^3J_{OH4,4}$ = 8.1 Hz, 1H, OH-4), 4.52 (d, $^2J_{H,H}$ = 11.0 Hz, 1H, H^{Bn}), 4.45 (dd, $^3J_{5,OH5}$ = 5.1, $^3J_{5,4}$ = 4.8 Hz, 1H, H-5), 3.76 (ddd, $^3J_{4,OH4}$ = 8.1 Hz, $^3J_{4,5}$ = 4.8 Hz, $^3J_{4,3}$ = 4.3 Hz, 1H, H-4), 3.71 (d, $^2J_{7a,7b}$ = 7.7 Hz, 1H, H-7a), 3.67 (d, $^3J_{3,4}$ = 4.3 Hz, 1H, H-3), 3.65 (dd, $^2J_{1a,1b}$ = 11.5 Hz, $^3J_{1a,OH1}$ = 6.7, 1H, H-1a), 3.60 (m, 1H, H-6), 3.58 (d, $^2J_{OH,5}$ = 5.1 Hz, 1H, OH-5), 3.57 (d, $^2J_{7b,7a}$ = 7.7 Hz, 1H, H-7b), 3.41

(dd, $^2J_{1b,1a}$ = 11.5 Hz, $^3J_{1b,OH}$ = 5.4 Hz, 1H, H-1b); ^{13}C NMR (151 MHz, d_6-DMSO) δ = 138.81 (C^{Ar}) 128.2 (2 × CH^{Ar}), 127.7 (2 × CH^{Ar}), 127.4 (C^{Ar}), 106.8 (C-2), 77.6 (C-6), 77.0 (C-4), 74.4 (C-8), 69.9 (C-3), 64.8 (C-7), 64.2 (C-6), 61.1 (C-1); HRMS (+ESI): *m/z* calc. for $C_{14}H_{18}O_6Na^+$ [M+Na]$^+$: 305.0996, found: 305.1002.

1.1.4.6 1,4,5-Tri-*O*-acetyl-2,7-anhydro-3-*O*-benzyl-D-*glycero*-β-D-*ribo*-hept-2-ulopyranose (S8)

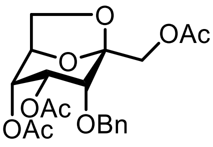

S8
$C_{20}H_{24}O_9$
408.40 g/mol

Compound **S8** was synthesized following a protocol by Heyns *et al.*[15] 2,7-Anhydro-3-*O*-benzyl-D-*glycero*-β-D-*ribo*-hept-2-ulopyranose (**S7**, 260 mg, 0.92 mmol, 1 equiv.) was dissolved in dry pyridine (3.2 mL) under Ar atmosphere and cooled to 0°C. After the consecutive addition of acetic anhydride (564.6 mg, 0.52 mL, 5.53 mmol, 6 equiv.) and DMAP (5.6 mg, 0.046 mmol, 0.05 equiv.), the ice bath was removed, and the reaction was stirred at room temperature for 3 h. The solvent as removed *in vacuo*, the residue was co-evaporated with toluene (3 × 15 mL) and the crude residue was purified *via* MPLC (25 g silica gel, 12-100% EA in *n*-heptane) to yield 1,4,5-tri-*O*-acetyl-2,7-anhydro-3-*O*-benzyl-D-*glycero*-β-D-*ribo*-hept-2-ulopyranose (**S8**) as a colourless oil (310 mg, 77%); R_f = 0.84 (EA, UV & CAM); $[\alpha]_D^{20}$ = –41.4 (c = 0.76 in $CHCl_3$); ^{1}H NMR (700 MHz, d_6-DMSO) δ = 7.38-7.28 (m, 5H, H^{Ar}), 5.15 (dd, $^3J_{5,4}$ = 4.5 Hz, $^3J_{5,6}$ = 2.3 Hz, 1H, H-5), 5.12 (dd, $^3J_{4,3}$ = 4.4 Hz, $^3J_{4,5}$ = 4.5 Hz, 1H, H-4), 4.67 (dd, $^3J_{6,7b}$ = 5.4 Hz, $^3J_{6,5}$ = 2.3 Hz, 1H, H-6), 4.61 (d, $^2J_{H,H}$ = 11.4 Hz, 1H, H^{Bn}), 4.48 (d, $^2J_{H,H}$ = 11.4 Hz, 1H, H^{Bn}), 4.32 (d, $^2J_{1a,1b}$ = 11.7 Hz, 1H, H-1a), 4.10 (d, $^2J_{1b,1a}$ = 11.7 Hz, 1H, H-1b), 3.98 (d, $^2J_{7a,7b}$ = 8.1 Hz, 1H, H-7a), 3.85 (d, $^3J_{3,4}$ = 4.4 Hz, 1H, H-3), 3.67 (dd, $^2J_{7b,7a}$ = 8.1 Hz, $^3J_{7b,6}$ = 5.4 Hz, 1H, H-7b), 2.06 (s, 3H, CH_3^{Ac-5}), 2.03 (s, 3H, CH_3^{Ac-1}), 1.94 (s, 3H, CH_3^{Ac-4}); ^{13}C NMR (176 MHz, d_6-DMSO) δ = 170.0o (C^{Ac-5}), 169.75 (C^{Ac-1}), 169.35 (C^{Ac-4}), 138.42 (C^{Ar}), 128.11 (2 × CH^{Ar}), 127.71 (2 × CH^{Ar}), 127.52 (CH^{Ar}), 105.45 (C-2), 74.93 (C-6), 74.20 (C-3), 73.85 (C-8), 67.73 (C-5), 65.83 (C-4), 65.54 (C-7), 61.85 (C-1), 20.69 (CH_3^{Ac-5}), 20.50 (CH_3^{Ac-1}), 20.45 (CH_3^{Ac-4}); HRMS (+ESI): *m/z* calc. for $C_{20}H_{24}O_9H^+$ [M+H]$^+$: 409.1491, found: 409.1493.

1.1.4.7 1,4,5-Tri-*O*-acetyl-2,7-anhydro-D-*glycero*-β-D-*ribo*-hept-2-ulopyranose (S9)

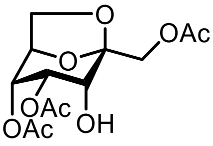

S9
$C_{13}H_{18}O_9$
318.28 g/mol

1,4,5-Tri-*O*-acetyl-2,7-anhydro-3-*O*-benzyl-D-*glycero*-β-D-*ribo*-hept-2-ulopyranose (**S8**, 370 mg, 0.91 mmol, 1 equiv.) was dissolved in ethanol (5 mL) and Pd/C (10% Pd, 85 mg, 0.057 mmol, 0.13 equiv.) was added. The reaction mixture was degassed (3 freezing/thawing cycles with liquid N_2 under vacuum) and stirred at room temperature under 1atm H_2 pressure for 24 h. The catalyst was removed by filtration over Celite® and the solvent was removed *in vacuo*. The crude residue was purified via MPLC (25 g silica gel, 24-100% EA in *n*-heptane) to yield 1,4,5-tri-*O*-acetyl-2,7-anhydro-D-*glycero*-β-D-*ribo*-hept-2-ulopyranose (**S9**) as a colourless oil (261 mg, 90%); R_f = 0.34 (*n*-heptane/EA 1:3, CAM); $[\alpha]_D^{20}$ = –43.3 (c = 0.8 in $CHCl_3$); ^{1}H NMR (600 MHz, $CDCl_3$) δ = 5.21 (ddd, $^3J_{5,4}$ = 4.4 Hz, $^3J_{5,6}$ = 2.6 Hz, $^3J_{3,5}$ = 1.1 Hz, 1H, H-5), 5.16 (dd, $^3J_{4,3}$ = 4.3 Hz, $^3J_{4,5}$ = 4.4 Hz, 1H, H-4), 4.70 (ddd,

$^{3}J_{6,7b}$ = 5.1 Hz, $^{3}J_{6,7a}$ = 2.7 Hz, $^{3}J_{6,5}$ = 2.6 Hz, 1H, H-6), 4.47 (d, $^{2}J_{1a,1b}$ = 12.0 Hz, 1H, H-1a), 4.36 (d, $^{2}J_{1b,1a}$ = 12.0 Hz, 1H, H-1b), 3.95 (ddd, $^{3}J_{3,OH}$ = 11.3 Hz, $^{3}J_{3,4}$ = 4.3 Hz, $^{3}J_{3,5}$ = 1.1 Hz, 1H, H-3), 3.94 (dd, $^{2}J_{7a,7b}$ = 8.3 Hz, $^{3}J_{7a,6}$ = 2.7 Hz, 1H, H-7a), 3.92 (dd, $^{2}J_{7a,7b}$ = 8.3 Hz, $^{3}J_{7b,6}$ = 5.1 Hz, 1H, H-7b), 2.40 (d, $^{3}J_{OH,3}$ = 11.3 Hz, 1H, OH-3), 2.18 (s, 3H, $CH_3^{Ac\text{-}5}$), 2.12 (s, 3H, $CH_3^{Ac\text{-}1}$), 2.10 (s, 3H, $CH_3^{Ac\text{-}4}$); ^{13}C NMR (150.93 MHz, $CDCl_3$) δ = 170.52 ($C^{Ac\text{-}5}$), 169.96 ($C^{Ac\text{-}1}$), 169.87 ($C^{Ac\text{-}4}$), 106.70 (C-2), 75.39 (C-6), 69.74 (C-5), 68.93 (C-3), 66.25 (C-7), 65.27 (C-4), 62.23 (C-1), 21.07 ($CH_3^{Ac\text{-}5}$), 20.91 ($CH_3^{Ac\text{-}1}$), 20.90 ($CH_3^{Ac\text{-}4}$); HRMS (+ESI): *m/z* calc. for $C_{13}H_{18}O_9Na^+$ $[M+Na]^+$: 341.0843, found: 341.0846

1.1.4.8 1,4,5-Tri-*O*-acetyl-2,7-anhydro-3-*O*-*para*-toluenesulfonyl-D-*glycero*-β-D-*ribo*-hept-2-ulopyranose (S10)

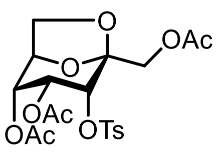

S10
$C_{20}H_{24}O_{11}S$
472.46 g/mol

Compound **S10** was synthesized following a protocol by Hofferberth *et al.*[12] 1,4,5-Tri-*O*-acetyl-2,7-anhydro-D-*glycero*-β-D-*ribo*-hept-2-ulopyranose (**S9**, 160 mg, 0.50 mmol, 1 equiv.) was dissolved in dry pyridine (2 mL) under Ar atmosphere and cooled to 0°C. After the consecutive addition of *para*-toluenesulfonyl chloride (239.6 mg, 1.76 mmol, 2.5 equiv.), DMAP (3.07 mg, 0.025 mmol, 0.05 equiv.) and triethylamine (254.4 mg, 0.35 mL, 2.51 mmol, 5 equiv.) the ice bath was removed, and the reaction mixture was stirred at room temperature for 6 h. The solvent was removed *in vacuo* and the remaining pyridine was co-evaporated with toluene (2 × 15 mL). The residue was dissolved in water and extracted with DCM (3 × 15 mL). The crude residue was purified via MPLC (25 g silica gel, 24-100% EA in *n*-heptane) to yield 1,4,5-tri-*O*-acetyl-2,7-anhydro-3-*O*-*para*-toluenesulfonyl-D-*glycero*-β-D-*ribo*-hept-2-ulopyranose (**S10**) as a light yellow solid (232 mg, 98 %); R_f = 0.57 (*n*-heptane/EA 1:3, UV & CAM); $[\alpha]_D^{20}$ = –21.6 (c = 1.0 in $CHCl_3$); ^{1}H NMR (600 MHz, $CDCl_3$) δ = 7.82 (m, 2H, H^{Ar}), 7.33 (m, 2H, H^{Ar}), 5.31 (dd, $^{3}J_{4,3}$ = 4.5 Hz, $^{3}J_{4,5}$ = 4.5 Hz, 1H, H-4), 5.17 (ddd, $^{3}J_{5,4}$ = 4.5 Hz, $^{3}J_{5,6}$ = 2.5 Hz, $^{3}J_{5,7a}$ = 0.7 Hz 1H, H-5), 5.03 (d, $^{3}J_{3,4}$ = 4.5 Hz, 1H, H-3), 4.71 (dd, $^{3}J_{6,7b}$ = 5.1 Hz, 1H $^{3}J_{6,5}$ = 2.5 Hz, H-6), 4.09 (d, $^{2}J_{1a,1b}$ = 12.0 Hz, 1H, H-1a), 4.04 (d, $^{2}J_{1b,1a}$ = 12.0 Hz, 1H, H-1b), 3.95 (d, $^{2}J_{7a,7b}$ = 8.4 Hz, 1H, H-7a), 3.90 (dd, $^{2}J_{7b,7a}$ = 8.4, $^{3}J_{7b,6}$ = 5.1 Hz, 1H, H-7b), 2.44 (s, 3H, CH_3^{Tos}), 2.10 (s, 3H, $CH_3^{Ac\text{-}5}$), 2.08 (s, 3H, $CH_3^{Ac\text{-}1}$), 2.02 (s, 3H, $CH_3^{Ac\text{-}4}$); ^{13}C NMR (151 MHz, $CDCl_3$) δ = 170.46 ($C^{Ac\text{-}5}$), 169.79 ($C^{Ac\text{-}1}$), 169.63 ($C^{Ac\text{-}4}$), 145.08 (s, C^{Ar}), 134.24 (s, C^{Ar}), 129.70 (2 CH^{Ar}), 127.58 (2 CH^{Ar}), 104.65 (C-2), 75.57 (C-6), 72.81 (C-3), 67.73 (C-5), 66.07 (C-7),63.00 (C-4), 62.18 (C-1), 21.63 (s, CH_3^{Tos}), 20.75 ($CH_3^{Ac\text{-}5}$), 20.64 ($CH_3^{Ac\text{-}1}$), 20.59 ($CH_3^{Ac\text{-}4}$); HRMS (+ESI): *m/z* calc. for $C_{20}H_{24}O_{11}SNa^+$ $[M+Na]^+$: 495.0932, found: 495.0931.

1.1.4.9 1,2,4,5,6-Penta-*O*-acetyl-3-*O*-*para*-toluenesulfonyl-D-*glycero*-α-D-*ribo*-hept-2-ulopyranose (S11)

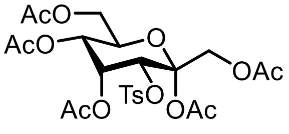

S11
$C_{24}H_{30}O_{14}S$
574.55 g/mol

Compound **S11** was synthesized following a protocol by Zottola *et al.*[19] 1,4,5-Tri-*O*-acetyl-2,7-anhydro-3-*O*-*para*-toluenesulfonyl-D-*glycero*-β-D-*ribo*-hept-2-ulopyranose (**S10**, 100 mg, 0.21 mmol, 1 equiv.) was dissolved in acetic anhydride (1.6 mL) under Ar atmosphere and cooled to 0°C. After the dropwise addition of triethylsilyltrifluoromethanesulfonate (44.3 mg, 38 µL, 0.32 mmol, 1.5 equiv.) the reaction mixture was stirred at 0°C for 24 h. A sat. aqueous $NaHCO_3$-sol. was added to pH 5. After diluting the mixture with DCM (10 mL), the layers were separated, and the aqueous layer was extracted with DCM (3× 10 mL). The combined organic layers were dried ($MgSO_4$), and the solvent was removed *in vacuo*. The crude residue was purified by reversed-phase chromatography (C_{18}-modified silica gel, MeCN/H_2O 1:1) to give 1,2,4,5,6-penta-*O*-acetyl-3-*O*-*para*-toluenesulfonyl-D-*glycero*-β-D-*ribo*-hept-2-ulopyranose (**S11**) as a colourless solid (62.74 mg, 52%); R_f = 0.57 (*n*-heptane/EA 1:3, UV & CAM); $[\alpha]_D^{20}$ 57.7 (*c* = 0.73 in $CHCl_3$); ^1H NMR (700 MHz, $CDCl_3$) δ = 7.78-7.75 (m, 2H, H^{Ar}), 7.38-7.32 (m, 2H, H^{Ar}), 5.64 (dd, $^3J_{4,3}$ = 3.3 Hz, $^3J_{4,5}$ = 3.3 Hz, 1H, H-4) 5.00 (dd, $^3J_{5,4}$ = 3.3 Hz, $J_{5,6}$ = 10.3 Hz, 1H, H-5), 4.89 (d, $^3J_{3,4}$ = 3.3 Hz, 1H, H-3), 4.80 (d, $^2J_{1a,1b}$ = 11.7 Hz, 1H, H-1a), 4.23 (d, $^2J_{1b,1a}$ = 11.7 Hz, 1H, H-1b), 4.23 (d, $^2J_{7a,7b}$ = 12.1 Hz, $^3J_{7a,6}$ = 5.2 Hz, 1H, H-7a), 4.18 (ddd, $^3J_{6,5}$ = 10.3 Hz, $^3J_{6,7a}$ = 5.2 Hz, $^3J_{6,7b}$ = 2.0 Hz, 1H, H-6), 4.14 (dd, $^2J_{7b,7a}$ = 12.1 Hz, $^3J_{7b,6}$ = 2.0 Hz, 1H, H-7b), 2.46 (s, CH_3^{Tos}), 2.10 (s, 3H, $CH_3^{Ac\text{-}4}$), 2.07 (s, 3H, $CH_3^{Ac\text{-}2}$), 2.06 (s, 3H, $CH_3^{Ac\text{-}7}$), 2.06 (s, 3H, $CH_3^{Ac\text{-}5}$), 1.98 (s, 3H, $CH_3^{Ac\text{-}1}$); ^{13}C NMR (176 MHz, d_6-DMSO) δ = 170.65 ($C^{Ac\text{-}7}$), 169.62 ($C^{Ac\text{-}1}$), 169.35 ($C^{Ac\text{-}4}$), 169.06 ($C^{Ac\text{-}5}$), 167.18 ($C^{Ac\text{-}2}$), 145.69 (C^{Ar}), 132.90 (C^{Ar}), 130.00 (2 CH^{Ar}), 127.91 (2 CH^{Ar}), 100.17 (C-2), 71.05 (C-3), 67.21 (C-4), 66.75 (C-6), 65.14 (C-5), 62.26 (C-1), 61.60 (C-7), 21.71 (CH_3^{Tos}), 21.69 ($CH_3^{Ac\text{-}4}$), 20.69 ($CH_3^{Ac\text{-}7}$), 20.69 ($CH_3^{Ac\text{-}5}$), 20.56 ($CH_3^{Ac\text{-}2}$), 20.42 ($CH_3^{Ac\text{-}1}$); HRMS (+ESI): *m/z* calc. for $C_{24}H_{30}O_{14}SNa^+$ $[M+Na]^+$: 597.1248, found: 597.1253.

1.1.4.10 1,4,5-Tri-*O*-acetyl-2,7-anhydro-3-*O*-4-nitrobenzenesulfonyl-D-*glycero*-β-D-*ribo*-hept-2-ulopyranose (S12)

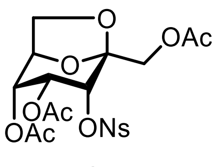

S12
$C_{19}H_{21}NO_{13}S$
503.43 g/mol

Compound **S12** was synthesized following a protocol by Hofferberth *et al.*[12] 1,4,5-Tri-*O*-acetyl-2,7-anhydro-D-*glycero*-β-D-*ribo*-hept-2-ulopyranose (**S9**, 70 mg, 0.22 mmol, 1 equiv.) was dissolved in dry pyridine (2 mL) under Ar atmosphere and cooled to 0°C. After the addition of 4-nitrobenzolsulfonyl chloride (58.49 mg, 0.26 mmol, 1.2 equiv.), DMAP (1.5 mg, 0.013 mmol, 0.05 equiv.) and triethylamine (44.52 mg, 0.061 mL, 0.44 mmol, 2 equiv.) the ice bath was removed, and the reaction mixture was stirred at room temperature for 6 h. The solvent was removed *in vacuo* and the remaining pyridine was co-evaporated with toluene (2 × 10 mL). The residue was dissolved in water and extracted with DCM (3 × 10 mL). The combined organic layers were dried ($MgSO_4$), concentrated *in vacuo* and the remaining crude residue purified via MPLC (25 g silica gel, 24-100% EA in *n*-heptane) to yield

1,4,5-tri-*O*-acetyl-2,7-anhydro-3-*O*-4-nitrobenzenesulfonyl-D-*glycero*-β-D-*ribo*-hept-2-ulopyranose (**S12**) as a yellowish solid (104 mg, 98 %); R_f = 0.57 (*n*-heptane/EA 1:3, CAM); $[\alpha]_D^{20}$ = –17.6 (c = 0.8 in $CHCl_3$); ^1H NMR (700 MHz, $CDCl_3$) δ = 8.43-8.36 (m, 2H, H^{Ar}), 8.17-8.13 (m, 2H, H^{Ar}), 5.35 (dd, $^3J_{4,3}$ = 4.5 Hz, $^3J_{4,5}$ = 4.5 Hz, 1H, H-4) 5.18 (dd, $^3J_{5,4}$ = 4.5 Hz, $J_{5,6}$ = 2.6 Hz, 1H, H-5), 5.13 (d, $^3J_{3,4}$ = 4.5 Hz, 1H, H-3), 4.74 (dd, $^3J_{6,7b}$ = 5.0 Hz, $^3J_{6,5}$ = 2.6 Hz, 1H, H-6), 4.12 (d, $^2J_{1a,1b}$ = 12.2 Hz, 1H, H-1a), 4.04 (d, $^2J_{1b,1a}$ = 12.2 Hz, 1H, H-1b), 3.97 (d, $^2J_{7a,7b}$ = 8.6 Hz, 1H, H-7a), 3.93 (dd, $^2J_{7b,7a}$ = 8.6 Hz, $^3J_{7b,6}$ = 5.0 Hz, 1H, H-7b), 2.11 (s, 3H, $CH_3^{Ac\text{-}5}$), 2.10 (s, 3H, $CH_3^{Ac\text{-}1}$), 2.04 (s, 3H, $CH_3^{Ac\text{-}4}$); ^{13}C NMR (151 MHz, $CDCl_3$) δ = 170.19 ($C^{Ac\text{-}5}$), 169.71 ($C^{Ac\text{-}1}$), 169.33 ($C^{Ac\text{-}4}$), 142.72 (s, C^{Ar}), 133.66 (s, C^{Ar}), 128.86 (2 CH^{Ar}), 124.35 (2 CH^{Ar}), 104.42 (C-2), 75.57 (C-6), 73.77 (C-3), 67.70 (C-5), 66.15 (C-7), 62.90 (C-4), 62.05 (C-1), 20.78 ($CH_3^{Ac\text{-}5}$), 20.59 ($CH_3^{Ac\text{-}1}$), 20.57 ($CH_3^{Ac\text{-}4}$); HRMS (+ESI): *m/z* calc. for $C_{19}H_{21}NO_{13}SNa^+$ $[M+Na]^+$: 526.0626, found: 526.0621.

1.1.4.11 1,2,4,5,6-Penta-*O*-acetyl-3-*O*-4-nitrobenzenesulfonyl-D-*glycero*-α-D-*ribo*-hept-2-ulopyranose (S13)

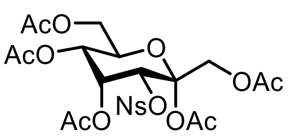

S13
$C_{23}H_{27}NO_{16}S$
605.52 g/mol

Compound **S13** was synthesized following a protocol by Zottola *et al.*[19] 1,4,5-Tri-*O*-acetyl-2,7-anhydro-3-*O*-4-nitrobenzenesulfonyl-D-*glycero*-β-D-*ribo*-hept-2-ulopyranose (**S12**, 50 mg, 0.1 mmol, 1 equiv.) was dissolved in acetic anhydride (0.8 mL) under Ar atmosphere and cooled to 0°C. After the dropwise addition of triethylsilyltrifluoromethanesulfonate (20.8 mg, 0.018 mL, 0.15 mmol, 1.5 equiv.) the reaction mixture was stirred at 0°C for 24 h. A sat. aqueous $NaHCO_3$-sol. was added to pH 5 and the reaction mixture diluted with DCM (10 mL). The layers were separated, and the aqueous layer was extracted with DCM (3 × 10 mL). The combined organic layers were dried ($MgSO_4$), and the solvent was removed under reduced pressure. The crude residue was purified by reversed-phase chromatography (C_{18}-silica gel, MeCN/H_2O 1:1) to give 1,2,4,5,6-penta-*O*-acetyl-3-*O*-4-nitrobenzenesulfonyl-D-*glycero*-β-D-*ribo*-hept-2-ulopyranose (**S13**) as a colourless solid (27.9 mg, 46%); R_f = 0.57 (*n*-heptane/EA 1:3, UV & CAM); $[\alpha]_D^{20}$ = +27.7 (c = 0.83 in $CHCl_3$); ^1H NMR (700 MHz, $CDCl_3$) δ = 8.43-8.38 (m, 2H, H^{Ar}), 8.11-8.07 (m, 2H, H^{Ar}), 5.60 (dd, $^3J_{4,5}$ = 3.4 Hz, $^3J_{4,3}$ = 3.4 Hz, 1H, H-4), 5.08 (d, $^3J_{3,4}$ = 3.4 Hz, 1H, H-3), 5.02 (dd, $^3J_{5,6}$ = 10.4 Hz, $^3J_{5,4}$ = 3.4 Hz, 1H, H-5), 4.82 (d, $^2J_{1a,1b}$ = 11.9 Hz, 1H, H-1a), 4.39 (d, $^2J_{1b,1a}$ = 11.9 Hz, 1H, H-1b), 4.25 (d, $^2J_{7a,7b}$ = 12.3 Hz, $^3J_{7a,6}$ = 5.2 Hz, 1H, H-7a), 4.20 (ddd, $^3J_{6,5}$ = 10.4 Hz, $^3J_{6,7a}$ = 5.2 Hz, $^3J_{6,7b}$ = 2.0 Hz, 1H, H-6), 4.15 (dd, $^2J_{7b,7a}$ = 12.3 Hz, $^3J_{7b,6}$ = 2.0 Hz, 1H, H-7b), 2.14 (s, 3H, $CH_3^{Ac\text{-}4}$), 2.09 (s, 3H, $CH_3^{Ac\text{-}2}$), 2.07 (s, 3H, $CH_3^{Ac\text{-}7}$), 2.03 (s, 3H, $CH_3^{Ac\text{-}5}$), 1.98 (s, 3H, $CH_3^{Ac\text{-}1}$); ^{13}C NMR (176 MHz, d_6-DMSO) δ = 170.61 ($C^{Ac\text{-}7}$), 169.67 ($C^{Ac\text{-}1}$), 169.26 ($C^{Ac\text{-}4}$), 169.05 ($C^{Ac\text{-}5}$), 167.00 ($C^{Ac\text{-}2}$), 151.12 (C^{Ar}), 141.50 (C^{Ar}), 129.33 (2 CH^{Ar}), 124.58 (2 CH^{Ar}), 100.06 (C-2), 71.97 (C-3), 66.91 (C-4), 66.87 (C-6), 65.05 (C-5), 62.46 (C-1), 61.47 (C-7), 21.62 ($CH_3^{Ac\text{-}4}$), 20.70 ($CH_3^{Ac\text{-}7}$), 20.68 ($CH_3^{Ac\text{-}5}$), 20.46 ($CH_3^{Ac\text{-}2}$), 20.37 ($CH_3^{Ac\text{-}1}$); HRMS (+ESI): *m/z* calc. for $C_{23}H_{27}NO_{16}SNa^+$ $[M+Na]^+$: 628.0943, found: 628.0948.

2 Biochemical assays

2.1 HILIC-MS

All solvents were of MS-grade. Water and MeCN were purchased from Honeywell (Seelze, Germany). Concentrated formic acid was obtained from VWR (Radnor, Pennsylvania, USA) and ammonium bicarbonate as well as ammonium hydroxide from Sigma Aldrich (St. Louis, Missouri, USA). Hydrophilic interaction liquid chromatography (HILIC) was performed using an Atlantis Premier BEH Z-HILIC Column (2.1 mm x 150 mm; 1.7 µm) (Waters, Milford, MA, USA) on a Vanquish Horizon HPLC system (Thermo Fisher Scientific, Waltham, MA, USA). Mobile phase A consisted of 15 mM ammonium bicarbonate in water (pH = 9.00), and mobile phase B of 15 mM ammonium bicarbonate in a 9:1 mixture (v/v) of MeCN & H_2O (pH = 9.00). The separation was achieved using a 15 min gradient as follows: the start conditions were 100% B with a gradient to 85% B at 2 min, followed by an isocratic elution at 85 % until 7 min, from 7-10 min a ramp to 40% B was applied, and then an isocratic elution at 40% B from 10-11 min followed. The system was re-equilibrated using 100% B from 11-15 min. The column temperature was 30°C and the flow rate 250 µL/min throughout the chromatographic run. Mass spectrometry analysis was performed on Thermo Scientific™ Orbitrap ID-X™ Tribrid™ Mass Spectrometer with an electrospray ion source (ESI) in negative ion mode. ESI source parameters were set as follows: spray voltage 2900 V, sheath gas 40, auxiliary gas 8, ion transfer tube temperature 275°C. An MS full scan was conducted in a range of 200 – 600 *m/z* at a resolution of 60 000. Chromatographic data was analyzed with FreeStyle.

2.2 Cellular uptake assays

An aqueous solution of sugar (**3DFS** or **4DFS**, 0.1 M) was added to adherent human dermal fibroblasts cultured in DMEM medium containing 10% FBS to a final concentration of 1 mM for the respective sugar. No fluorinated sugar was added to the control sample. Cells were incubated at 37°C for 10 min, then the culture medium was removed and cells were thoroughly washed (3 × 5 mL water). The cells were shock-frozen with liquid nitrogen in the plates and stored at −80°C. Directly before HILIC-MS analysis, the cells were taken from the freezers and instantly lysed by the addition of an 8:2 (v/v) mixture of methanol and water (300 µL), scraped off the plates and transferred into an Eppendorf tube. The suspension was centrifuged (18 000 g, 20 min, 12°C) to clear from precipitates. The supernatant was then collected and evaporated at 45°C with a SpeedVac^{TM}. The remaining small molecule extract was dissolved in a mixture of MeCN/H_2O (1:1 (v/v), 160 µL) and subjected to HILIC-MS-analysis.

2.3 Kinase assay

Recombinant sedoheptulose kinase (SHPK) was expressed in *E.coli* and purified as previously described.[22] ADP-Quest^{TM} Assay kit was obtained from DisoverX and used according to the manufacturer's instructions. In short: 20 µL SHPK (0.009 µg/µL in assay buffer), 10 µL sugar solution (D-sedoheptulose, **3DFS** or **4DFS**; 10 mM in water), 20 µL reagent **A**, 40 µL reagent **B** and 10 µL ATP solution (2 mM in 10 mM HEPES pH 7.6) were mixed in a 96 well plate. Fluorescence was measured on a BioTek Synergy 2 plate reader (excitation wavelength: 530 nm, emission wavelength: 590 nm)

for 10 min (signal read-out every 2 min). All experiments were performed in triplicates. RFU mean of negative control (sedoheptulose with no ATP added) was subtracted from all other values for analysis. The shown graph represents the change in signal from starting- to endpoint compared to the positive control (**sedo**) which was normalized to 100%. Data analysis was done with GraphPad Prism.

Table S1: Relative fluorescence units from the sedoheptulose kinase (SHPK) assay.

Time	Sedo No ATP			Sedo			3DFS			4DFS		
00:03	13634	19726	18726	23908	18552	16844	16004	16613	17368	18110	19984	23988
02:03	13973	20184	19080	26081	20340	18493	16189	17304	17684	20079	22309	26610
04:03	14175	20441	19266	27823	22006	19936	16200	17681	17830	21768	24345	28790
06:03	14263	20565	19335	29169	23442	21240	16111	17899	17882	23157	25870	30395
08:03	14320	20508	19428	29941	24206	22329	16083	18073	17853	23593	26412	30572
10:03	14366	20520	19407	30160	24531	22903	16132	18345	18099	23616	26560	30282

2.4 Stability assays[23]

Human transaldolase (TALDO1) was purchased from ProSpecBio (Rehovot, Israel) and used as received. Transaldolase stability assays contained the following components in water: 1 mM sugar (**sedo** or **4DFS**), 1 mM ATP, 10 mM HEPES pH 7.6, 20 mM KCl, 10 mM $MgCl_2$, 1 mM G3P, 0.045 µg SHPK & 1 µg TALDO1 in a final volume of 25 µL. The reactions were incubated at 30°C for 1.5 h, then diluted 1:100 with MeCN/water 1:1 (v/v) and subjected to HILIC-MS analysis. The shown graph represents the peak area of the respective sugar phosphate compared to the negative control (no TALDO1 added) which was normalized to 100%. All experiments were performed in triplicates. Statistical analysis was done with GraphPad Prism.

Human transketolase (TKT) was purchased from ProSpecBio (Rehovot, Israel) and used as received. Transketolase stability assays contained the following components in water: 1 mM sugar (**sedo** or **4DFS**), 1 mM ATP, 10 mM HEPES pH 7.6, 20 mM KCl, 10 mM $MgCl_2$, 1 mM G3P, 0.5 mM ThPP, 0.045 µg SHPK & 1 µg TKT in a final volume of 25 µL. The reactions were incubated at 30°C for 0.5 h, then diluted 1:100 with MeCN/water 1:1 (v/v) and subjected to HILIC-MS analysis. The shown graph represents the peak area of the respective sugar phosphate compared to the negative control (no TKT added) which was normalized to 100%. All experiments were performed in triplicates. Statistical analysis was done with GraphPad Prism.

3 Appendix
3.1 Chromatograms
3.1.1 Uptake Assay

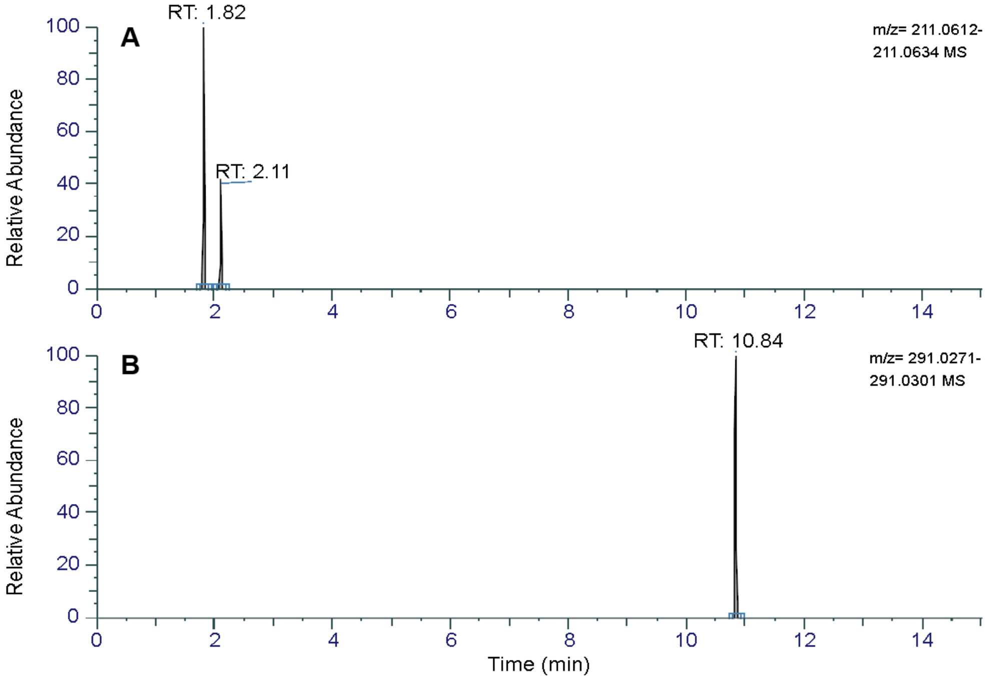

Figure S1: Extracted-ion chromatograms (**A**: *m/z* = 211.0623, **B**: *m/z* = 291.0286; mass tolerance = 5 ppm) of uptake assay control sample (no fluorinated sugar added). *m/z* calc. for $C_7H_{12}FO_6^-$ [M-H]$^-$: 211.0623 (**3DFS** & **4DFS**). *m/z* calc. for $C_7H_{13}FO_9P^-$ [M-H]$^-$: 291.0286 (**3DFS**- & **4DFS**-phosphate).

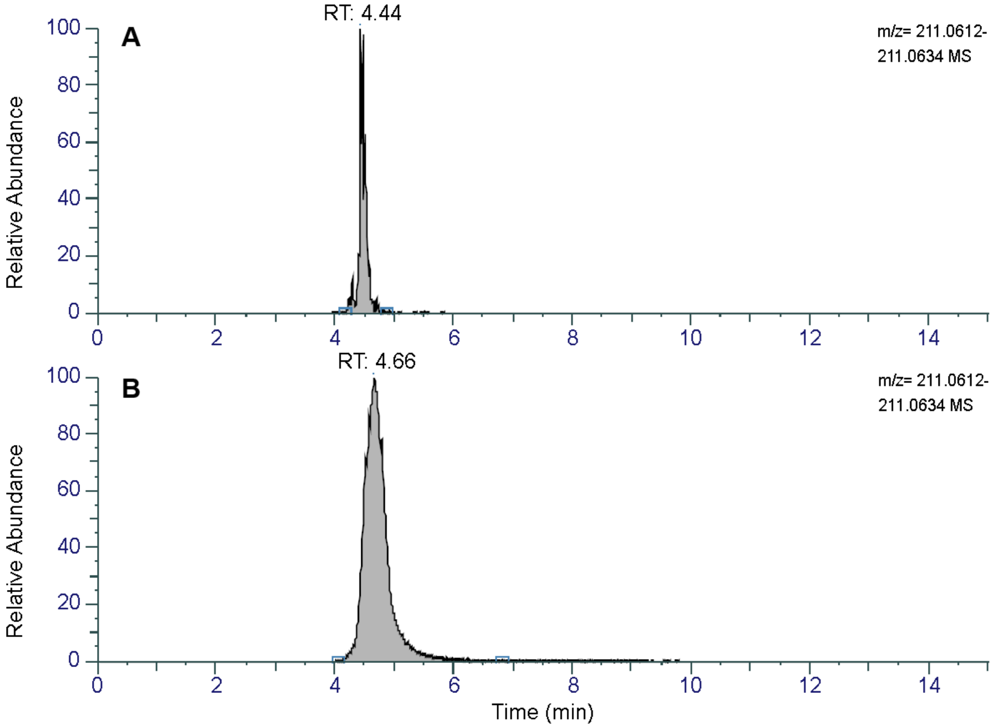

Figure S2: Extracted-ion chromatograms (*m/z* = 211.0623; mass tolerance = 5 ppm) of **3DFS** (**A**) and **4DFS** (**B**) standards (10 µM in MeCN/H_2O (1:1 v/v/). *m/z* calc. for $C_7H_{12}FO_6^-$ [M-H]$^-$: 211.0623 (**3DFS** & **4DFS**).

3.1.2 Stability assay - TALDO1

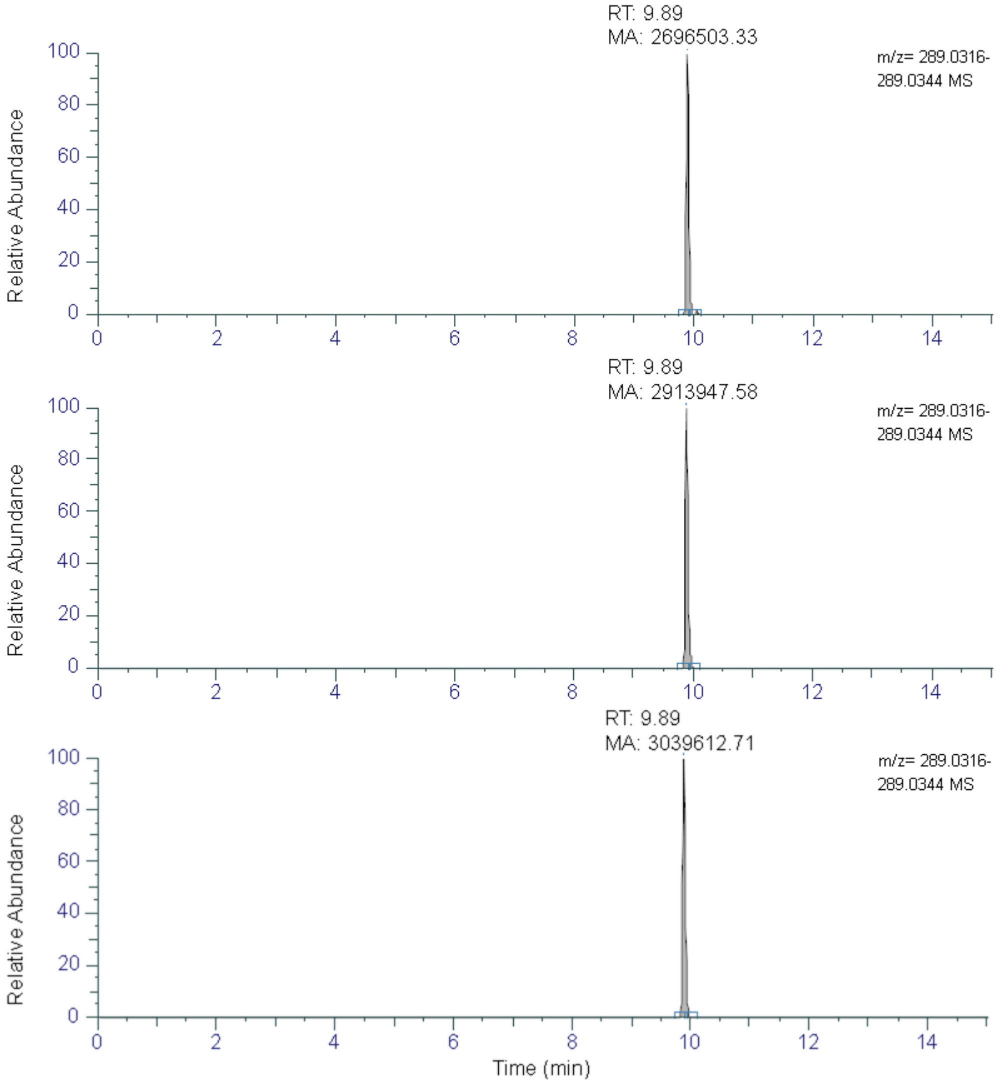

Figure S3: Extracted-ion chromatograms (*m/z* = 289.0330; mass tolerance = 5 ppm) of **sedo** triplicate control samples for TALDO1 – stability assay (no TALDO1 added). *m/z* calc. for $C_7H_{14}O_{10}P^-$ $[M\text{-}H]^-$: 289.0330 (**S7P**).

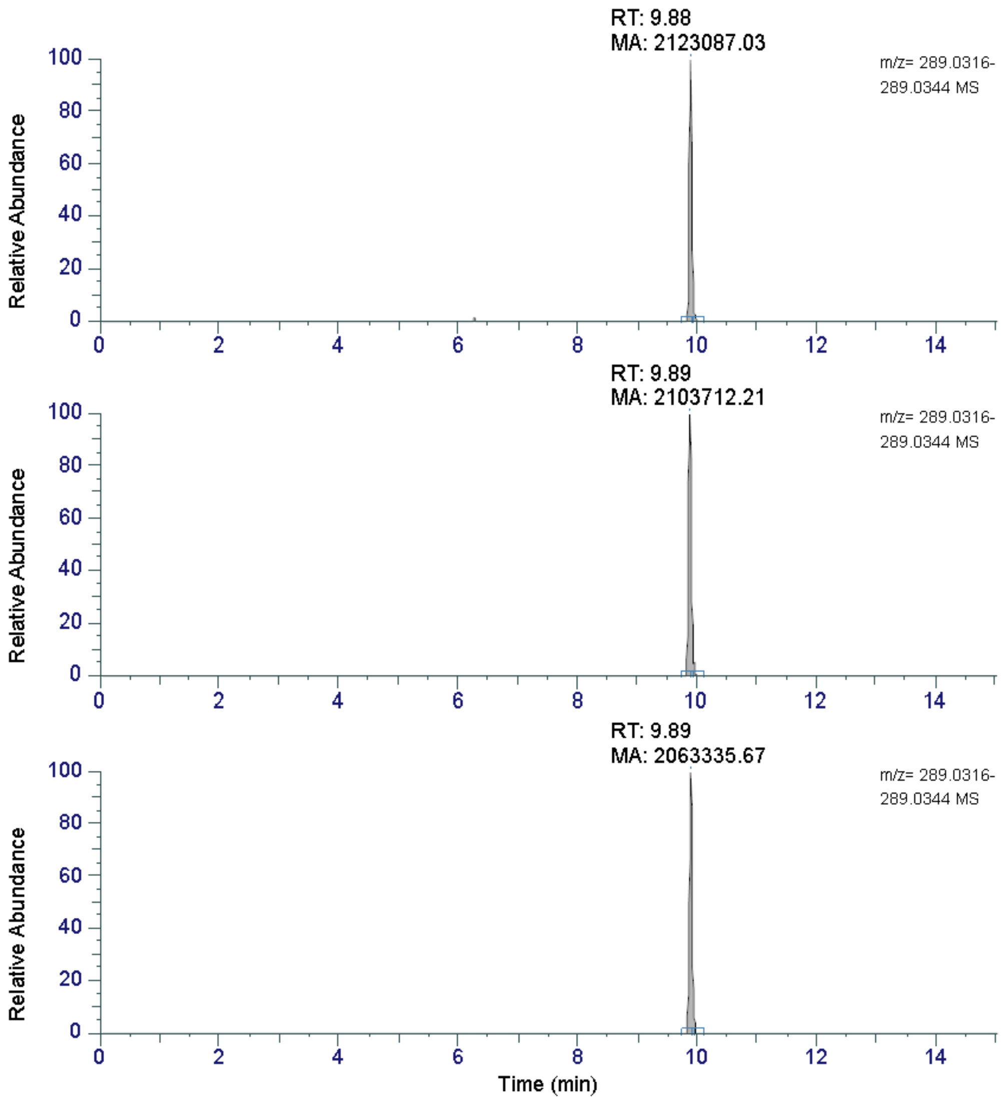

Figure S4: Extracted-ion chromatograms (*m/z* = 289.0330; mass tolerance = 5 ppm) of **sedo** triplicate samples for TALDO1 – stability assay. *m/z* calc. for $C_7H_{14}O_{10}P^-$ $[M\text{-}H]^-$: 289.0330 (**S7P**).

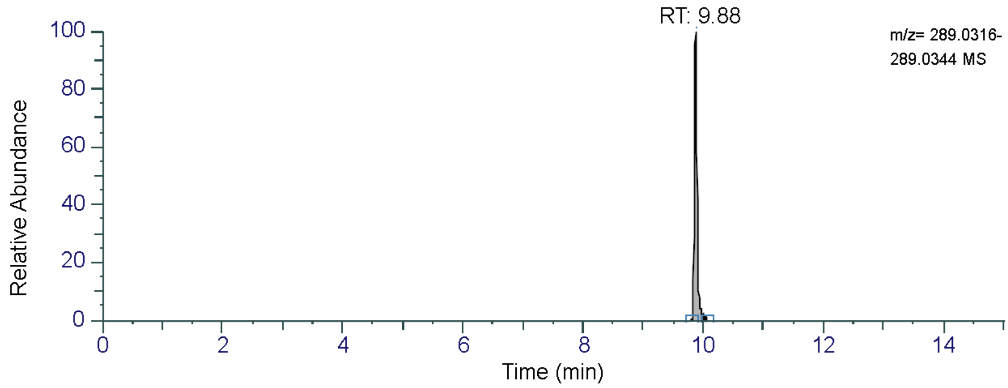

Figure S5: Extracted-ion chromatogram (*m/z* = 289.0330; mass tolerance = 5 ppm) of **S7P** standard (10 µM in MeCN/H_2O (1:1 v/v/). *m/z* calc. for $C_7H_{14}O_{10}P^-$ $[M\text{-}H]^-$: 289.0330 (**S7P**).

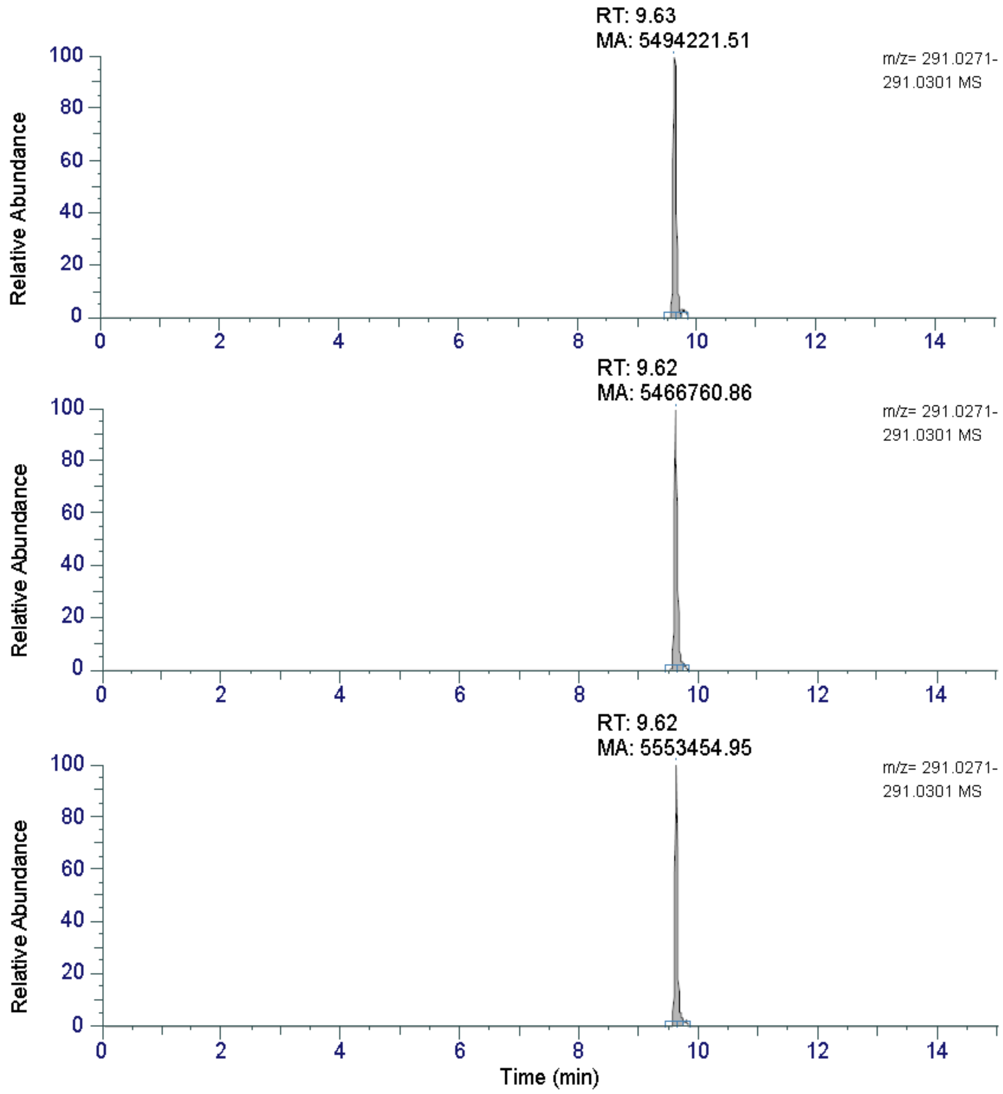

Figure S6: Extracted-ion chromatograms (*m/z* = 291.0286; mass tolerance = 5 ppm) of **4DFS** triplicate control samples for TALDO1 – stability assay (no TALDO1 added). *m/z* calc. for $C_7H_{13}FO_9P^-$ $[M-H]^-$: 291.0286 (**4DFS**-phosphate).

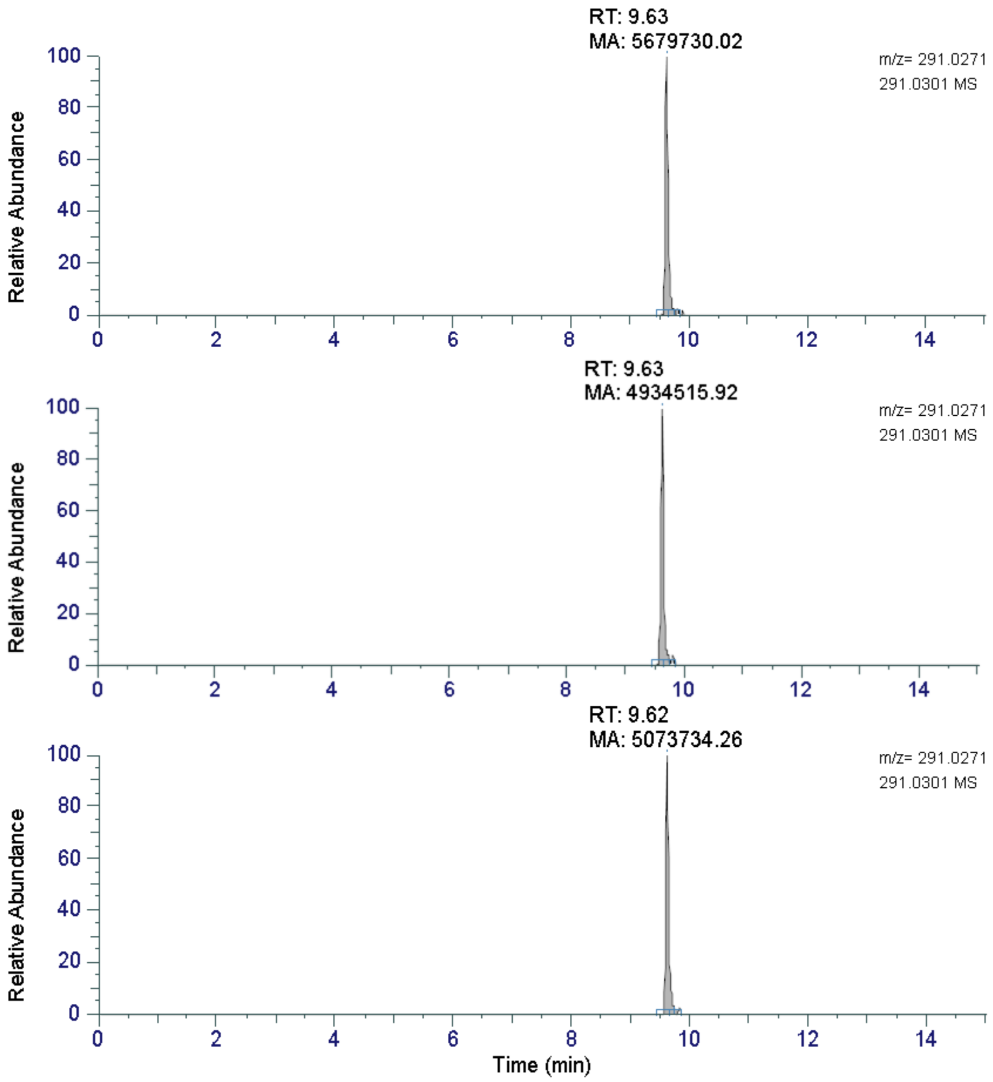

Figure S7: Extracted-ion chromatograms (*m/z* = 291.0286; mass tolerance = 5 ppm) of **4DFS** triplicate samples for TALDO1 – stability assay. *m/z* calc. for $C_7H_{13}FO_9P^-$ $[M\text{-}H]^-$: 291.0286 (**4DFS**-phosphate).

3.1.3 Stability assay – TKT

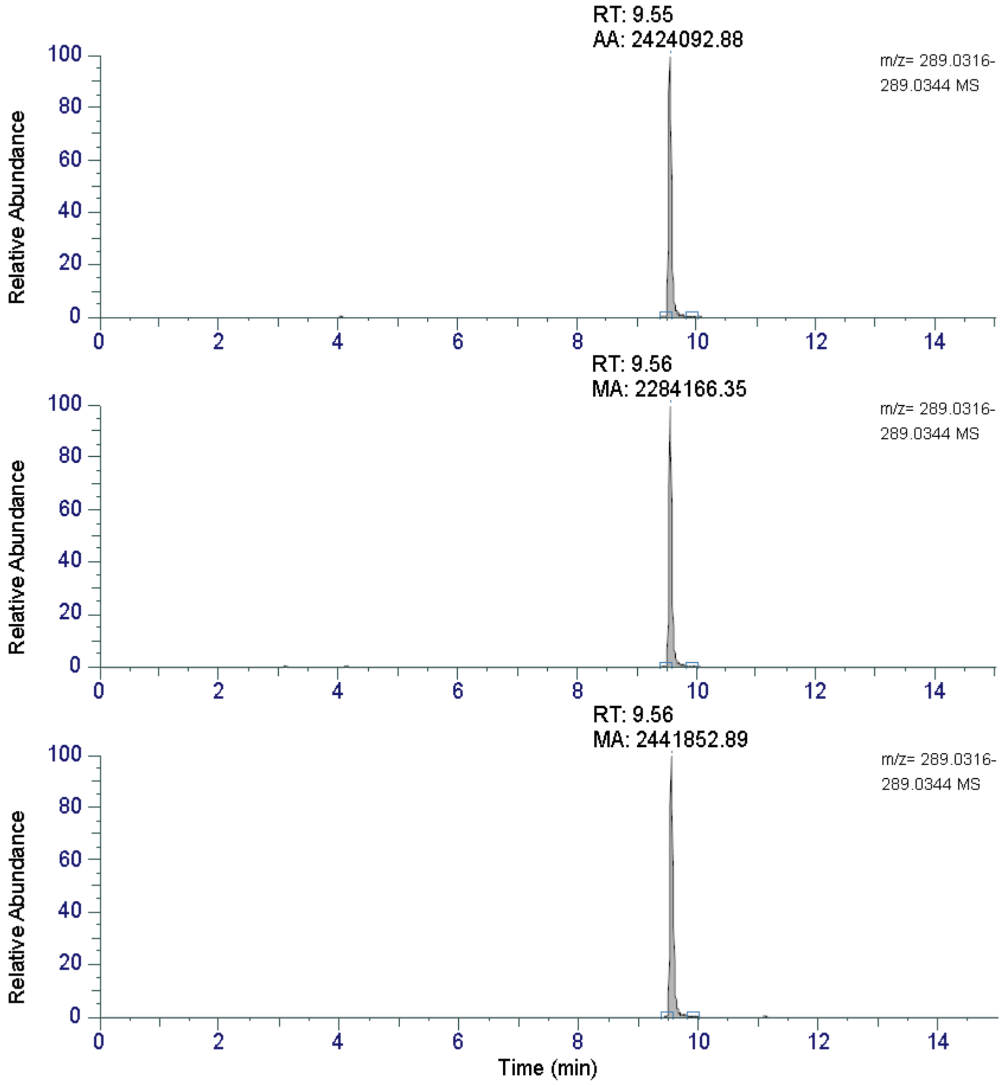

Figure S8: Extracted-ion chromatograms (*m/z* = 289.0330; mass tolerance = 5 ppm) of **sedo** triplicate control samples for TKT – stability assay (no TKT added). *m/z* calc. for $C_7H_{14}O_{10}P^-$ [M-H]$^-$: 289.0330 (**S7P**).

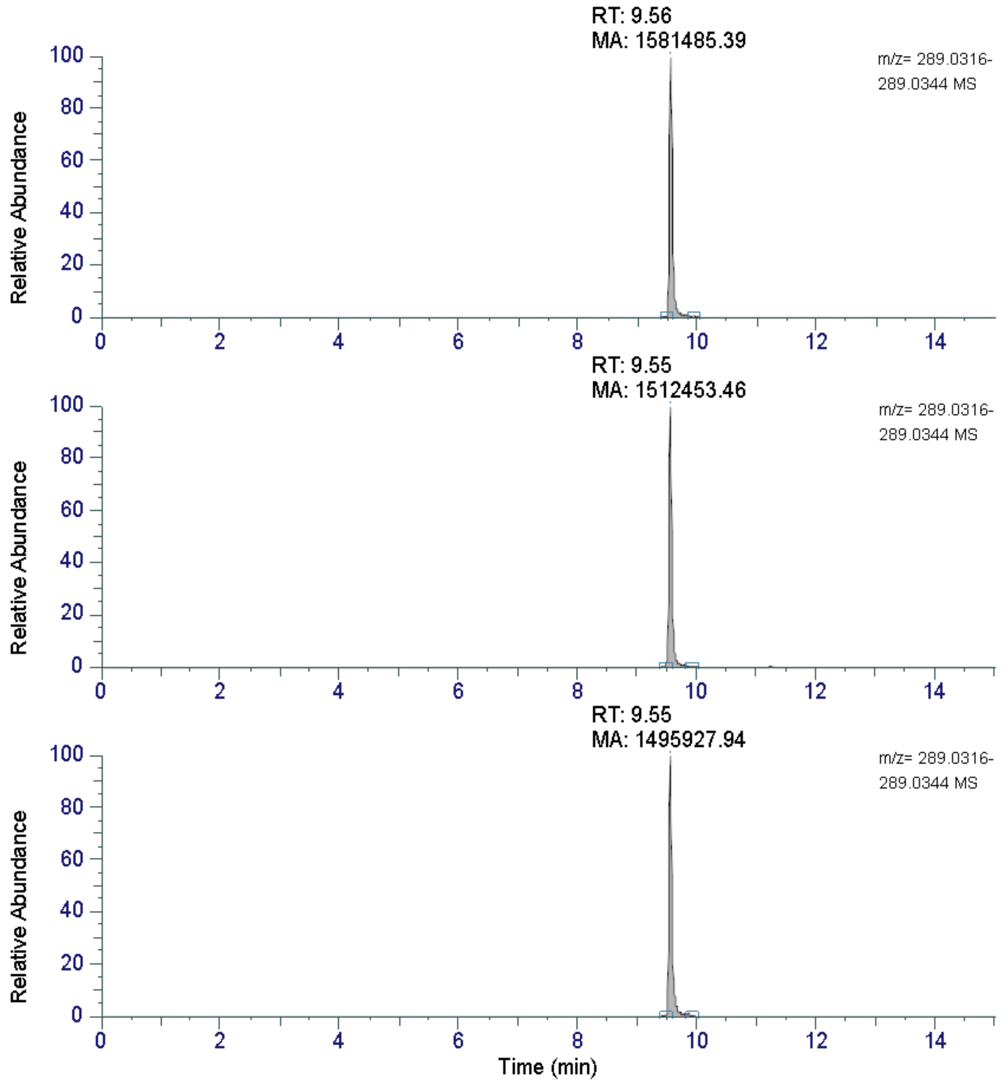

Figure S9: Extracted-ion chromatograms (*m/z* = 289.0330; mass tolerance = 5 ppm) of **sedo** triplicate samples for TKT – stability assay. *m/z* calc. for $C_7H_{14}O_{10}P^-$ [M-H]$^-$: 289.0330 (**S7P**).

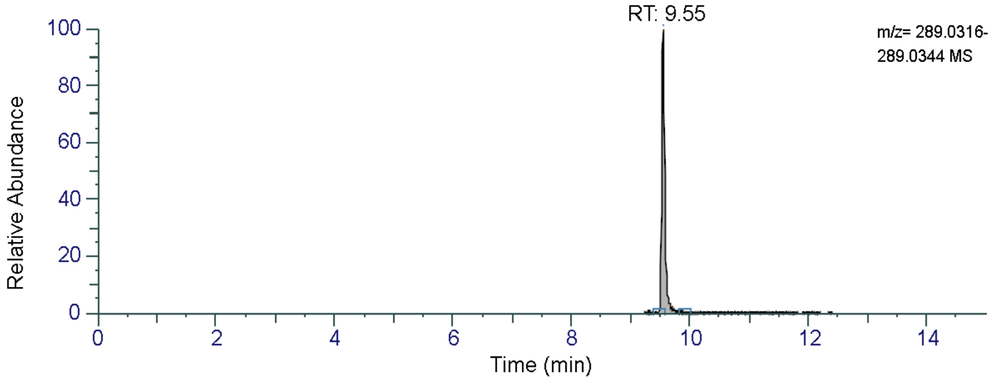

Figure S10: Extracted-ion chromatogram (*m/z* = 289.0330; mass tolerance = 5 ppm) of **S7P** standard (10 µM in MeCN/H_2O (1:1 v/v/). *m/z* calc. for $C_7H_{14}O_{10}P^-$ [M-H]$^-$: 289.0330 (**S7P**).

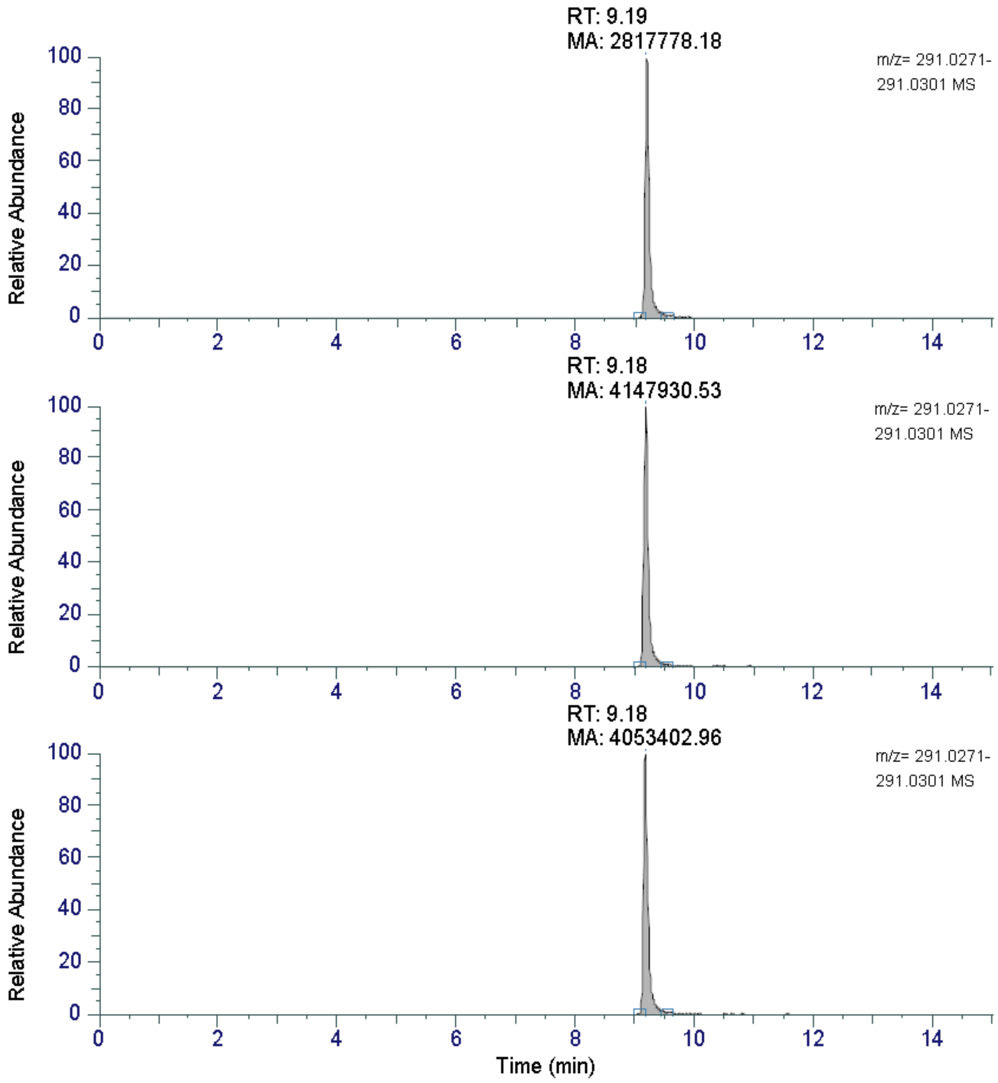

Figure S11: Extracted-ion chromatograms (*m/z* = 291.0286; mass tolerance = 5 ppm) of **4DFS** triplicate control samples for TKT – stability assay (no TKT added). *m/z* calc. for $C_7H_{13}FO_9P^-$ $[M-H]^-$: 291.0286 (**4DFS**-phosphate).

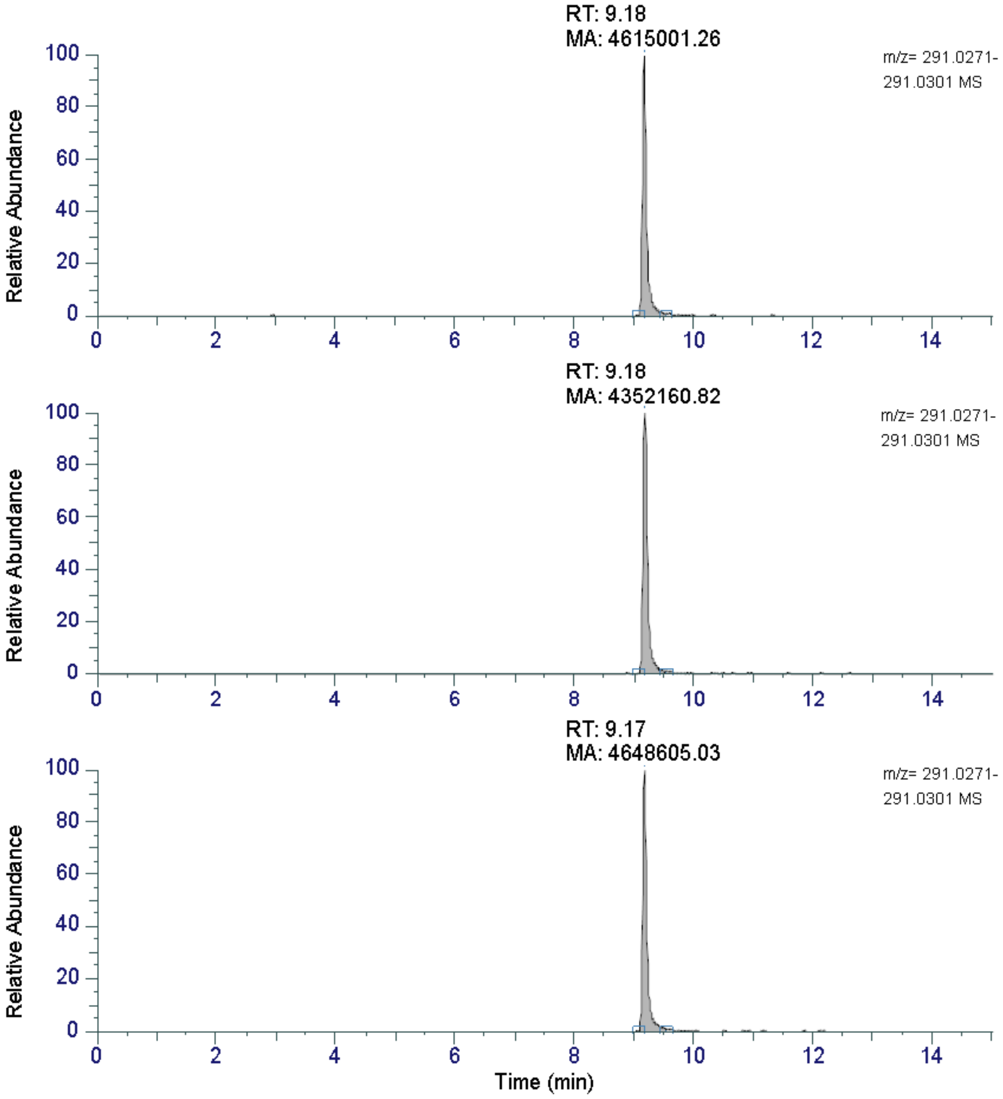

Figure S12: Extracted-ion chromatograms (*m/z* = 291.0286; mass tolerance = 5 ppm) of **4DFS** triplicate samples for TKT – stability assay. *m/z* calc. for $C_7H_{13}FO_9P^-$ $[M-H]^-$: 291.0286 (**4DFS**-phosphate).

3.2 NMR spectra

Pictures of the recorded ^{1}H, ^{13}C and ^{19}F NMR spectra of all synthetized compounds are available as additional material. The first spectrum shown in each series is the full ^{1}H NMR spectrum. Expansions are depicted where they were regarded as necessary. Then the ^{13}C NMR spectrum is depicted in the same manner, followed by the proton decoupled - and finally the proton coupled ^{19}F NMR spectrum. The x-axes and peak labels are is in ppm for all shown spectra. Structures are always given on top of the full ^{1}H NMR spectrum. Integrals are denoted below the x-axes where they are regarded as necessary and the integration range is marked. The numbering and order of compounds is in accordance with the numbering and order of substances in the main text.

3.2.1 1-(*p*-Toluenesulfonyl)imidazole (S1)

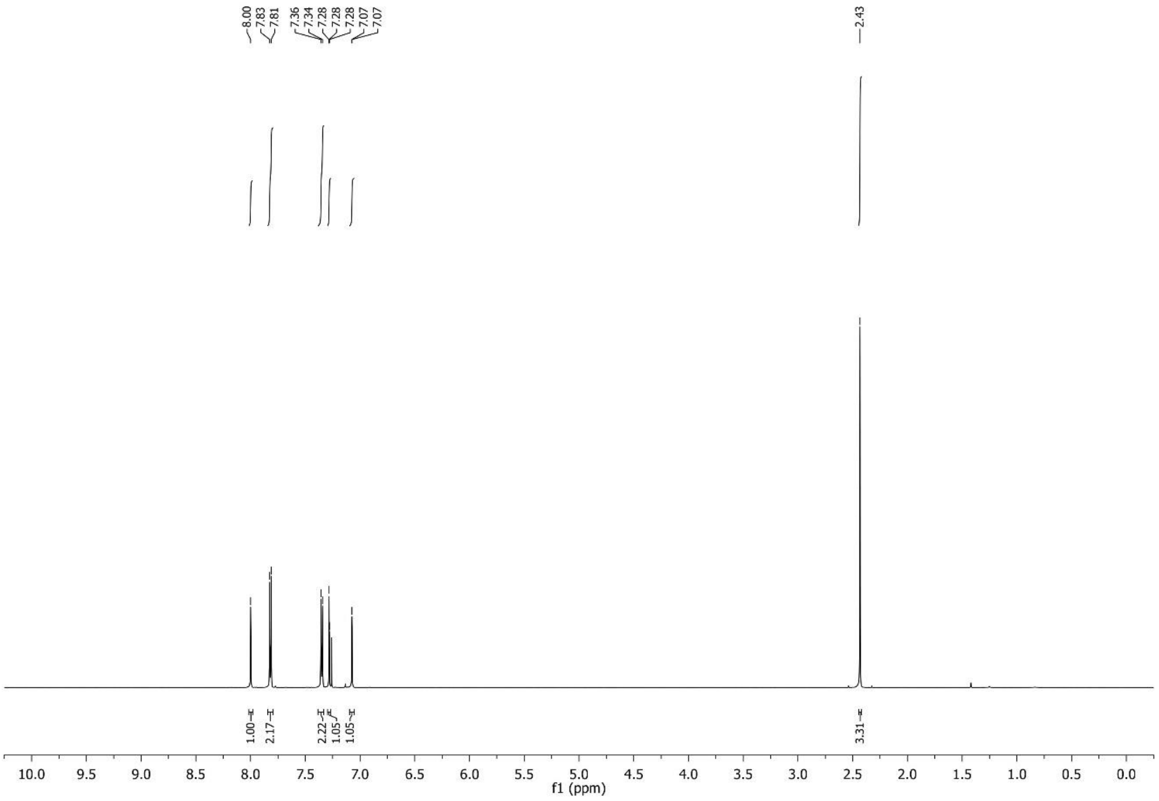

Figure S13: ^{1}H NMR (600 MHz, $CDCl_3$) of 1-(*p*-toluenesulfonyl)imidazole (**S1**).

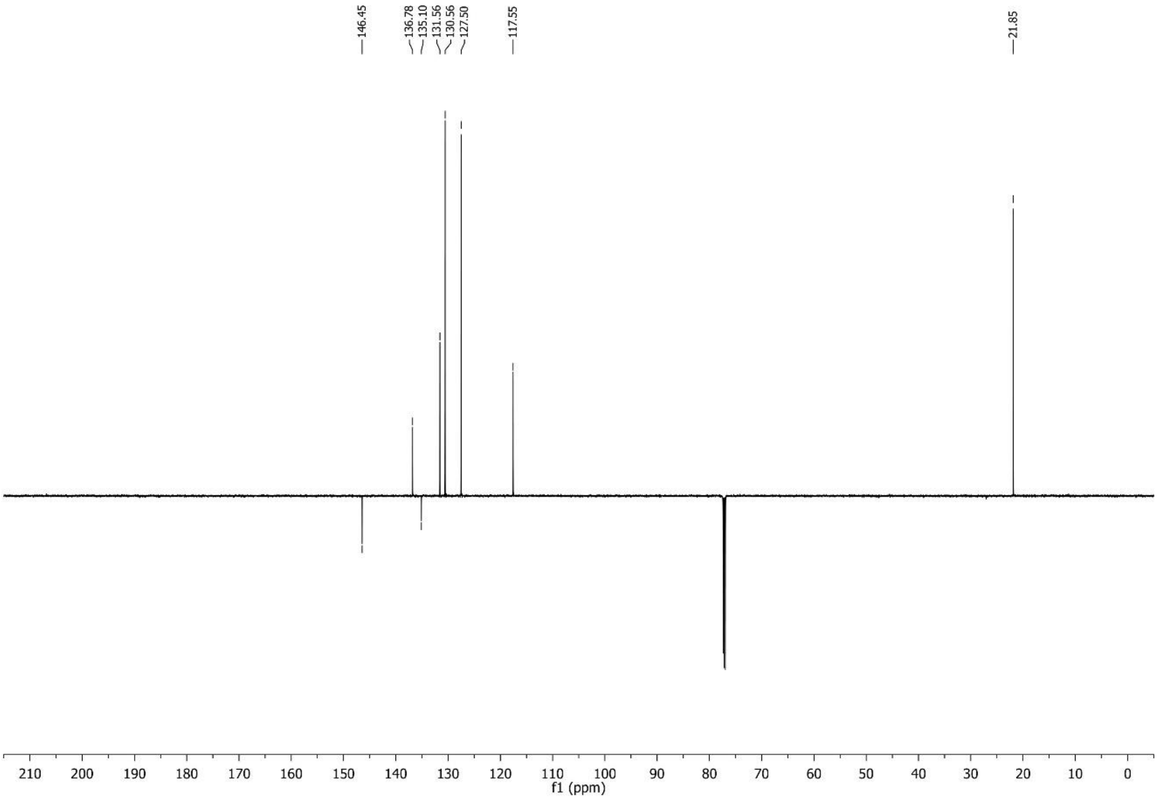

Figure S14: ^{13}C NMR (151 MHz, $CDCl_3$) of 1-(*p*-toluenesulfonyl)imidazole (**S1**).

3.2.2 Methyl 2,3-anhydro-4,6-*O*-benzylidene-α-D-*manno*-pyranoside (2)

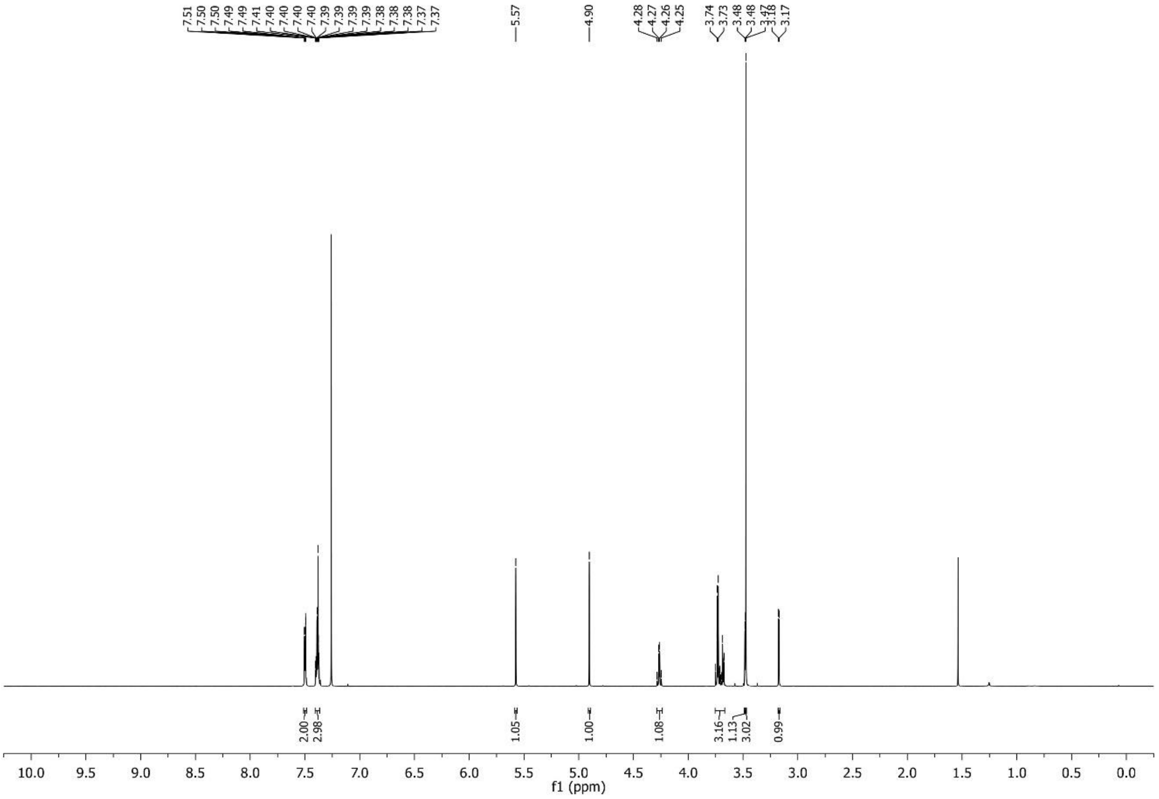

Figure S15: ^{1}H NMR (700 MHz, $CDCl_3$) of methyl 2,3-anhydro-4,6-*O*-benzylidene-α-D-*manno*-pyranoside (**2**).

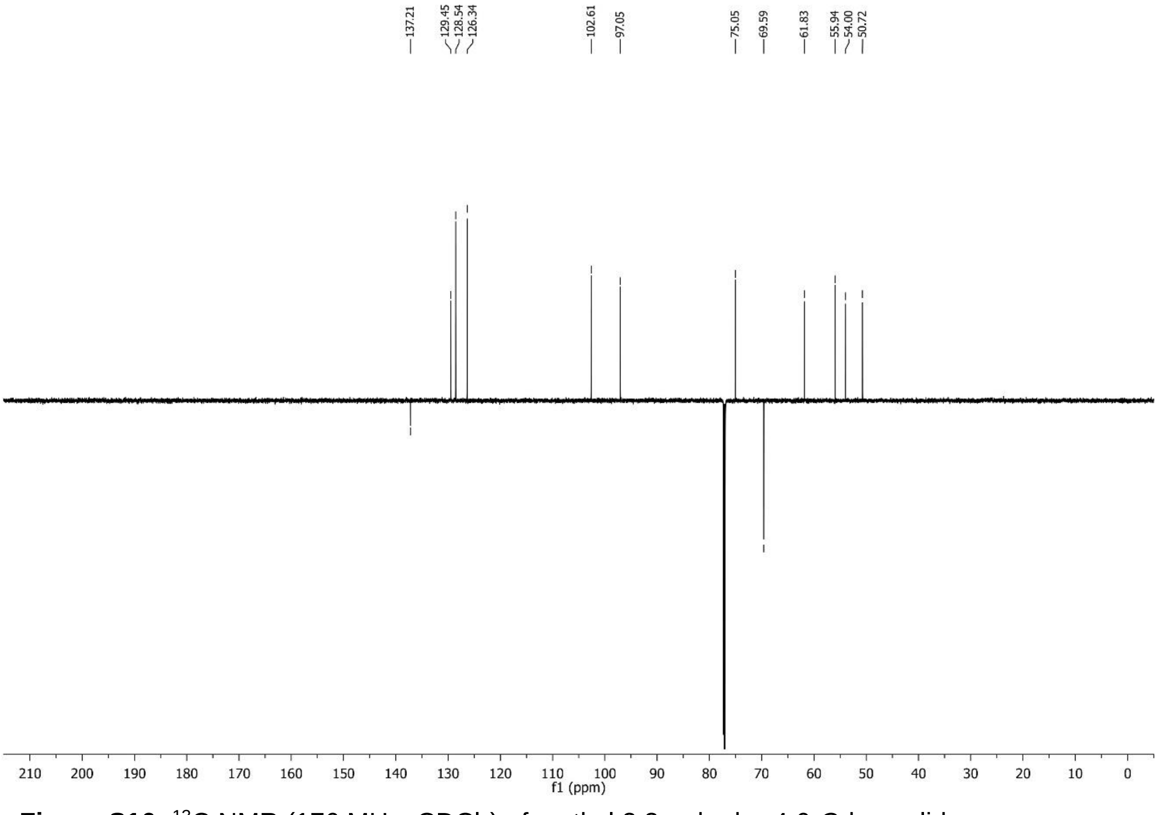

Figure S16: ^{13}C NMR (176 MHz, $CDCl_3$) of methyl 2,3-anhydro-4,6-*O*-benzylidene-α-D-*manno*-pyranoside (**2**).

3.2.3 Methyl 4,6-*O*-benzylidene-3-deoxy-3-fluoro-α-D-*altro*-pyranoside (3)

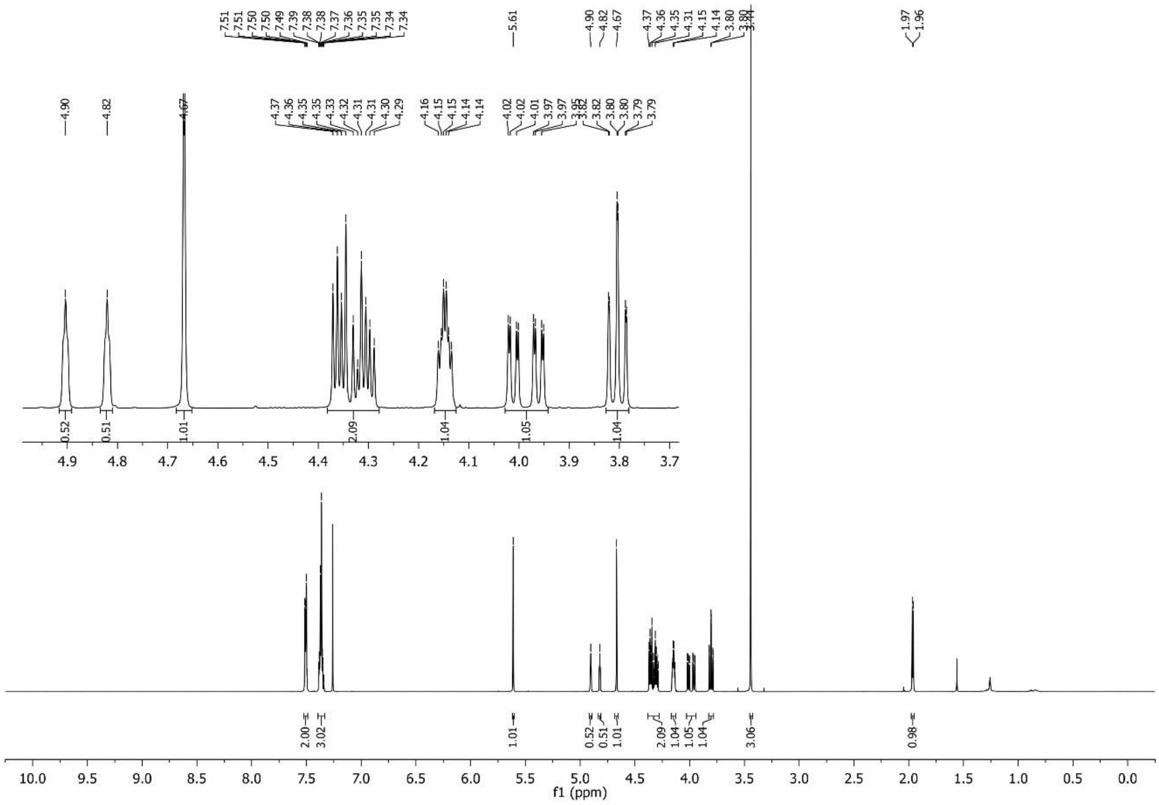

Figure S17: ^{1}H NMR (600 MHz, $CDCl_3$) of methyl 4,6-*O*-benzylidene-3-deoxy-3-fluoro-α-D-*altro*-pyranoside (**3**).

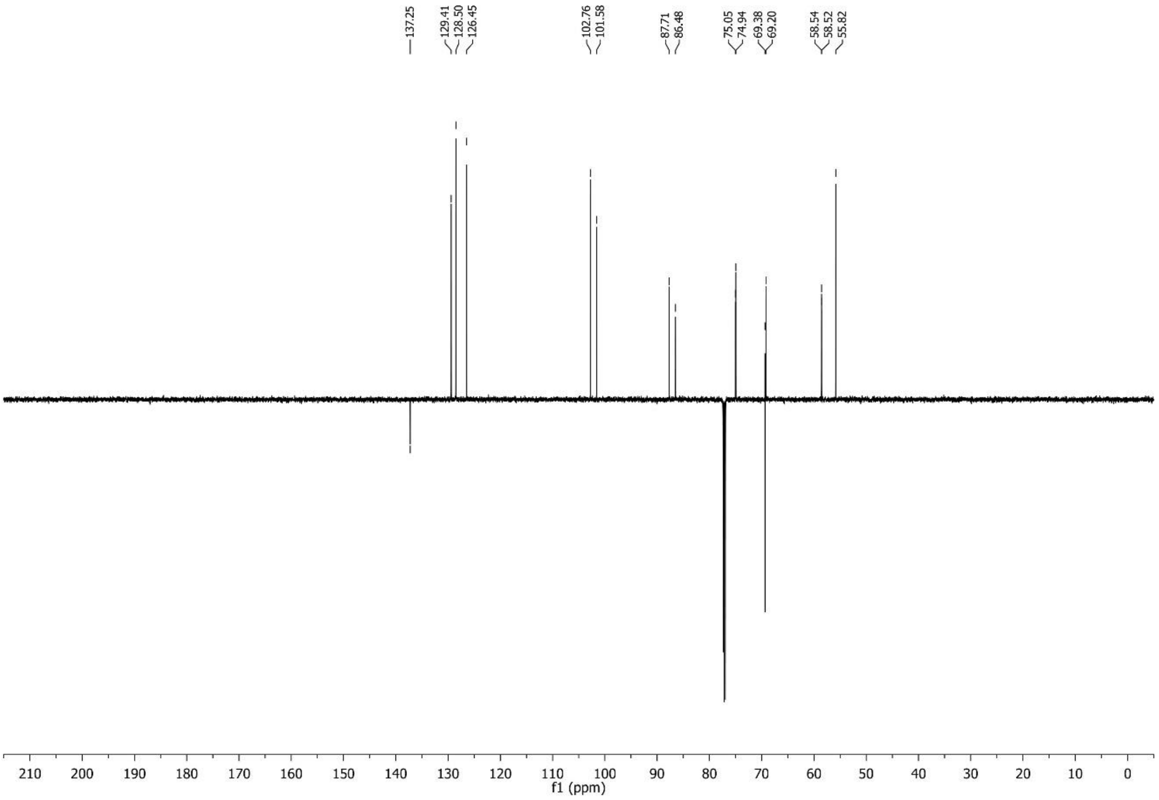

Figure S18: ^{13}C NMR (151 MHz, $CDCl_3$) of methyl 4,6-*O*-benzylidene-3-deoxy-3-fluoro-α-D-*altro*-pyranoside (**3**).

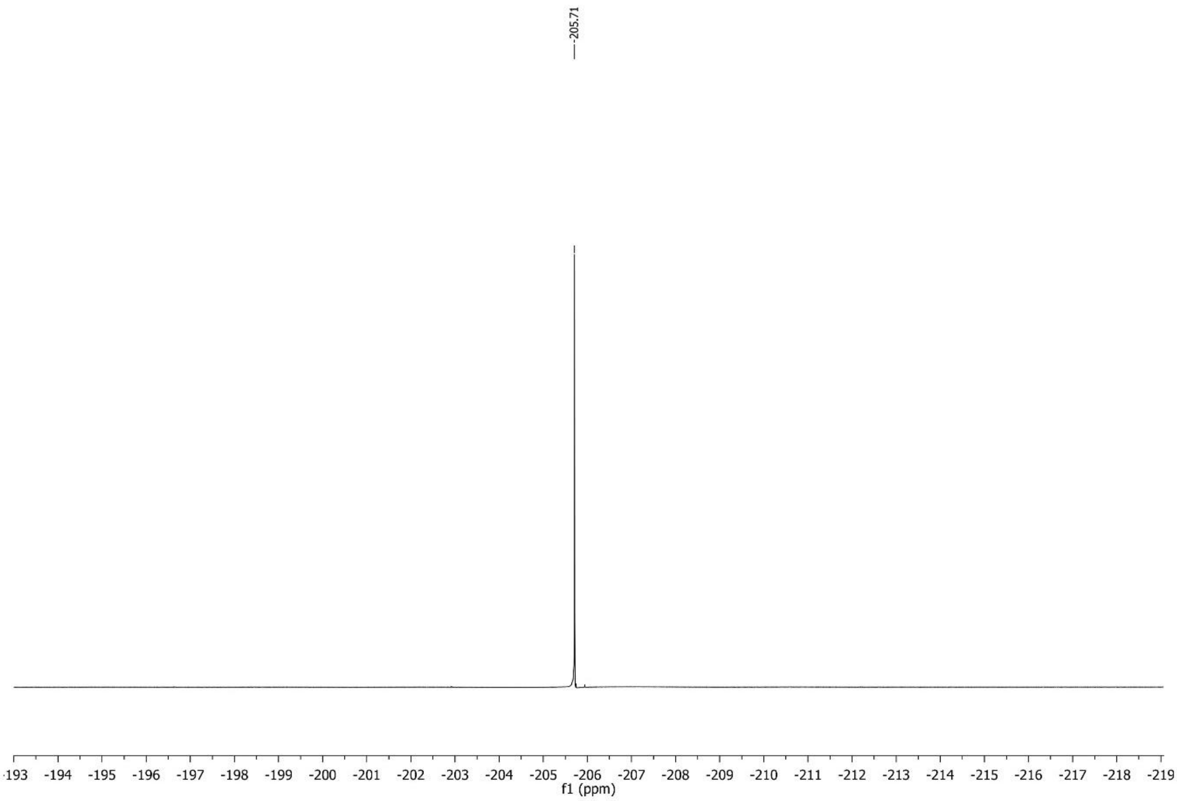

Figure S19: $^{19}F\{^{1}H\}$ NMR (659 MHz, $CDCl_3$) methyl 4,6-*O*-benzylidene-3-deoxy-3-fluoro-α-D-*altro*-pyranoside (**3**).

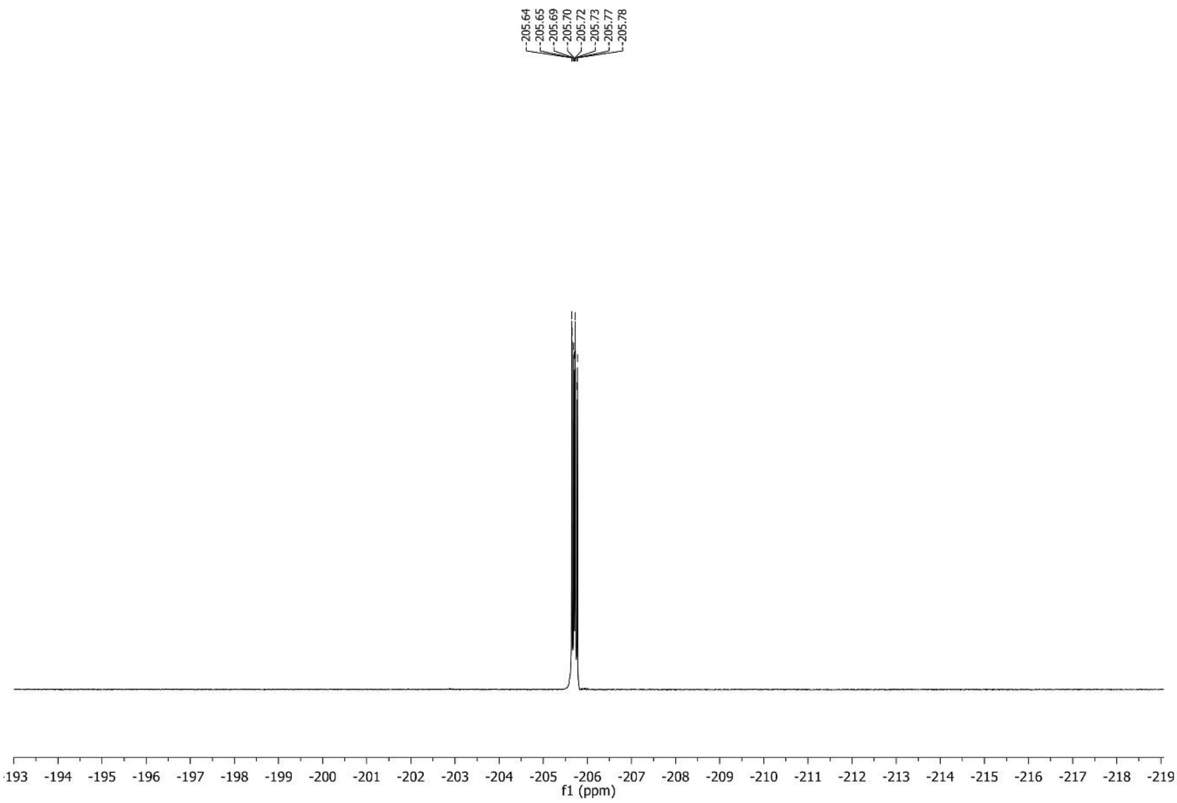

Figure S20: ^{19}F NMR (659 MHz, $CDCl_3$) of methyl 4,6-*O*-benzylidene-3-deoxy-3-fluoro-α-D-*altro*-pyranoside (**3**).

3.2.4 Methyl 4-*O*-benzyl-3-deoxy-3-fluoro-α-D-*altro*-pyranoside (4)

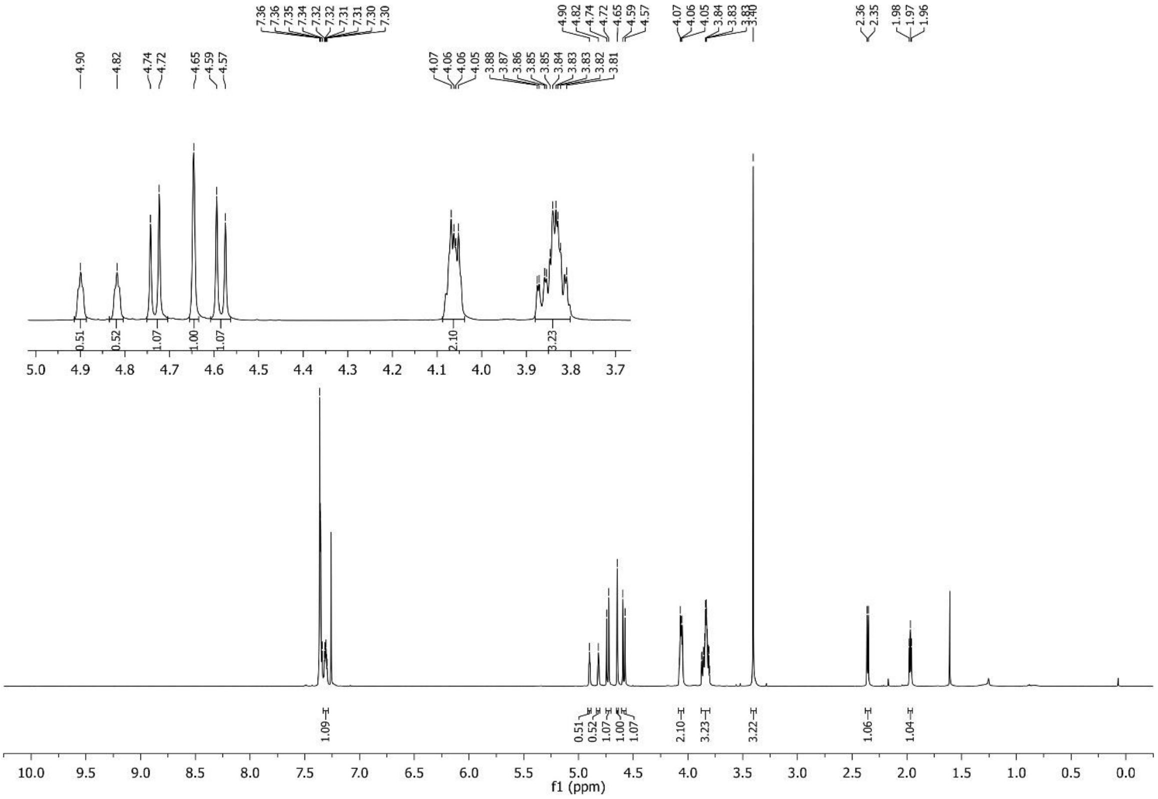

Figure S21: ^{1}H NMR (600 MHz, $CDCl_3$) of methyl 4-*O*-benzyl-3-deoxy-3-fluoro-α-D-*altro*-pyranoside (**4**).

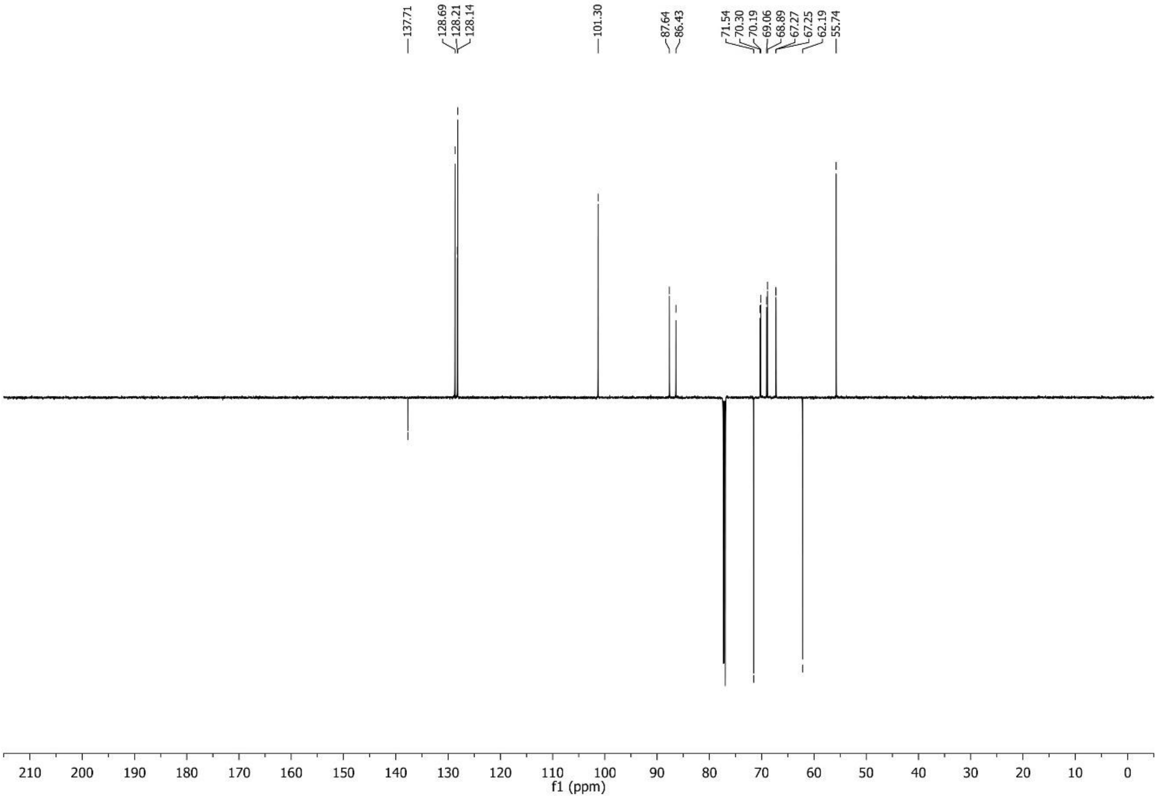

Figure S22: ^{13}C NMR (151 MHz, $CDCl_3$) of methyl 4-*O*-benzyl-3-deoxy-3-fluoro-α-D-*altro*-pyranoside (**4**).

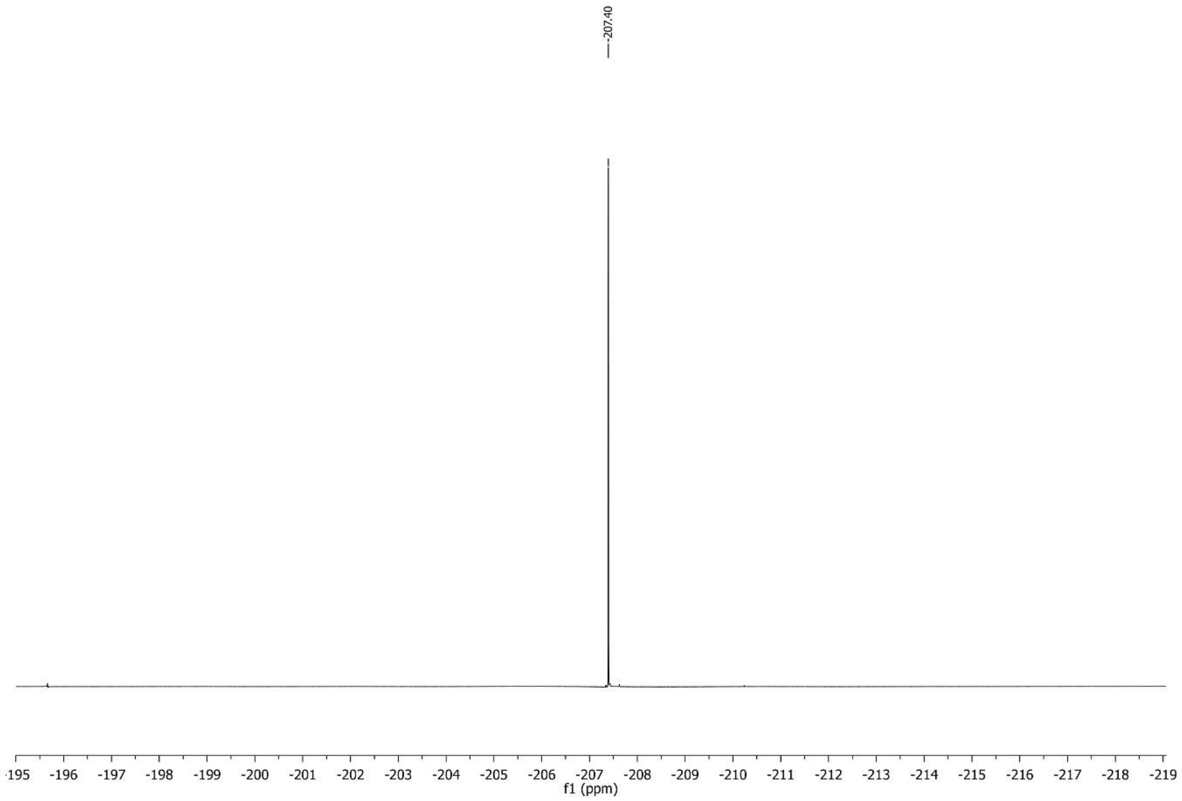

Figure S23: ^{19}F{^{1}H} NMR (659 MHz, $CDCl_3$) of of methyl 4-*O*-benzyl-3-deoxy-3-fluoro-α-D-*altro*-pyranoside (**4**).

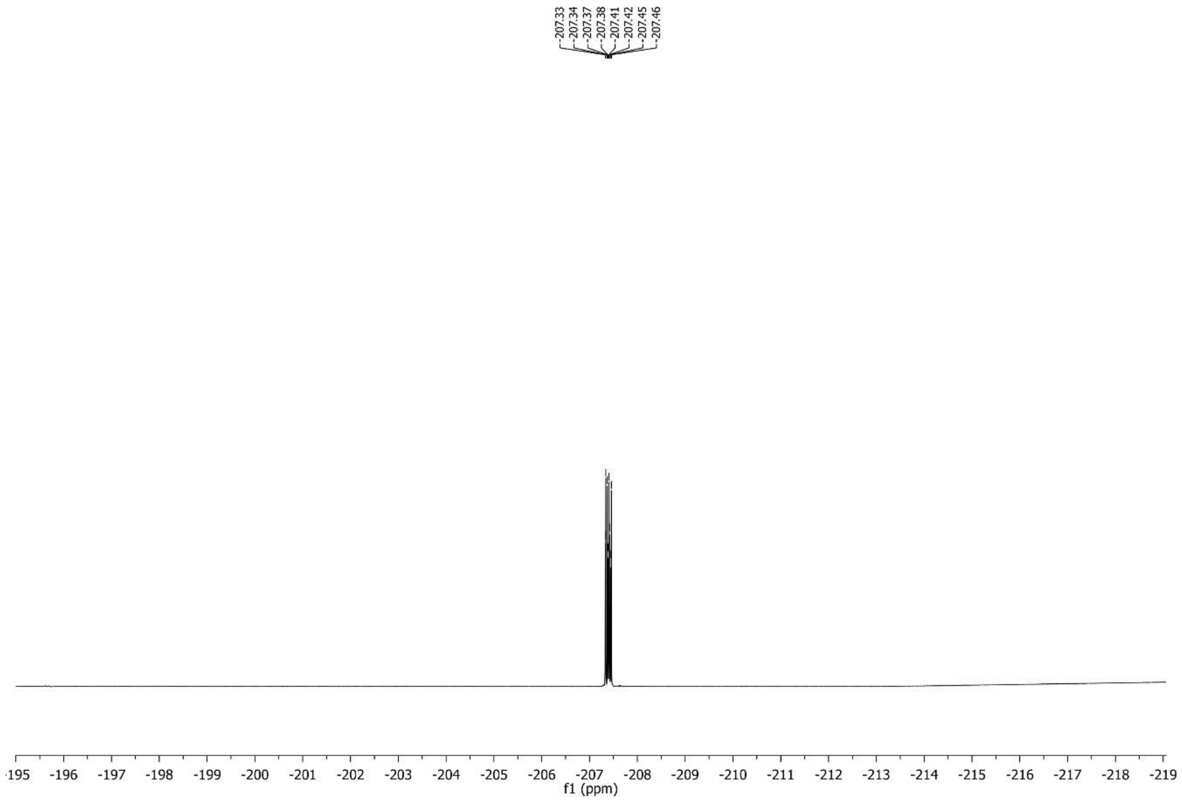

Figure S24: ^{19}F NMR (659 MHz, $CDCl_3$) of methyl 4-*O*-benzyl-3-deoxy-3-fluoro-α-D-*altro*-pyranoside (**4**).

3.2.5 Methyl 2,4,6-tri-*O*-benzyl-3-deoxy-3-fluoro-α-D-*altro*-pyranoside (5)

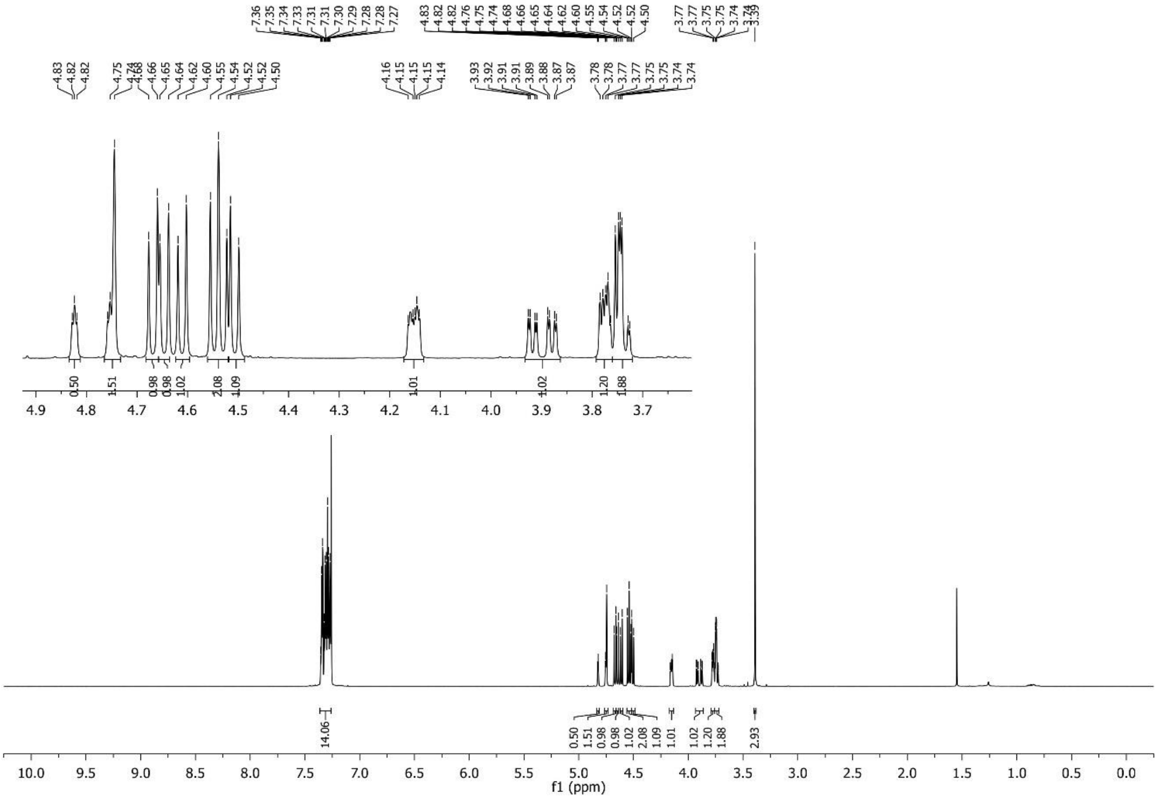

Figure S25: ^{1}H NMR (700 MHz, $CDCl_3$) of methyl 2,4,6-tri-*O*-benzyl-3-deoxy-3-fluoro-α-D-*altro*-pyranoside (**5**).

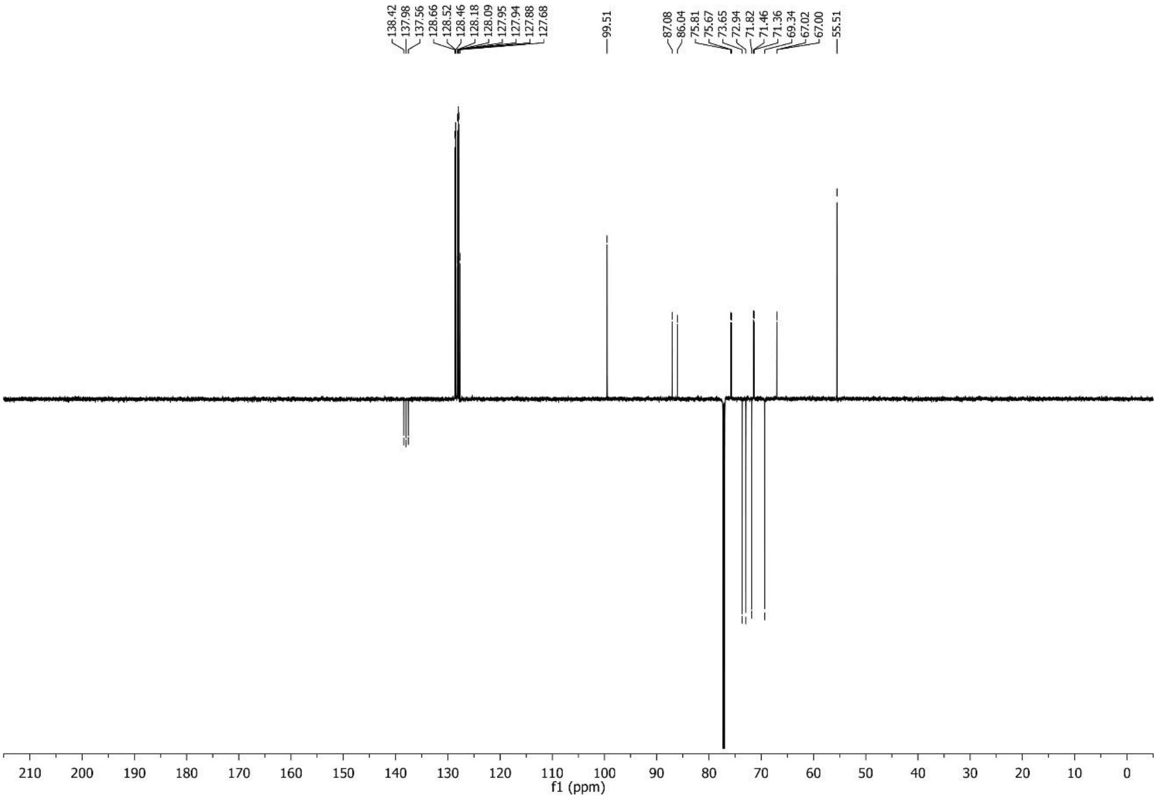

Figure S26: ^{13}C NMR (176 MHz, $CDCl_3$) of methyl 2,4,6-tri-*O*-benzyl-3-deoxy-3-fluoro-α-D-*altro*-pyranoside (**5**).

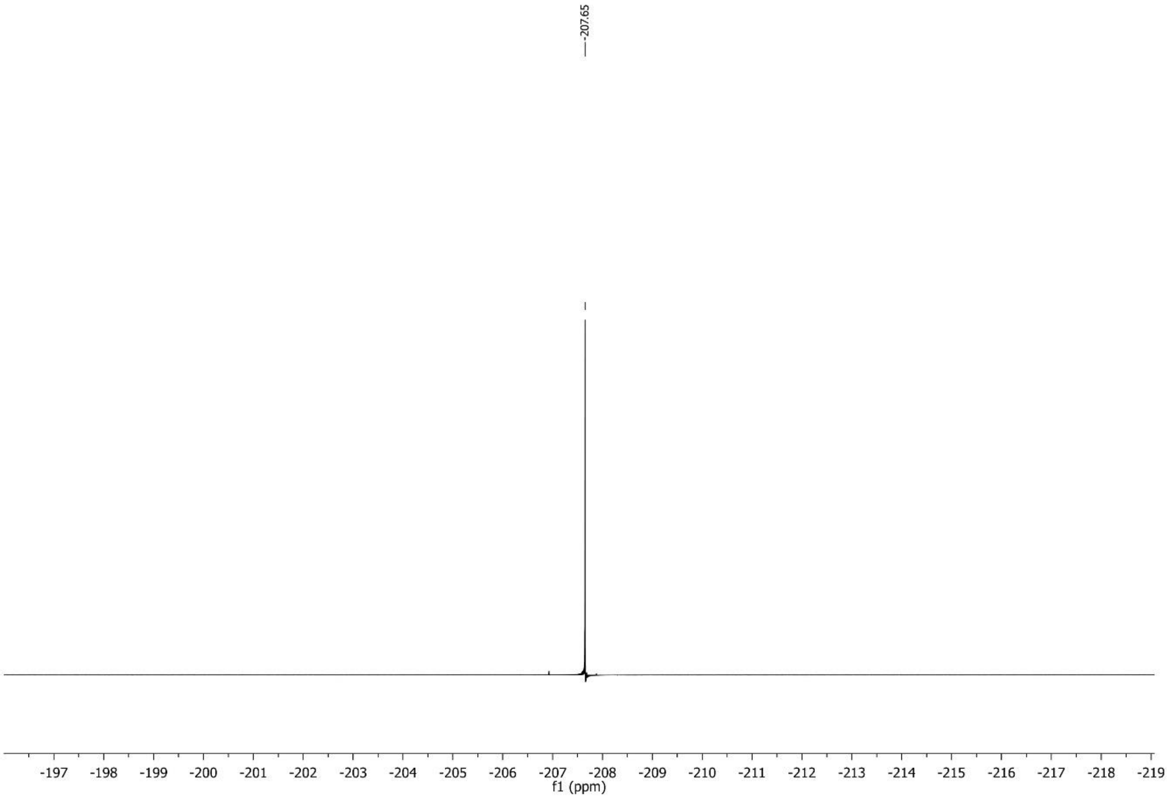

Figure S27: $^{19}F\{^{1}H\}$ NMR (659 MHz, $CDCl_3$) of methyl 2,4,6-tri-*O*-benzyl-3-deoxy-3-fluoro-α-D-*altro*-pyranoside (**5**).

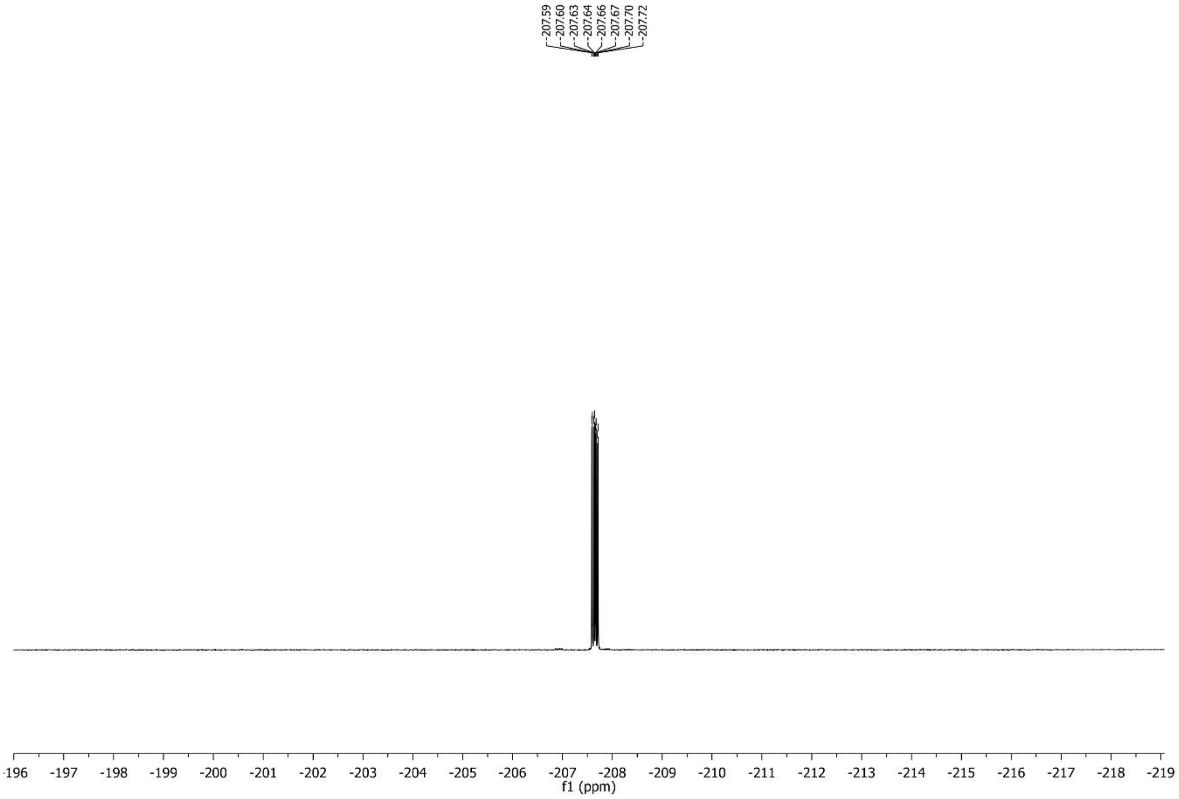

Figure S28: ^{19}F NMR (659 MHz, $CDCl_3$) of methyl 2,4,6-tri-*O*-benzyl-3-deoxy-3-fluoro-α-D-*altro*-pyranoside (**5**).

3.2.6 2,4,6-Tri-*O*-benzyl-3-deoxy-3-fluoro-D-altrose (6)

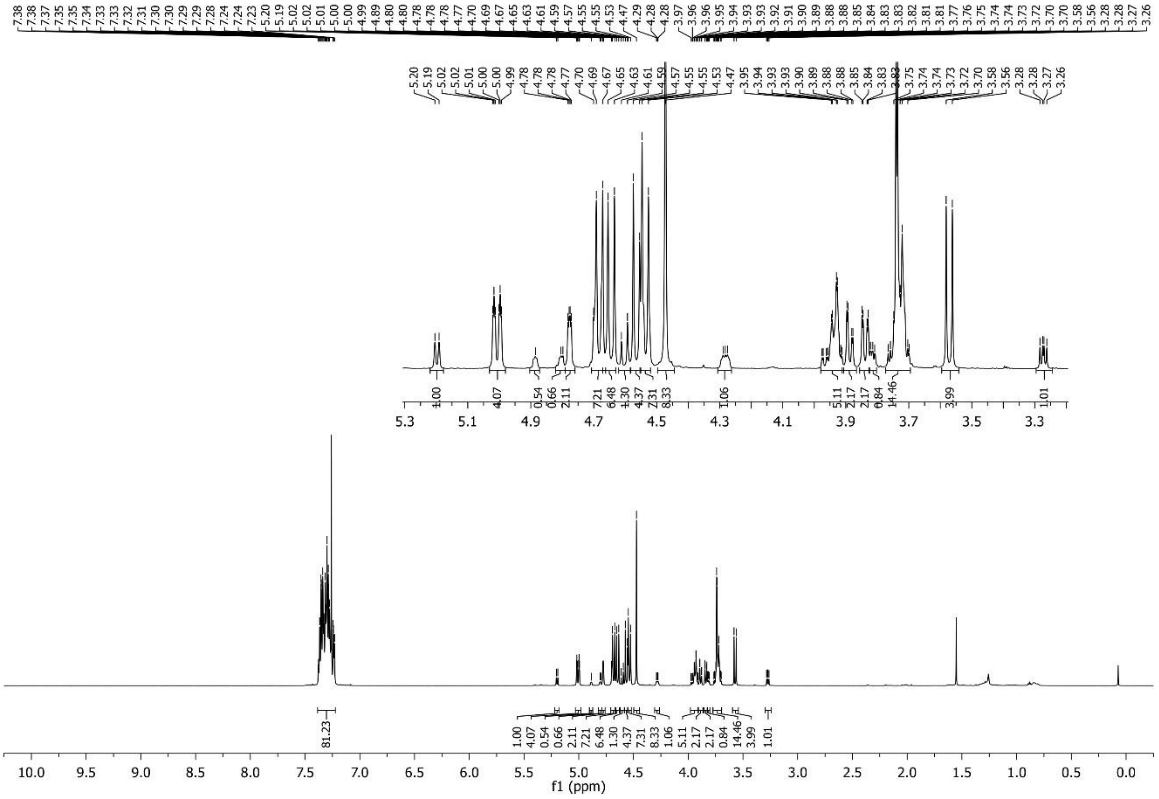

Figure S29: ^{1}H NMR (600 MHz, $CDCl_3$) of 2,4,6-tri-*O*-benzyl-3-deoxy-3-fluoro-D-altrose (**6**).

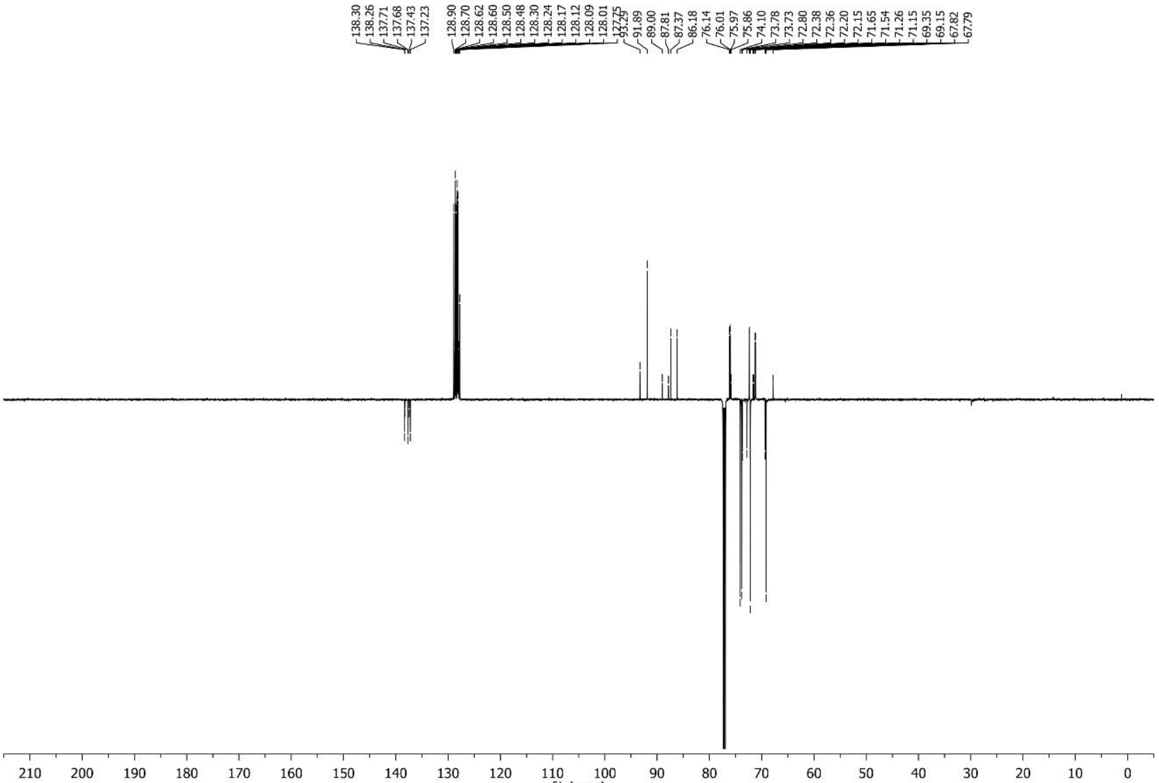

Figure S30: ^{13}C NMR (151 MHz, $CDCl_3$) of 2,4,6-tri-*O*-benzyl-3-deoxy-3-fluoro-D-altrose (**6**).

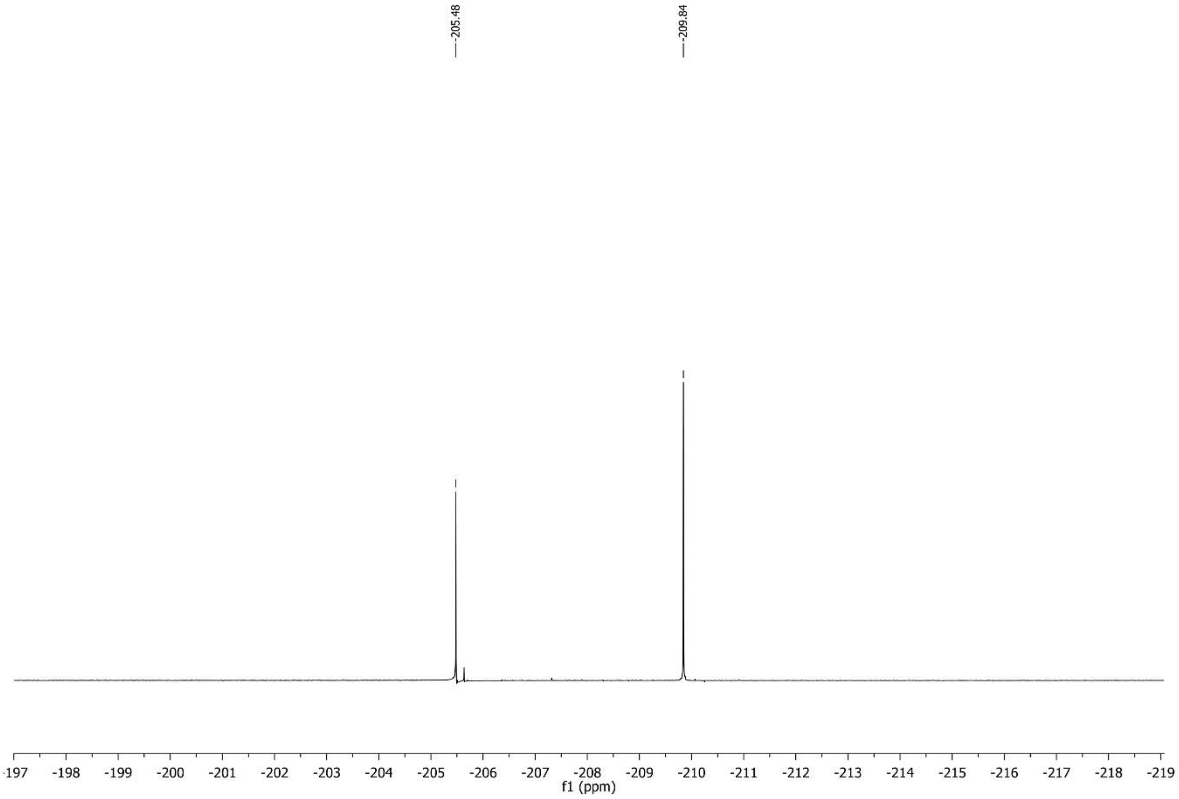

Figure S31: $^{19}F\{^{1}H\}$ NMR (659 MHz, $CDCl_3$) of 2,4,6-tri-*O*-benzyl-3-deoxy-3-fluoro-D-altrose (**6**).

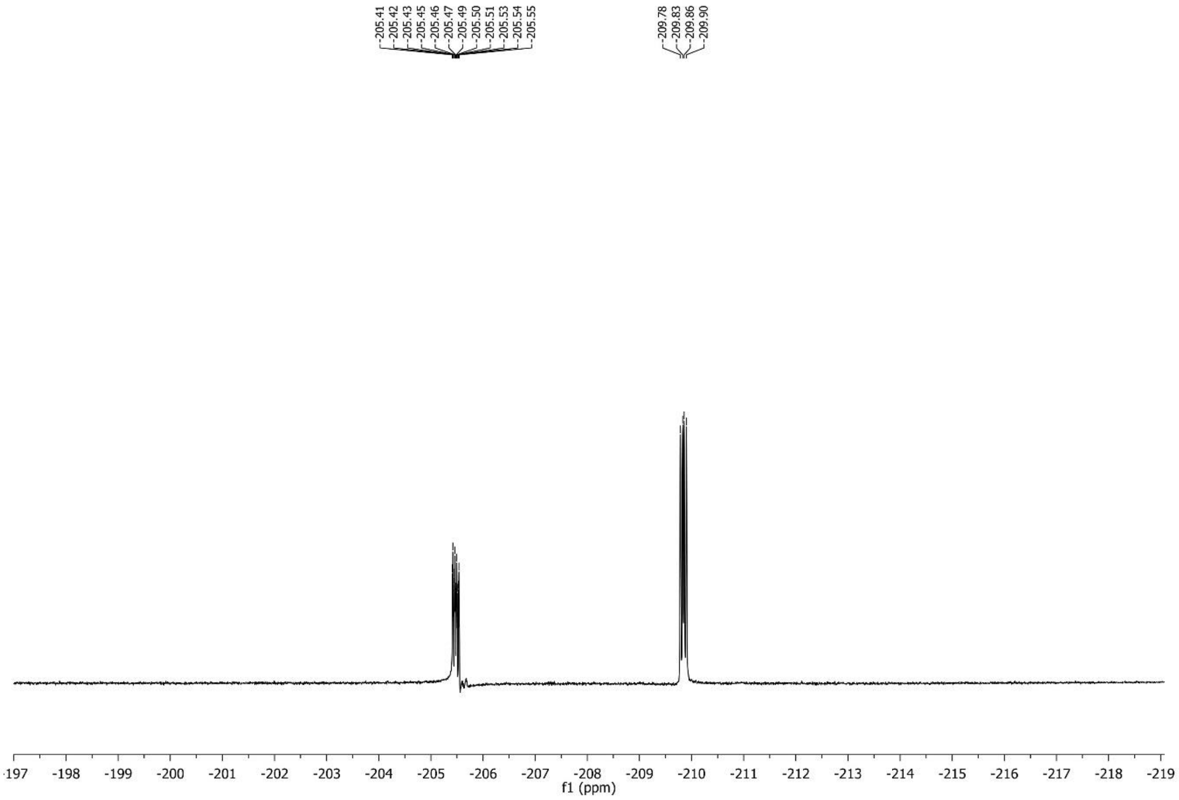

Figure S32: ^{19}F NMR (659 MHz, $CDCl_3$) of 2,4,6-tri-*O*-benzyl-3-deoxy-3-fluoro-D-altrose (**6**).

3.2.7 2,4,6-Tri-*O*-benzyl-3-deoxy-3-fluoro-D-*altrono*-1,5-lactone (7)

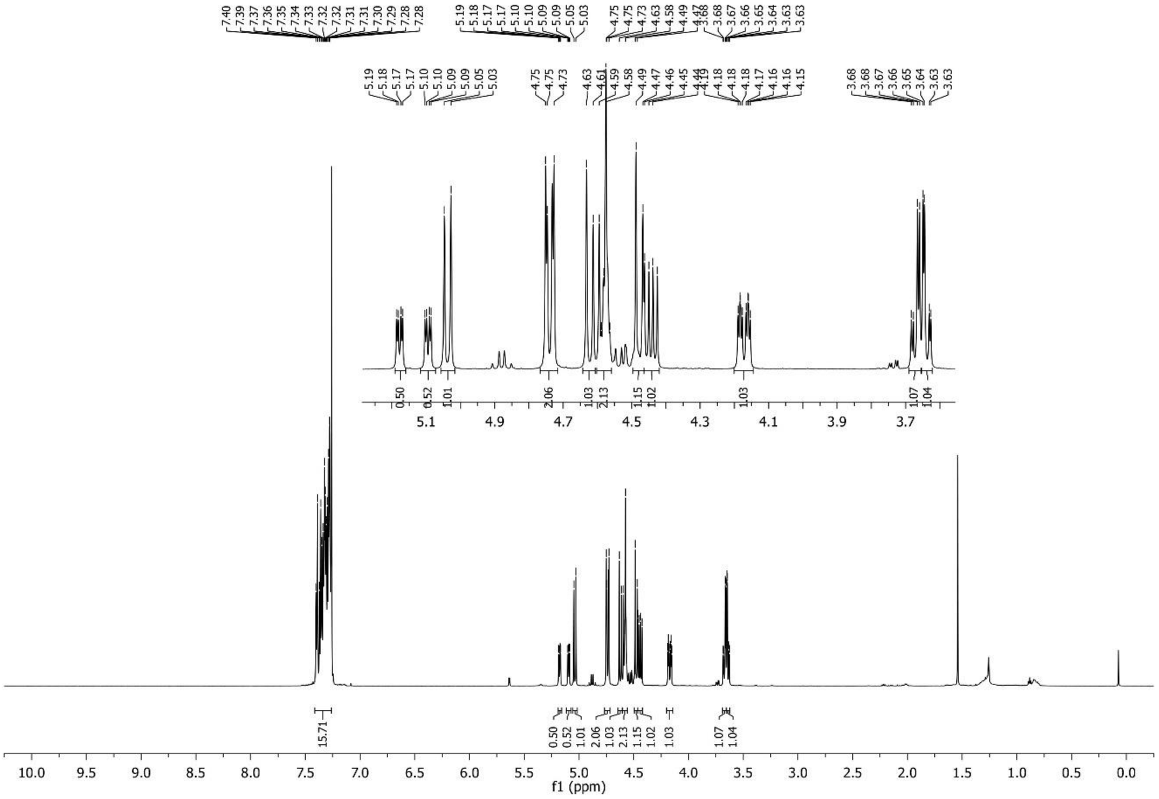

Figure S33: ^{1}H NMR (600 MHz, $CDCl_3$) of 2,4,6-tri-*O*-benzyl-3-deoxy-3-fluoro-D-*altrono*-1,5-lactone (**7**).

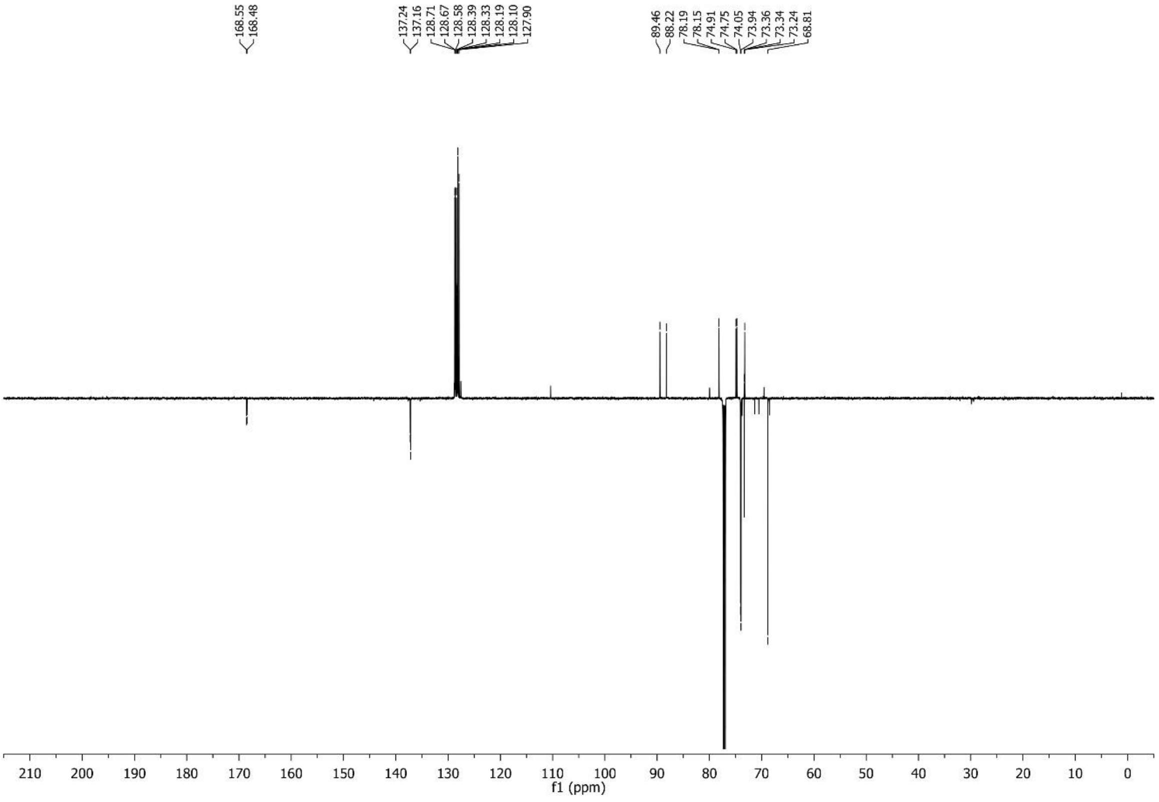

Figure S34: ^{13}C NMR (151 MHz, $CDCl_3$) of 2,4,6-tri-*O*-benzyl-3-deoxy-3-fluoro-D-*altrono*-1,5-lactone (**7**).

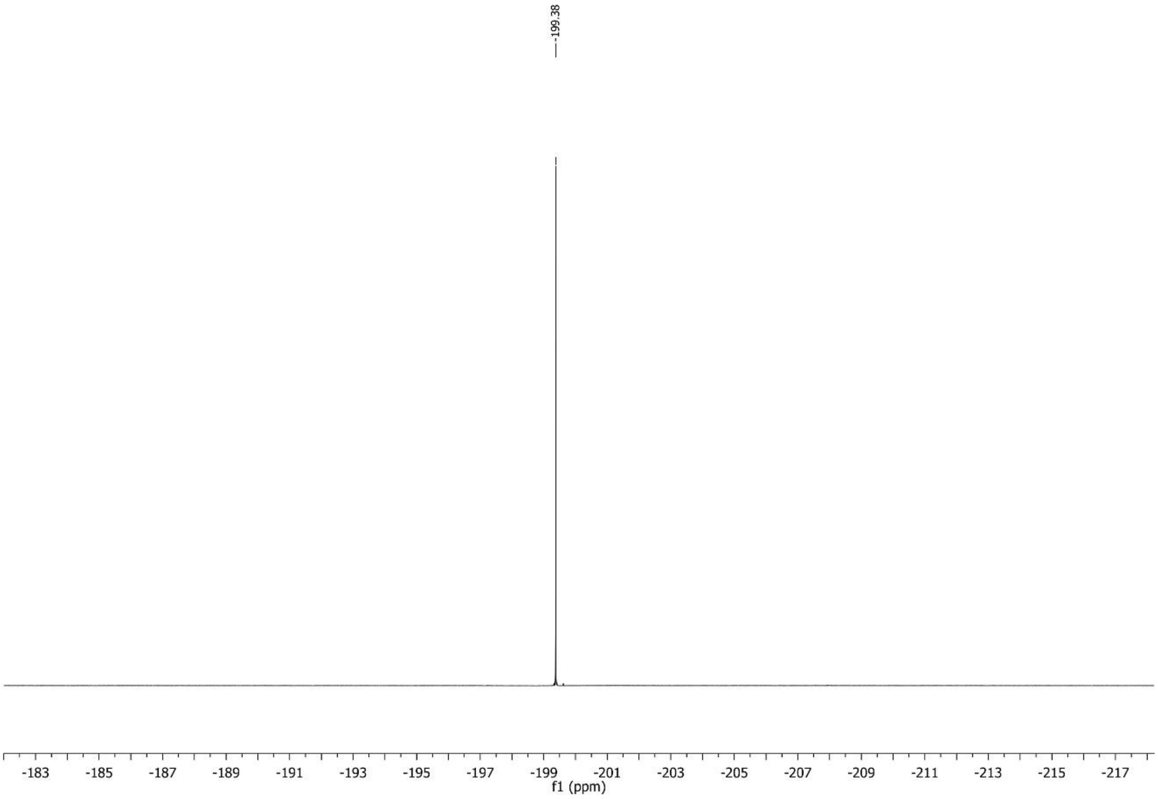

Figure S35: $^{19}F\{^{1}H\}$ NMR (659 MHz, $CDCl_3$) of 2,4,6-tri-*O*-benzyl-3-deoxy-3-fluoro-D-*altrono*-1,5-lactone (**7**).

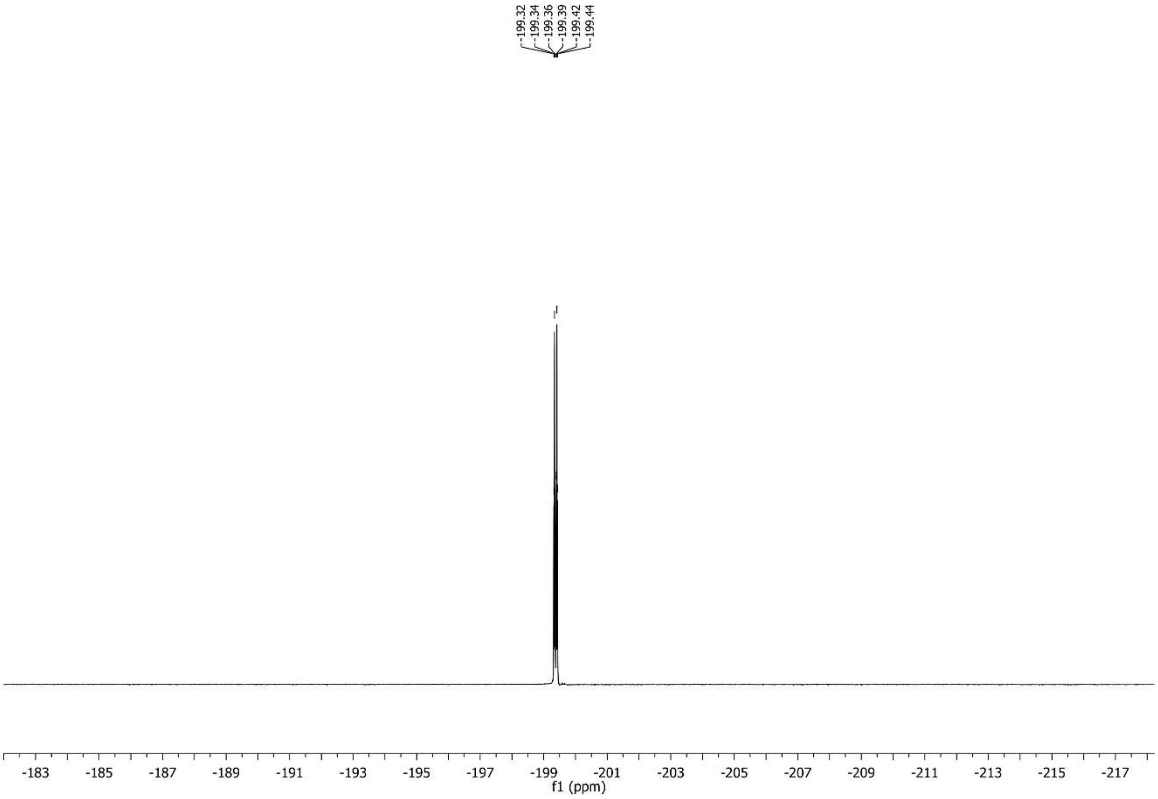

Figure S36: ^{19}F NMR (659 MHz, $CDCl_3$) of 2,4,6-tri-*O*-benzyl-3-deoxy-3-fluoro-D-*altrono*-1,5-lactone (**7**).

3.2.8 Bis(cyclopentadienyl)dimethyltitanium/Petasis' reagent (S2)

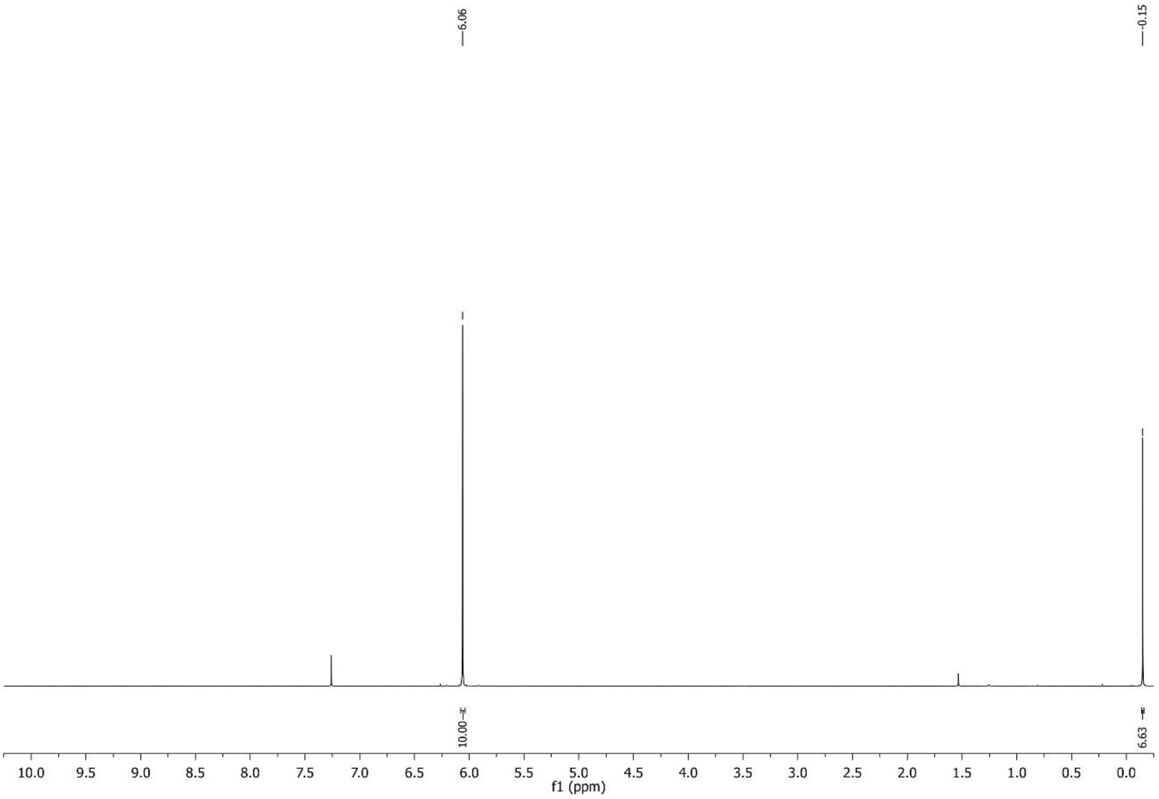

Figure S37: ^{1}H NMR (600 MHz, $CDCl_3$) of bis(cyclopentadienyl)dimethyltitanium/Petasis' reagent (**S2**).

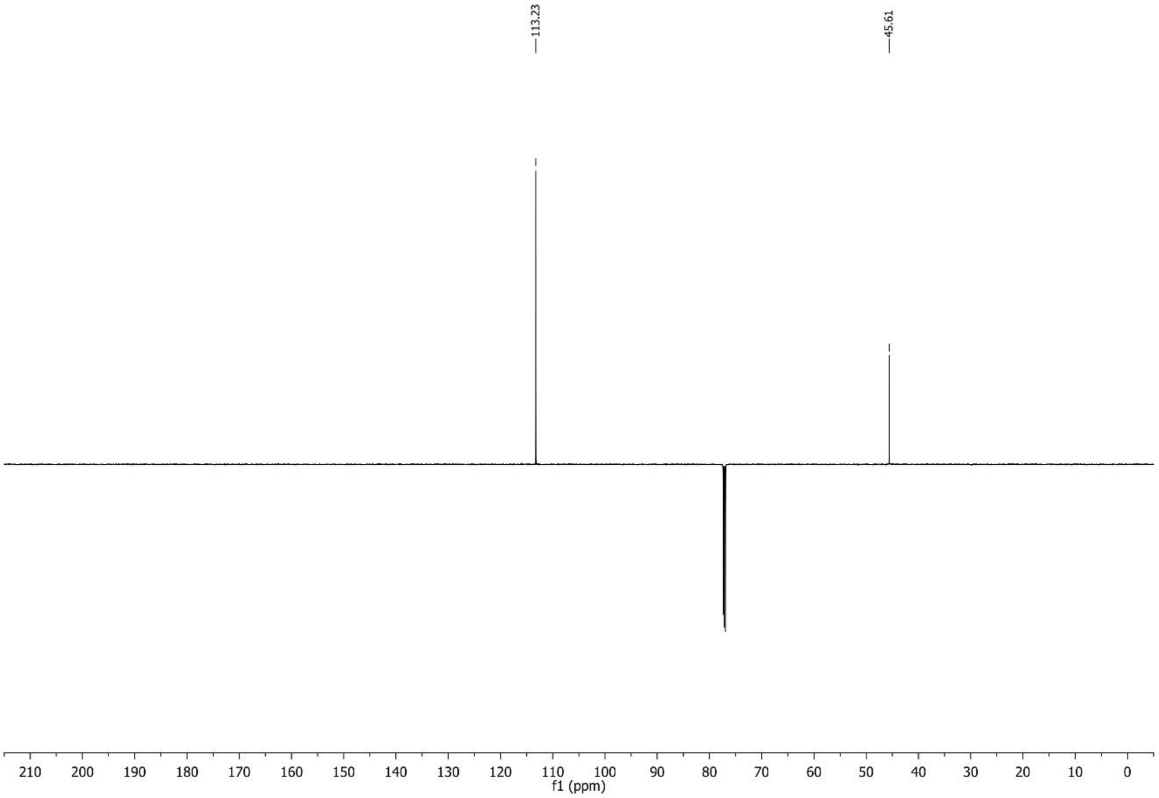

Figure S38: ^{13}C NMR (151 MHz, $CDCl_3$) of bis(cyclopentadienyl)dimethyltitanium/Petasis' reagent (**S2**).

3.2.9 2,6-Anhydro-3,5,7-tri-*O*-benzyl-1,4-dideoxy-4-fluoro-D-*altro*-hept-1-enitol (8)

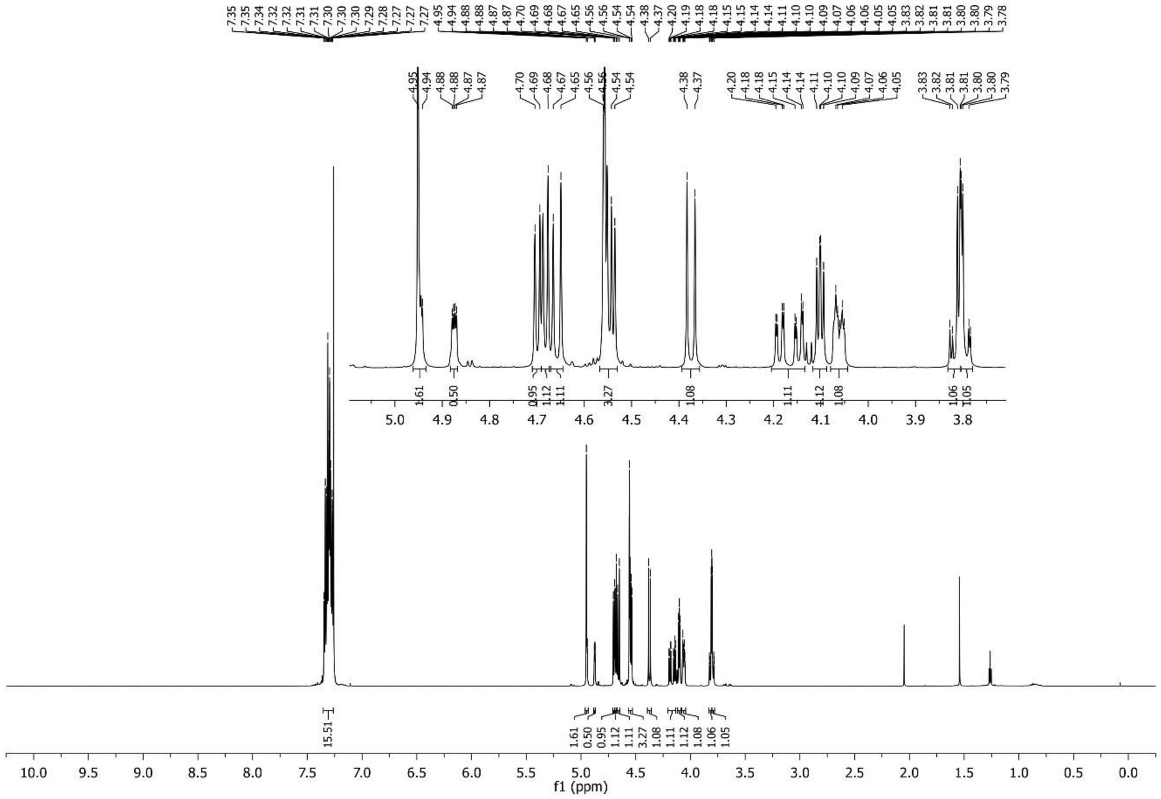

Figure S39: ^{1}H NMR (700 MHz, $CDCl_3$) of 2,6-anhydro-3,5,7-tri-*O*-benzyl-1,4-dideoxy-4-fluoro-D-*altro*-hept-1-enitol (**8**).

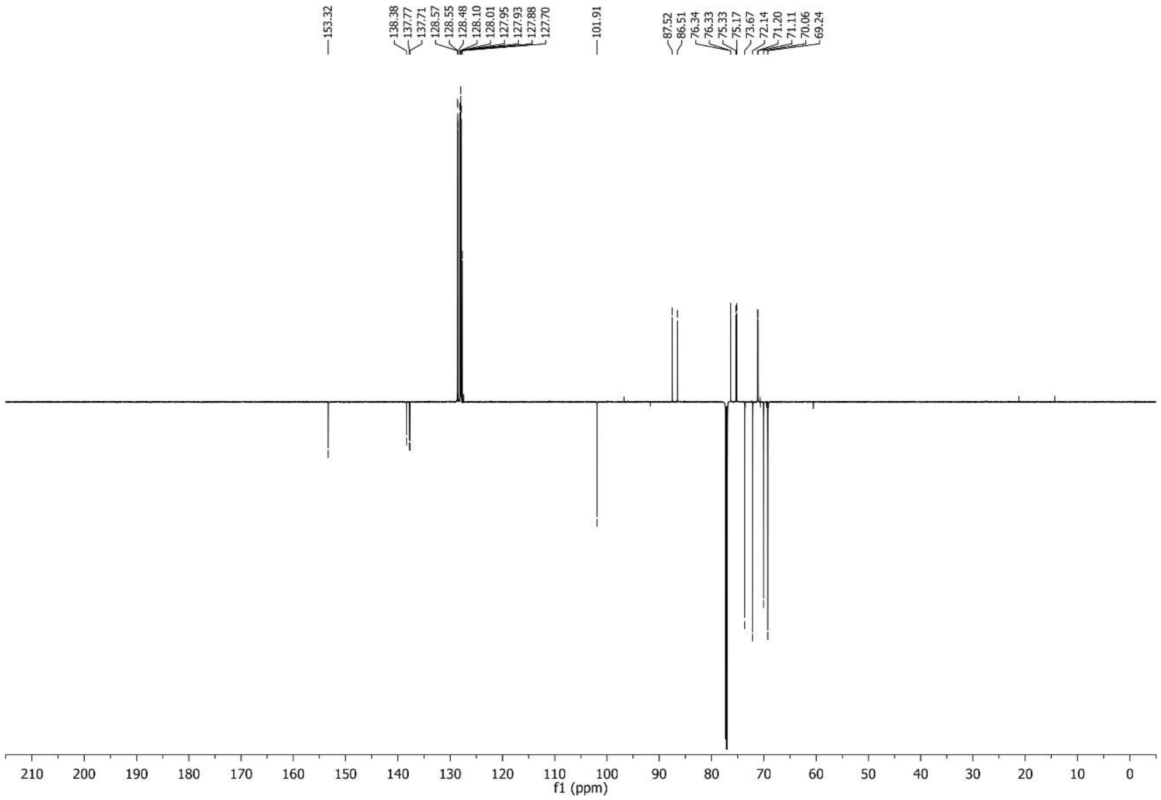

Figure S40: ^{13}C NMR (176 MHz, $CDCl_3$) of 2,6-anhydro-3,5,7-tri-*O*-benzyl-1,4-dideoxy-4-fluoro-D-*altro*-hept-1-enitol (**8**).

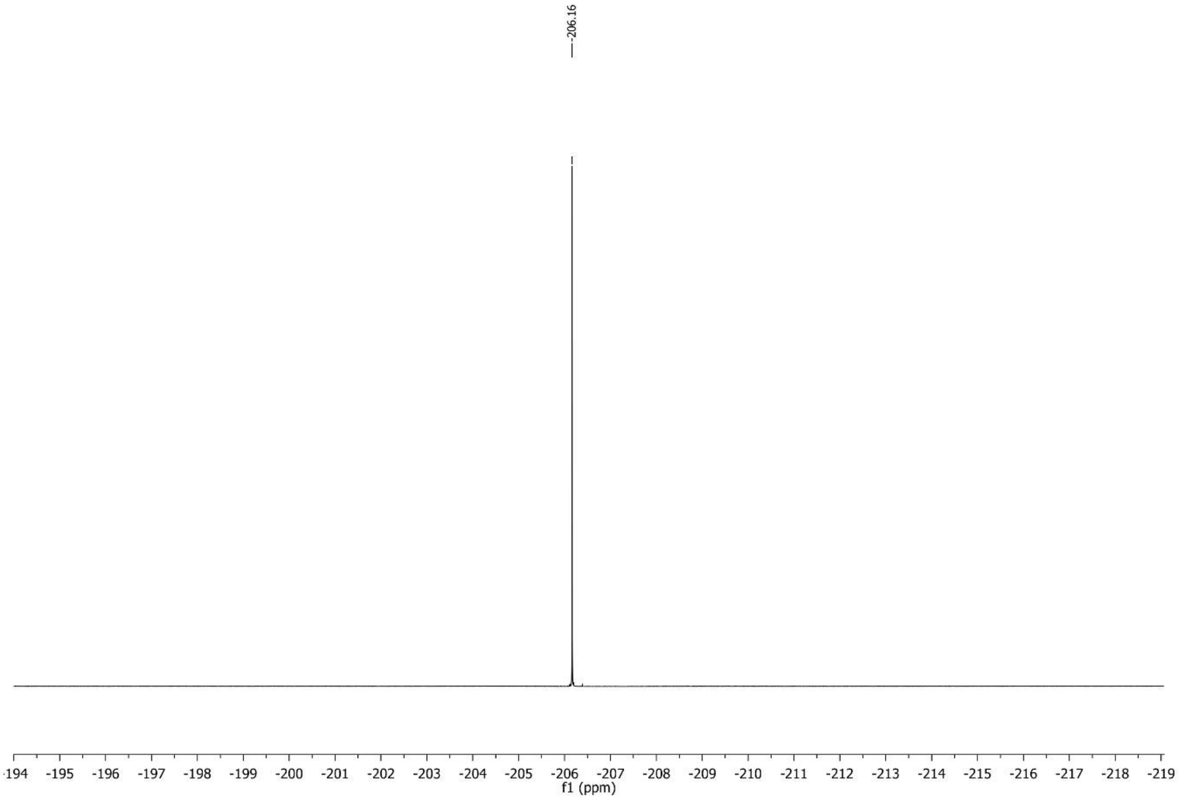

Figure S41: $^{19}F\{^{1}H\}$ NMR (659 MHz, $CDCl_3$) of 2,6-anhydro-3,5,7-tri-*O*-benzyl-1,4-dideoxy-4-fluoro-D-*altro*-hept-1-enitol (**8**).

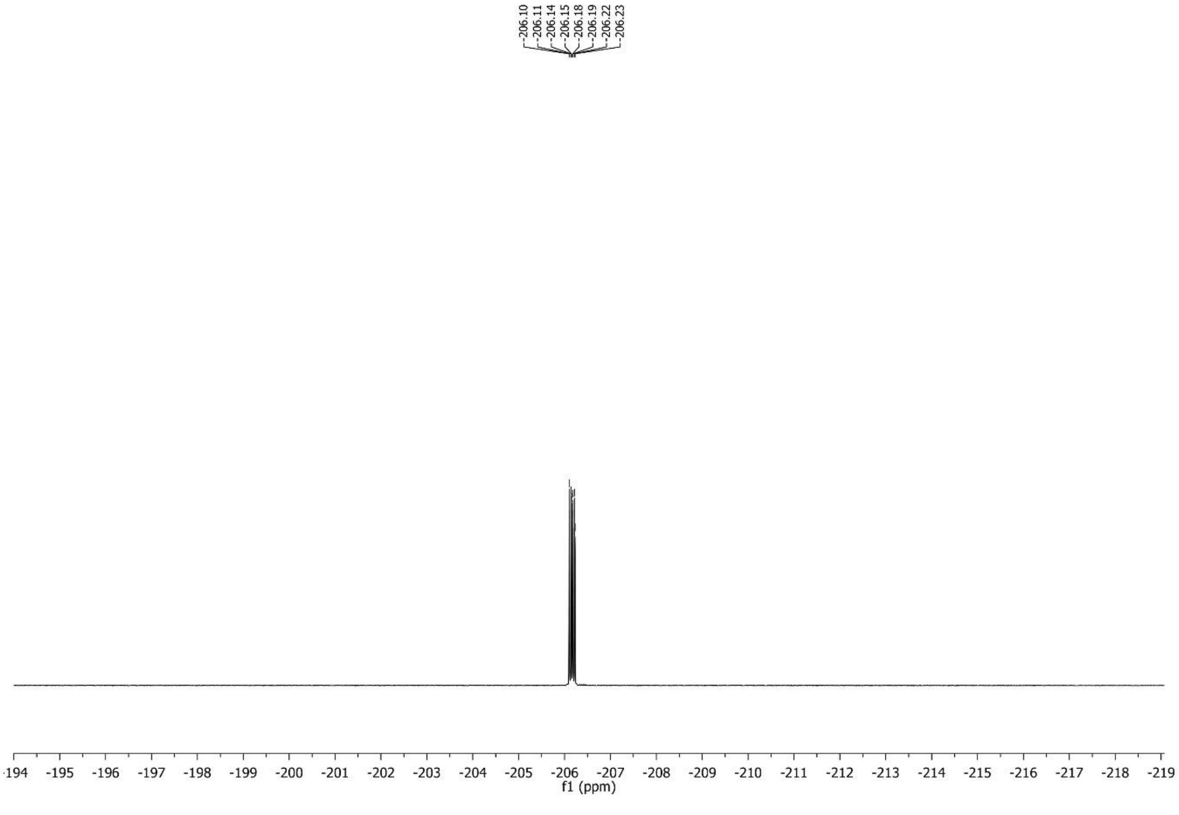

Figure S42: ^{19}F NMR (659 MHz, $CDCl_3$) of 2,6-anhydro-3,5,7-tri-*O*-benzyl-1,4-dideoxy-4-fluoro-D-*altro*-hept-1-enitol (**8**).

3.2.10 3,5,7-Tri-*O*-benzyl-4-deoxy-4-fluoro-D-*glycero*-D-*arabino*-hept-2-ulopyranose (9)

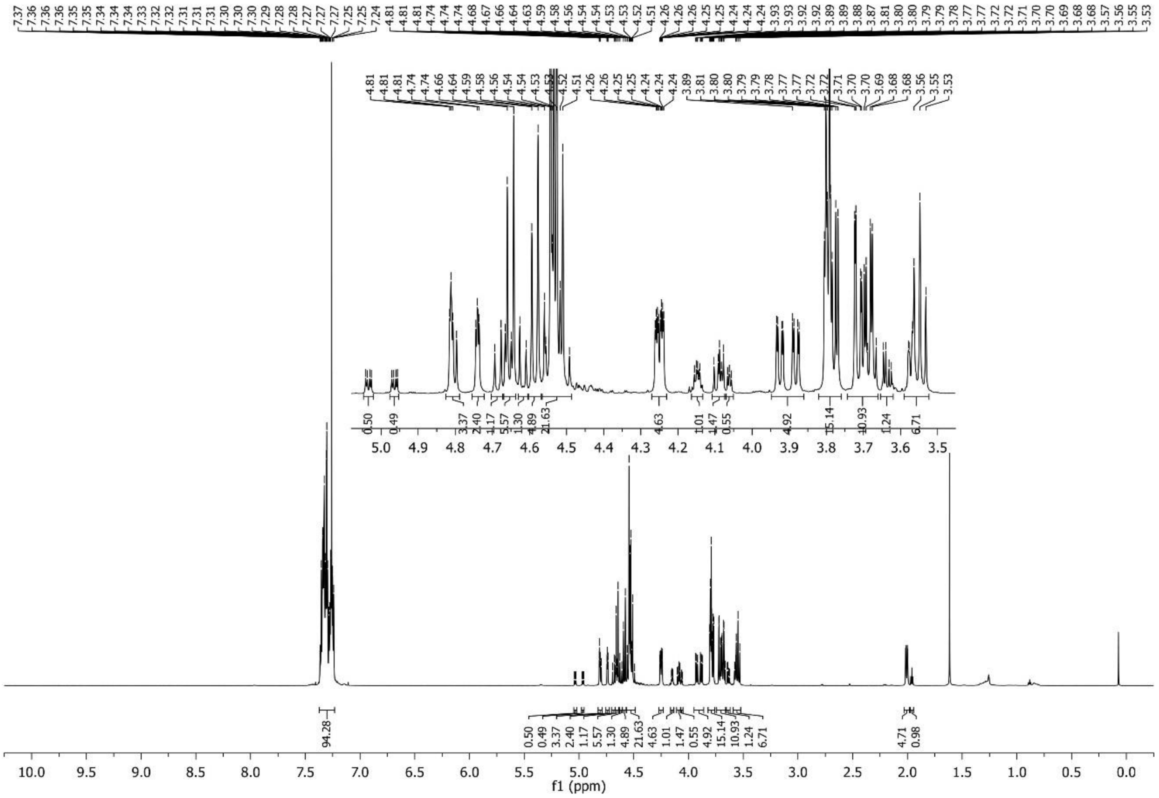

Figure S43: ^{1}H NMR (700 MHz, $CDCl_3$) of 3,5,7-tri-*O*-benzyl-4-deoxy-4-fluoro-D-*glycero*-D-*arabino*-hept-2-ulopyranose (**9**).

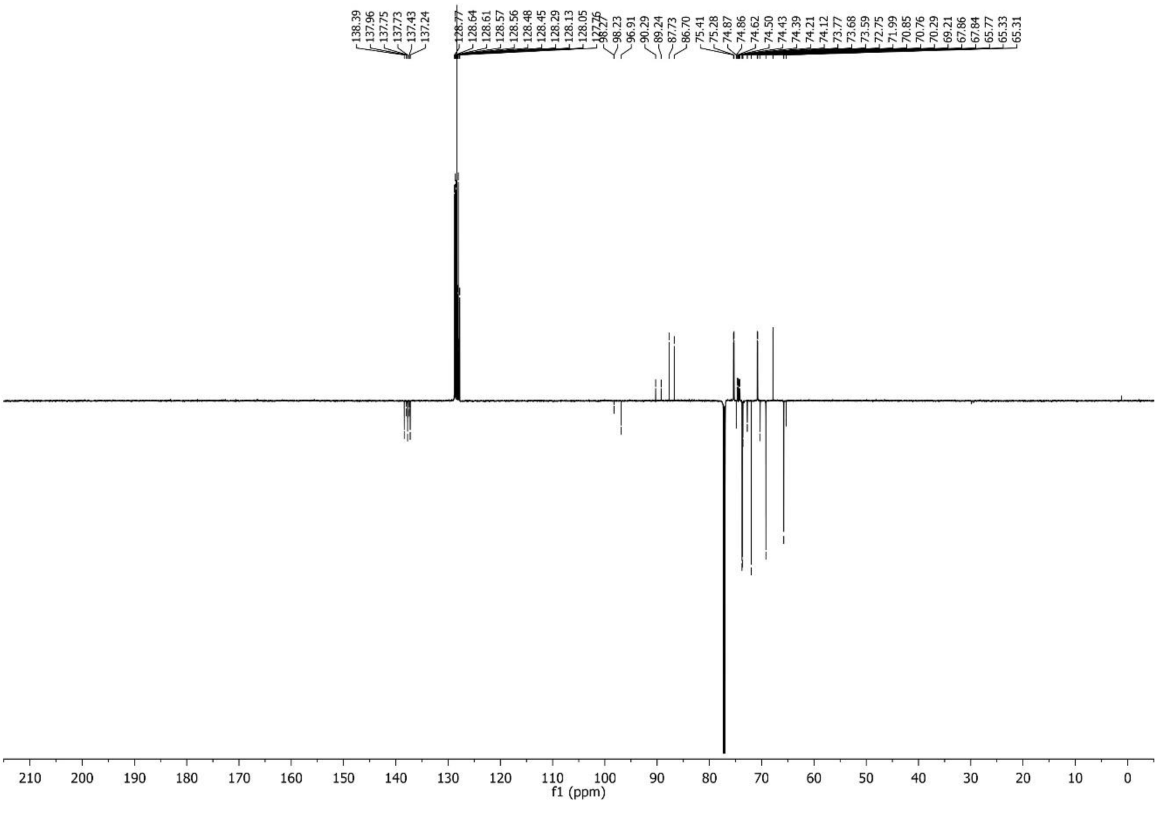

Figure S44: ^{13}C NMR (176 MHz, $CDCl_3$) of 3,5,7-tri-*O*-benzyl-4-deoxy-4-fluoro-D-*glycero*-D-*arabino*-hept-2-ulopyranose (**9**).

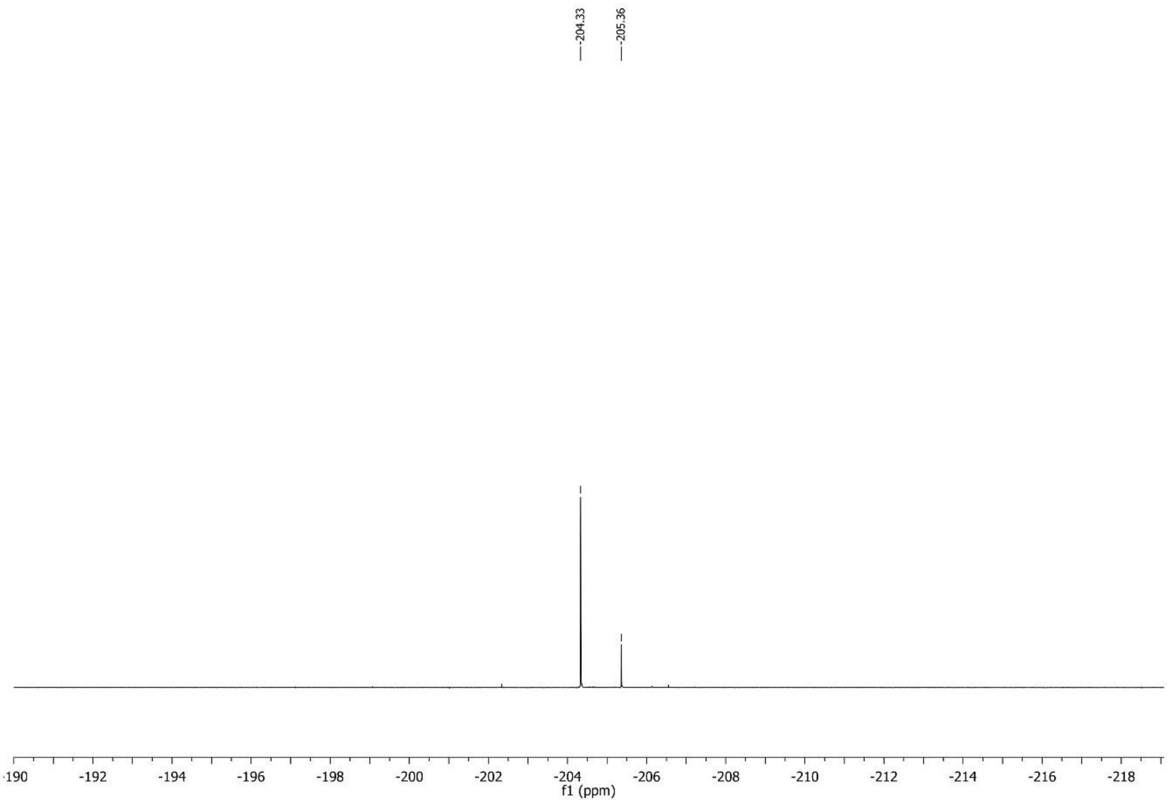

Figure S45: $^{19}F\{^{1}H\}$ NMR (659 MHz, $CDCl_3$) of 3,5,7-tri-*O*-benzyl-4-deoxy-4-fluoro-D-*glycero*-D-*arabino*-hept-2-ulopyranose (**9**).

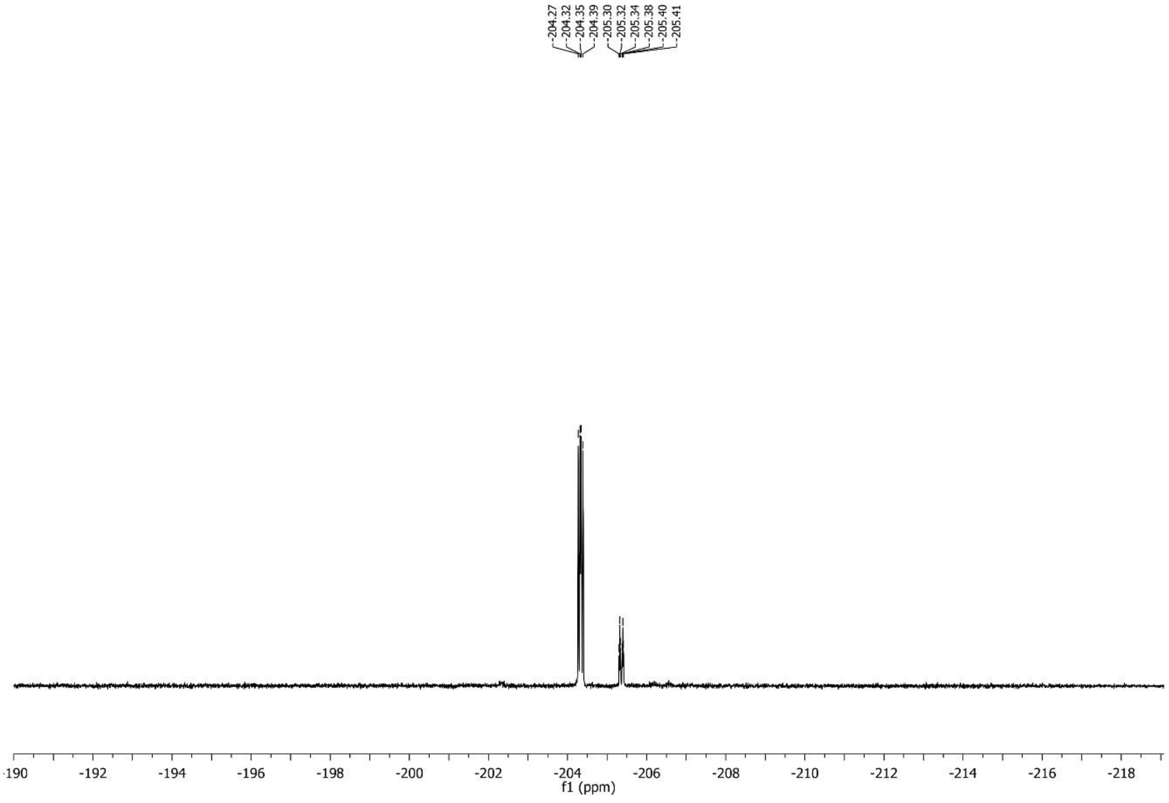

Figure S46: ^{19}F NMR (659 MHz, $CDCl_3$) of 3,5,7-tri-*O*-benzyl-4-deoxy-4-fluoro-D-*glycero*-D-*arabino*-hept-2-ulopyranose (**9**).

3.2.11 4-Deoxy-4-fluoro-D-sedoheptulose/ 4-Deoxy-4-fluoro-D-*glycero*-D-*arabino*-hept-2-ulopyranose & 4-deoxy-4-fluoro-D-*glycero*-D-*arabino*-hept-2-ulofuranose (4DFS)

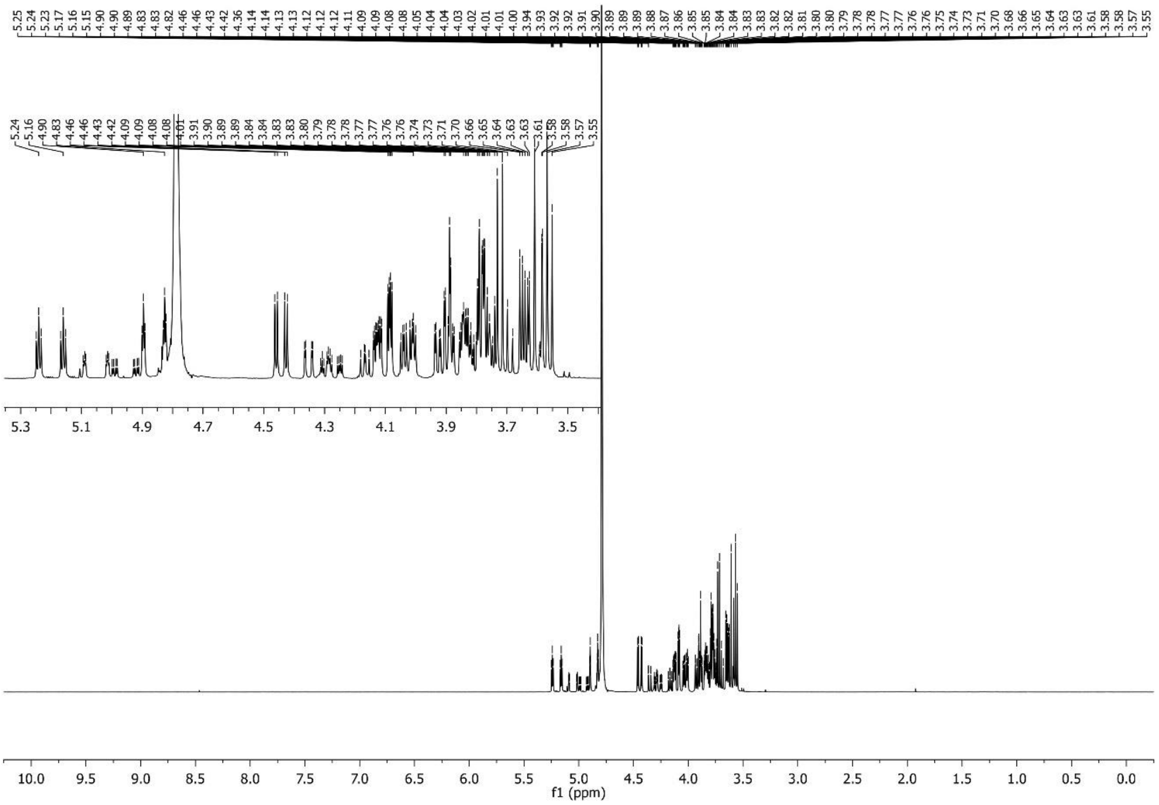

Figure S47: ^{1}H NMR (700 MHz, D_2O) of 4-deoxy-4-fluoro-D-sedoheptulose (**4DFS**).

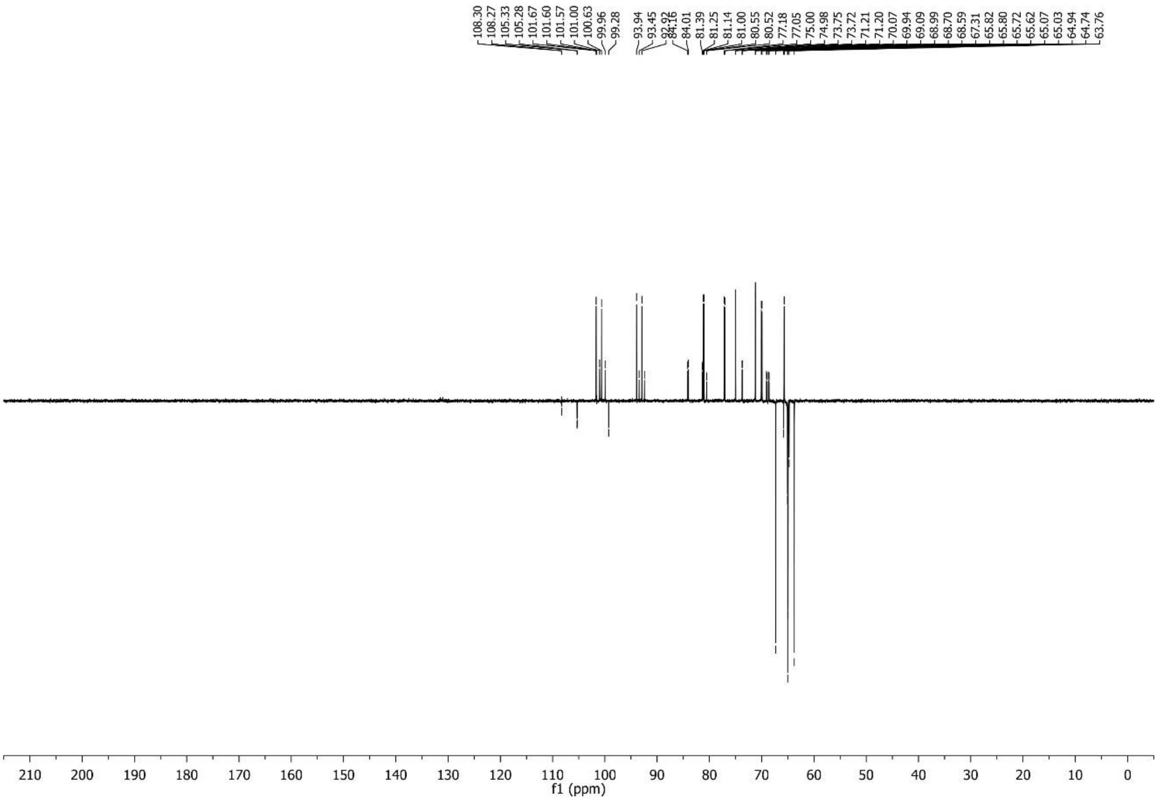

Figure S48: ^{13}C NMR (176 MHz, D_2O) of 4-deoxy-4-fluoro-D-sedoheptulose (**4DFS**).

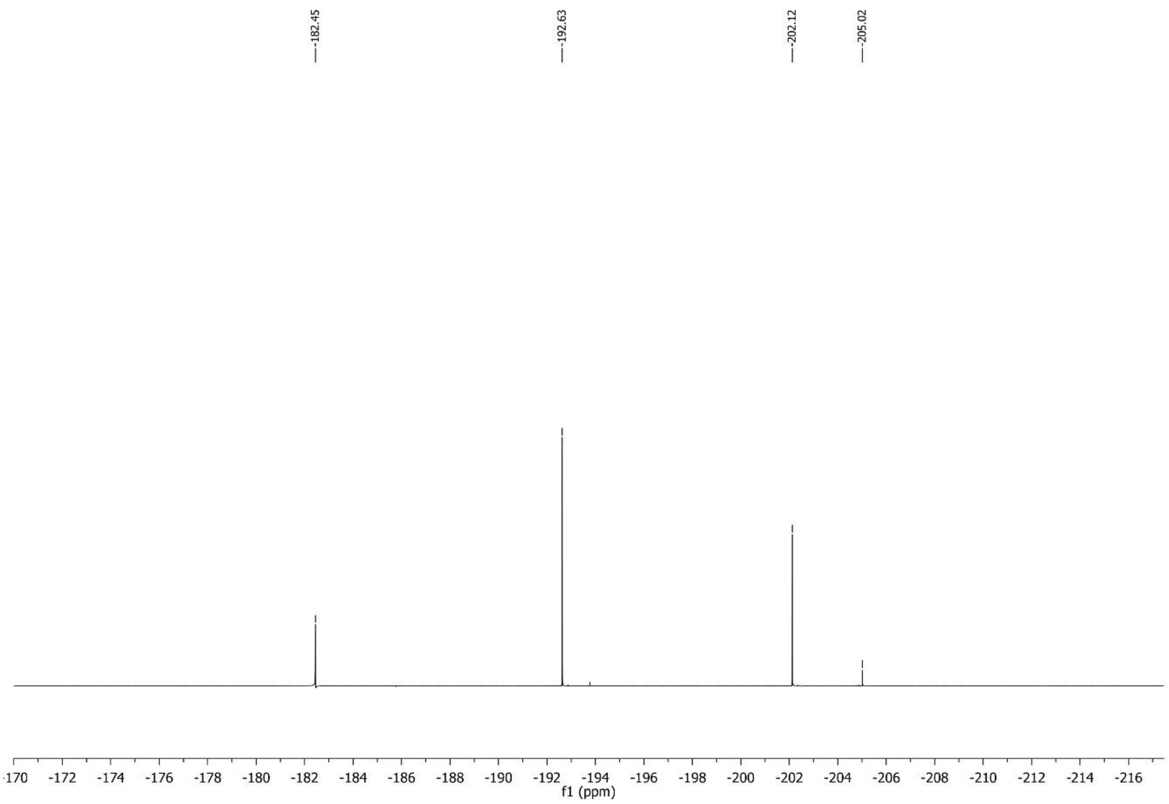

Figure S49: $^{19}F\{^{1}H\}$ NMR (659 MHz, D_2O) of 4-deoxy-4-fluoro-D-sedoheptulose (**4DFS**).

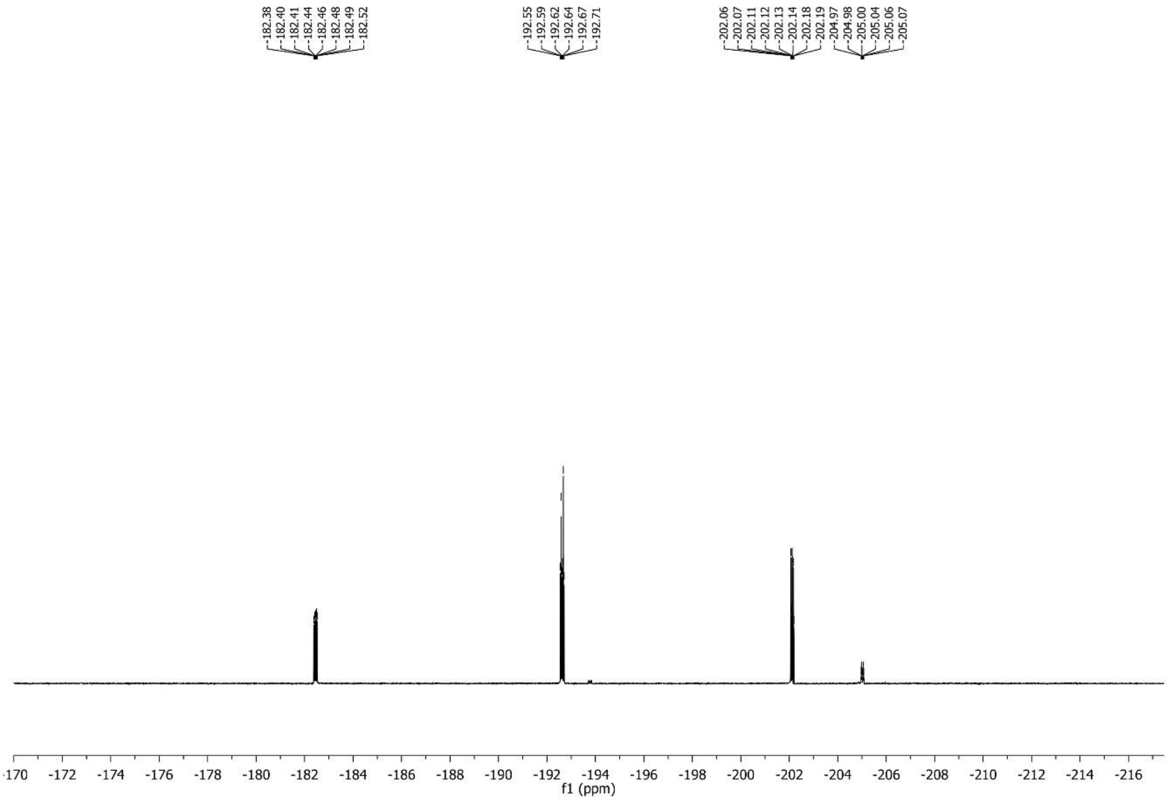

Figure S50: ^{19}F NMR (659 MHz, D_2O) of 4-deoxy-4-fluoro-D-sedoheptulose (**4DFS**).

3.2.12 Methyl 4,6-*O*-benzylidene-2,3-di-*O*-*para*-toluenesulfonyl-α-D-*gluco*-pyranoside (10)

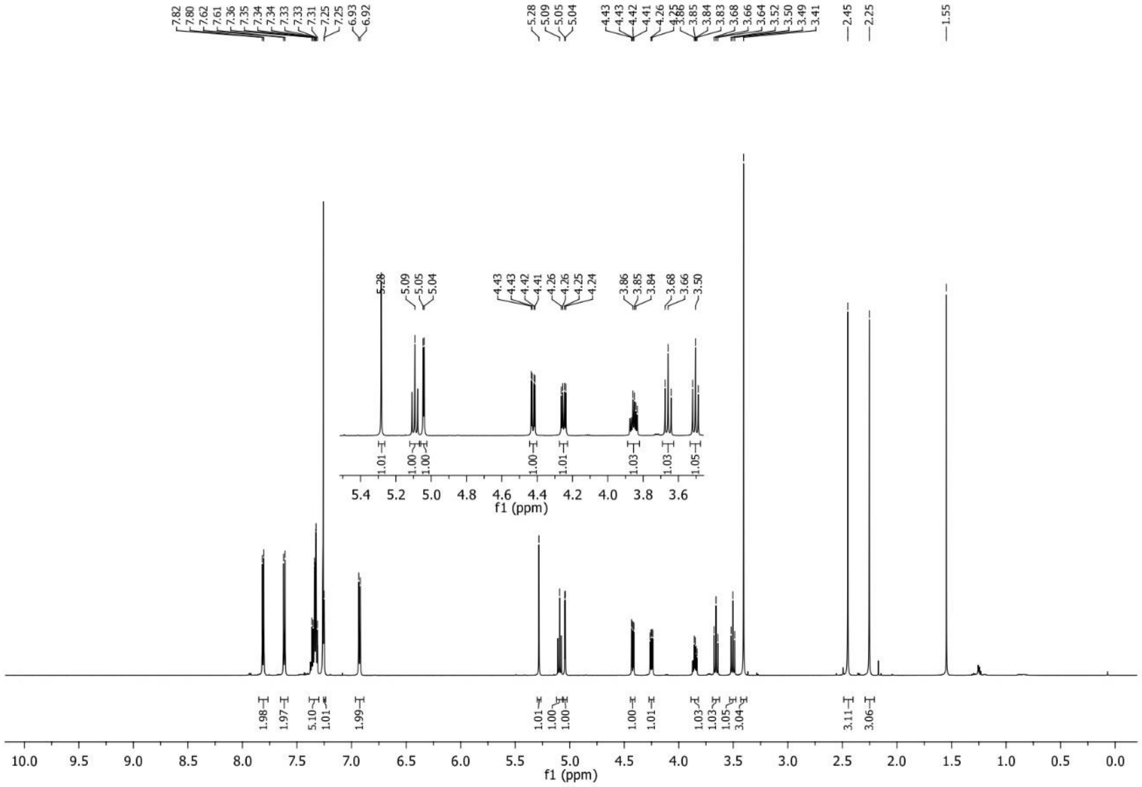

Figure S51: ^{1}H NMR (600 MHz, $CDCl_3$) of methyl 4,6-*O*-benzylidene-2,3-di-*O*-*para*-toluenesulfonyl-α-D-*gluco*-pyranoside (**10**).

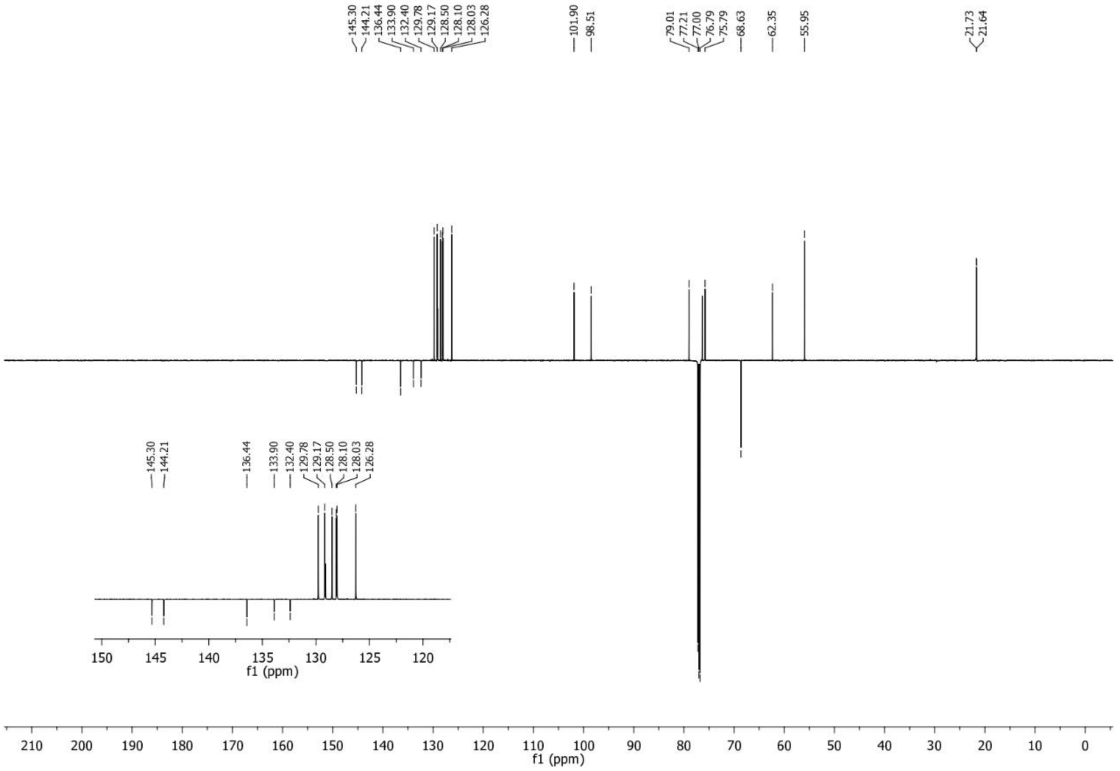

Figure S52: ^{13}C NMR (151 MHz, $CDCl_3$) of methyl 4,6-*O*-benzylidene-2,3-di-*O*-*para*-toluenesulfonyl-α-D-*gluco*-pyranoside (**10**).

3.2.13 Methyl 2,3-anhydro-4,6-*O*-benzylidene-α-D-*allo*-pyranoside (11)

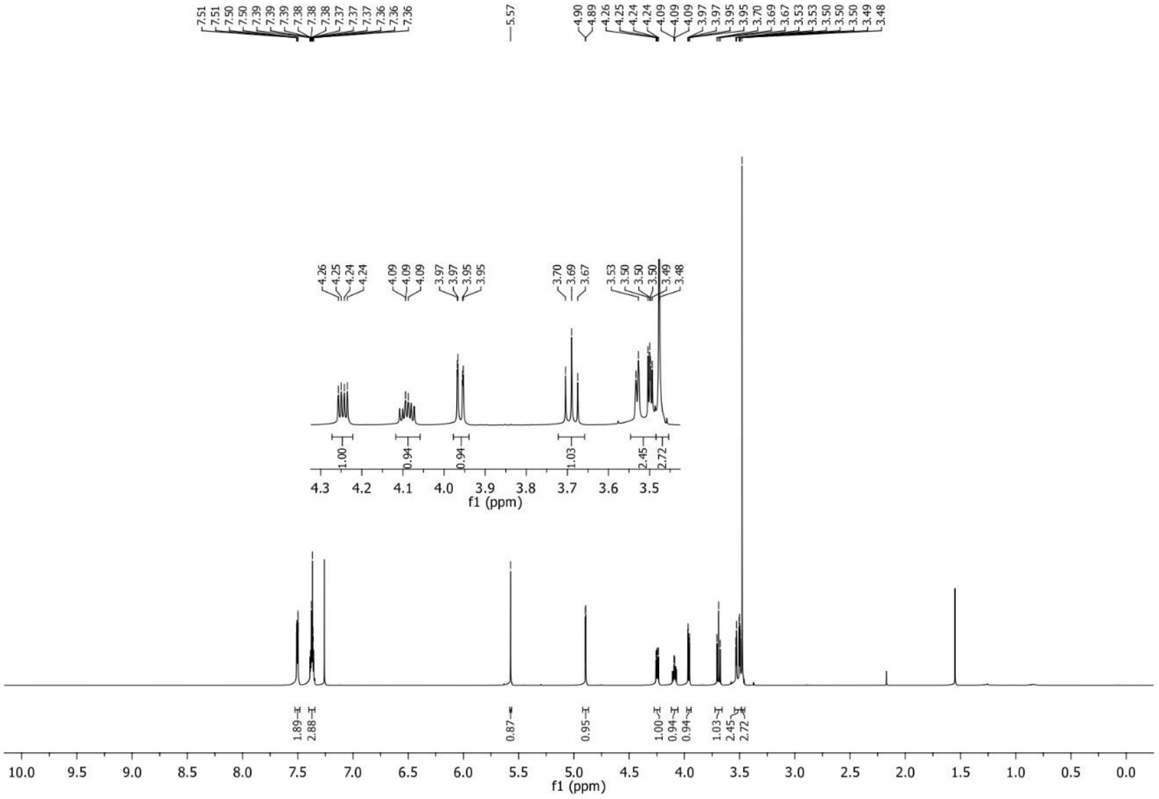

Figure S53: ^{1}H NMR (700 MHz, $CDCl_3$) of methyl 2,3-anhydro-4,6-*O*-benzylidene-α-D-*allo*-pyranoside (**11**).

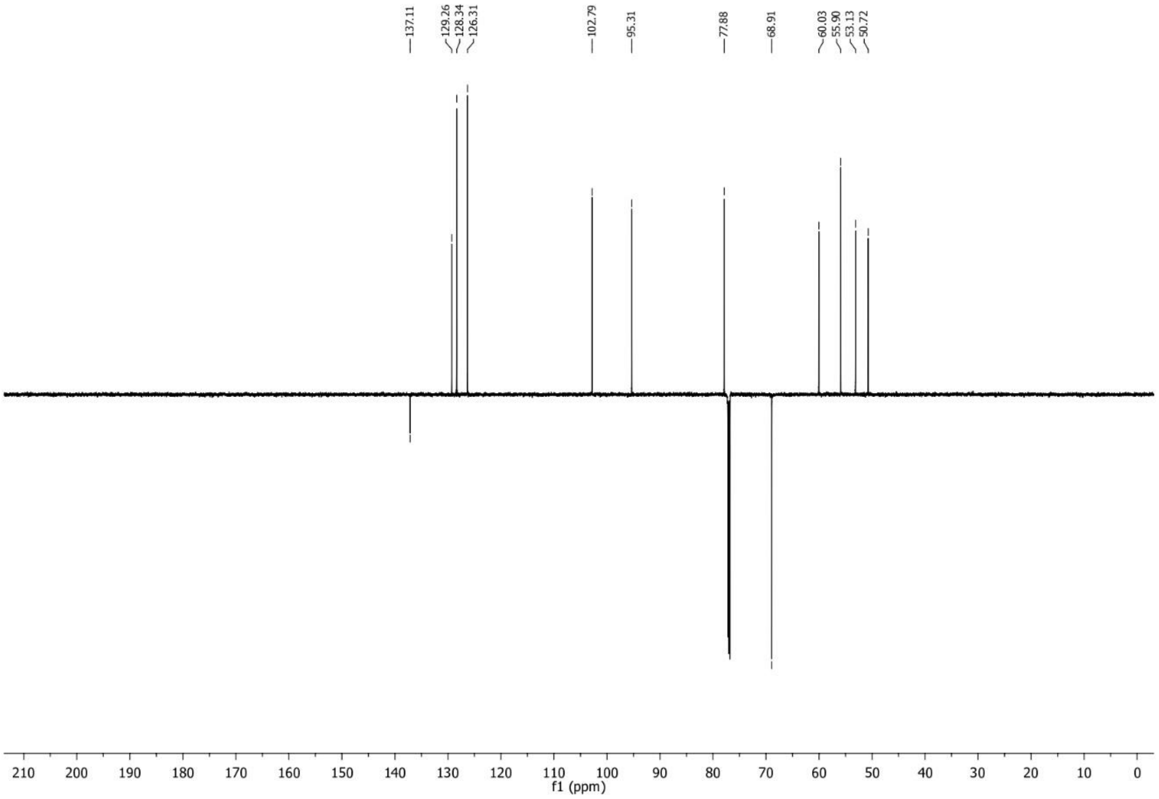

Figure S54: ^{13}C NMR (176 MHz, $CDCl_3$) of methyl 2,3-anhydro-4,6-*O*-benzylidene-α-D-*allo*-pyranoside (**11**).

3.2.14 Methyl 4,6-*O*-benzylidene-2-deoxy-2-fluoro-α-D-*altro*-pyranoside (12)

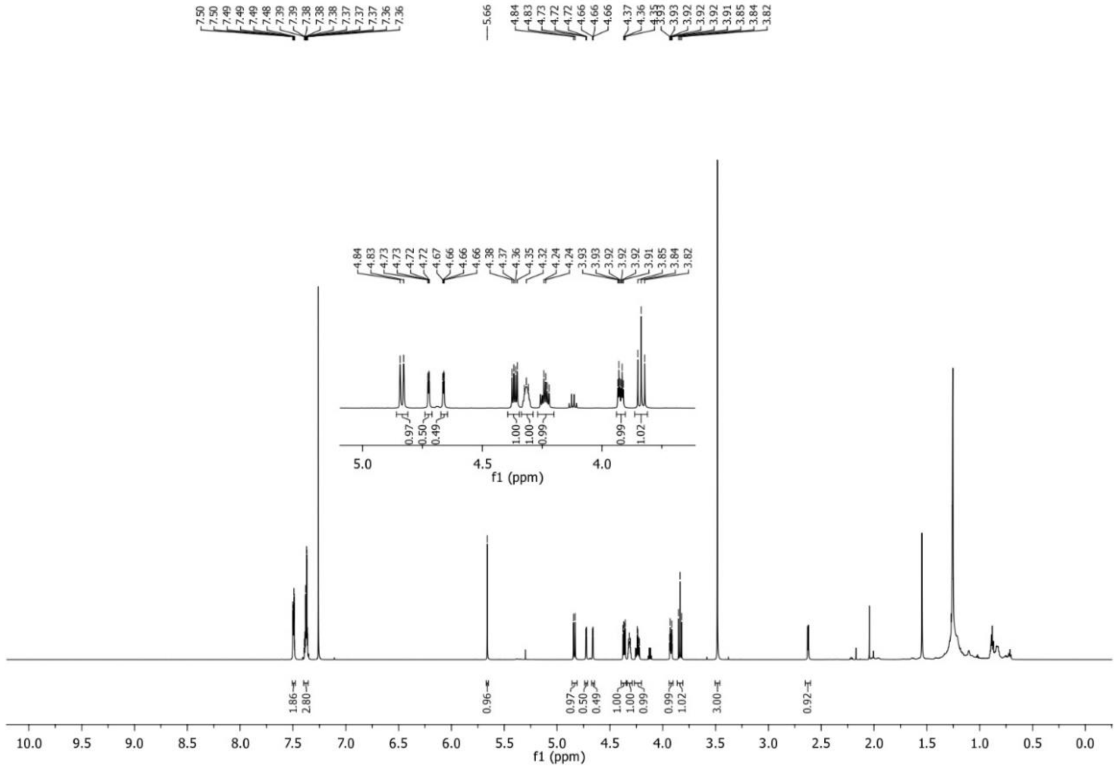

Figure S55: ^{1}H NMR (700 MHz, $CDCl_3$) of methyl 4,6-*O*-benzylidene-2-deoxy-2-fluoro-α-D-*altro*-pyranoside (**12**).

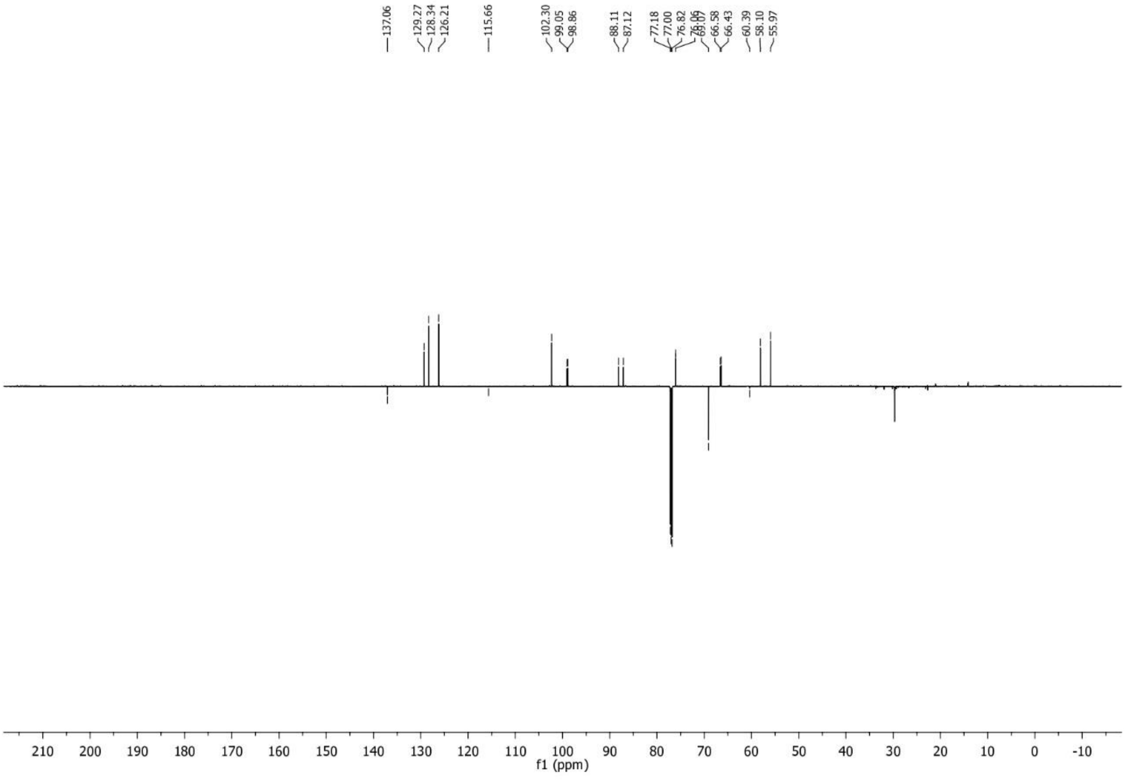

Figure S56: ^{13}C NMR (176 MHz, $CDCl_3$) of methyl 4,6-*O*-benzylidene-2-deoxy-2-fluoro-α-D-*altro*-pyranoside (**12**).

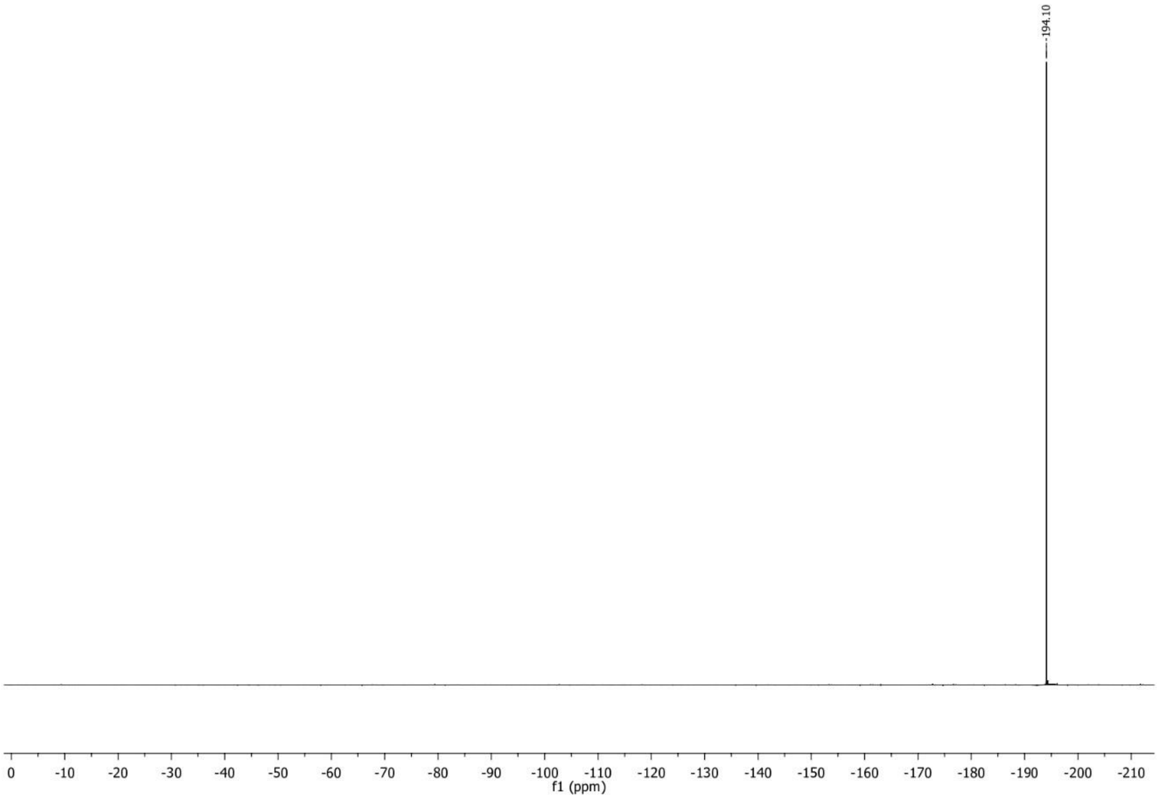

Figure S57: $^{19}F\{^{1}H\}$ NMR (659 MHz, $CDCl_3$) of methyl 4,6-*O*-benzylidene-2-deoxy-2-fluoro-α-D-*altro*-pyranoside (**12**).

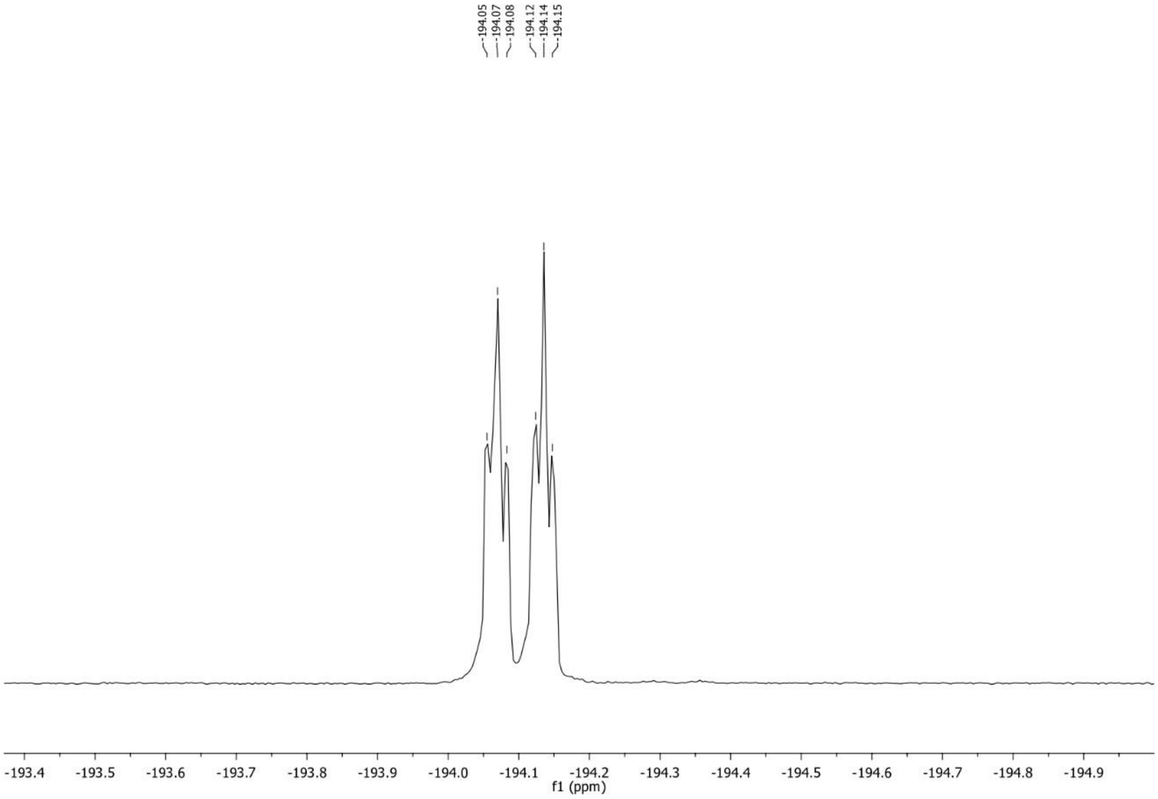

Figure S58: ^{19}F NMR (659 MHz, $CDCl_3$) of methyl 4,6-*O*-benzylidene-2-deoxy-2-fluoro-α-D-*altro*-pyranoside (**12**).

3.2.15 Methyl 4-*O*-benzyl-2-deoxy-2-fluoro-α-D-*altro*-pyranoside (13a) and methyl 6-*O*-benzyl-2-deoxy-2-fluoro-α-D-*altro*-pyranoside (13b)

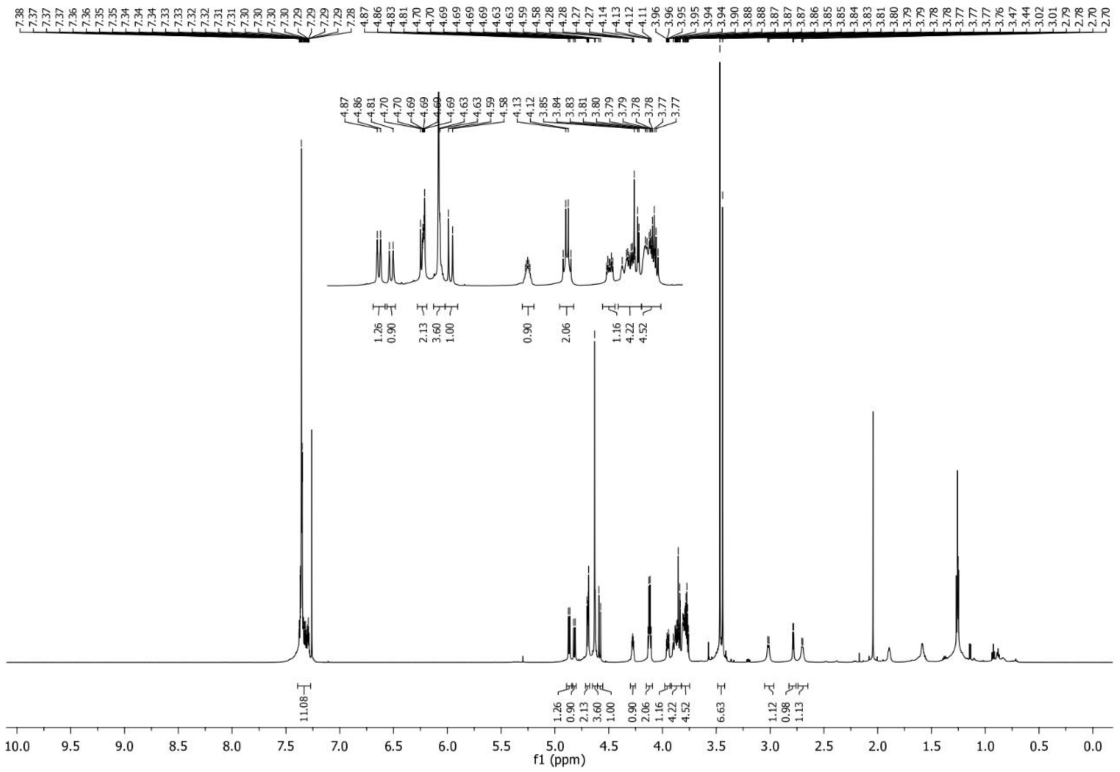

Figure S59: ^{1}H NMR (700 MHz, $CDCl_3$) of methyl 4-*O*-benzyl-2-deoxy-2-fluoro-α-D-*altro*-pyranoside (**13a**) and methyl 6-*O*-benzyl-2-deoxy-2-fluoro-α-D-*altro*-pyranoside (**13b**).

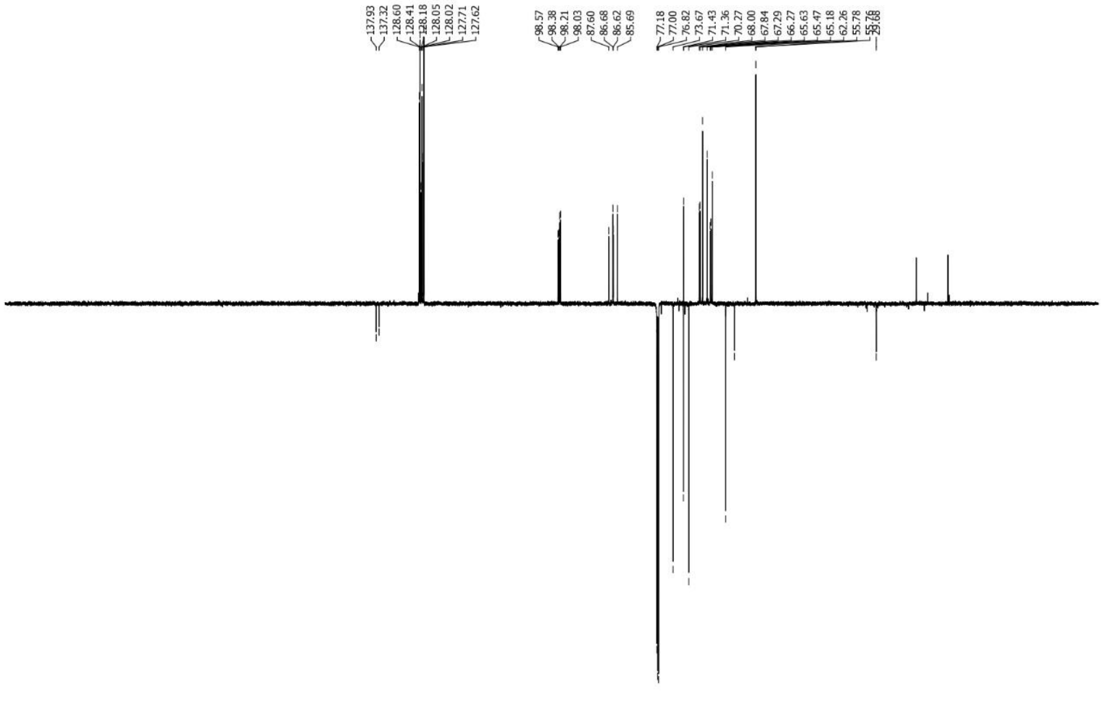

Figure S60: ^{13}C NMR (176 MHz, $CDCl_3$) of methyl 4-*O*-benzyl-2-deoxy-2-fluoro-α-D-*altro*-pyranoside (**13a**) and methyl 6-*O*-benzyl-2-deoxy-2-fluoro-α-D-*altro*-pyranoside (**13b**).

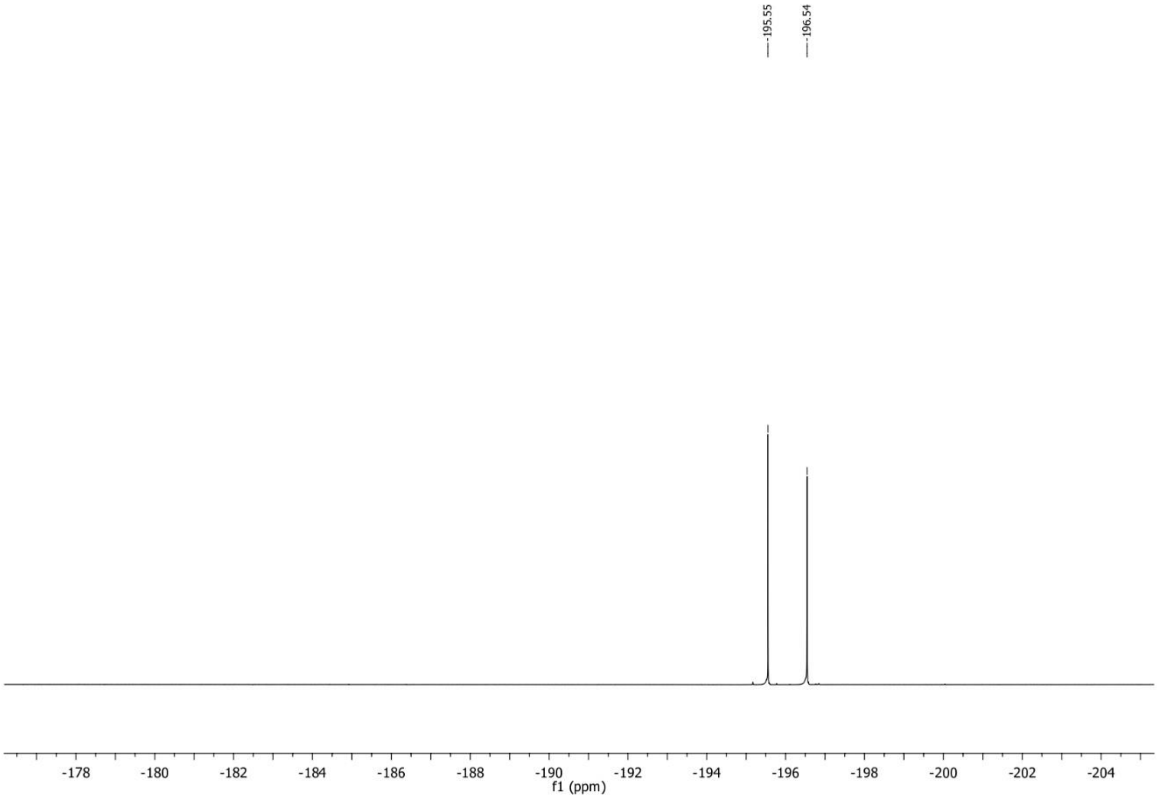

Figure S61: $^{19}F\{^{1}H\}$ NMR (659 MHz, $CDCl_3$) of methyl 4-*O*-benzyl-2-deoxy-2-fluoro-α-D-*altro*-pyranoside (**13a**) and methyl 6-*O*-benzyl-2-deoxy-2-fluoro-α-D-*altro*-pyranoside (**13b**).

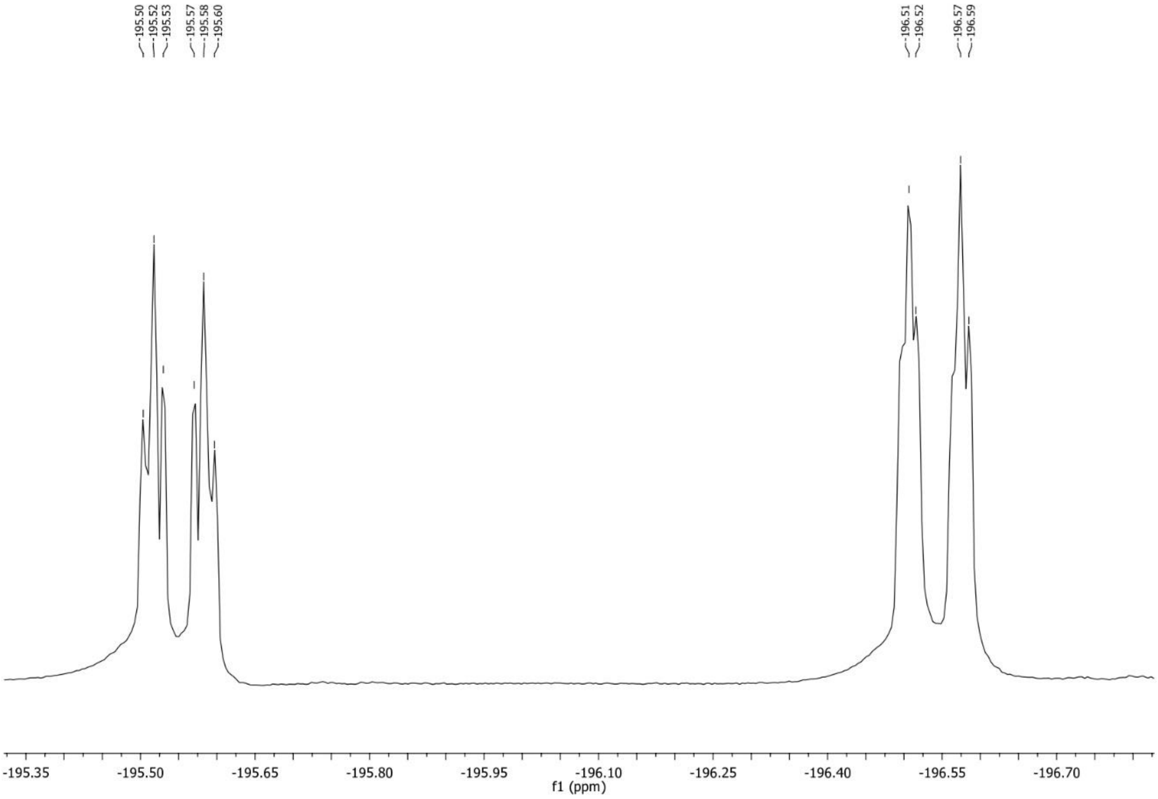

Figure S62: ^{19}F NMR (659 MHz, $CDCl_3$) of methyl 4-*O*-benzyl-2-deoxy-2-fluoro-α-D-*altro*-pyranoside (**13a**) and methyl 6-*O*-benzyl-2-deoxy-2-fluoro-α-D-*altro*-pyranoside (**13b**).

3.2.16 Methyl 3,4,6-tri-*O*-benzyl-2-deoxy-2-fluoro-α-D-*altro*-pyranoside (14)

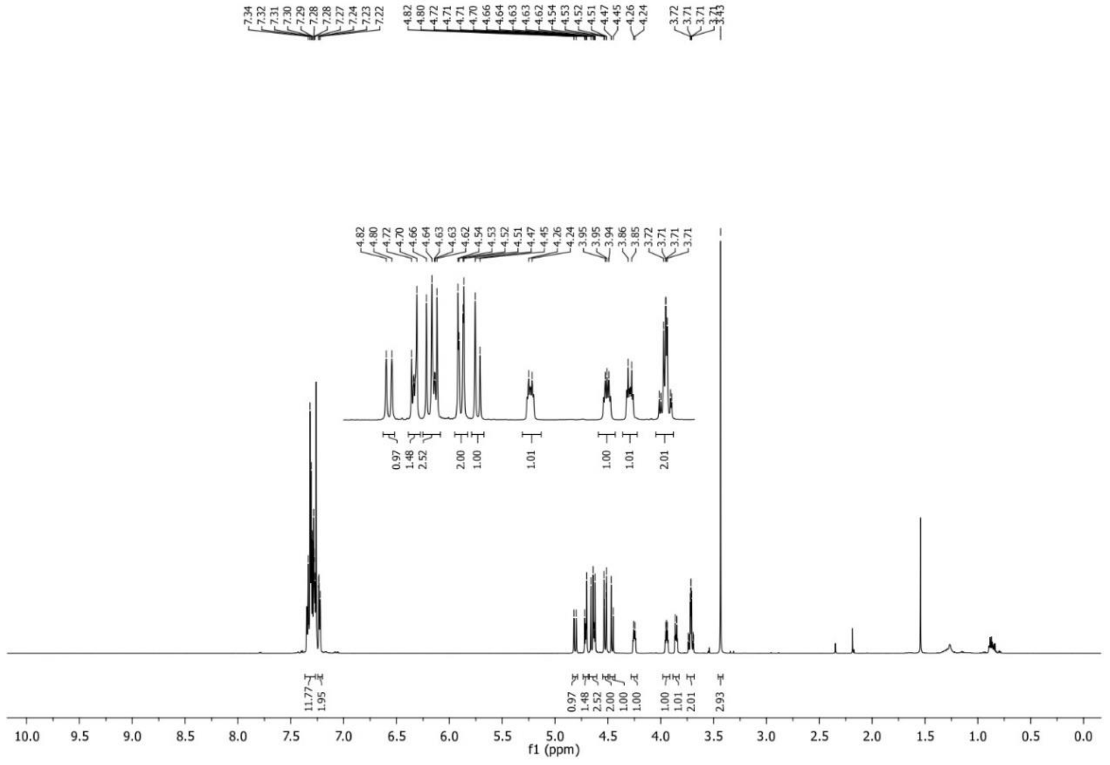

Figure S63: ^{1}H NMR (600 MHz, $CDCl_3$) of methyl 3,4,6-tri-*O*-benzyl-2-deoxy-2-fluoro-α-D-*altro*-pyranoside (**14**).

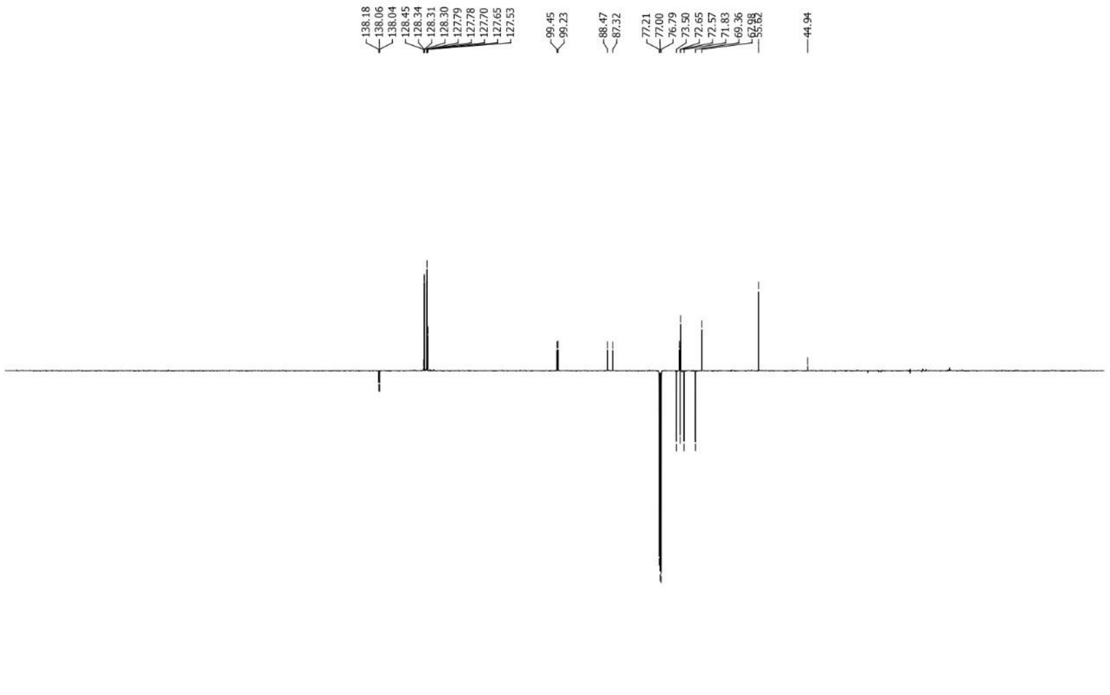

Figure S64: ^{13}C NMR (151 MHz, $CDCl_3$) of methyl 3,4,6-tri-*O*-benzyl-2-deoxy-2-fluoro-α-D-*altro*-pyranoside (**14**).

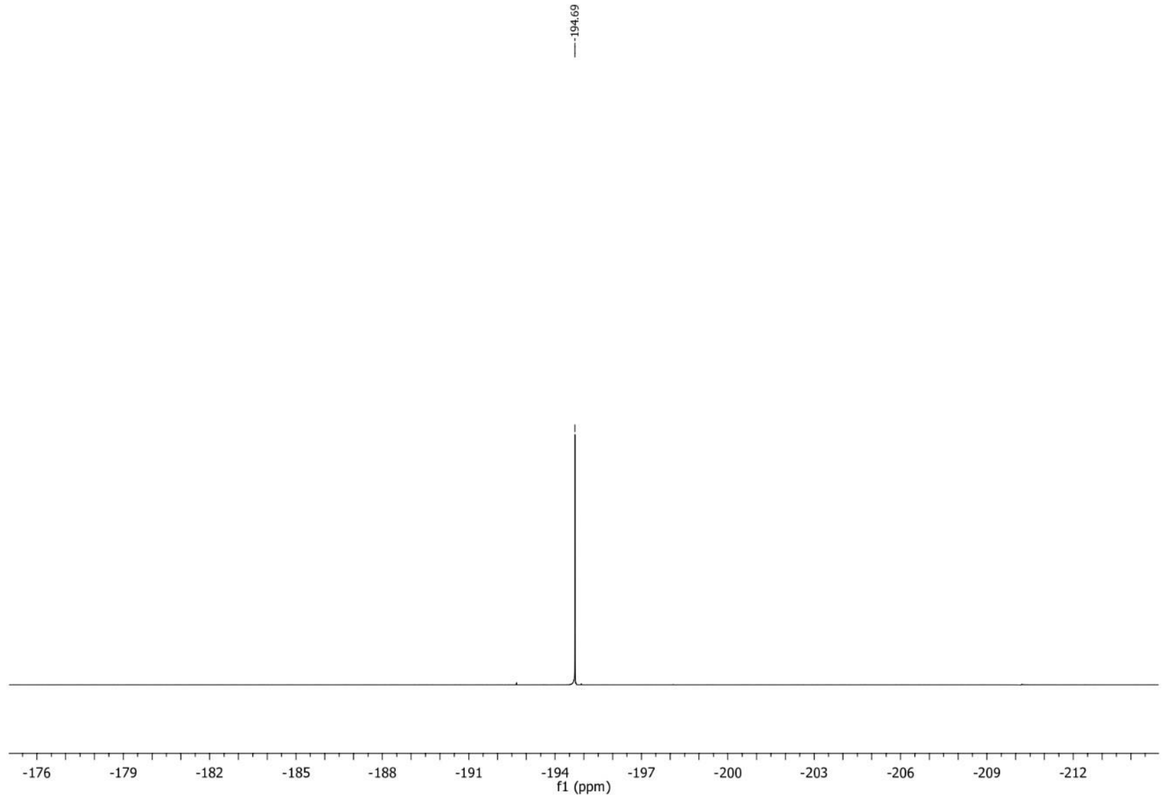

Figure S65: $^{19}F\{^{1}H\}$ NMR (659 MHz, $CDCl_3$) of methyl 3,4,6-tri-*O*-benzyl-2-deoxy-2-fluoro-α-D-*altro*-pyranoside (**14**).

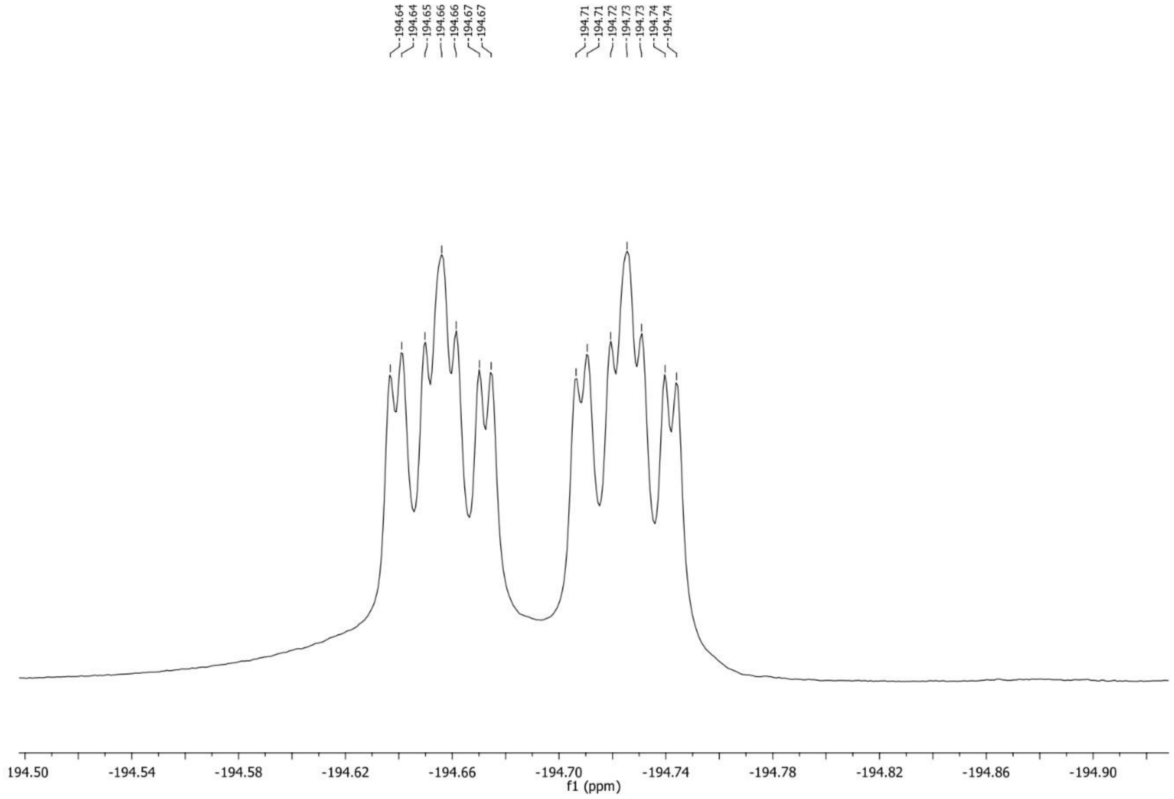

Figure S66: ^{19}F NMR (659 MHz, $CDCl_3$) of methyl 3,4,6-tri-*O*-benzyl-2-deoxy-2-fluoro-α-D-*altro*-pyranoside (**14**).

3.2.17 3,4,6-Tri-*O*-benzyl-2-deoxy-2-fluoro-α-D-*altrono*-1,5-lactone (15)

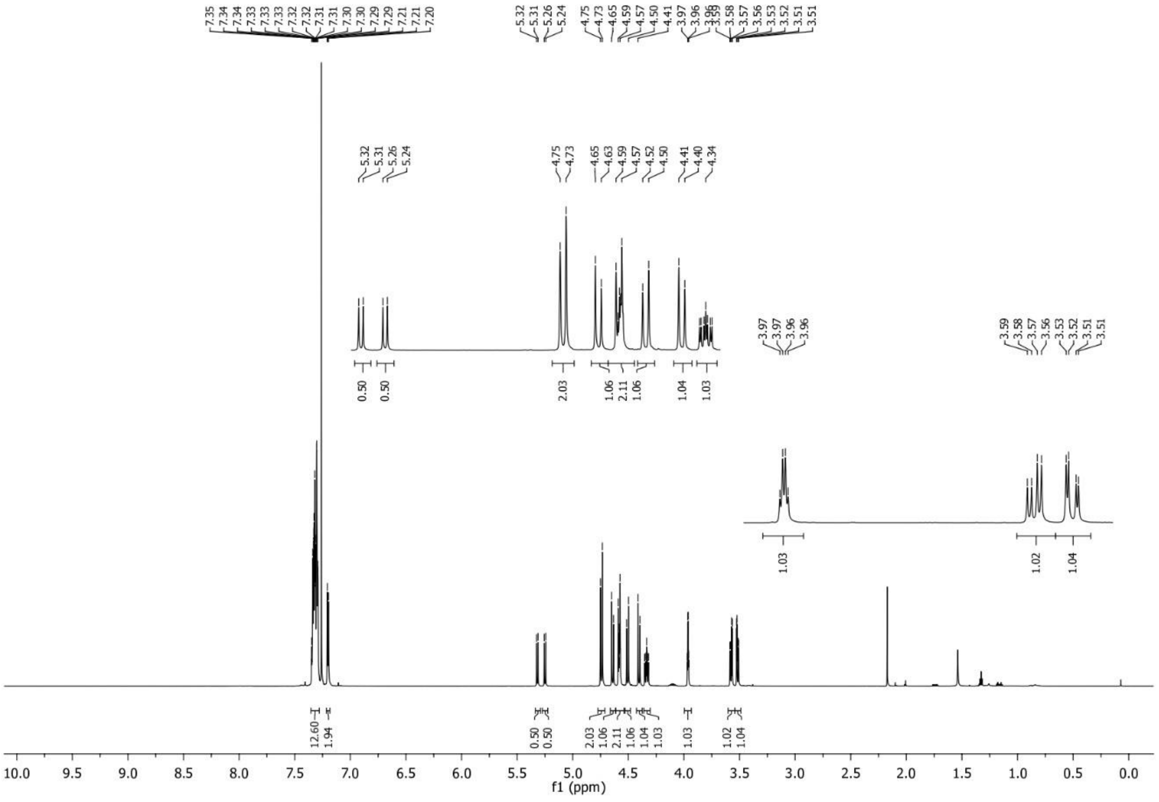

Figure S67: ^{1}H NMR (700 MHz, $CDCl_3$) of 3,4,6-tri-*O*-benzyl-2-deoxy-2-fluoro-α-D-*altrono*-1,5-lactone (**15**).

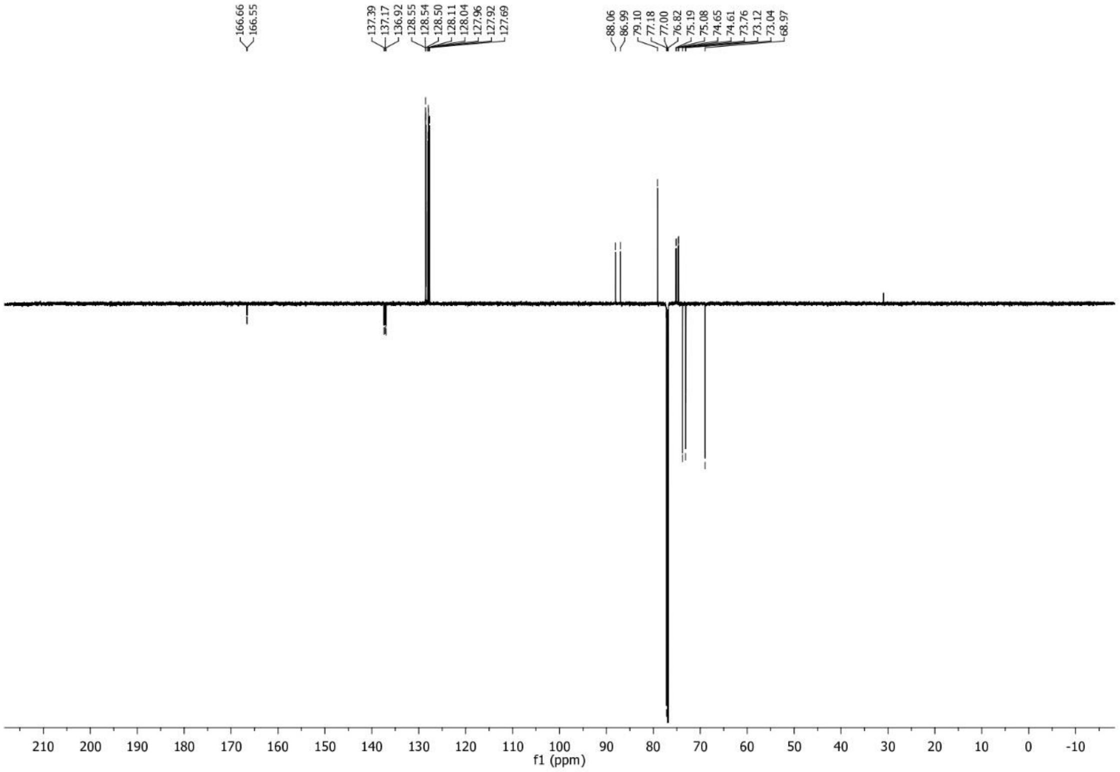

Figure S68: ^{13}C NMR (176 MHz, $CDCl_3$) of 3,4,6-tri-*O*-benzyl-2-deoxy-2-fluoro-α-D-*altrono*-1,5-lactone (**15**).

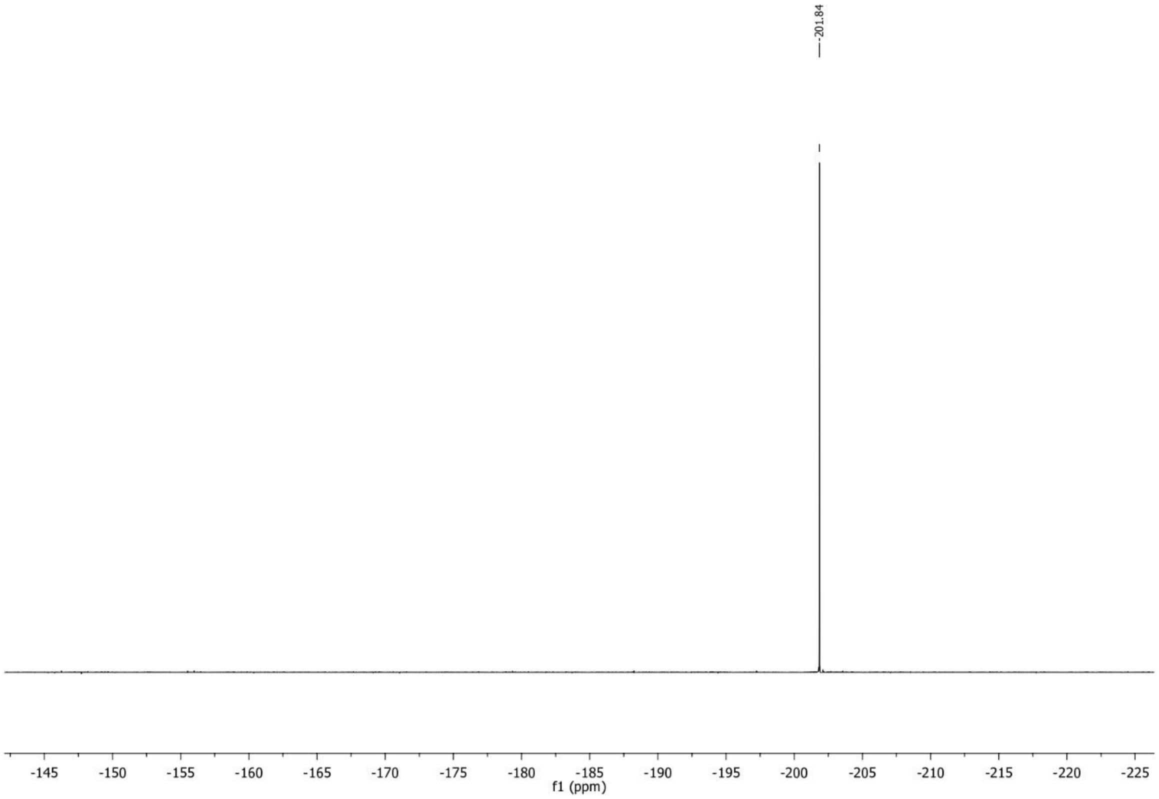

Figure S69: $^{19}F\{^{1}H\}$ NMR (659 MHz, $CDCl_3$) of3,4,6-tri-*O*-benzyl-2-deoxy-2-fluoro-α-D-*altrono*-1,5-lactone (**15**).

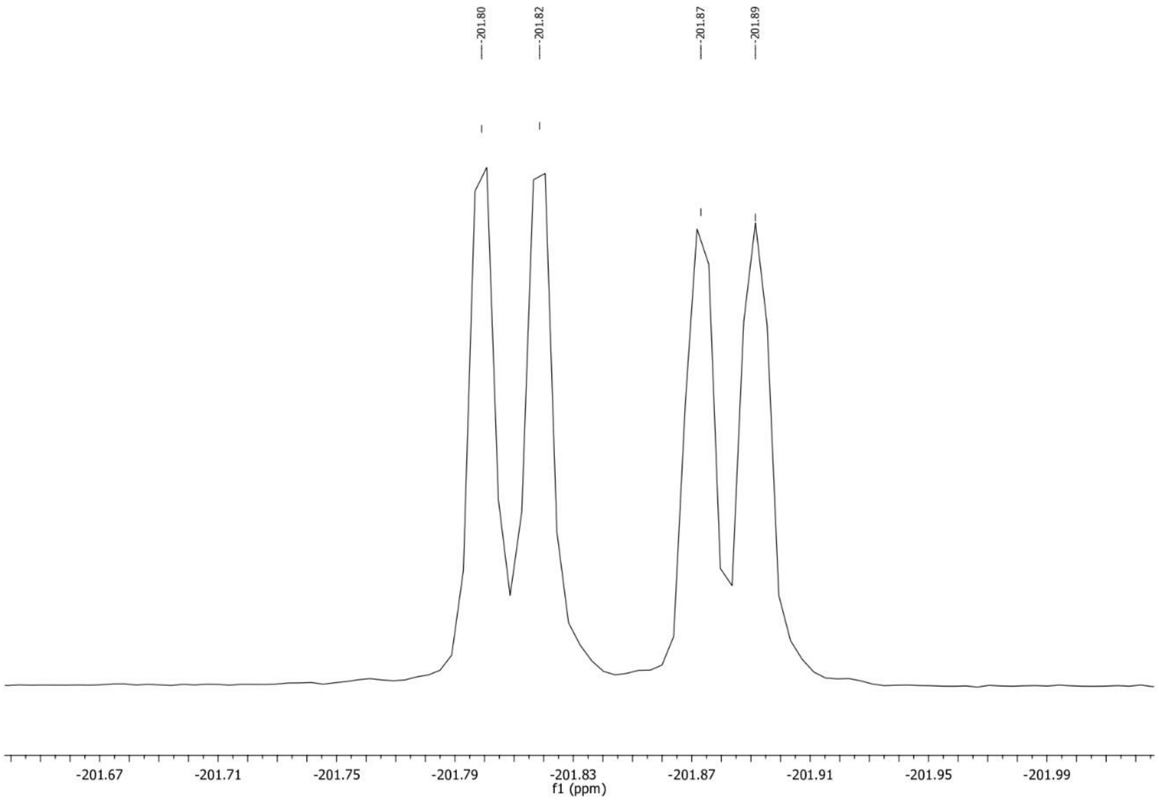

Figure S70: ^{19}F NMR (659 MHz, $CDCl_3$) of 3,4,6-tri-*O*-benzyl-2-deoxy-2-fluoro-α-D-*altrono*-1,5-lactone (**15**).

3.2.18 2,6-Anhydro-4,5,7-tri-*O*-benzyl-1,3-dideoxy-3-fluoro-D-*altro*-hept-1-enitol (16)

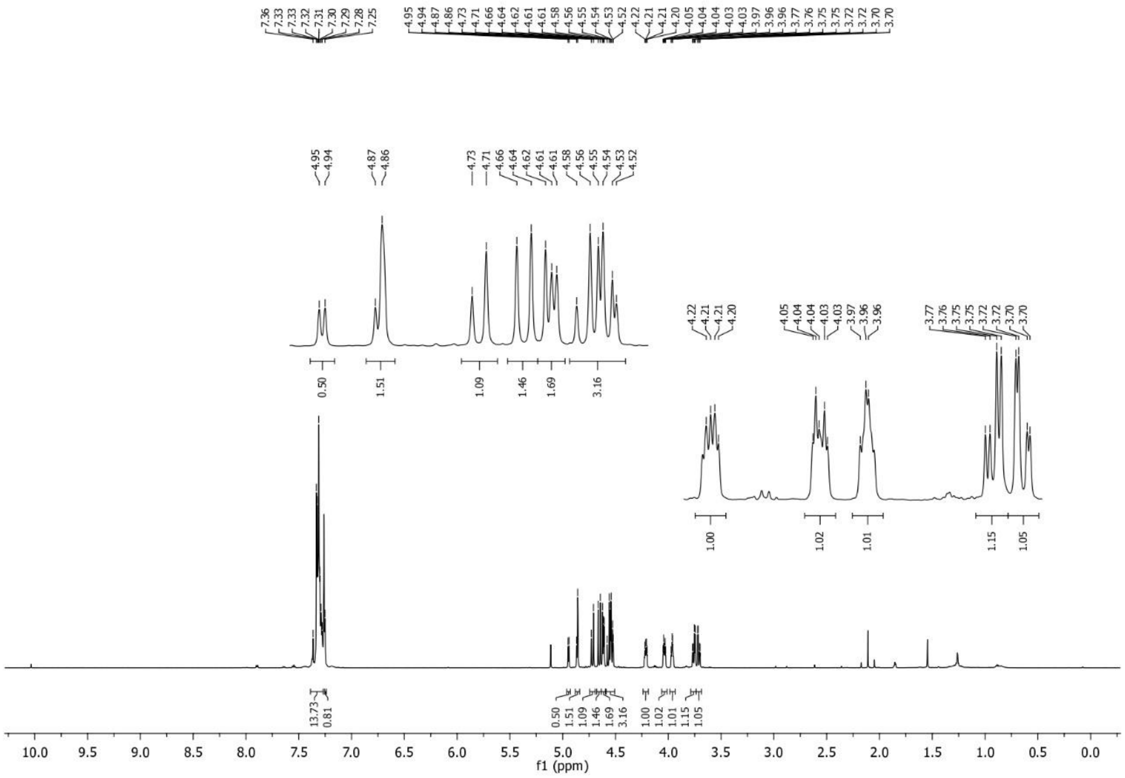

Figure S71: ^{1}H NMR (600 MHz, $CDCl_3$) of 2,6-anhydro-4,5,7-tri-*O*-benzyl-1,3-dideoxy-3-fluoro-D-*altro*-hept-1-enitol (**16**).

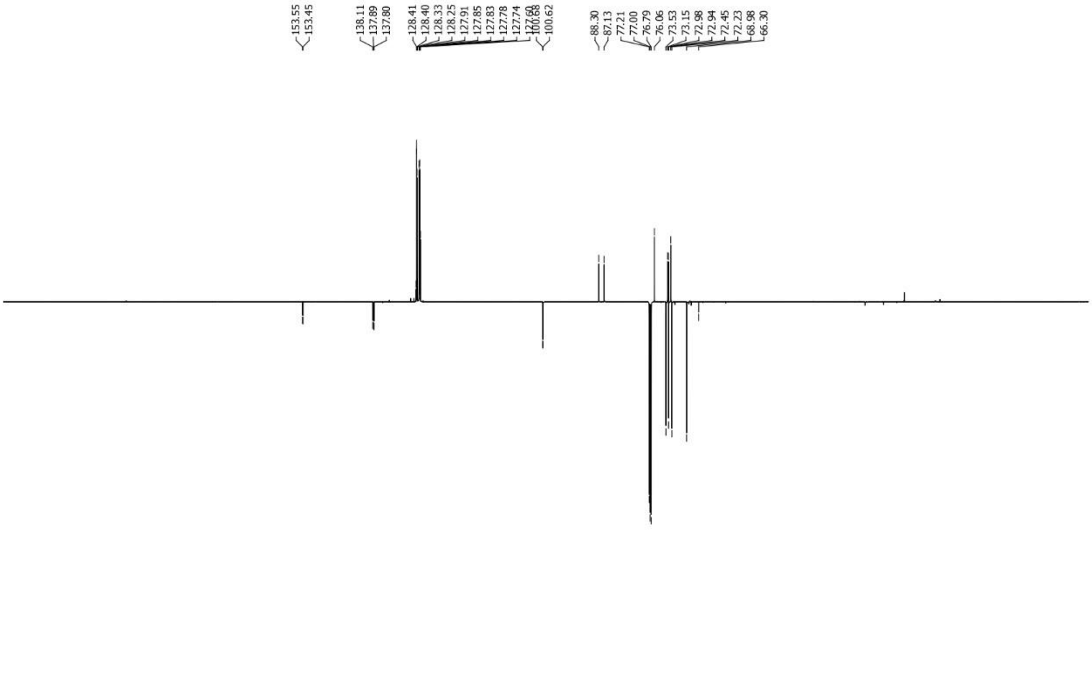

Figure S72: ^{13}C NMR (151 MHz, $CDCl_3$) of 2,6-anhydro-4,5,7-tri-*O*-benzyl-1,3-dideoxy-3-fluoro-D-*altro*-hept-1-enitol (**16**).

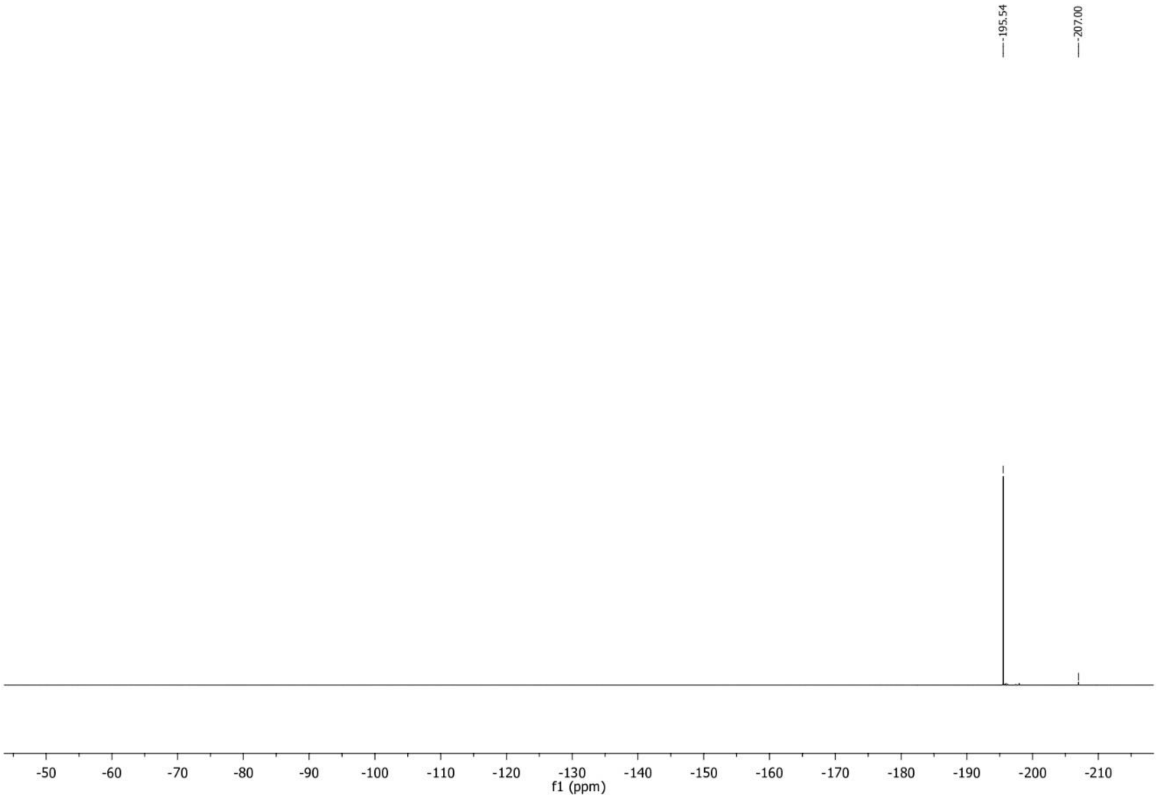

Figure S73: $^{19}F\{^{1}H\}$ NMR (659 MHz, $CDCl_3$) of 2,6-anhydro-4,5,7-tri-*O*-benzyl-1,3-dideoxy-3-fluoro-D-*altro*-hept-1-enitol (**16**).

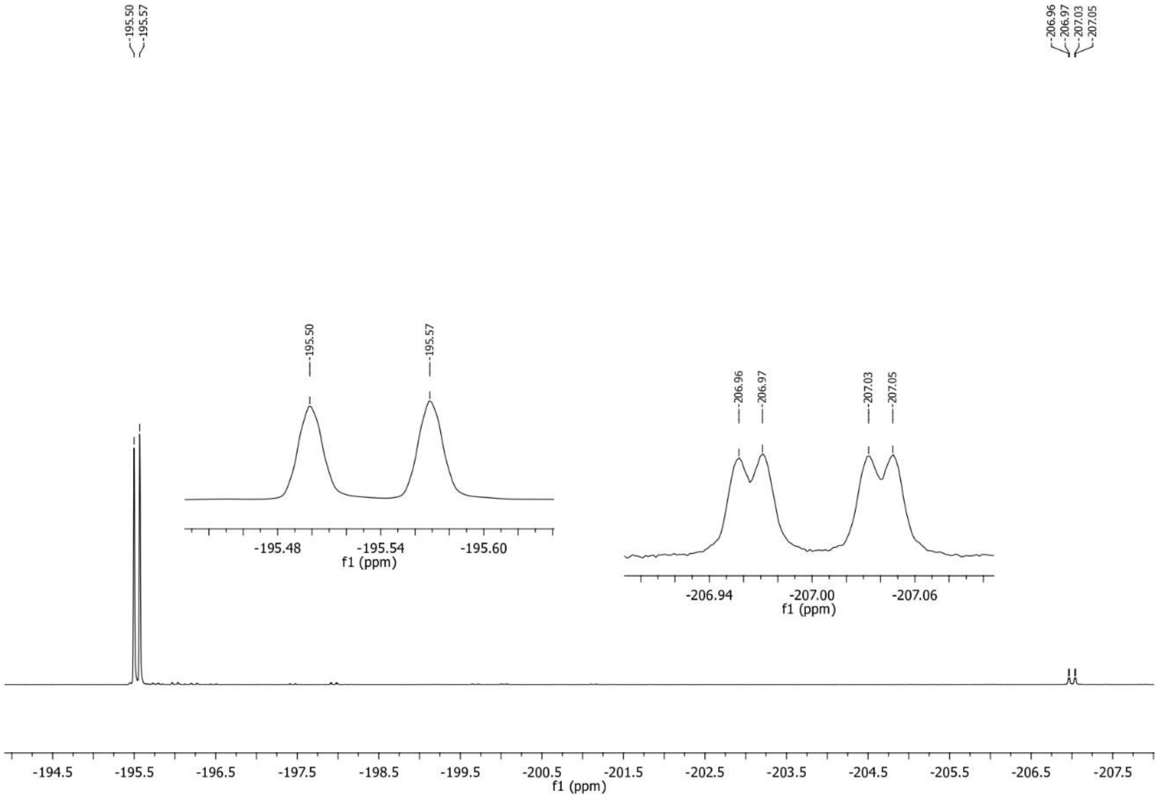

Figure S74: ^{19}F NMR (659 MHz, $CDCl_3$) of 2,6-anhydro-4,5,7-tri-*O*-benzyl-1,3-dideoxy-3-fluoro-D-*altro*-hept-1-enitol (**16**).

3.2.19 4,5,7-Tri-*O*-benzyl-3-deoxy-3-fluoro-D-*glycero*-D-*arabino*-hept-2-ulopyranose (17)

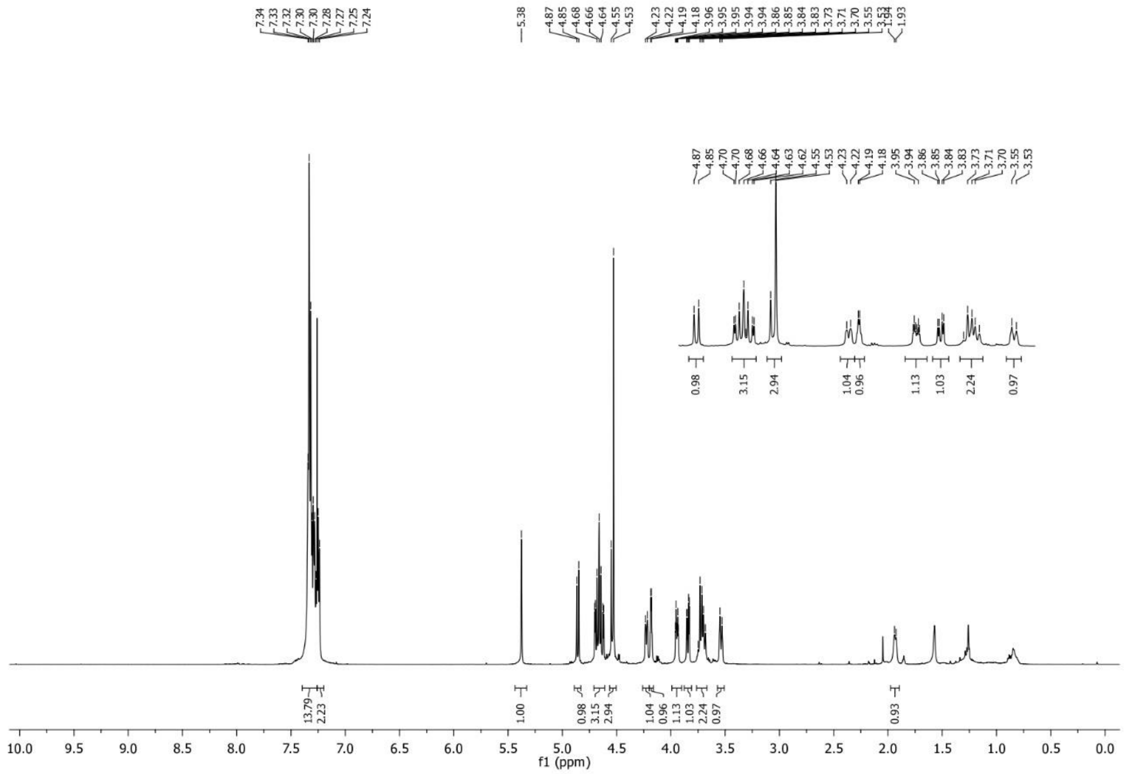

Figure S75: ^{1}H NMR (600 MHz, $CDCl_3$) of 4,5,7-tri-O-benzyl-3-deoxy-3-fluoro-D-*glycero*-D-*arabino*-hept-2-ulopyranose (**17**).

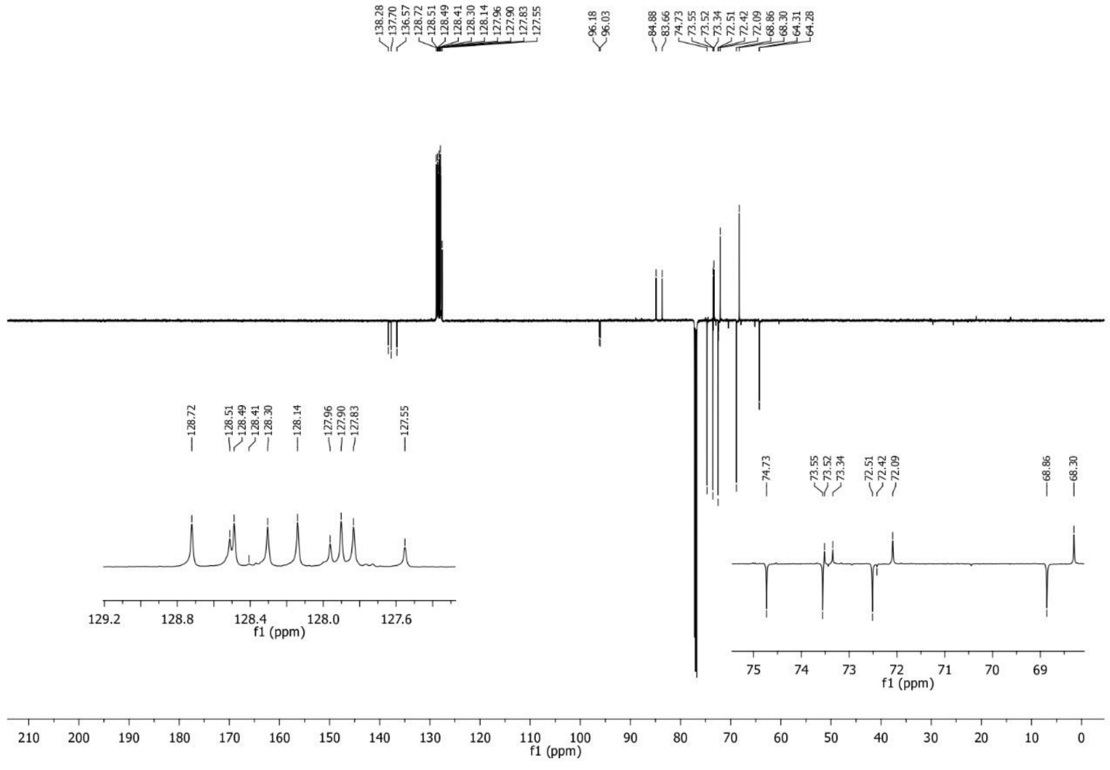

Figure S76: ^{13}C NMR (151 MHz, $CDCl_3$) of 4,5,7-tri-O-benzyl-3-deoxy-3-fluoro-D-*glycero*-D-*arabino*-hept-2-ulopyranose (**17**).

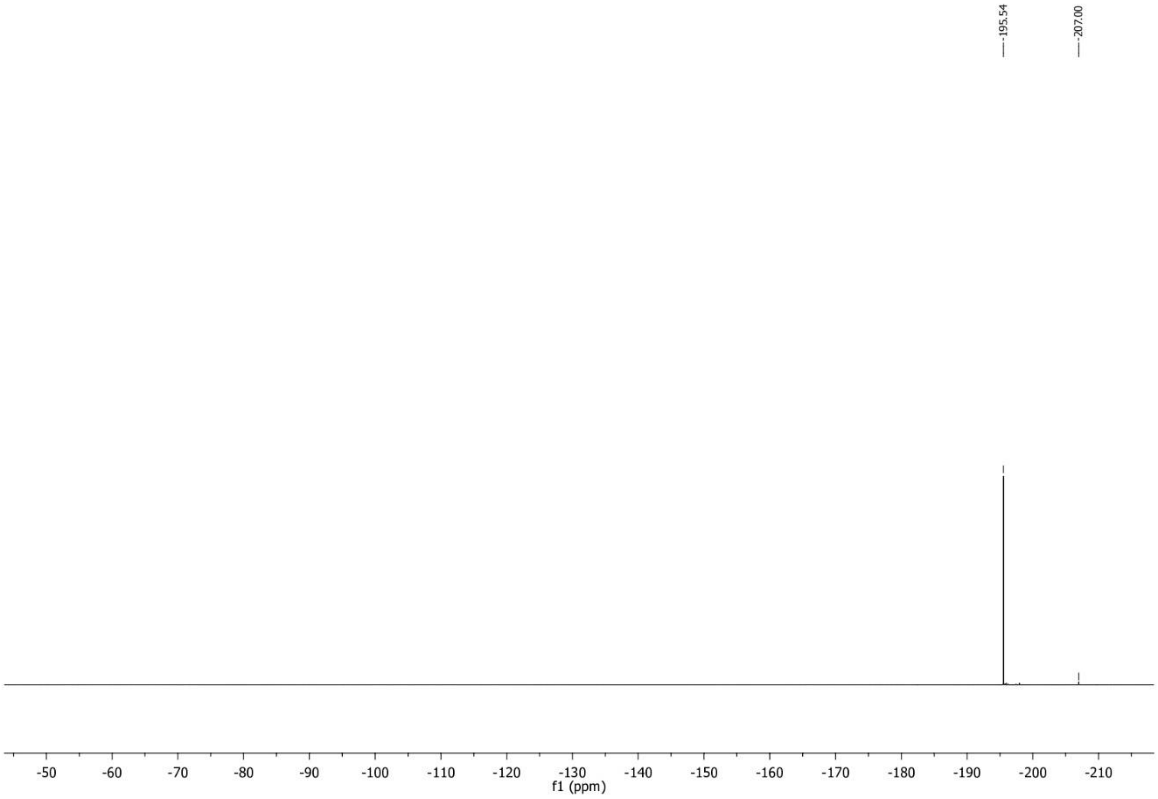

Figure S77: $^{19}F\{^{1}H\}$ NMR (659 MHz, $CDCl_3$) of 4,5,7-tri-O-benzyl-3-deoxy-3-fluoro-D-*glycero*-D-*arabino*-hept-2-ulopyranose (**17**).

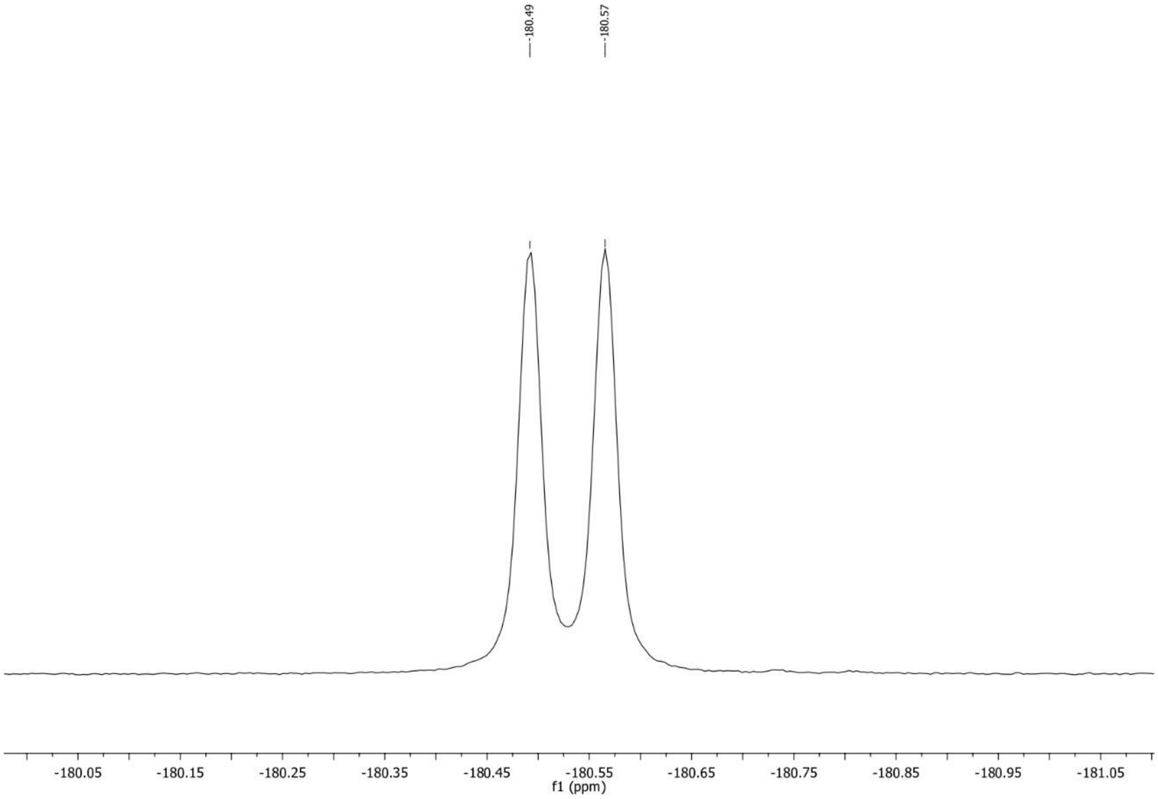

Figure S78: ^{19}F NMR (659 MHz, $CDCl_3$) of 4,5,7-tri-O-benzyl-3-deoxy-3-fluoro-D-*glycero*-D-*arabino*-hept-2-ulopyranose (**17**).

3.2.20 3-Deoxy-3-fluoro-D-sedoheptulose/ 3-Deoxy-3-fluoro-D-*glycero*-D-*arabino*-hept-2-ulopyranose & 3-deoxy-3-fluoro-D-*glycero*-D-*arabino*-hept-2-ulofuranose (3DFS)

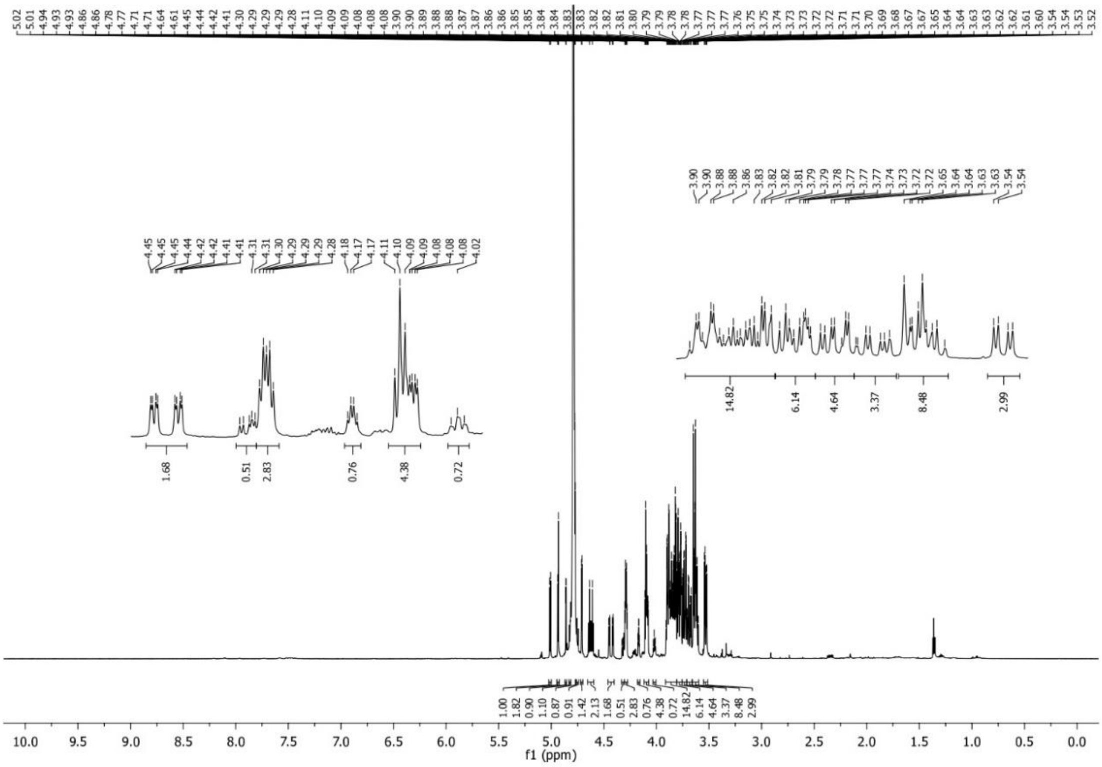

Figure S79: ^{1}H NMR (700 MHz, D_2O) of 3-deoxy-3-fluoro-D-sedoheptulose (**3DFS**).

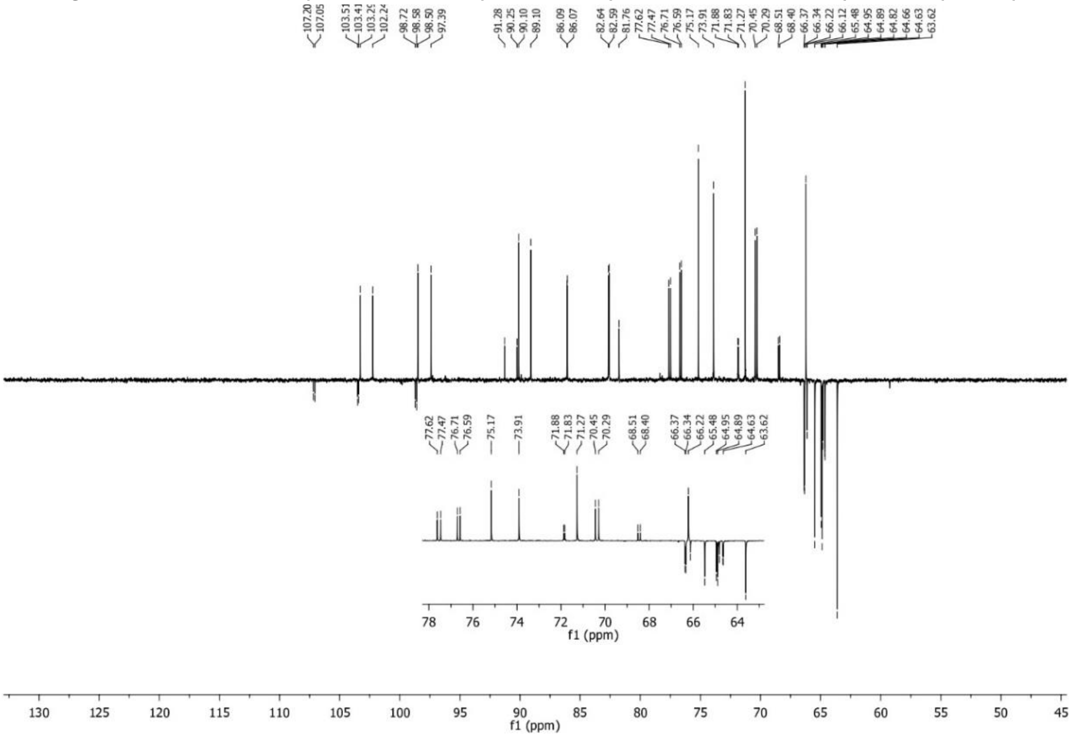

Figure S80: ^{13}C NMR (176 MHz, D_2O) of 3-deoxy-3-fluoro-D-sedoheptulose (**3DFS**).

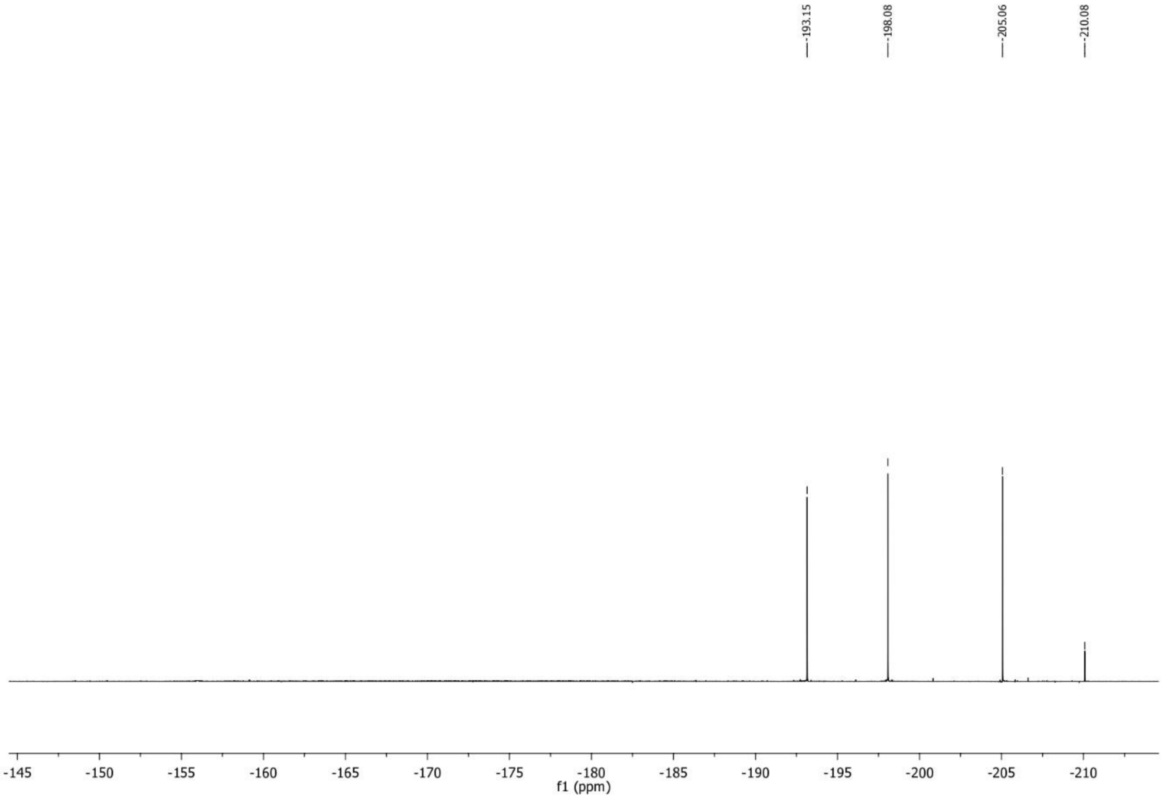

Figure S81: 19F{^{1}H} NMR (659 MHz, D_2O) of 3-deoxy-3-fluoro-D-sedoheptulose (**3DFS**).

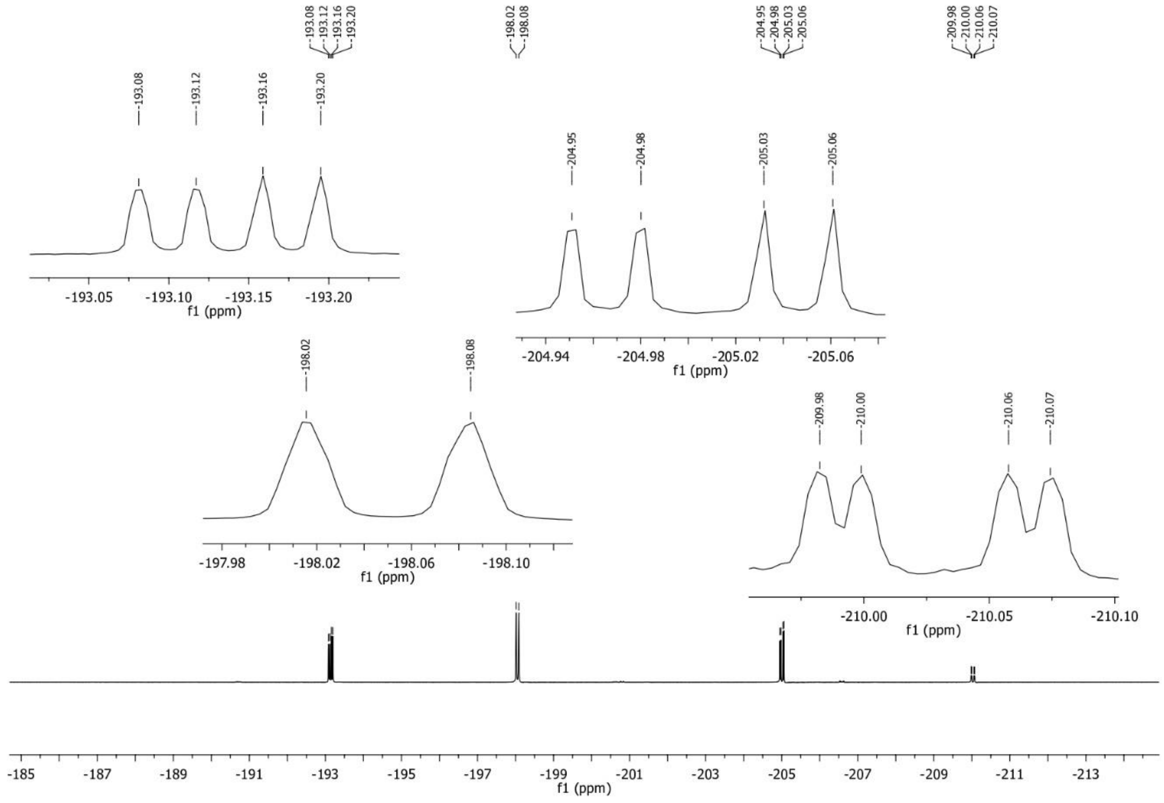

Figure S82: ^{19}F NMR (659 MHz, D_2O) of 3-deoxy-3-fluoro-D-sedoheptulose (**3DFS**).

3.2.21 2,7-Anhydro-4,5-*O*-isopropylidene-D-*glycero*-β-D-*arabino*-hept-2-ulopyranose (19)

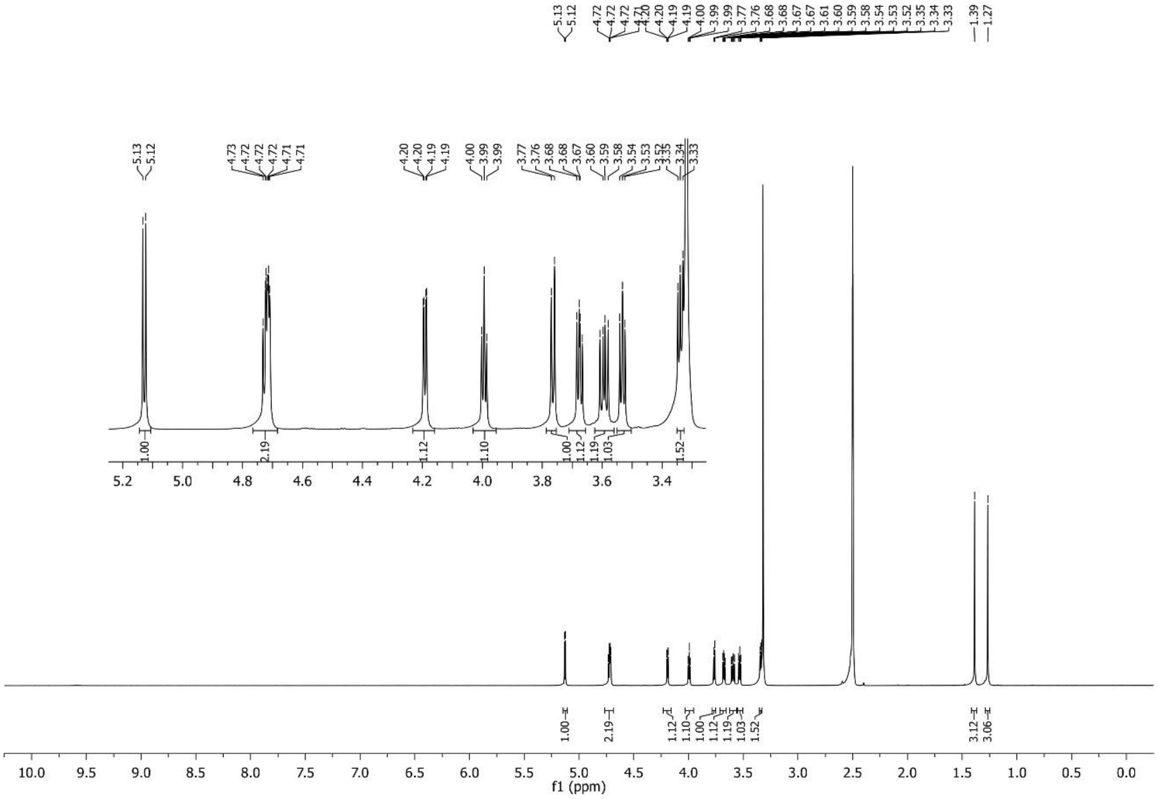

Figure S83: ^{1}H NMR (700 MHz, d_6-DMSO) of 2,7-anhydro-4,5-*O*-isopropylidene-D-*glycero*-β-D-*arabino*-hept-2-ulopyranose (**18**).

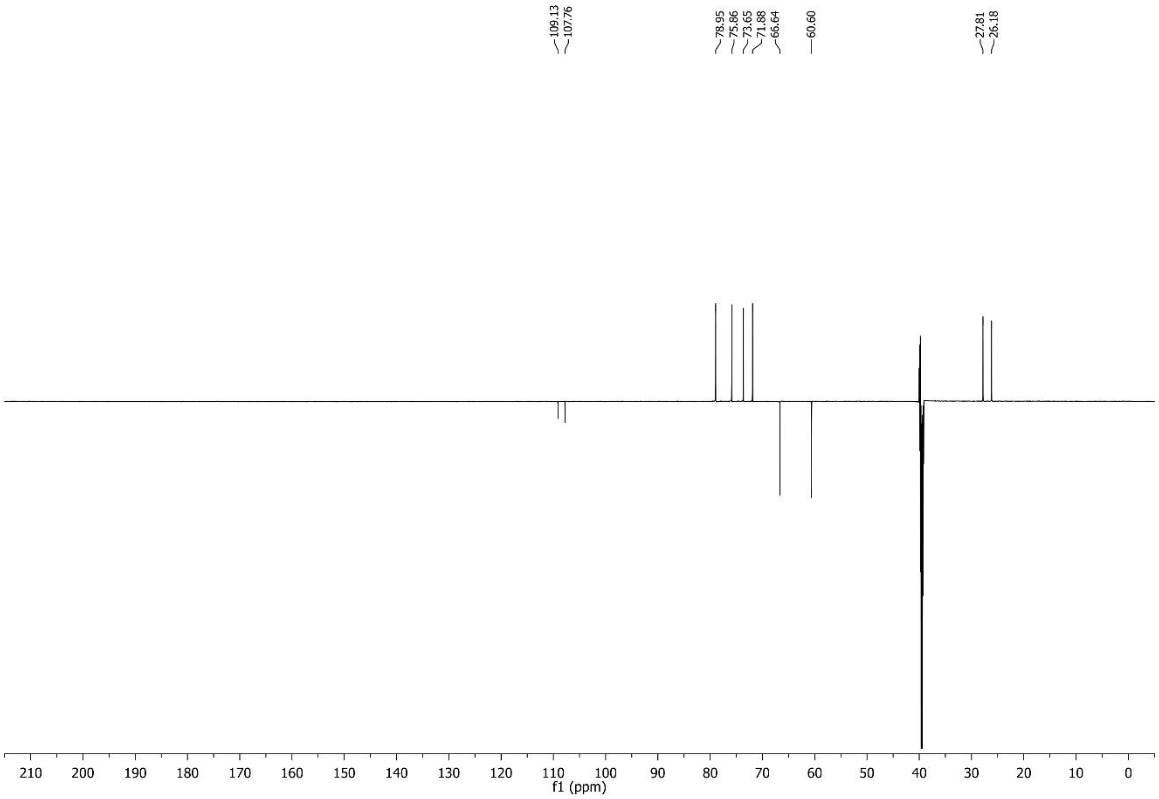

Figure S84: ^{13}C NMR (176 MHz, d_6-DMSO) of 2,7-anhydro-4,5-*O*-isopropylidene-D-*glycero*-β-D-*arabino*-hept-2-ulopyranose (**18**).

3.2.22 1,3-Di-*O*-acetyl-2,7-anhydro-4,5-*O*-isopropylidene-D-*glycero*-β-D-*arabino*-hept-2-ulopyranose (19)

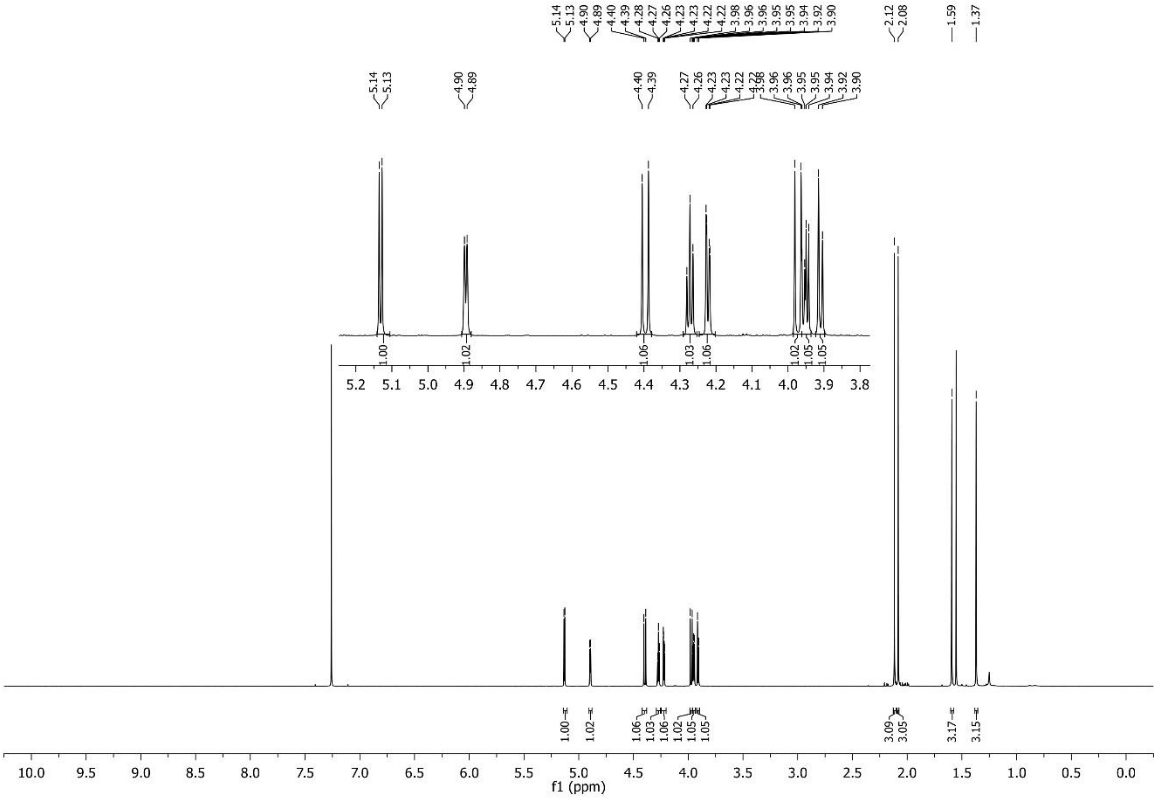

Figure S85: ^{1}H NMR (700 MHz, $CDCl_3$) of 1,3-di-*O*-acetyl-2,7-anhydro-4,5-*O*-isopropylidene-D-*glycero*-β-D-*arabino*-hept-2-ulopyranose (**19**).

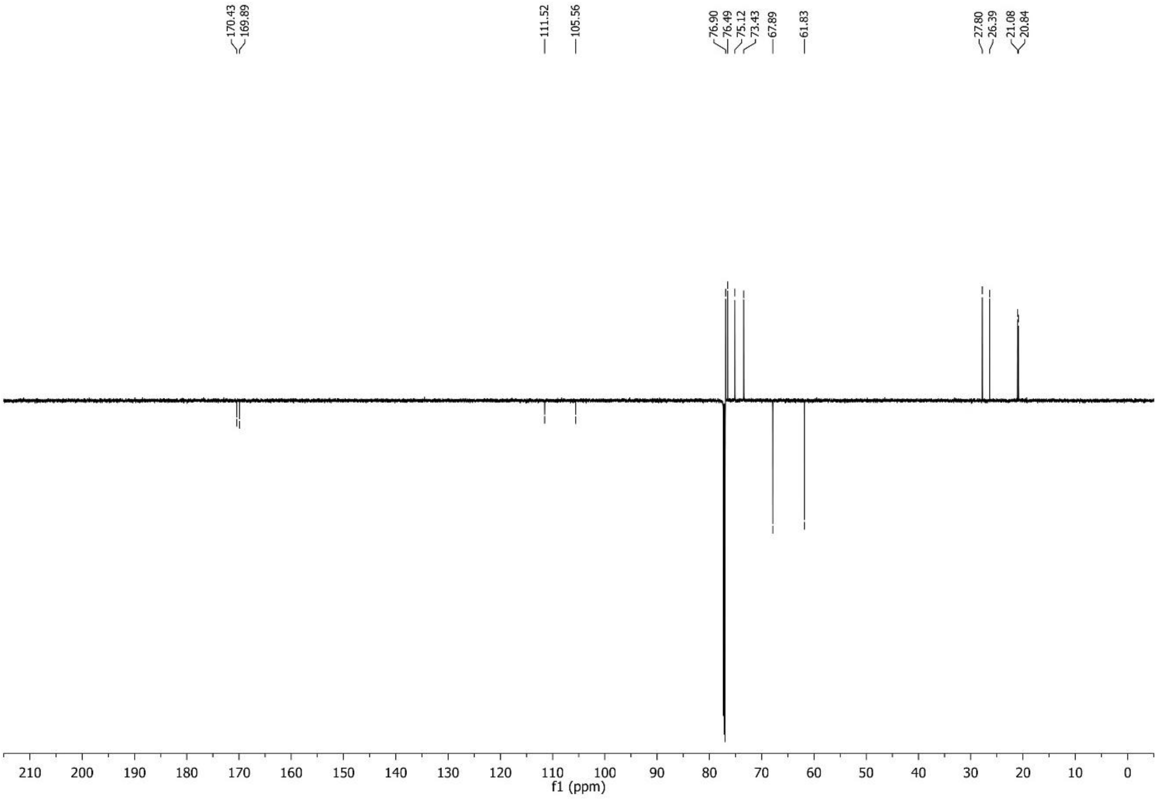

Figure S86: ^{13}C NMR (176 MHz, $CDCl_3$) of 1,3-di-*O*-acetyl-2,7-anhydro-4,5-*O*-isopropylidene-D-*glycero*-β-D-*arabino*-hept-2-ulopyranose (**19**).

3.2.23 1,3-Di-*O*-acetyl-2,7-anhydro-D-*glycero*-β-D-*arabino*-hept-2-ulopyranose (20)

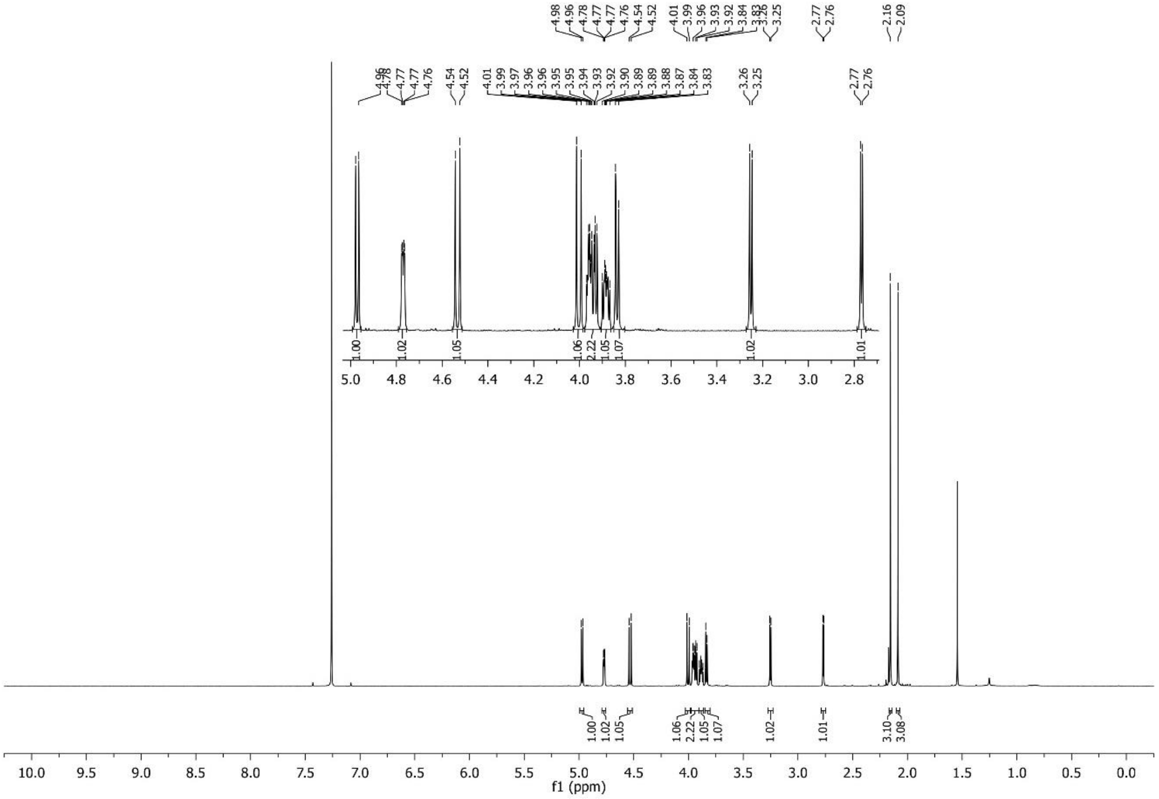

Figure S87: ^{1}H NMR (600 MHz, $CDCl_3$) of 1,3-di-*O*-acetyl-2,7-anhydro-D-*glycero*-β-D-*arabino*-hept-2-ulopyranose (**20**).

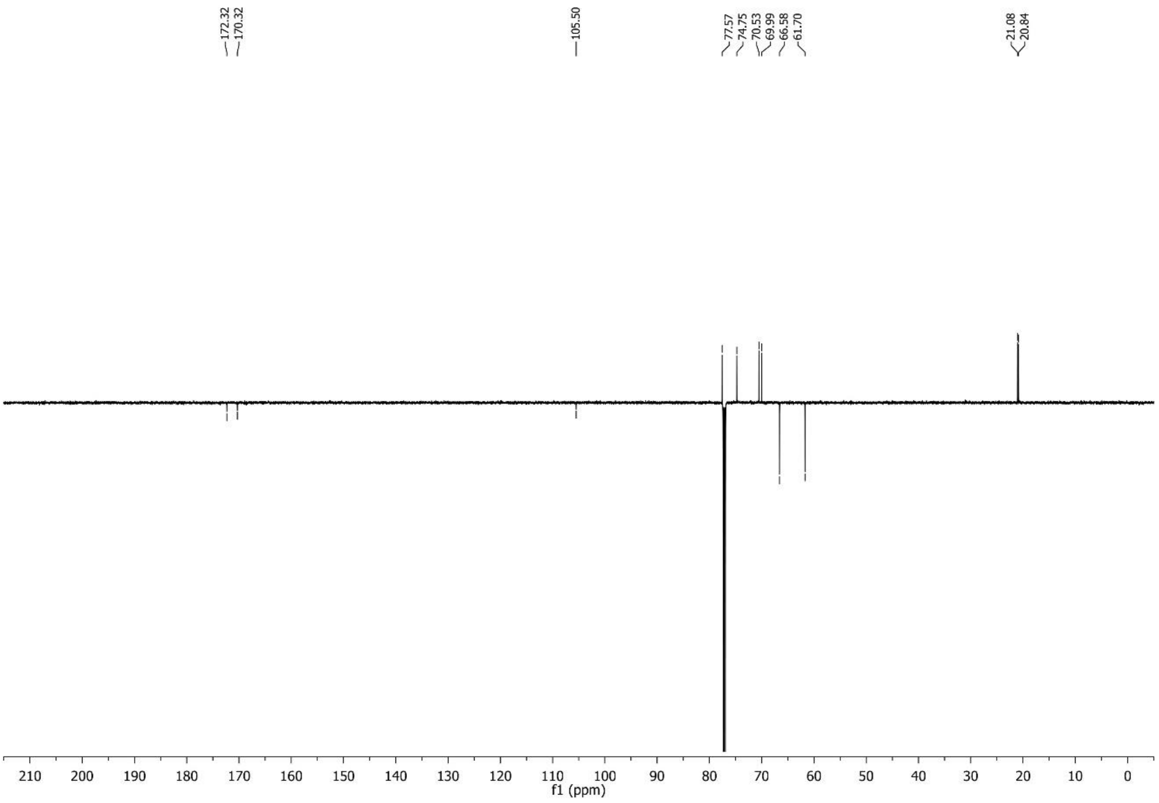

Figure S88: ^{13}C NMR (151 MHz, $CDCl_3$) of 1,3-di-*O*-acetyl-2,7-anhydro-D-*glycero*-β-D-*arabino*-hept-2-ulopyranose (**20**).

3.2.24 1,3-Di-*O*-acetyl-2,7-anhydro-4,5-*O*-benzylidene-D-*glycero*-β-D-*arabino*-hept-2-ulopyranose (21)

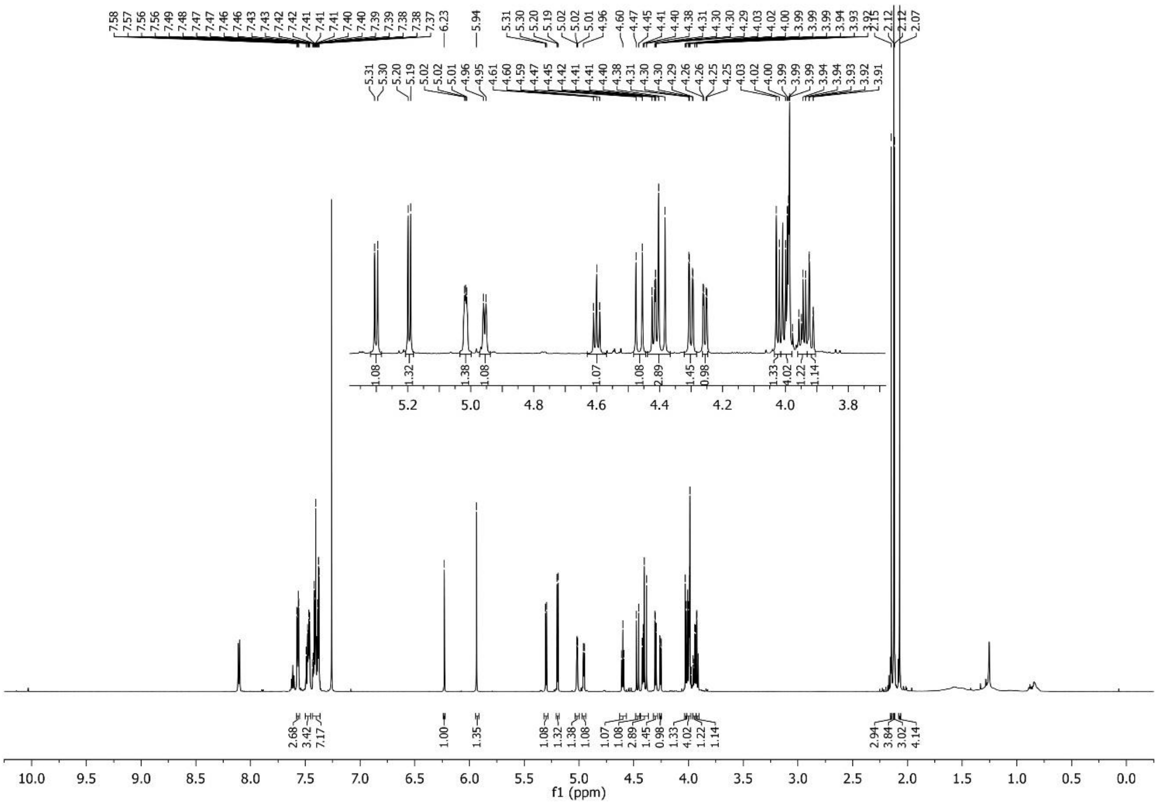

Figure S89: ^{1}H NMR (600 MHz, $CDCl_3$) of 1,3-di-*O*-acetyl-2,7-anhydro-4,5-*O*-benzylidene-D-*glycero*-β-D-*arabino*-hept-2-ulopyranose (**21**).

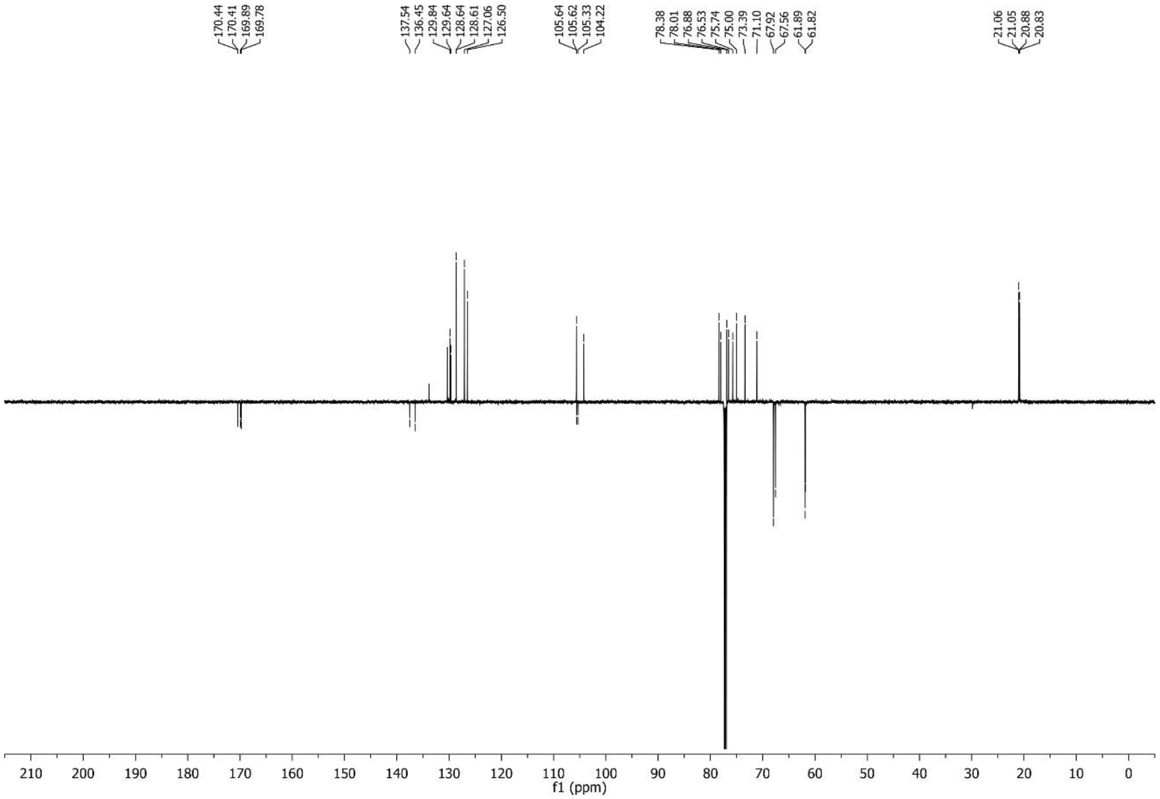

Figure S90: ^{13}C NMR (151 MHz, $CDCl_3$) of 1,3-di-*O*-acetyl-2,7-anhydro-4,5-*O*-benzylidene-D-*glycero*-β-D-*arabino*-hept-2-ulopyranose (**21**).

3.2.25 1,3-Di-*O*-acetyl-2,7-anhydro-5-*O*-benzoyl-D-*glycero*-β-D-*arabino*-hept-2-ulopyranose (22)

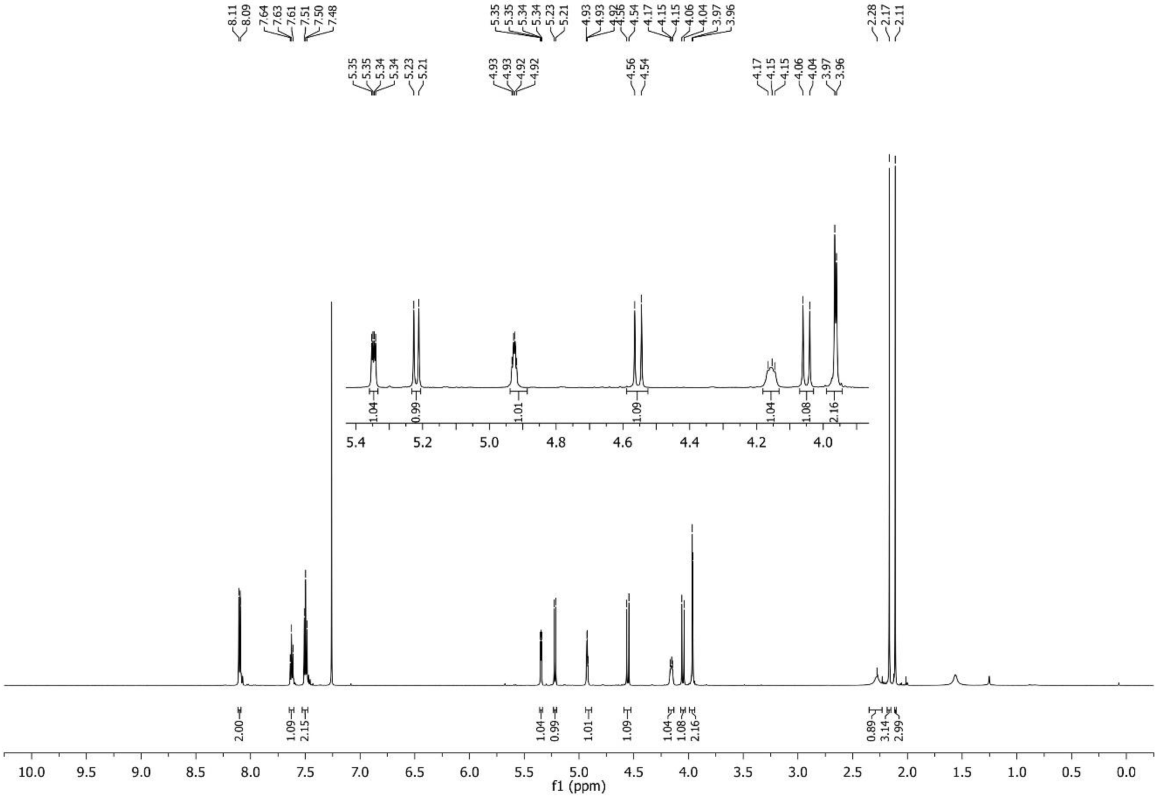

Figure S91: ^{1}H NMR (600 MHz, $CDCl_3$) of 1,3-di-*O*-acetyl-2,7-anhydro-5-*O*-benzoyl-D-*glycero*-β-D-*arabino*-hept-2-ulopyranose (**22**).

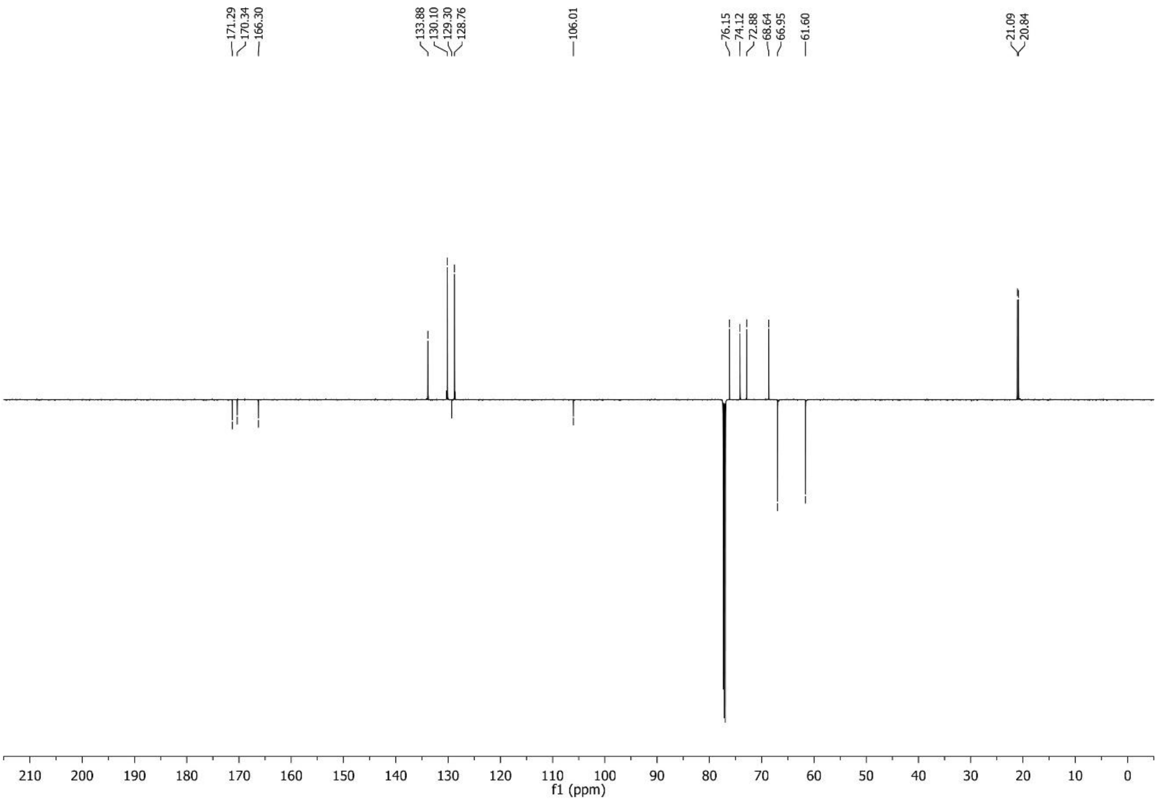

Figure S92: ^{13}C NMR (151 MHz, $CDCl_3$) of 1,3-di-*O*-acetyl-2,7-anhydro-5-*O*-benzoyl-D-*glycero*-β-D-*arabino*-hept-2-ulopyranose (**22**).

3.2.26 1,3-Di-*O*-acetyl-2,7-anhydro-5-*O*-benzoyl-4-trifluoromethanesulfonyl-D-*glycero*-β-D-*arabino*-hept-2-ulopyranose (23)

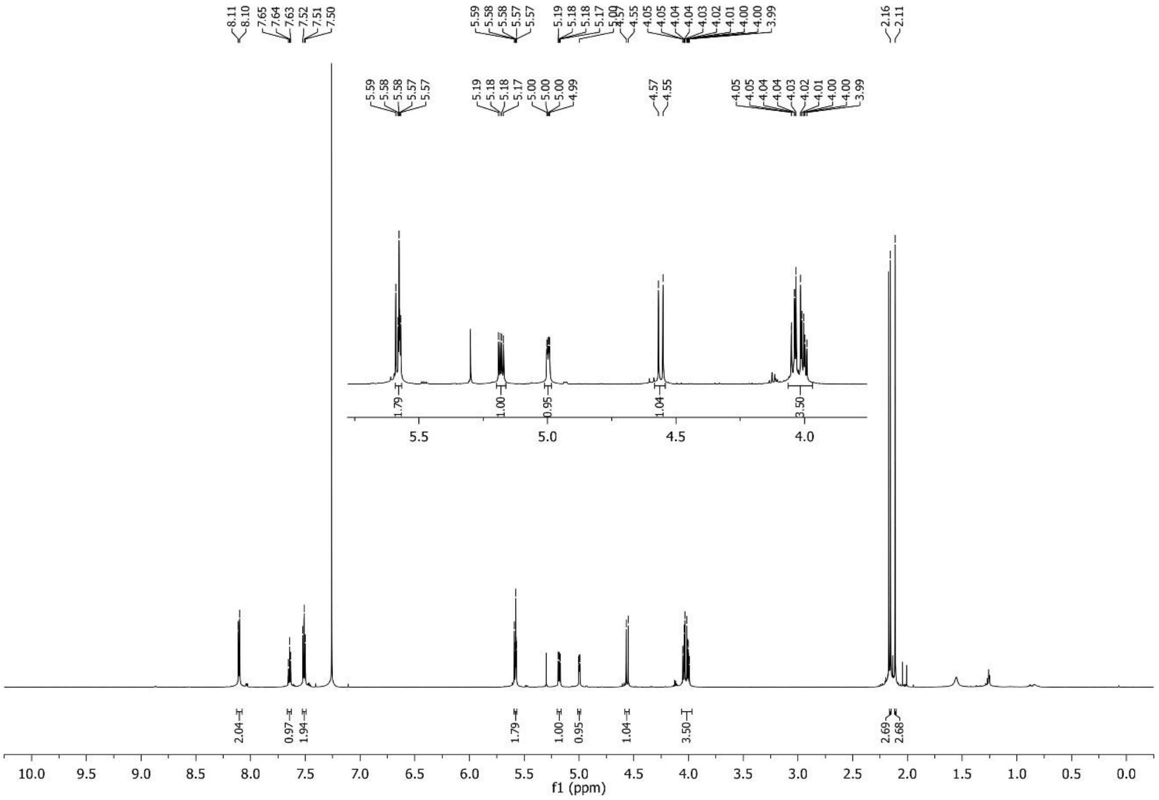

Figure S93: ^{1}H NMR (700 MHz, $CDCl_3$) of 1,3-di-*O*-acetyl-2,7-anhydro-5-*O*-benzoyl-4-trifluoromethanesulfonyl-D-*glycero*-β-D-*arabino*-hept-2-ulopyranose (**23**).

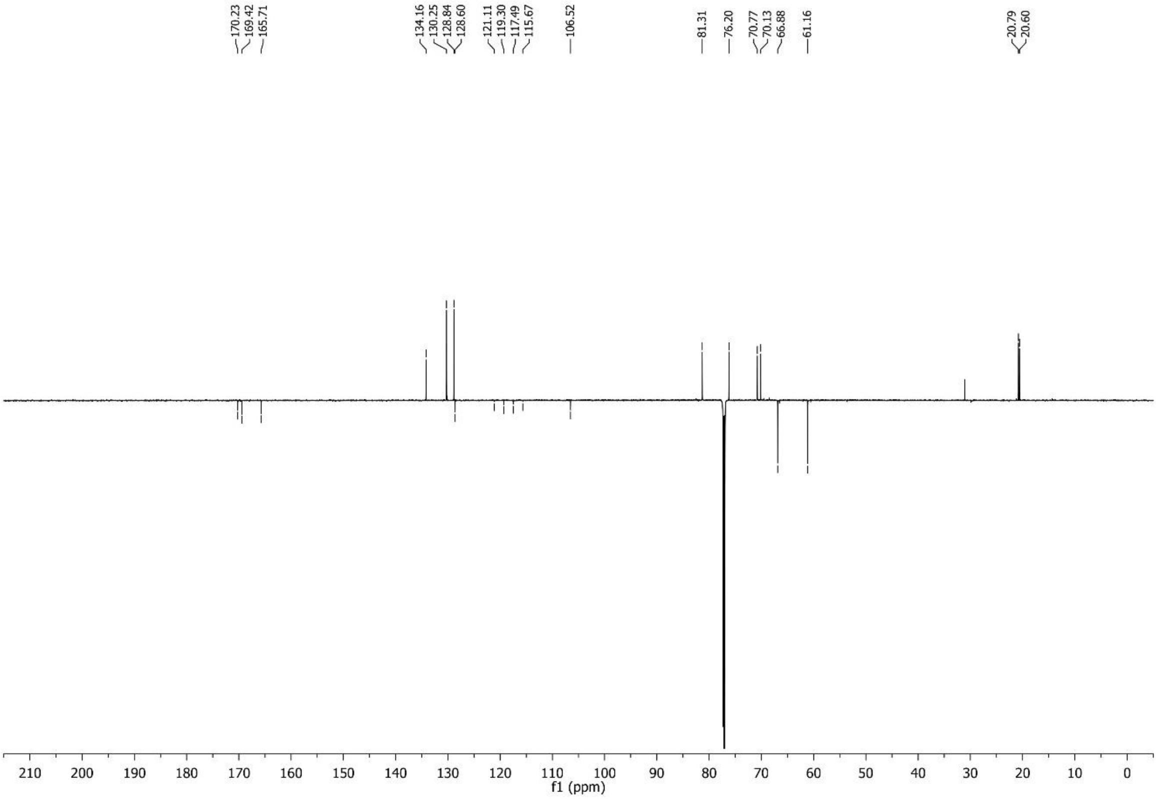

Figure S94: ^{13}C NMR (176 MHz, $CDCl_3$) of 1,3-di-*O*-acetyl-2,7-anhydro-5-*O*-benzoyl-4-trifluoromethanesulfonyl-D-*glycero*-β-D-*arabino*-hept-2-ulopyranose (**23**).

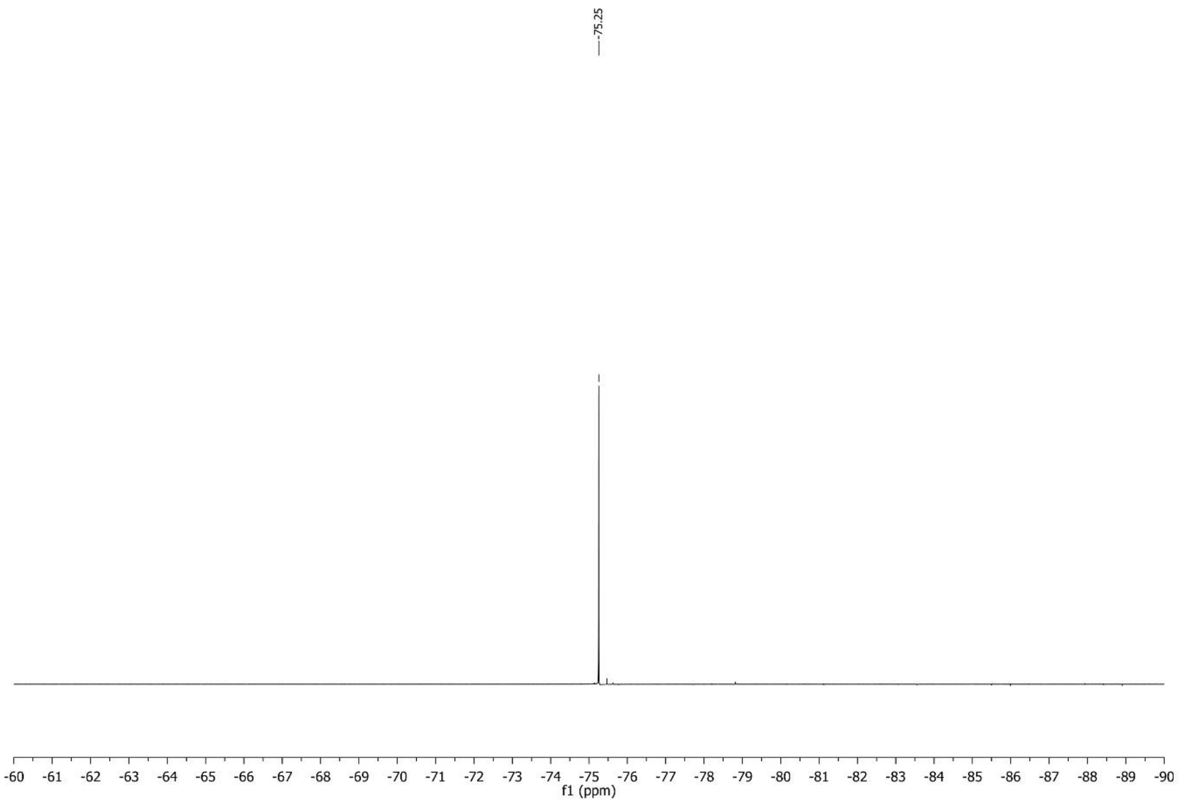

Figure S95: ^{19}F NMR (659 MHz, $CDCl_3$) of 1,3-di-*O*-acetyl-2,7-anhydro-5-*O*-benzoyl-4-trifluoromethanesulfonyl-D-*glycero*-β-D-*arabino*-hept-2-ulopyranose (**23**).

3.2.27 1,4-Di-*O*-acetyl-2,7-anhydro-5-*O*-benzoyl-D-*glycero*-β-D-*lyxo*-hept-2-ulopyranose (24a)

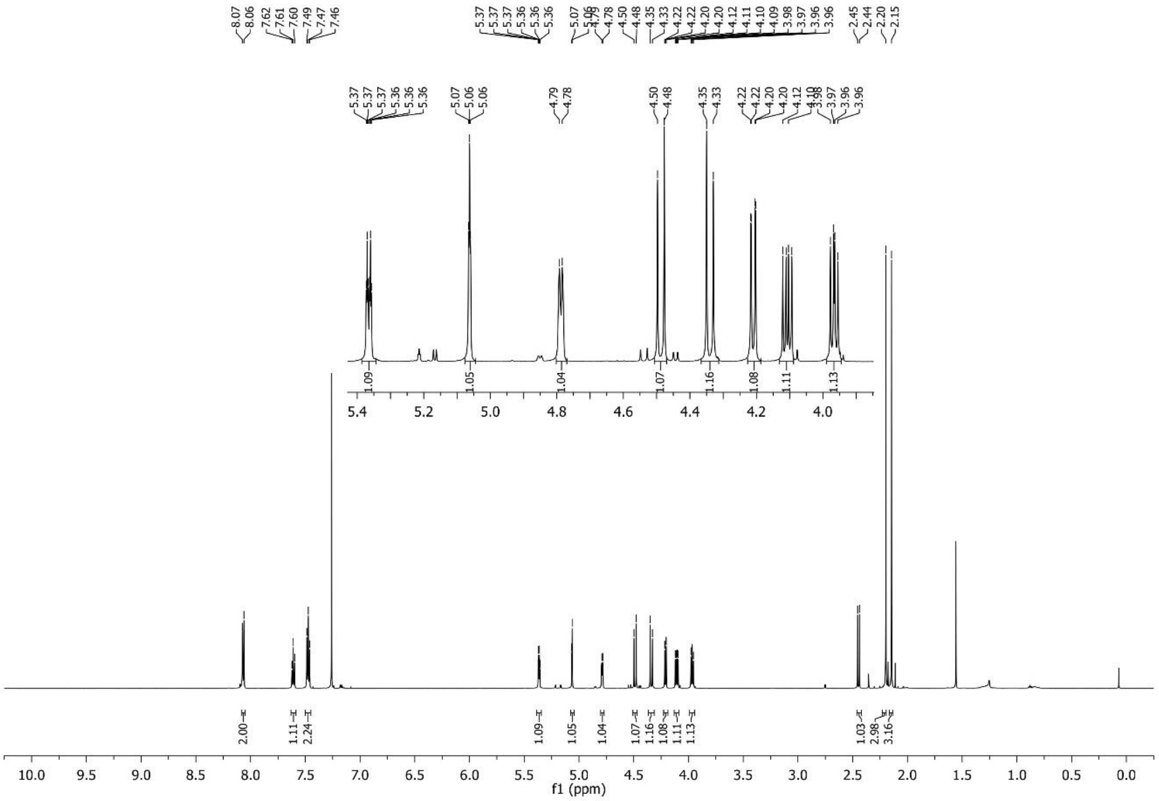

Figure S96: ^{1}H NMR (600 MHz, $CDCl_3$) of 1,4-di-*O*-acetyl-2,7-anhydro-5-*O*-benzoyl-D-*glycero*-β-D-*lyxo*-hept-2-ulopyranose (**24a**).

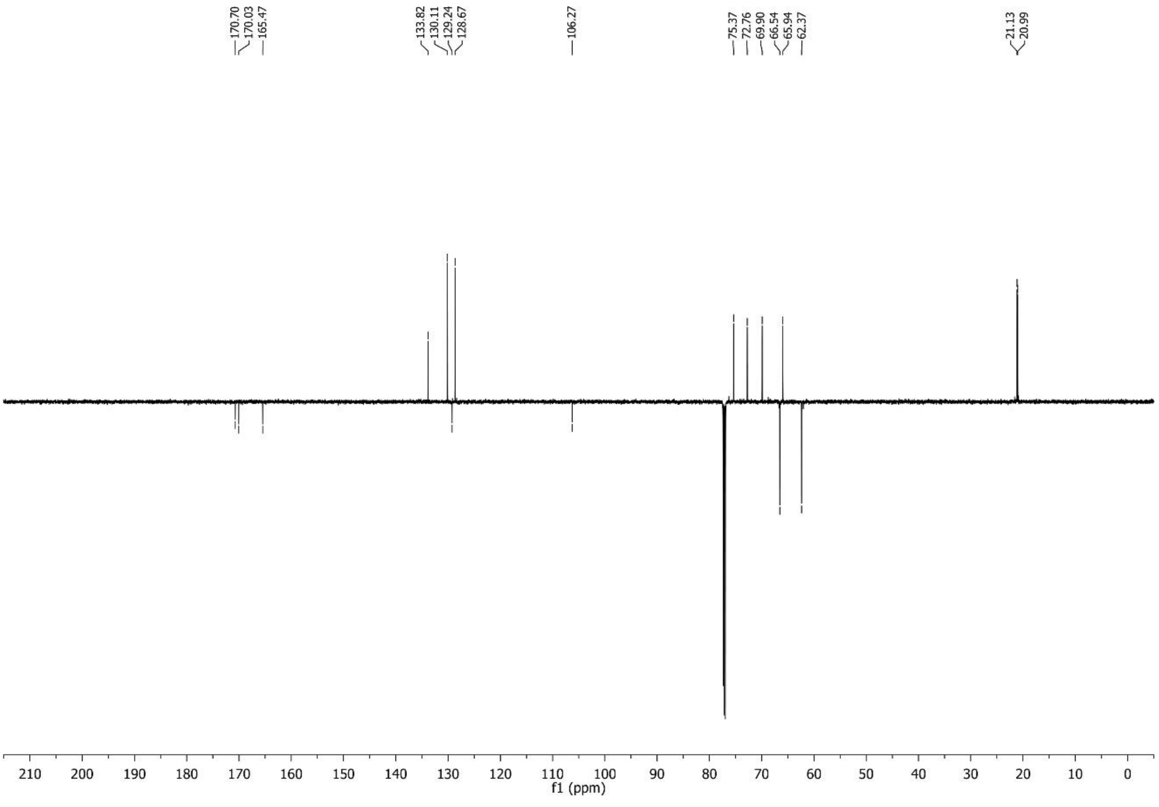

Figure S97: ^{13}C NMR (151 MHz, $CDCl_3$) of 1,4-di-*O*-acetyl-2,7-anhydro-5-*O*-benzoyl-D-*glycero*-β-D-*lyxo*-hept-2-ulopyranose (**24a**).

3.2.28 1,3-Di-*O*-acetyl-2,7-anhydro-5-*O*-benzoyl-D-*glycero*-β-D-*lyxo*-hept-2-ulopyranose (24b)

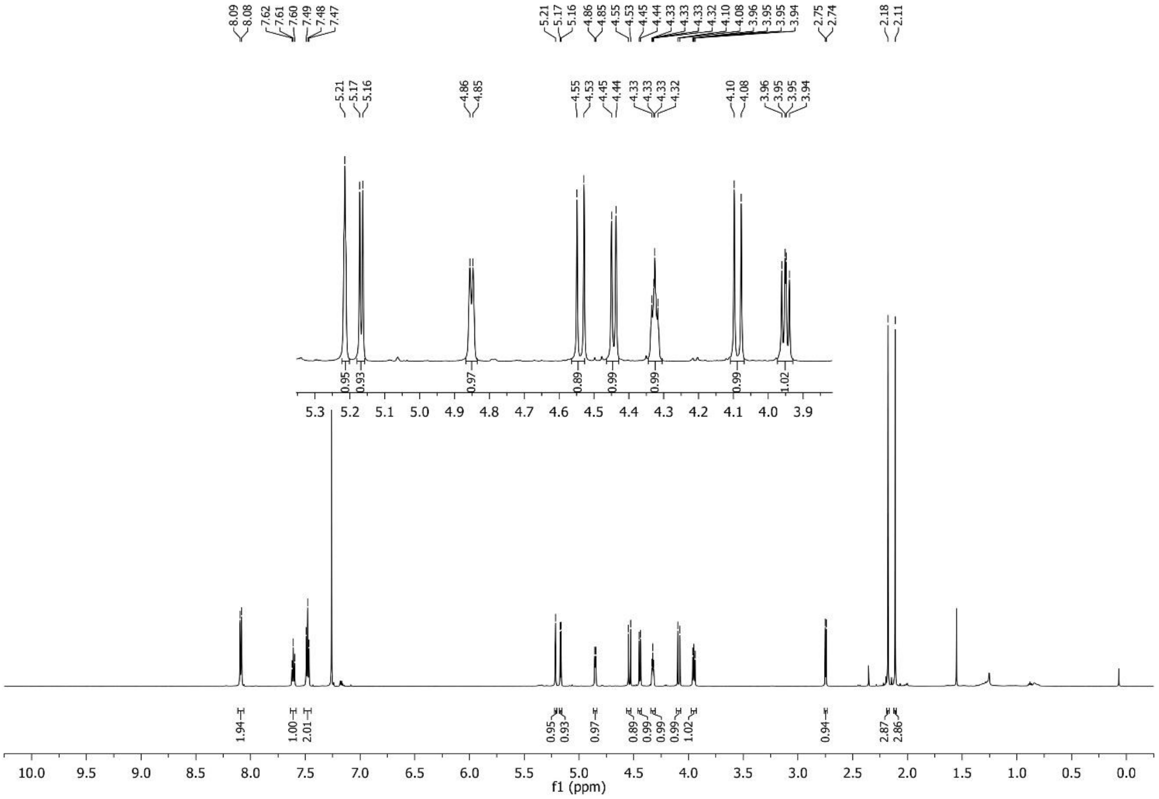

Figure S98: ^{1}H NMR (600 MHz, $CDCl_3$) of 1,3-di-*O*-acetyl-2,7-anhydro-5-*O*-benzoyl-D-*glycero*-β-D-*lyxo*-hept-2-ulopyranose (**24b**).

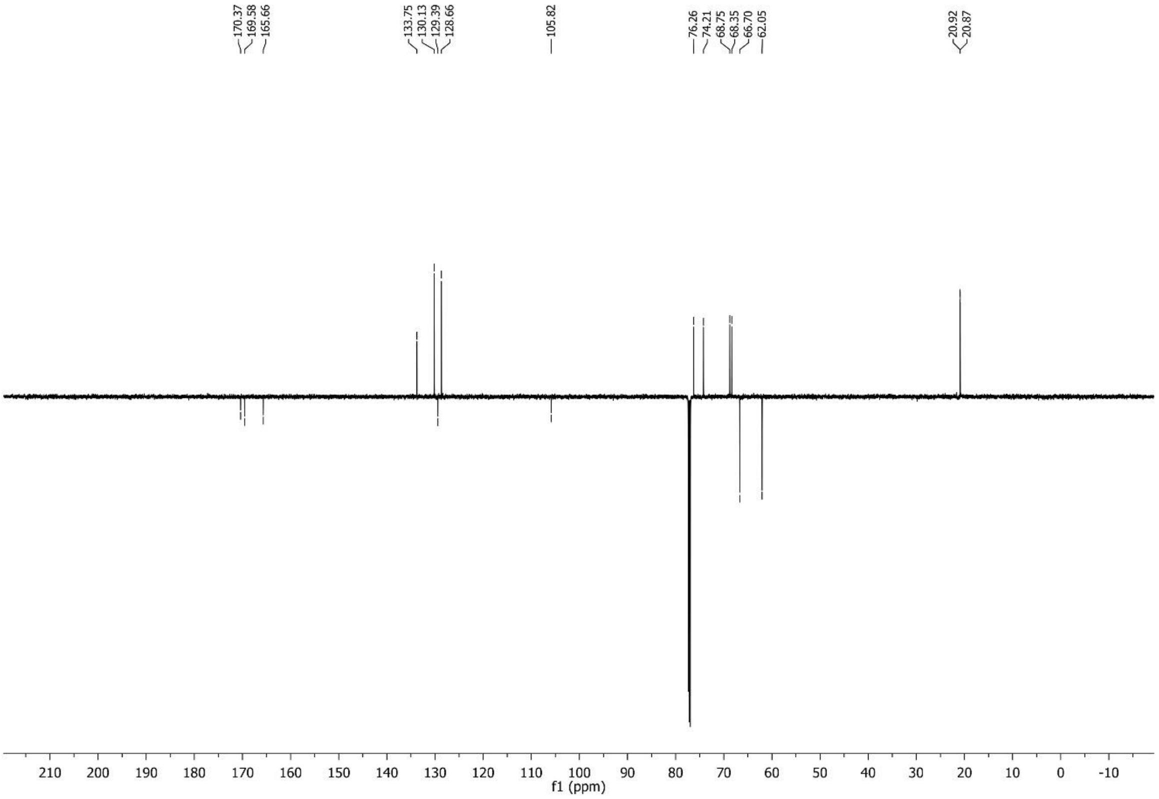

Figure S99: ^{13}C NMR (151 MHz, $CDCl_3$) of 1,3-di-*O*-acetyl-2,7-anhydro-5-*O*-benzoyl-D-*glycero*-β-D-*lyxo*-hept-2-ulopyranose (**24b**).

3.2.29 1,3-Di-*O*-acetyl-2,7-anhydro-5-*O*-benzoyl-4-*O*-chloroacetyl-D-*glycero*-β-D-*lyxo*-hept-2-ulopyranose (25)

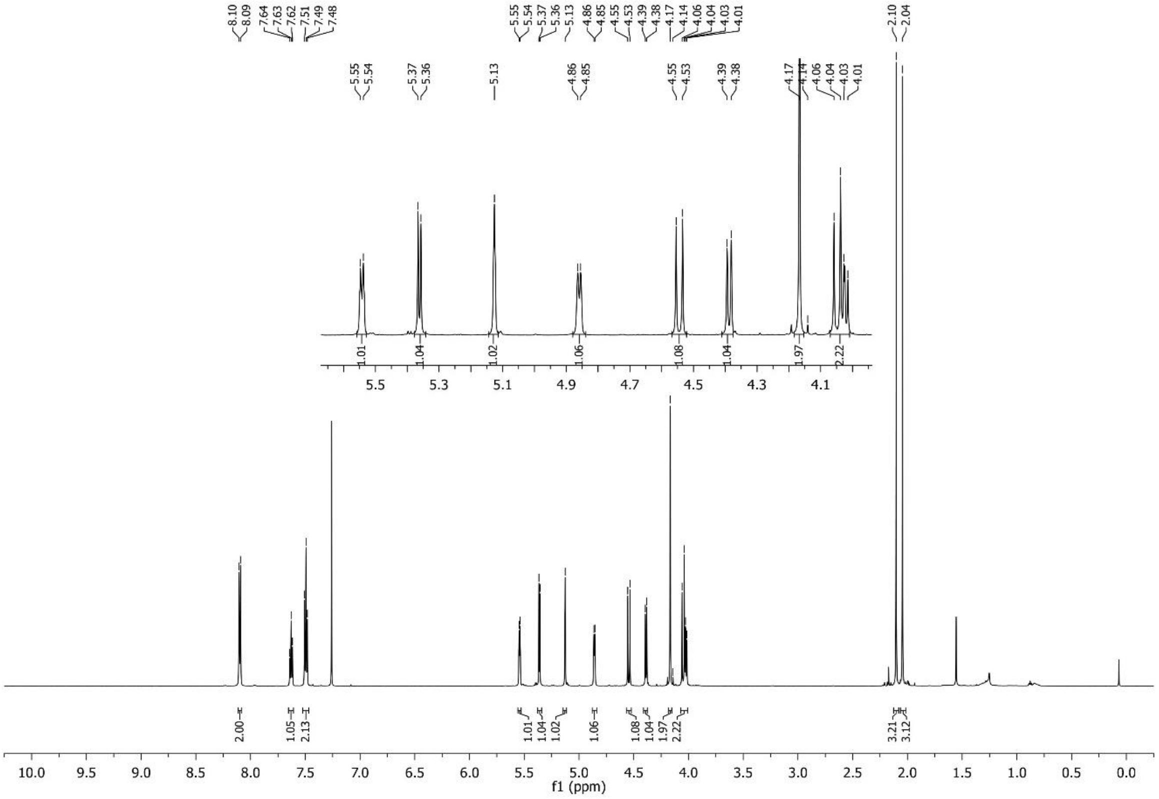

Figure S100: ^{1}H NMR (600 MHz, $CDCl_3$) of 1,3-di-*O*-acetyl-2,7-anhydro-5-*O*-benzoyl-4-*O*-chloroacetyl-D-*glycero*-β-D-*lyxo*-hept-2-ulopyranose (**25**).

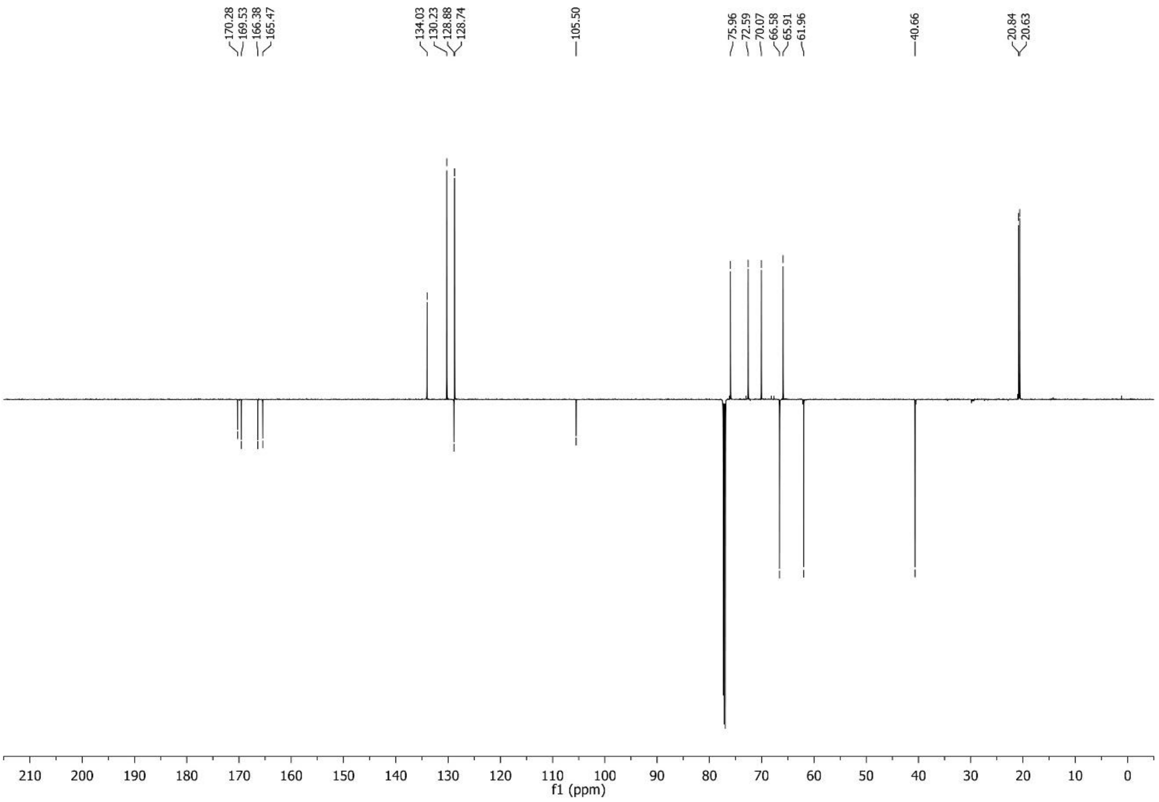

Figure S101: ^{13}C NMR (151 MHz, $CDCl_3$) of 1,3-di-*O*-acetyl-2,7-anhydro-5-*O*-benzoyl-4-*O*-chloroacetyl-D-*glycero*-β-D-*lyxo*-hept-2-ulopyranose (**25**).

3.2.30 1,2,3,7-Tetra-*O*-acetyl-5-*O*-benzoyl-4-*O*-chloroacetyl-D-*glycero*-α-D-*lyxo*-hept-2-ulopyranose (26)

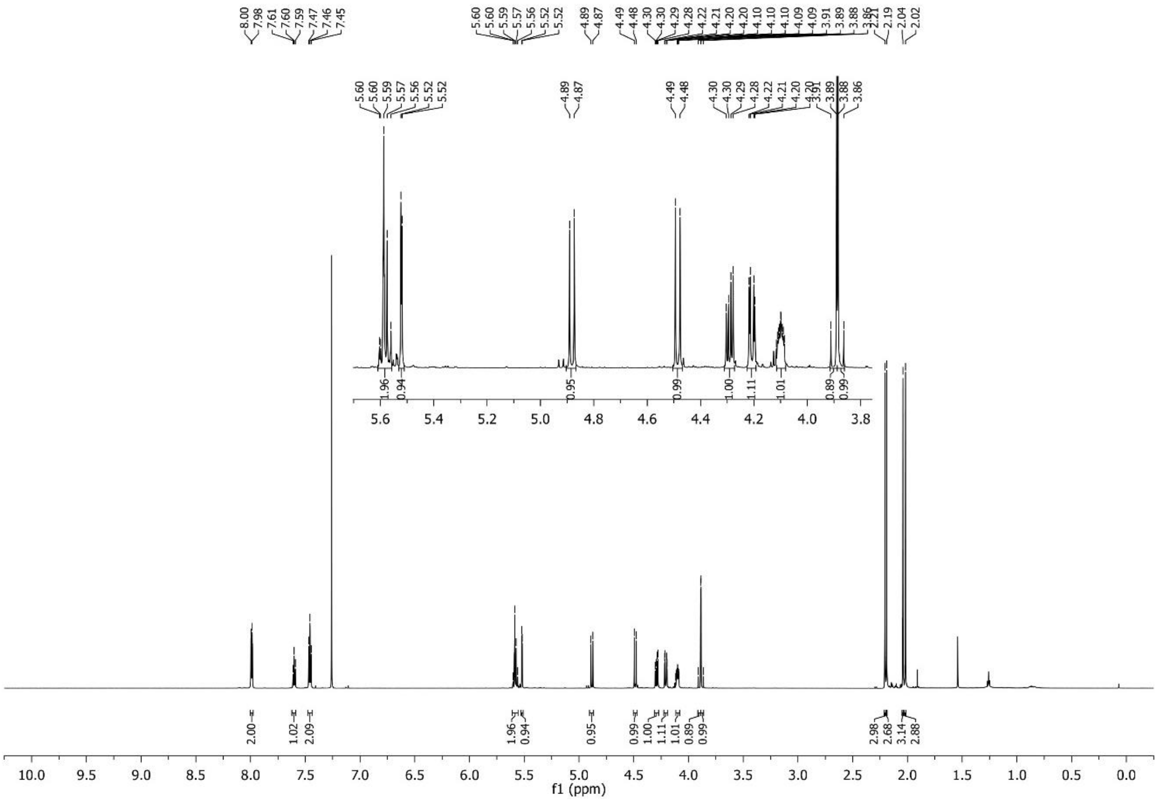

Figure S102: ^{1}H NMR (700 MHz, $CDCl_3$) of 1,2,3,7-tetra-*O*-acetyl-5-*O*-benzoyl-4-*O*-chloroacetyl-D-*glycero*-α-D-*lyxo*-hept-2-ulopyranose (**26**).

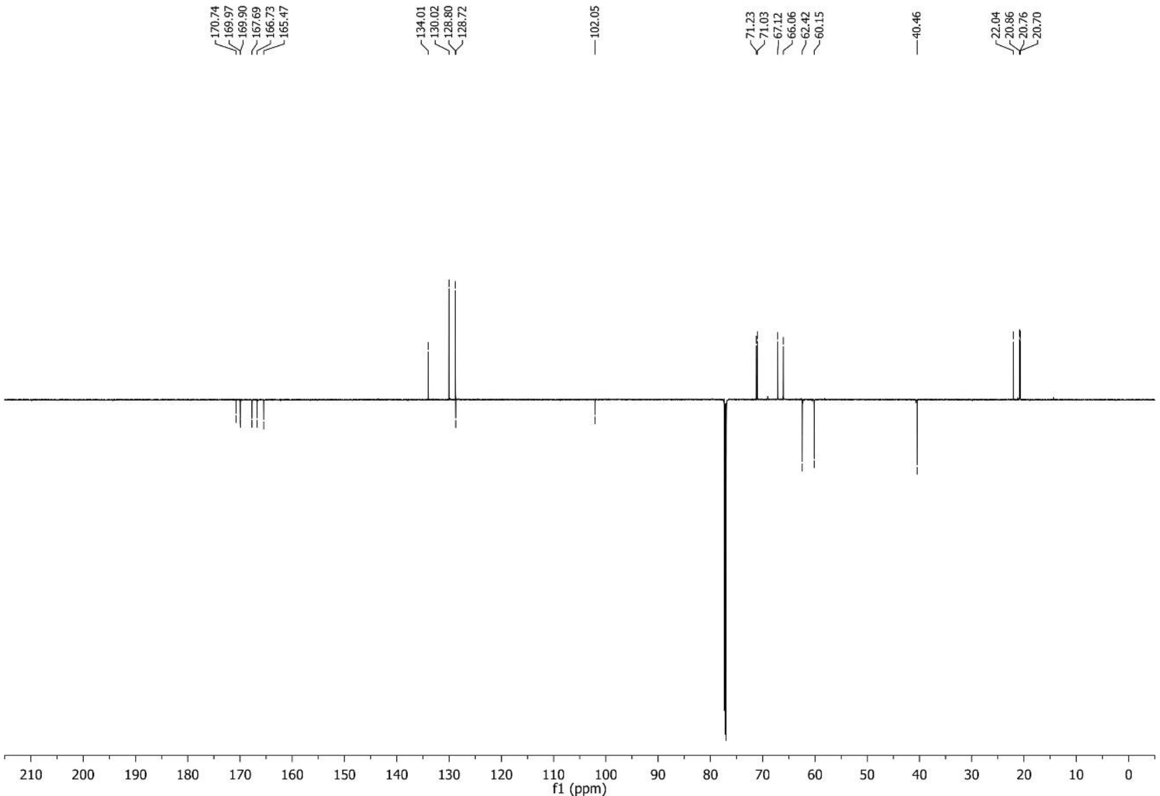

Figure S103: ^{13}C NMR (176 MHz, $CDCl_3$) of 1,2,3,7-tetra-*O*-acetyl-5-*O*-benzoyl-4-*O*-chloroacetyl-D-*glycero*-α-D-*lyxo*-hept-2-ulopyranose (**26**).

3.2.31 1,2,3,7-Tetra-*O*-acetyl-5-*O*-benzoyl-D-*glycero*-α-D-*lyxo*-hept-2-ulopyranose (27)

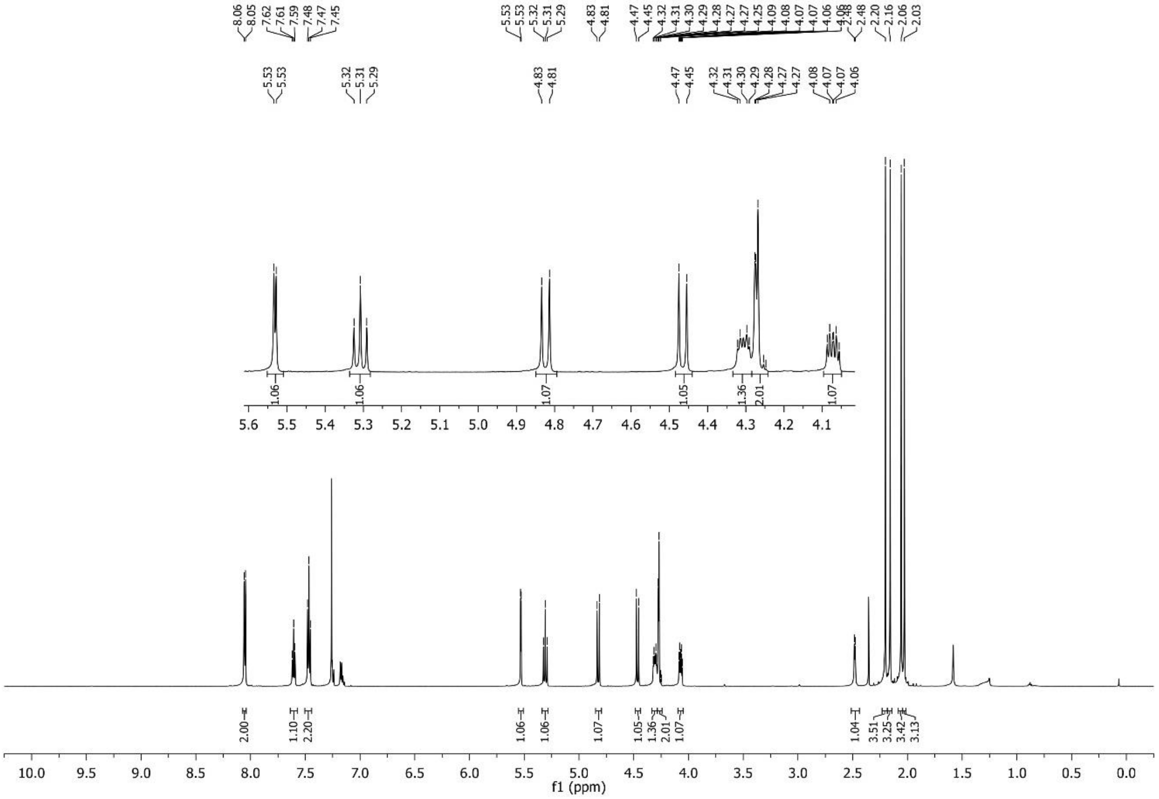

Figure S104: ^{1}H NMR (600 MHz, $CDCl_3$) of 1,2,3,7-tetra-*O*-acetyl-5-*O*-benzoyl-D-*glycero*-α-D-*lyxo*-hept-2-ulopyranose (**27**).

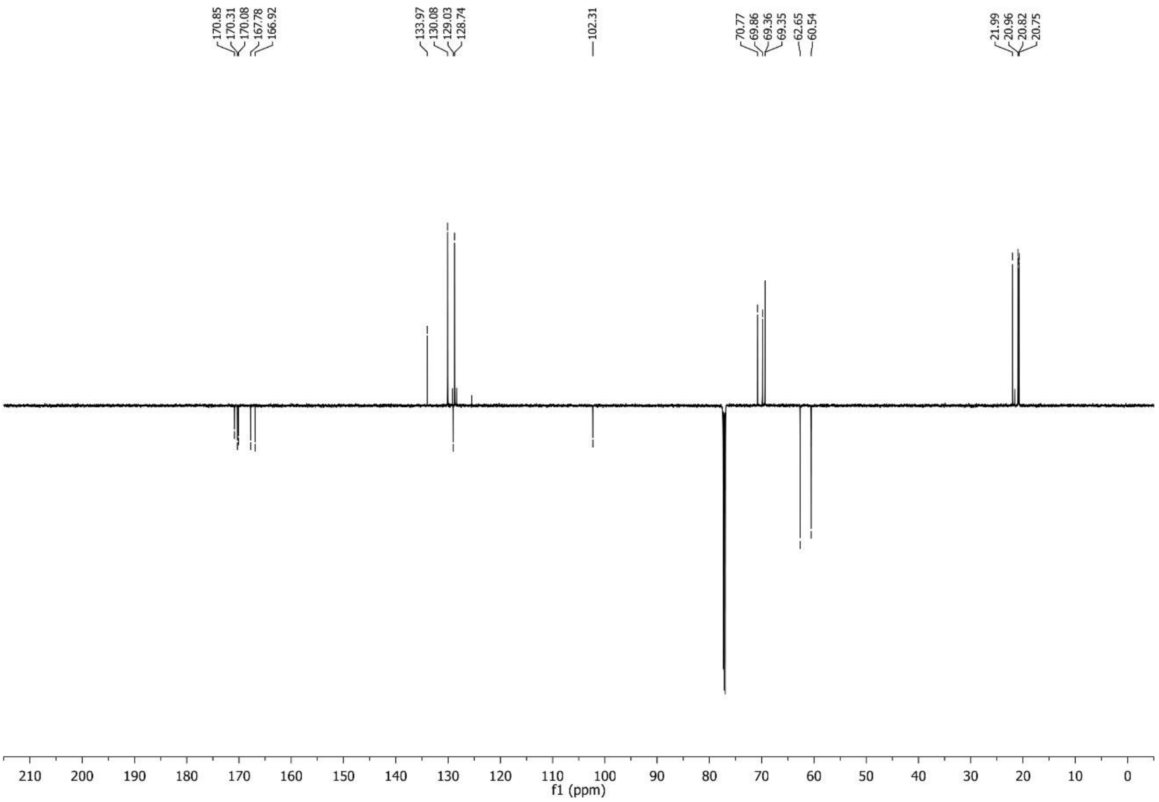

Figure S105: ^{13}C NMR (151 MHz, $CDCl_3$) of 1,2,3,7-tetra-*O*-acetyl-5-*O*-benzoyl-D-*glycero*-α-D-*lyxo*-hept-2-ulopyranose (**27**).

3.2.32 1,2,3,7-Tetra-*O*-acetyl-5-*O*-benzoyl-4-*O*-trifluoromethanesulfonyl-D-*glycero*-α-D-*lyxo*-hept-2-ulopyranose (28a)

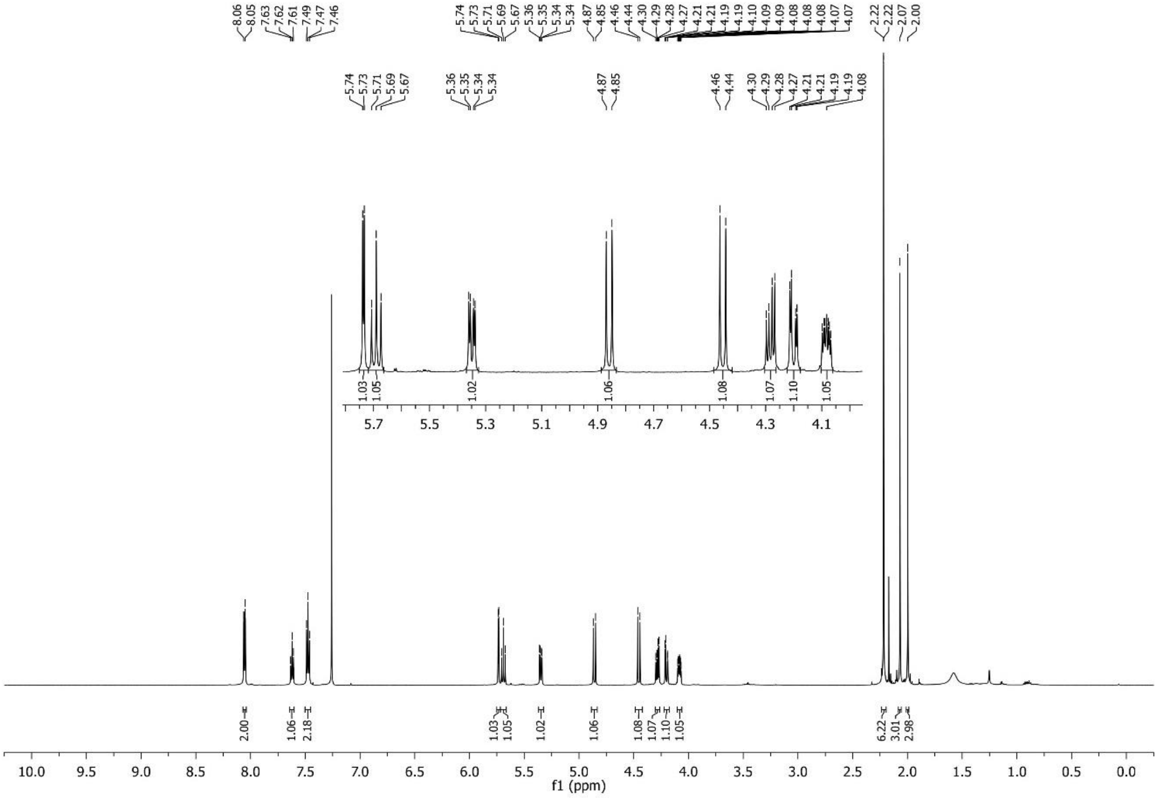

Figure S106: ^{1}H NMR (600 MHz, $CDCl_3$) of 1,2,3,7-tetra-*O*-acetyl-5-*O*-benzoyl-4-*O*-trifluoromethanesulfonyl-D-*glycero*-α-D-*lyxo*-hept-2-ulopyranose (**28a**).

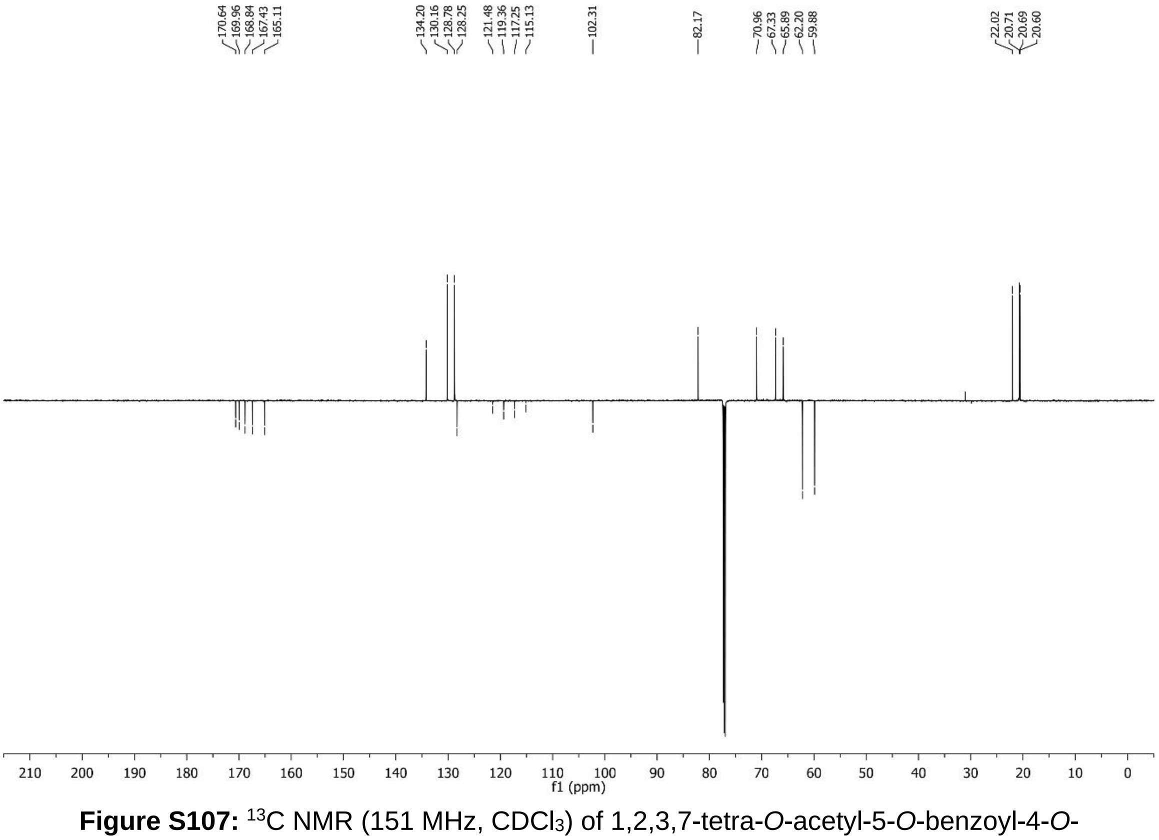

Figure S107: ^{13}C NMR (151 MHz, $CDCl_3$) of 1,2,3,7-tetra-*O*-acetyl-5-*O*-benzoyl-4-*O*-trifluoromethanesulfonyl-D-*glycero*-α-D-*lyxo*-hept-2-ulopyranose (**28a**).

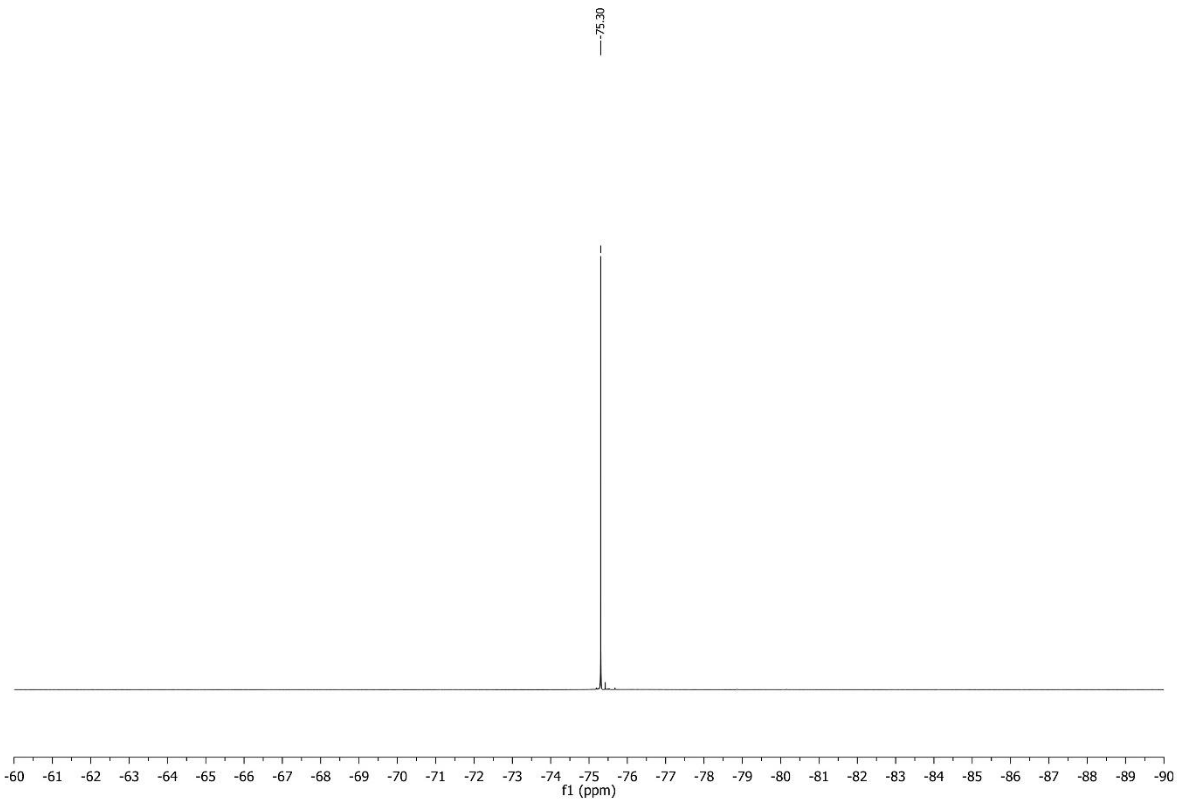

Figure S108: ^{19}F NMR (659 MHz, $CDCl_3$) of 1,2,3,7-tetra-*O*-acetyl-5-*O*-benzoyl-4-*O*-trifluoromethanesulfonyl-D-*glycero*-α-D-*lyxo*-hept-2-ulopyranose (**28a**).

3.2.33 1,2,3,7-Tetra-*O*-acetyl-5-*O*-benzoyl-4-*O*-4-nitrobenzenesulfonyl-D-*glycero*-α-D-*lxyo*-hept-2-ulopyranose (28b)

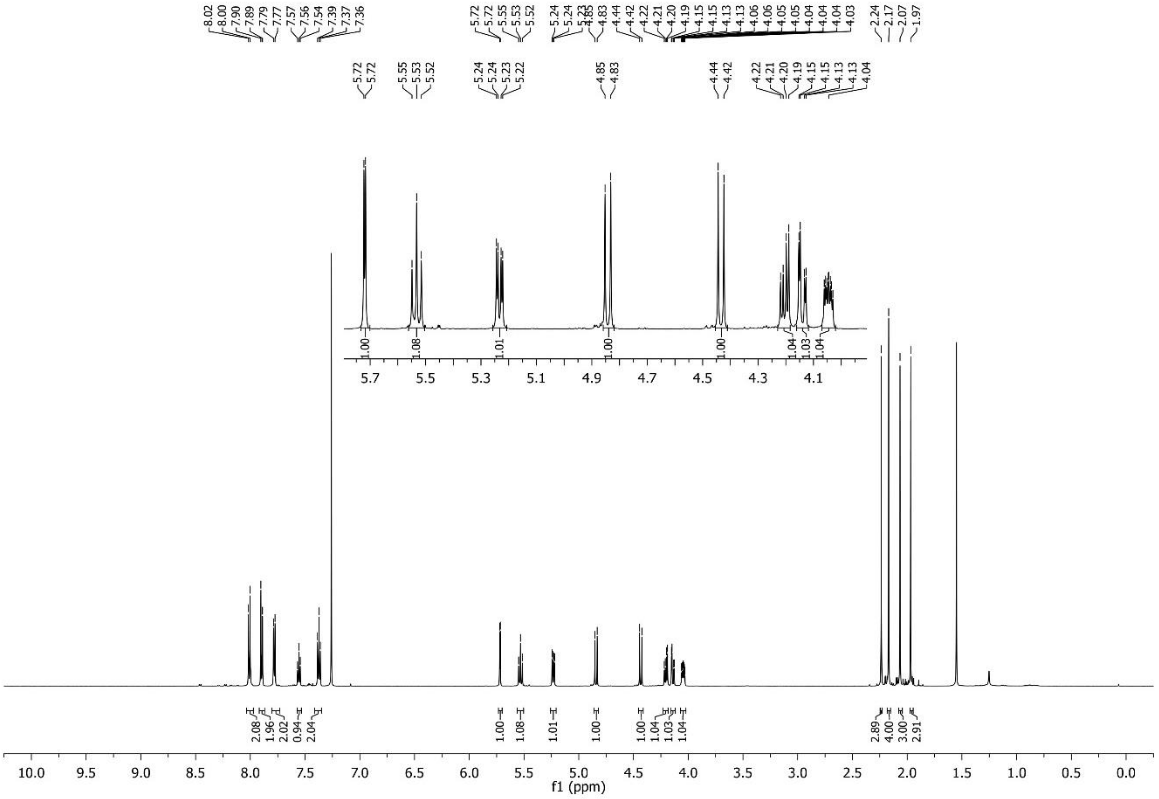

Figure S109: ^{1}H NMR (600 MHz, $CDCl_3$) of 1,2,3,7-tetra-*O*-acetyl-5-*O*-benzoyl-4-*O*-4-nitrobenzenesulfonyl-D-*glycero*-α-D-*lxyo*-hept-2-ulopyranose (**28b**).

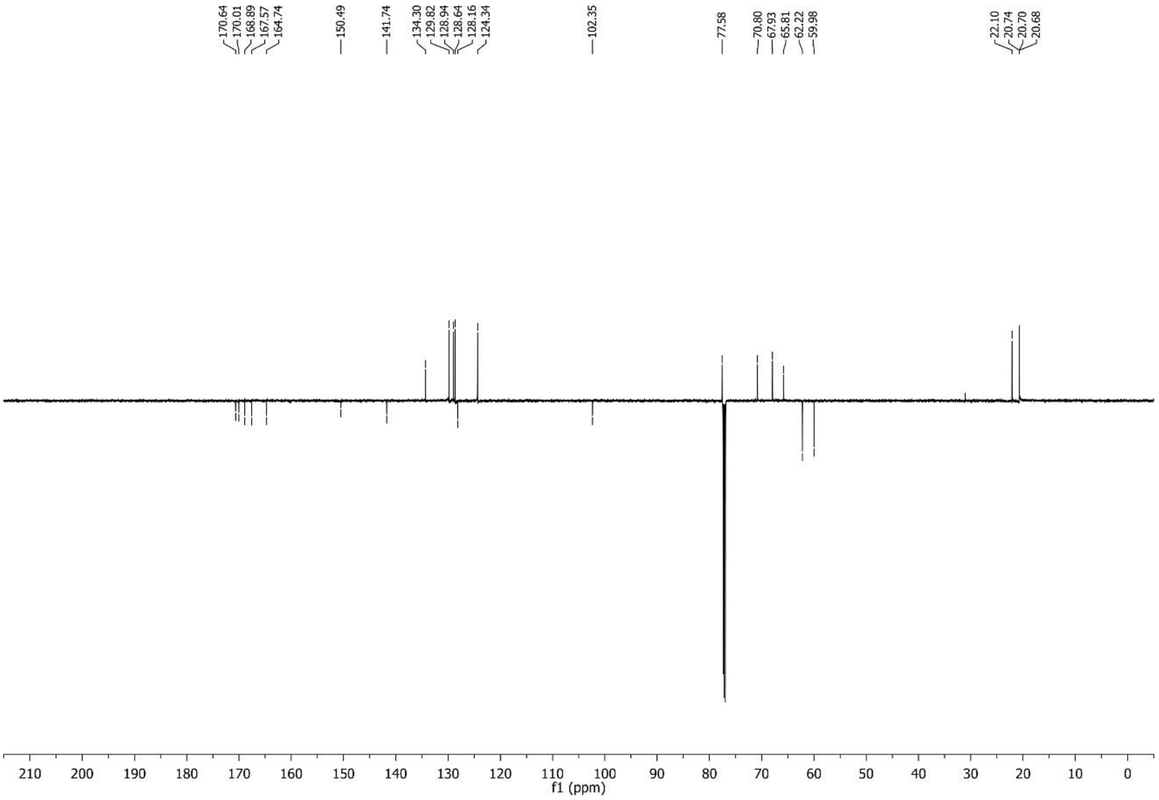

Figure S110: ^{13}C NMR (151 MHz, $CDCl_3$) of 1,2,3,7-tetra-*O*-acetyl-5-*O*-benzoyl-4-*O*-4-nitrobenzenesulfonyl-D-*glycero*-α-D-*lxyo*-hept-2-ulopyranose (**28b**).

3.2.34 2,7-Anhydro-4,5-*O*-isopropyliden-1-*O*-trityl-D-glycero-β-D-arabino-hept-2-ulopyranose (S3)

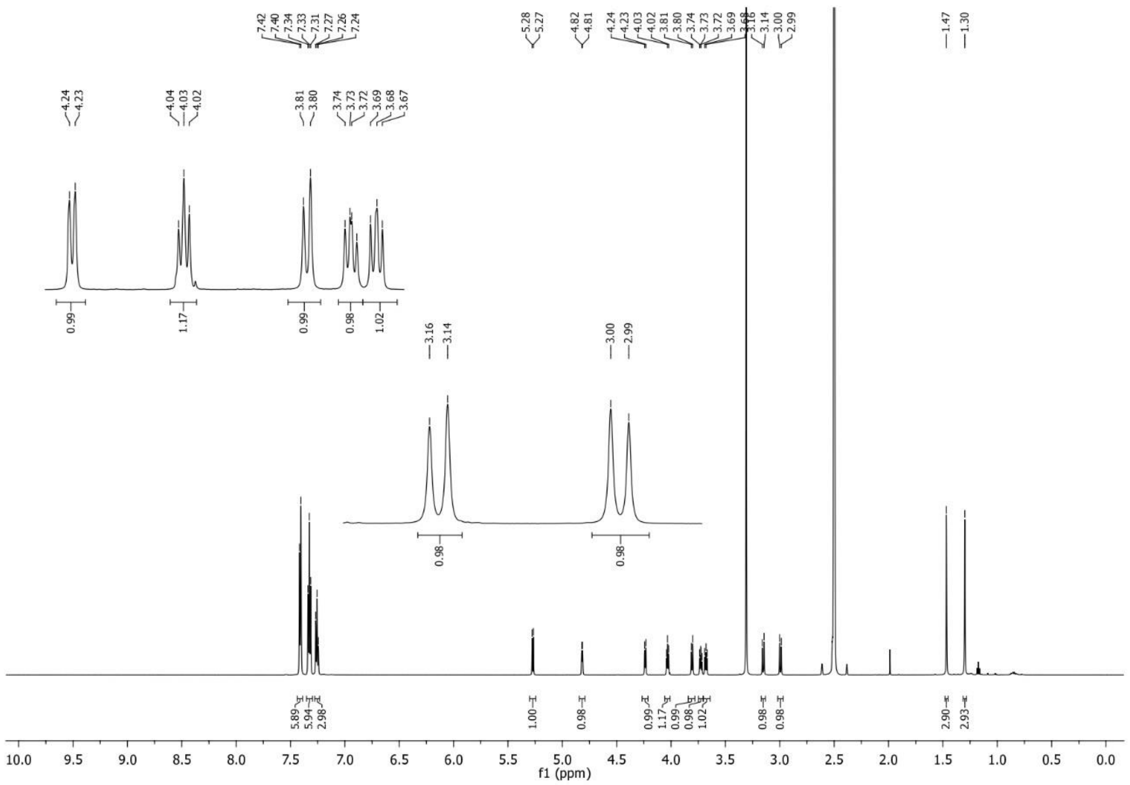

Figure S111: ^{1}H NMR (600 MHz, *d*$_6$-DMSO) of 2,7-anhydro-4,5-*O*-isopropyliden-1-*O*-trityl-D-*glycero*-β-D-*arabino*-hept-2-ulopyranose (**S3**).

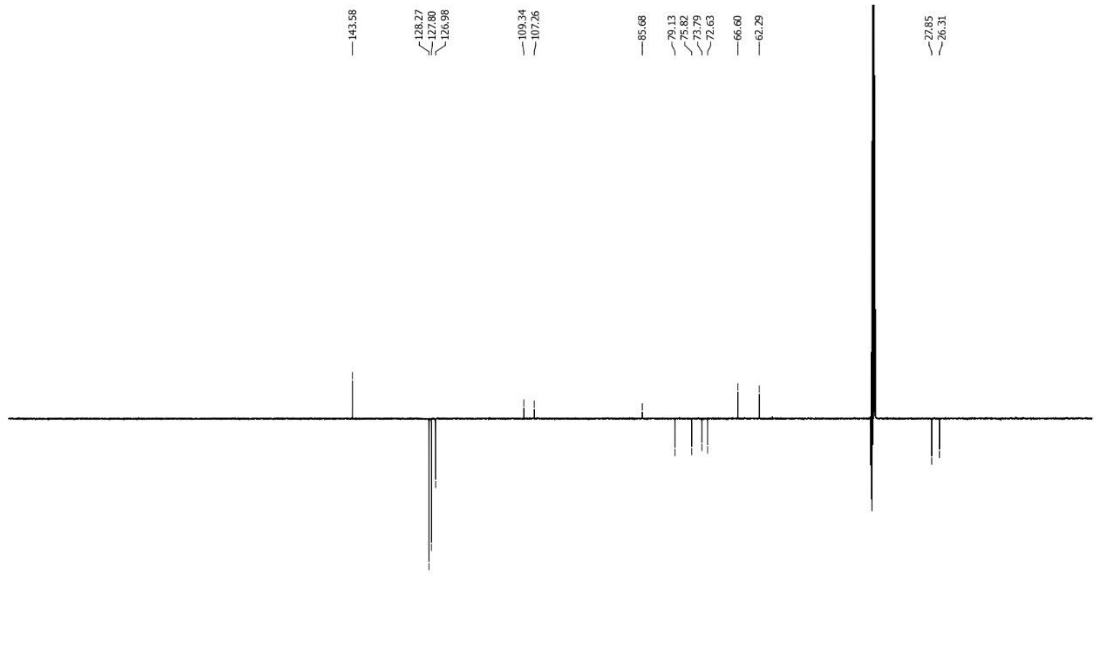

Figure S112: ^{13}C NMR (151 MHz, *d*$_6$-DMSO) of 2,7-anhydro-4,5-*O*-isopropyliden-1-*O*-trityl-D-*glycero*-β-D-*arabino*-hept-2-ulopyranose (**S3**).

3.2.35 2,7-Anhydro-4,5-*O*-isopropylidene-1-*O*-trityl-D-*glycero*-β-D-*xylulo*-hept-2-ulopyranose (S4)

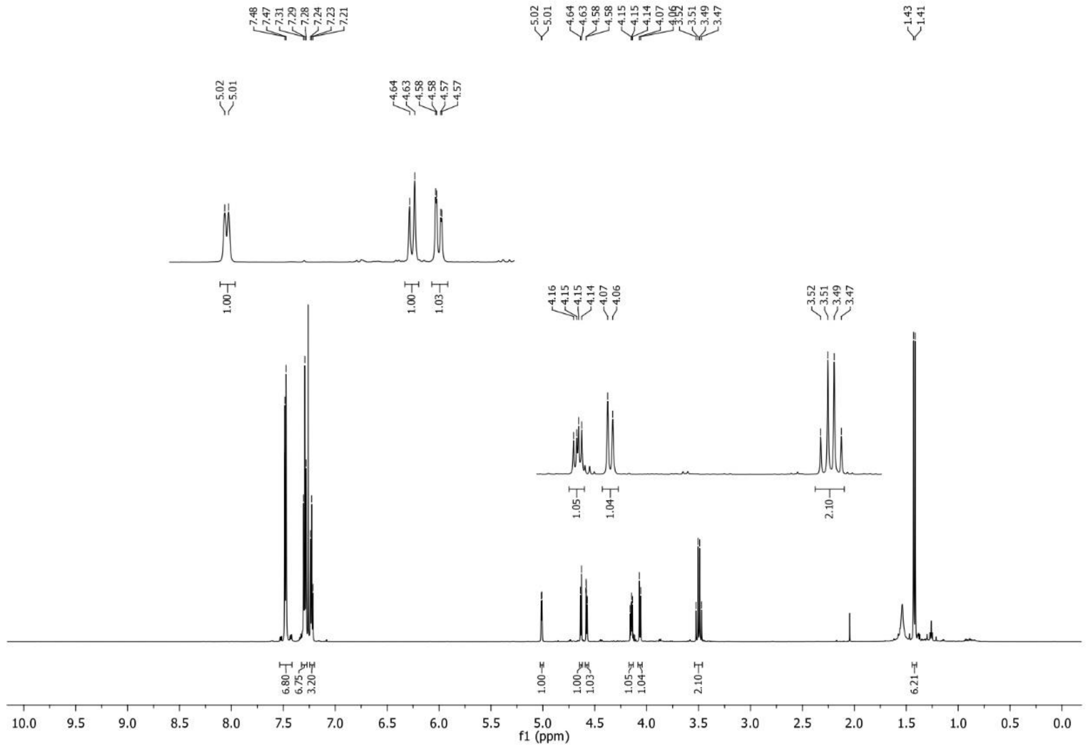

Figure S113: ^{1}H NMR (600 MHz, $CDCl_3$) of 2,7-anhydro-4,5-*O*-isopropylidene-1-*O*-trityl-D-*glycero*-β-D-*xylulo*-hept-2-ulopyranose (**S4**).

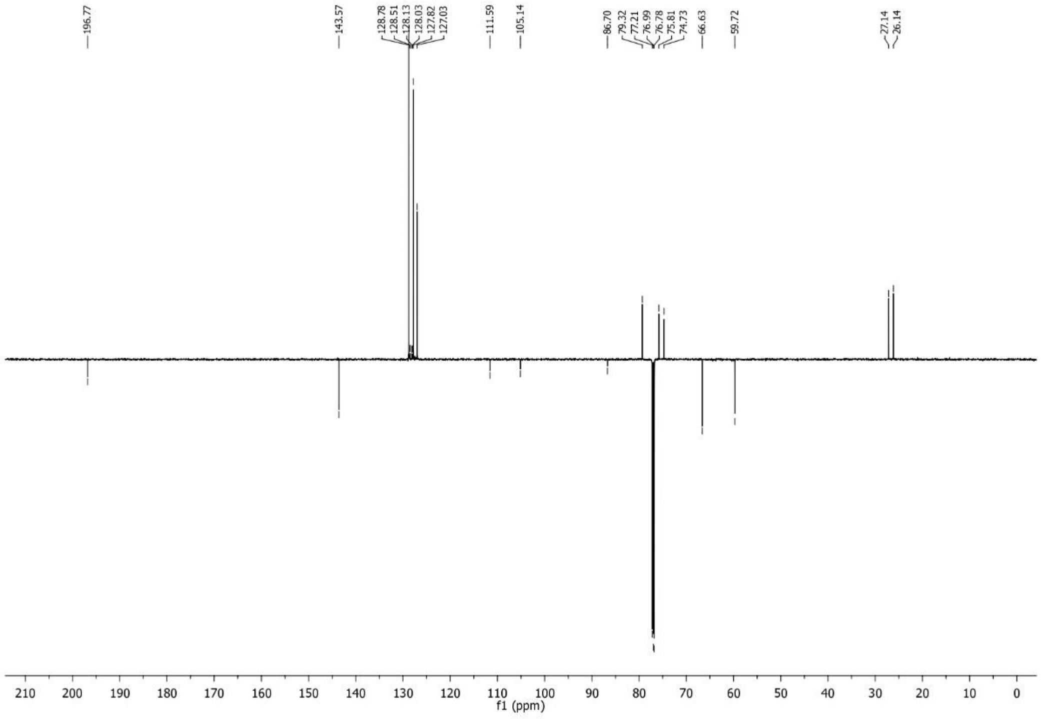

Figure S114: ^{13}C NMR (151 MHz, $CDCl_3$) of 2,7-anhydro-4,5-*O*-isopropylidene-1-*O*-trityl-D-*glycero*-β-D-*xylulo*-hept-2-ulopyranose (**S4**).

3.2.36 2,7-Anhydro-4,5-*O*-isopropylidene-1-*O*-trityl-D-*glycero*-β-D-*ribo*-hept-2-ulopyranose (S5)

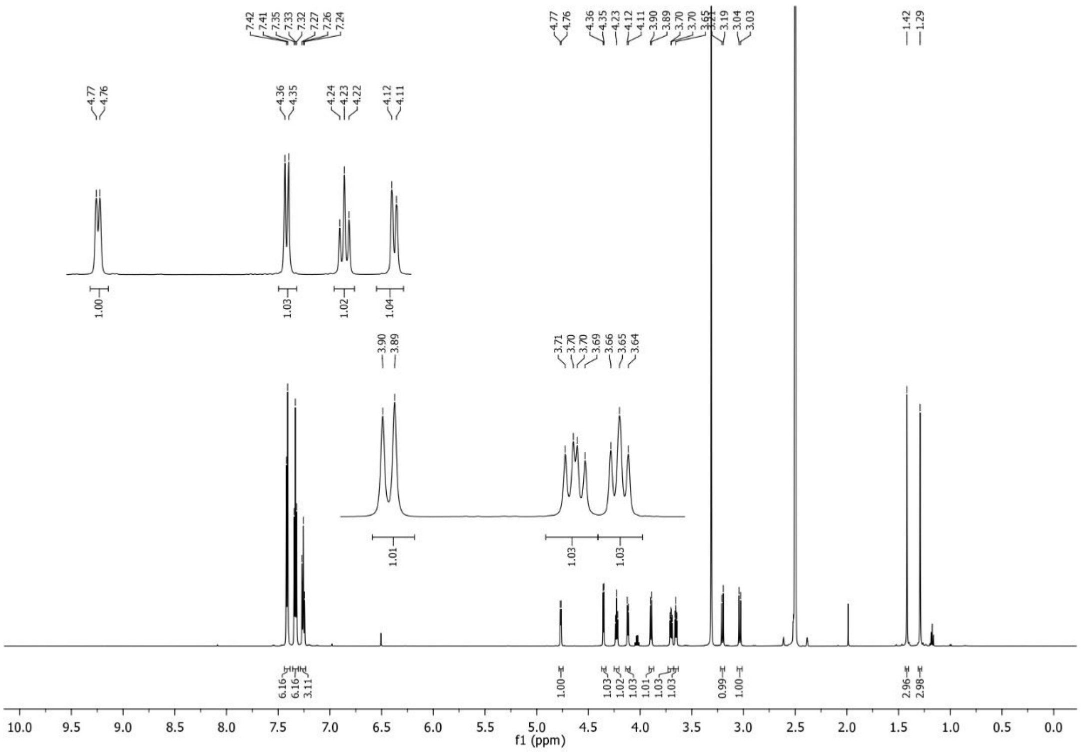

Figure S115: ^{1}H NMR (600 MHz, d_6-DMSO) of 2,7- anhydro-4,5-*O*-isopropylidene-1-*O*-trityl-D-*glycero*-β-D-*ribo*-hept-2-ulopyranose (**S5**).

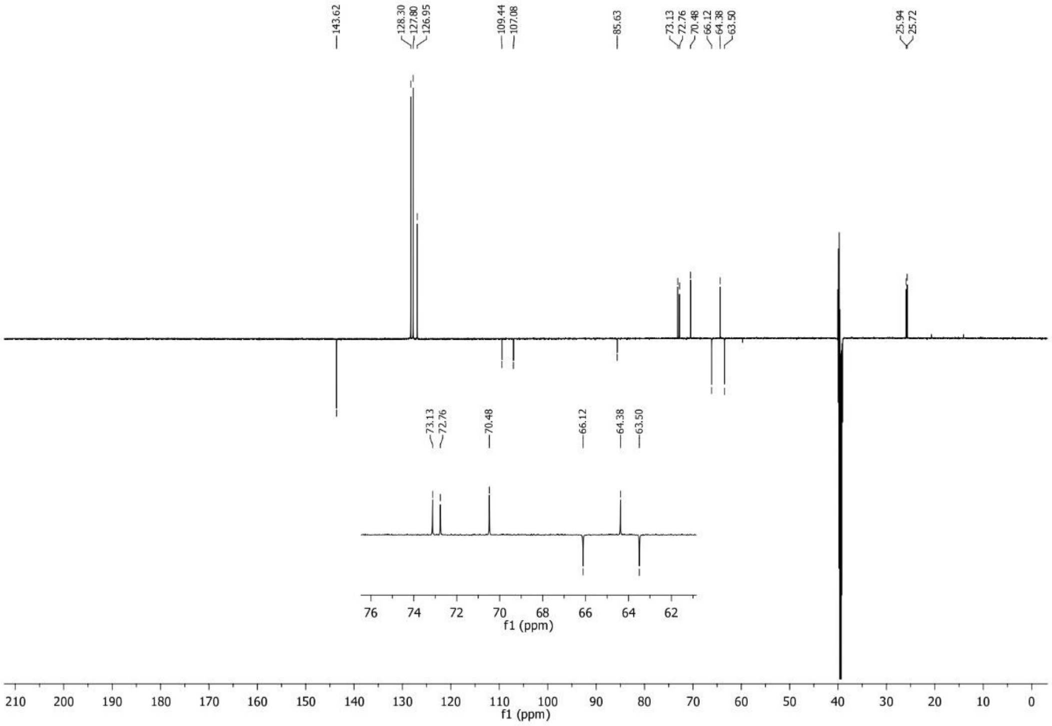

Figure S116: ^{13}C NMR (151 MHz, d_6-DMSO) of 2,7- anhydro-4,5-*O*-isopropylidene-1-*O*-trityl-D-*glycero*-β-D-*ribo*-hept-2-ulopyranose (**S5**).

3.2.37 2,7-Anhydro-3-*O*-benzyl-4,5-*O*-isopropylidene-1-*O*-trityl-D-*glycero*-β-D-*ribo*-hept-2-ulopyranose (S6)

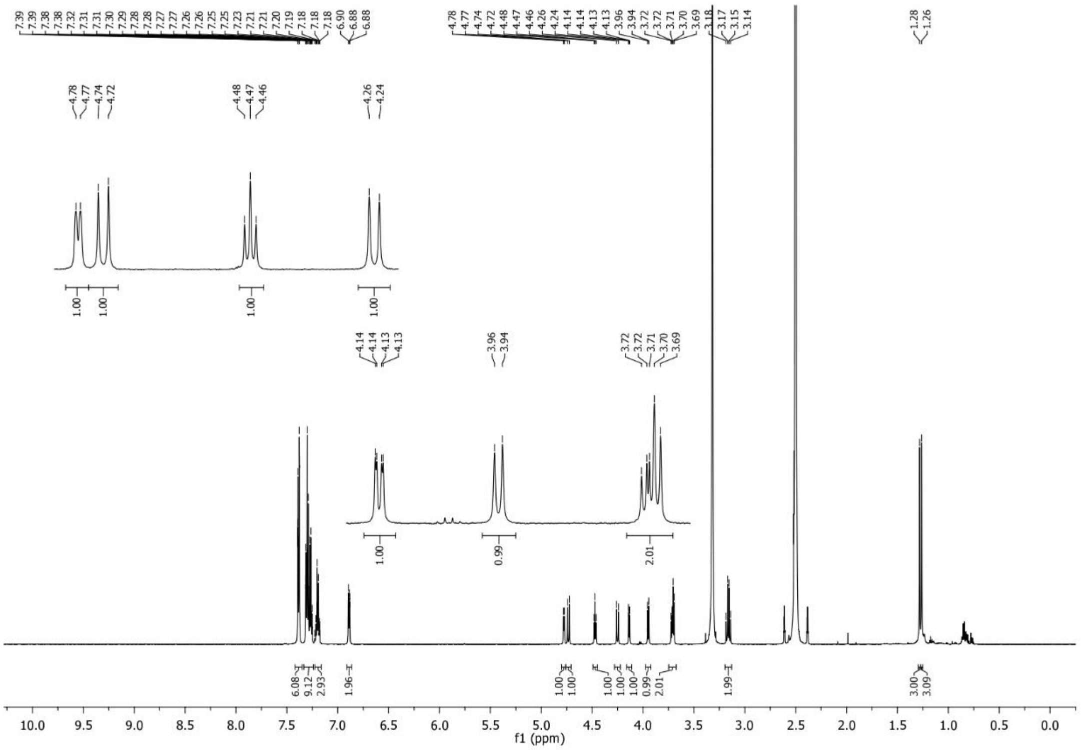

Figure S117: ^{1}H NMR (600 MHz, d_6-DMSO) of 2,7-anhydro-3-*O*-benzyl-4,5-*O*-isopropylidene-1-*O*-trityl-D-*glycero*-β-D-*ribo*-hept-2-ulopyranose (**S6**).

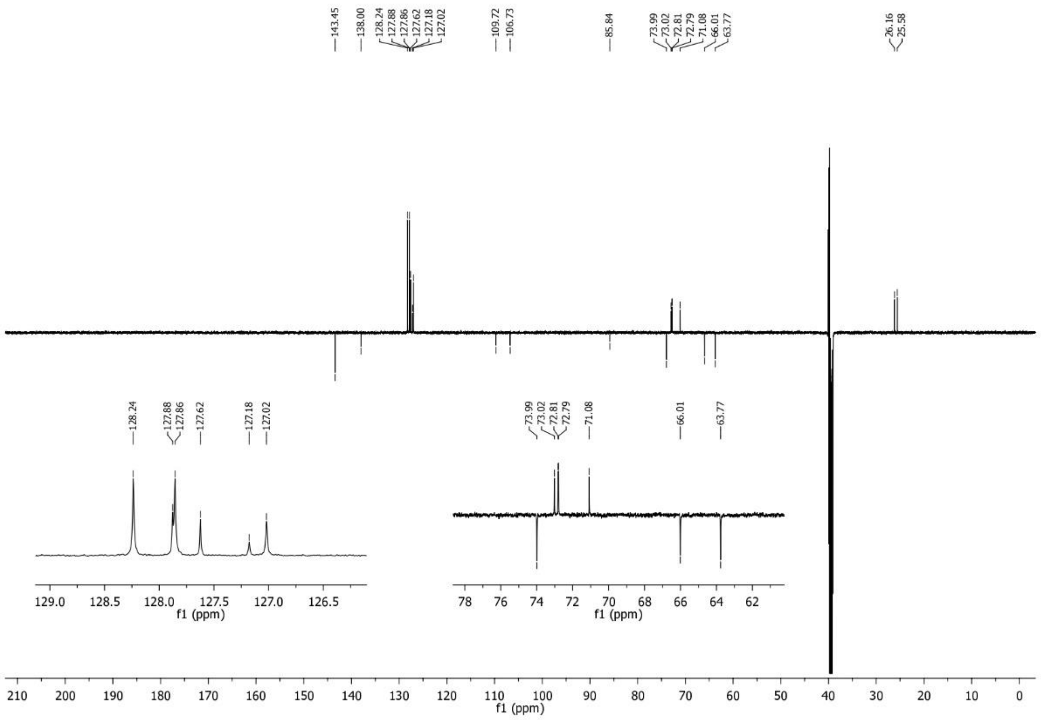

Figure S118: ^{13}C NMR (151 MHz, d_6-DMSO) of 2,7-anhydro-3-*O*-benzyl-4,5-*O*-isopropyliden-1-*O*-trityl-D-*glycero*-β-D-*ribo*-hept-2-ulopyranose (**S6**).

3.2.38 2,7-Anhydro-3-*O*-benzyl-D-*glycero*-β-D-*ribo*-hept-2-ulopyranose (S7)

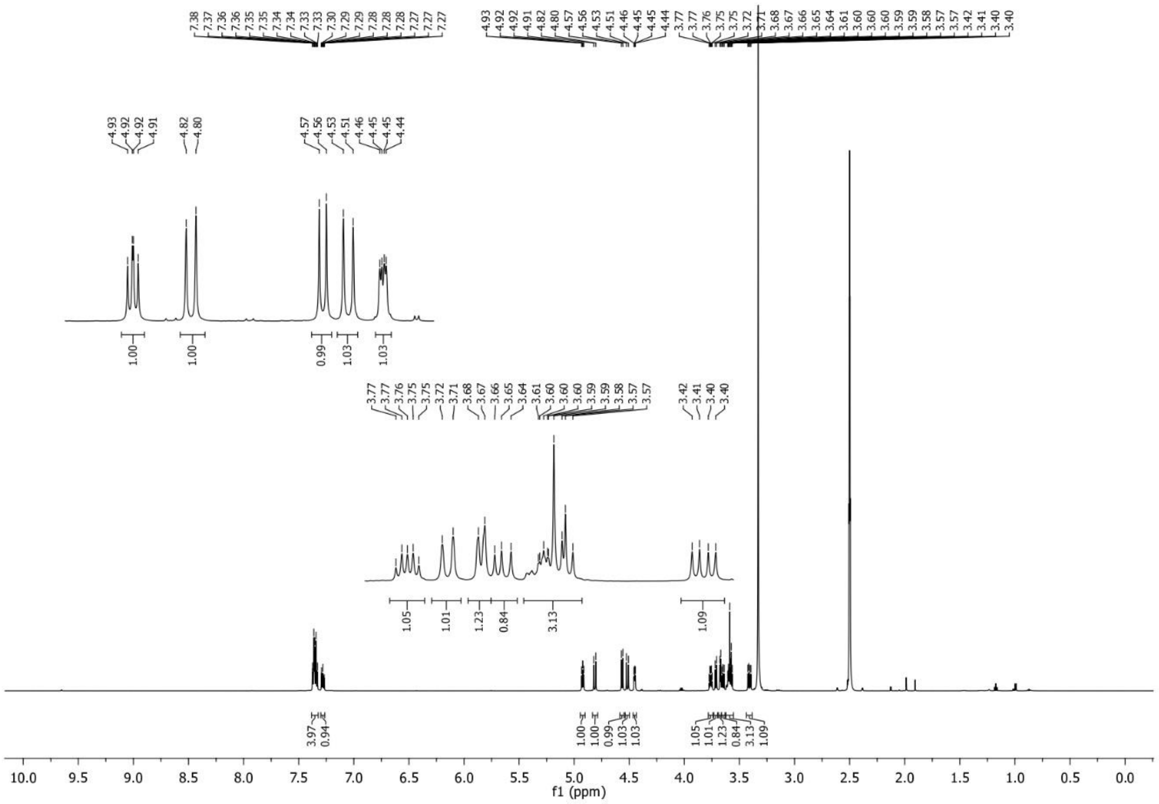

Figure S119: ^{1}H NMR (600 MHz, d_6-DMSO) of 2,7-anhydro-3-*O*-benzyl-D-*glycero*-β-D-*ribo*-hept-2-ulopyranose (**S7**).

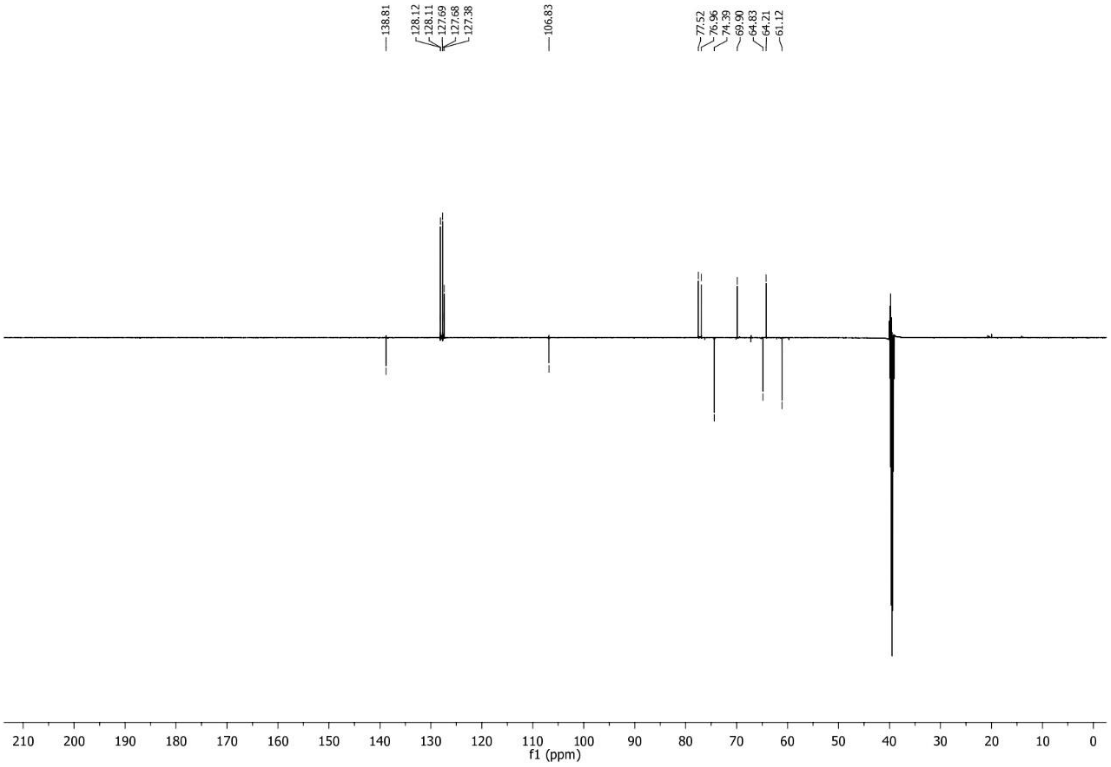

Figure S120: ^{13}C NMR (151 MHz, d_6-DMSO) of 2,7-anhydro-3-*O*-benzyl-D-*glycero*-β-D-*ribo*-hept-2-ulopyranose (**S7**).

3.2.39 1,4,5-Tri-*O*-acetyl-2,7-anhydro-3-*O*-benzyl-D-*glycero*-β-D-*ribo*-hept-2-ulopyranose (S8)

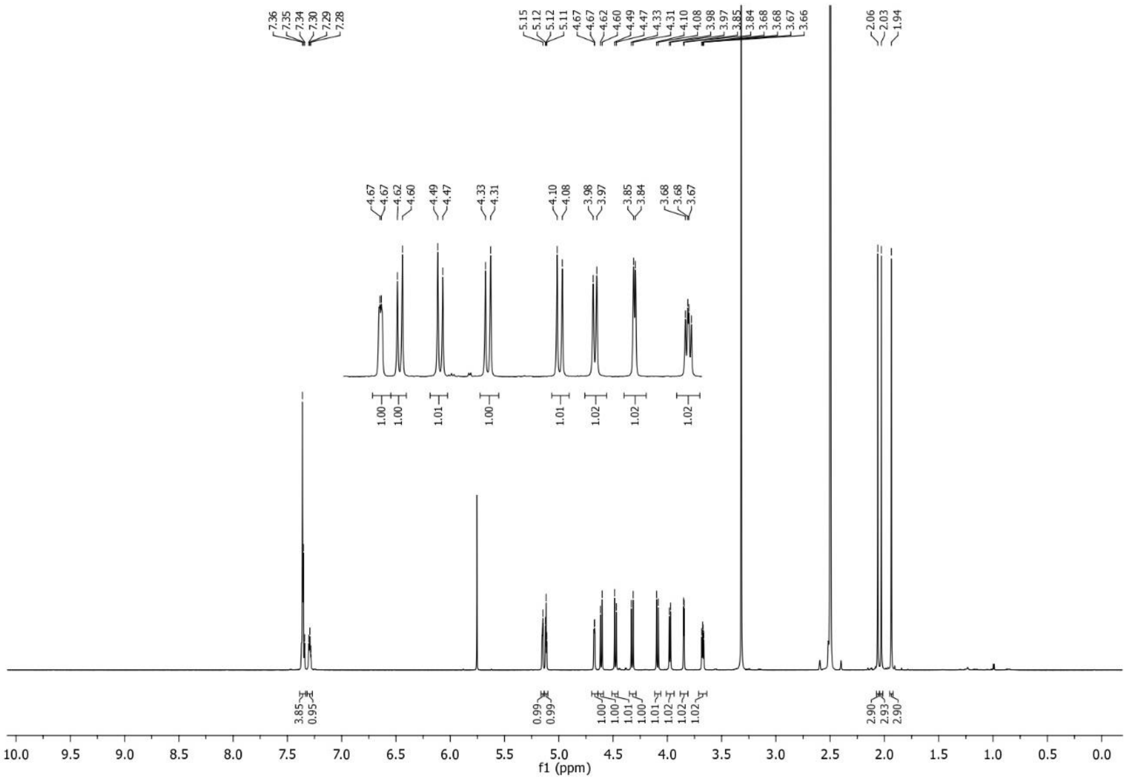

Figure S121: ^{1}H NMR (700 MHz, d_6-DMSO) of 1,4,5-tri-*O*-acetyl-2,7-anhydro-3-*O*-benzyl-D-*glycero*-β-D-*ribo*-hept-2-ulopyranose (**S8**).

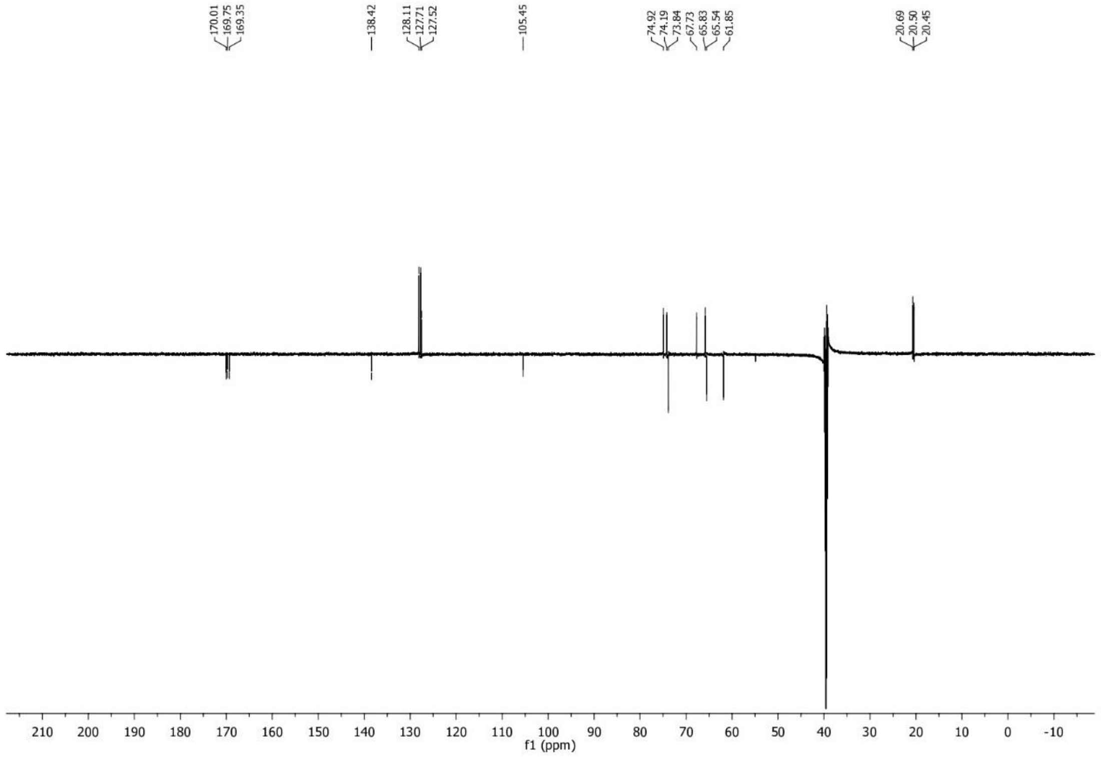

Figure S122: ^{13}C NMR (176 MHz, d_6-DMSO) of 1,4,5-tri-*O*-acetyl-2,7-anhydro-3-*O*-benzyl-D-*glycero*-β-D-*ribo*-hept-2-ulopyranose (**S8**).

3.2.40 1,4,5-Tri-*O*-acetyl-2,7-anhydro-D-*glycero*-β-D-*ribo*-hept-2-ulopyranose (S9)

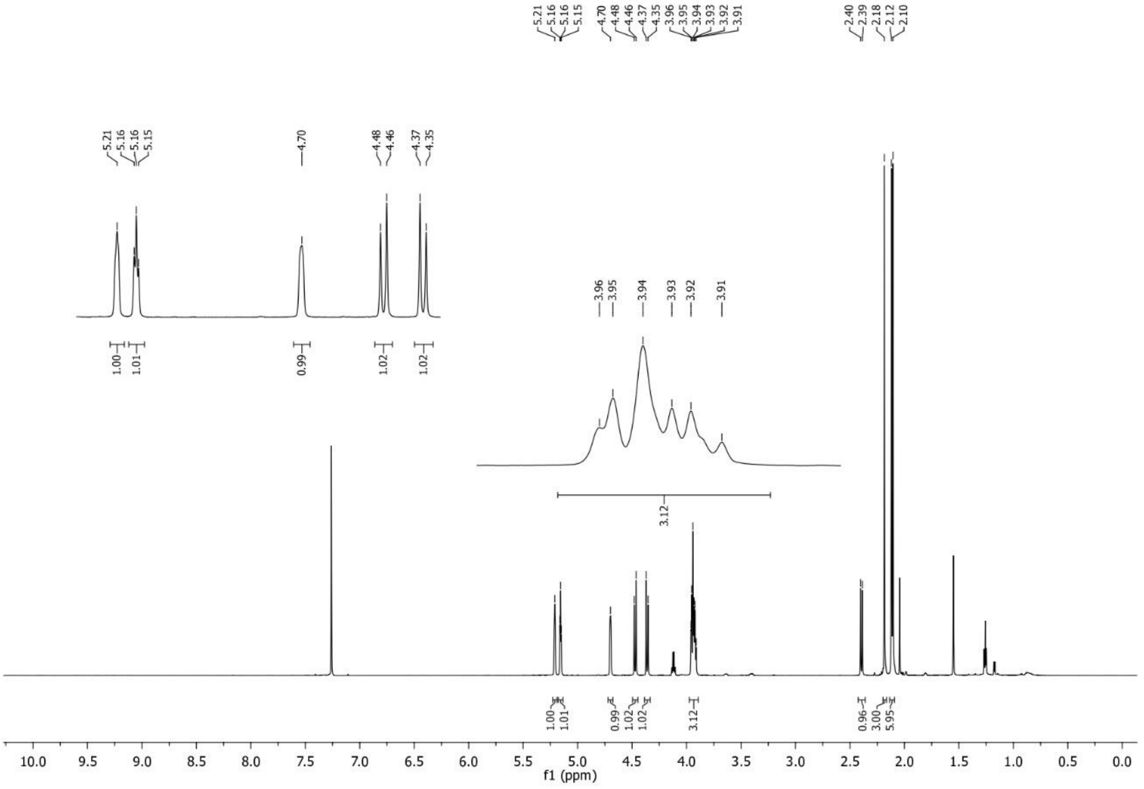

Figure S123: ^{1}H NMR (600 MHz, $CDCl_3$) of 1,4,5-tri-*O*-acetyl-2,7-anhydro-D-*glycero*-β-D-*ribo*-hept-2-ulopyranose (**S9**).

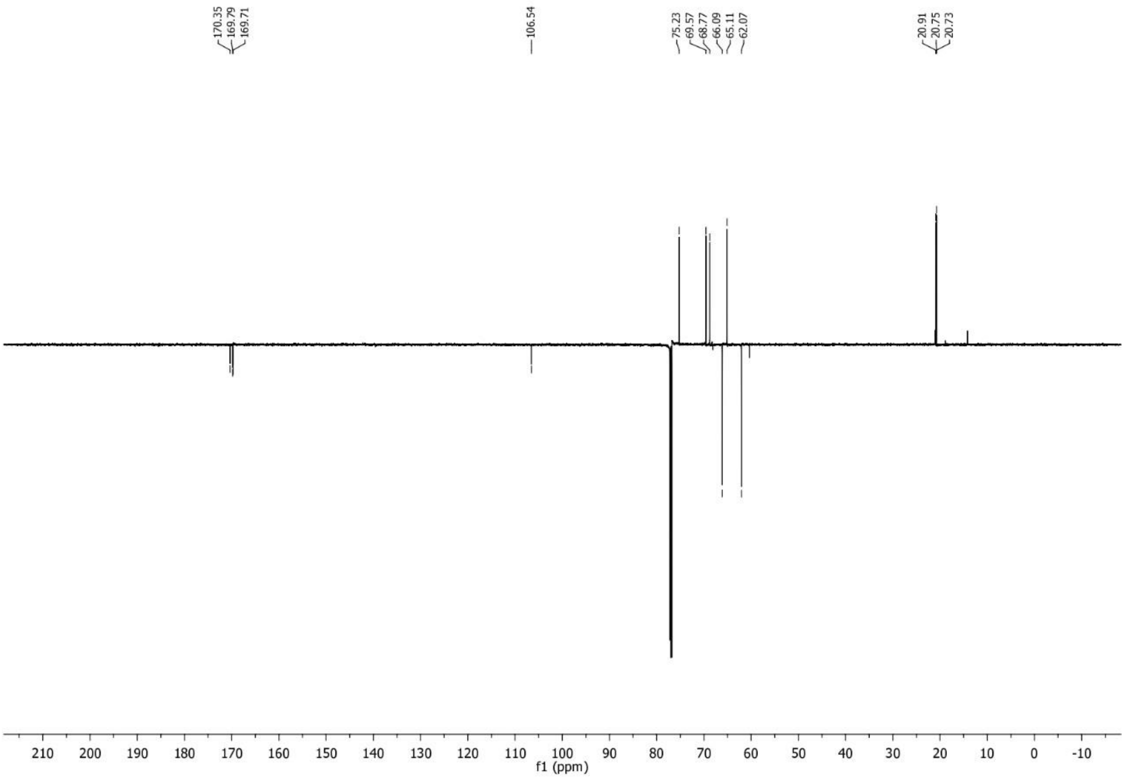

Figure S124: ^{13}C NMR (151 MHz, $CDCl_3$) of 1,4,5-tri-*O*-acetyl-2,7-anhydro-D-*glycero*-β-D-*ribo*-hept-2-ulopyranose (**S9**).

3.2.41 1,4,5-Tri-*O*-acetyl-2,7-anhydro-3-*O*-*para*-toluenesulfonyl-D-*glycero*-β-D-*ribo*-hept-2-ulopyranose (S10)

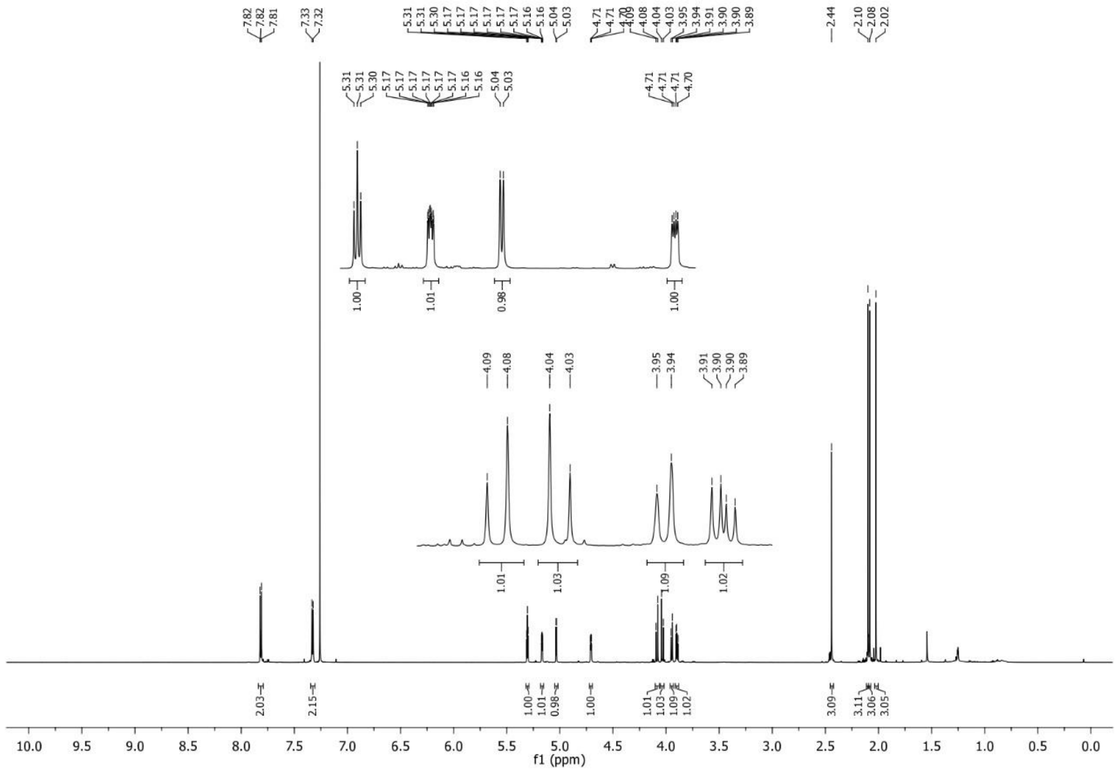

Figure S125: ^{1}H NMR (600 MHz, $CDCl_3$) of 1,4,5-tri-*O*-acetyl-2,7-anhydro-3-*O*-*para*-toluenesulfonyl-D-*glycero*-β-D-*ribo*-hept-2-ulopyranose (**S10**).

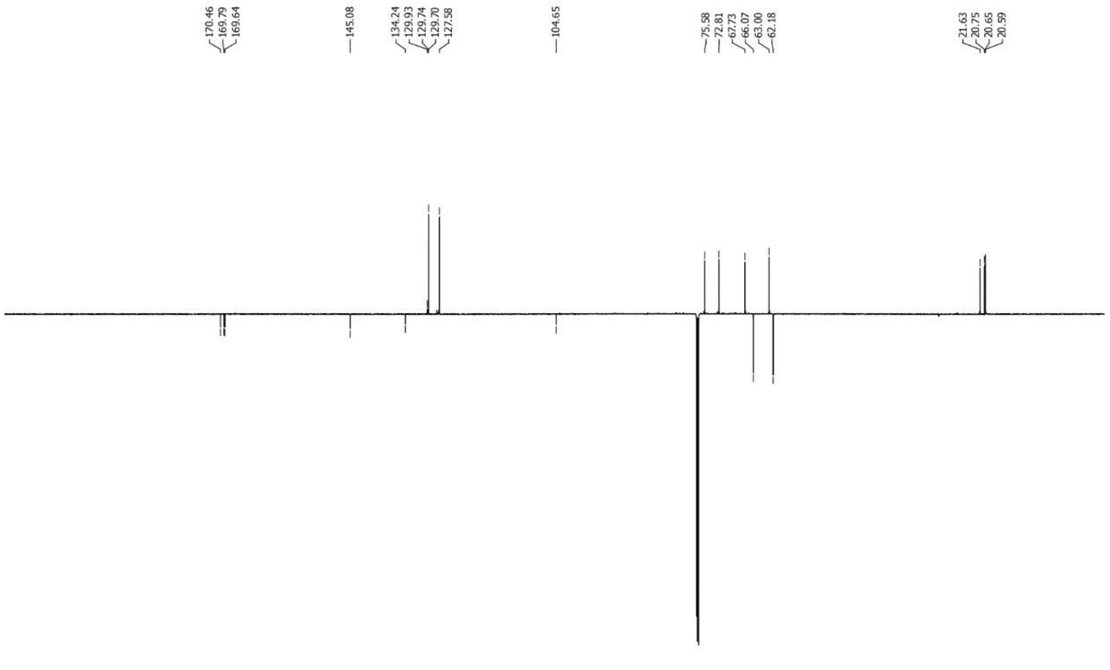

Figure S126: ^{13}C NMR (151 MHz, $CDCl_3$) of 1,4,5-tri-*O*-acetyl-2,7-anhydro-3-*O*-*para*-toluenesulfonyl-D-*glycero*-β-D-*ribo*-hept-2-ulopyranose (**S10**).

3.2.42 1,2,4,5,6-Penta-*O*-acetyl-3-*O*-*para*-toluenesulfonyl-D-*glycero*-β-D-*ribo*-hept-2-ulopyranose (S11)

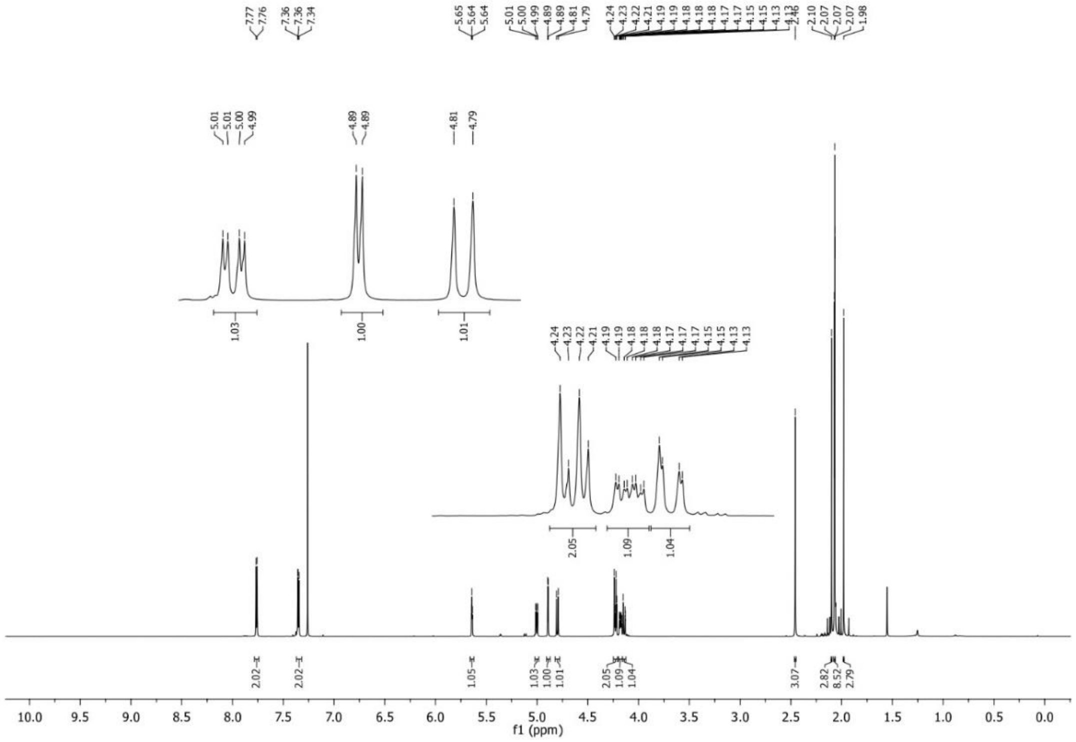

Figure S127: ^{1}H NMR (700 MHz, $CDCl_3$) of 1,2,4,5,6-penta-*O*-acetyl-3-*O*-*para*-toluenesulfonyl-D-*glycero*-β-D-*ribo*-hept-2-ulopyranose (**S11**).

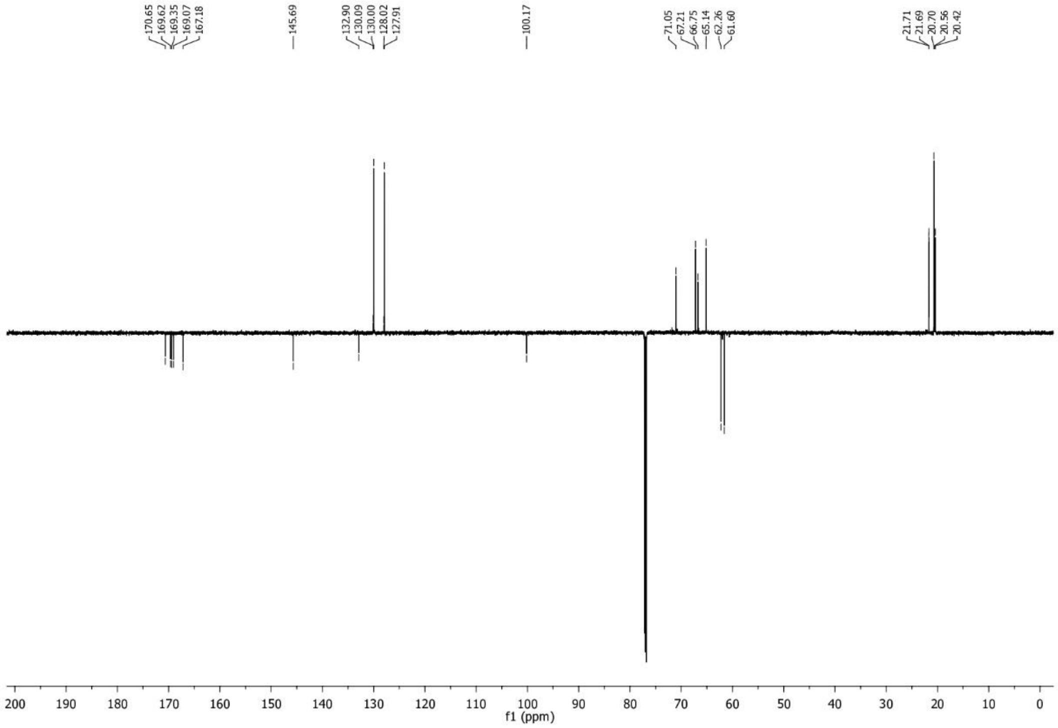

Figure S128: ^{13}C NMR (176 MHz, $CDCl_3$) of 1,2,4,5,6-penta-*O*-acetyl-3-*O*-*para*-toluenesulfonyl-D-*glycero*-β-D-*ribo*-hept-2-ulopyranose (**S11**).

3.2.43 1,4,5-Tri-*O*-acetyl-2,7-anhydro-3-*O*-4-nitrobenzenesulfonyl-D-*glycero*-β-D-*ribo*-hept-2-ulopyranose (S12)

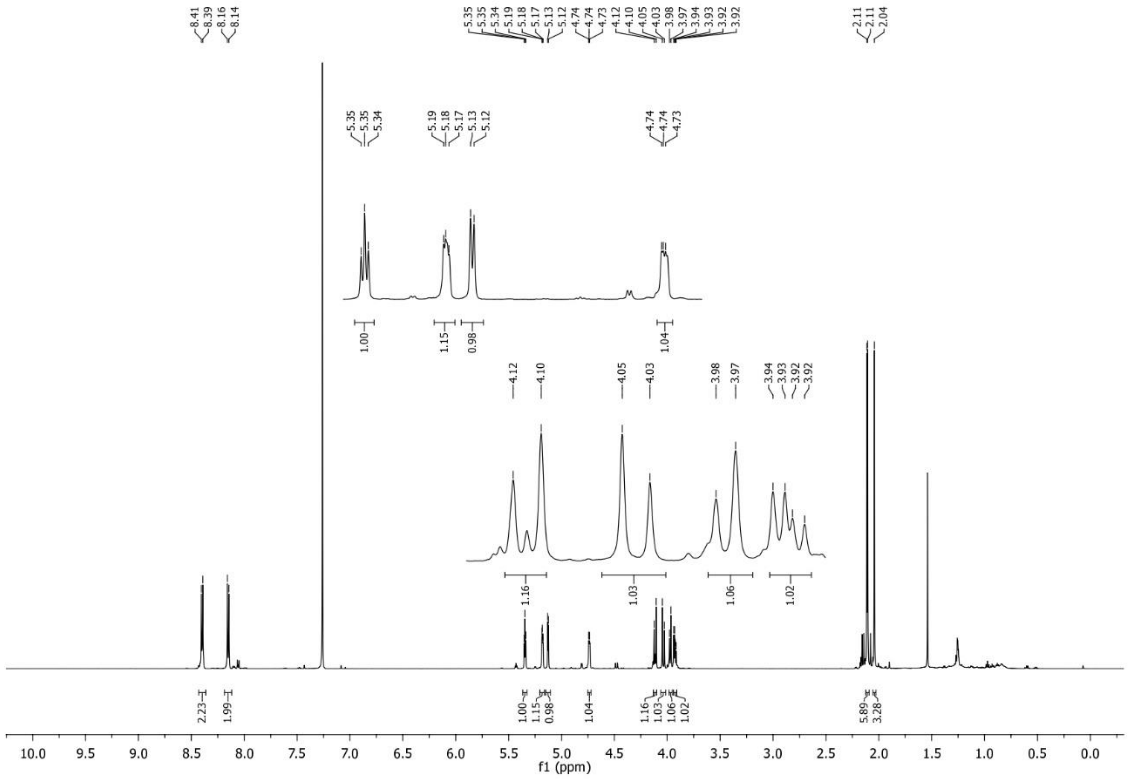

Figure S129: ^{1}H NMR (700 MHz, $CDCl_3$) of 1,4,5-tri-*O*-acetyl-2,7-anhydro-3-*O*-4-nitrobenzenesulfonyl-D-*glycero*-β-D-*ribo*-hept-2-ulopyranose (**S12**).

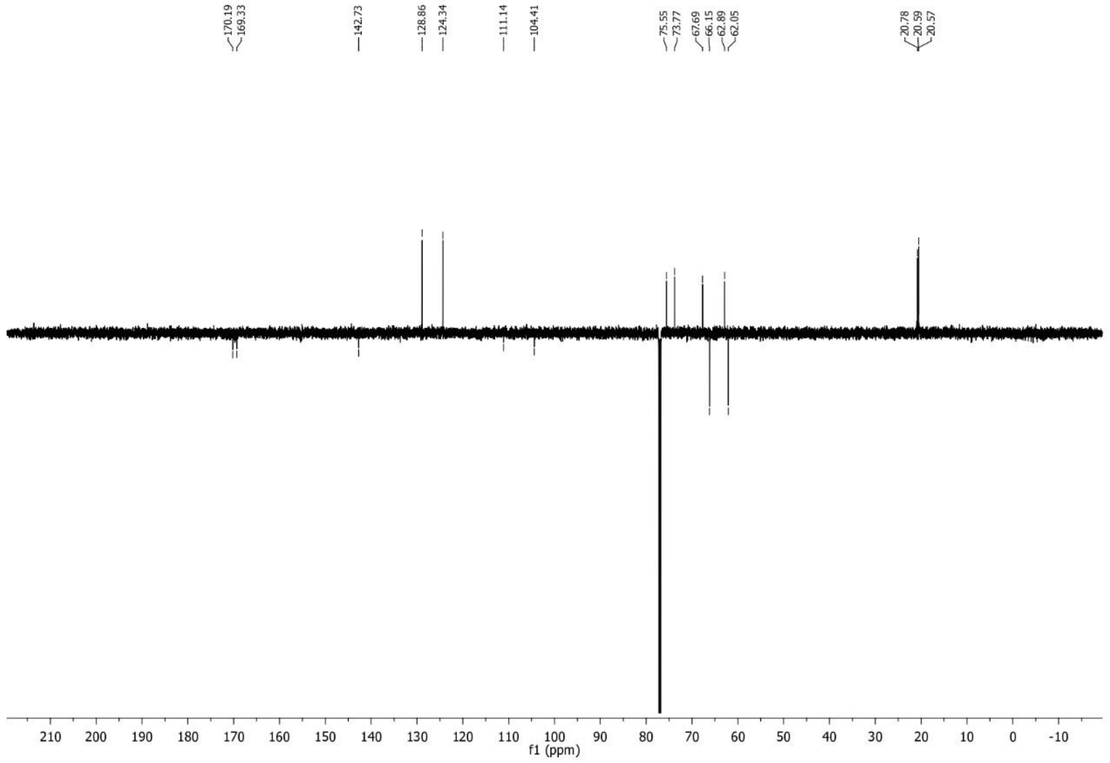

Figure S130: ^{13}C NMR (176 MHz, $CDCl_3$) of 1,4,5-tri-*O*-acetyl-2,7-anhydro-3-*O*-4-nitrobenzenesulfonyl-D-*glycero*-β-D-*ribo*-hept-2-ulopyranose (**S12**).

3.2.44 1,2,4,5,6-Penta-*O*-acetyl-3-*O*-4-nitrobenzenesulfonyl-D-*glycero*-β-D-*ribo*-hept-2-ulopyranose (S13)

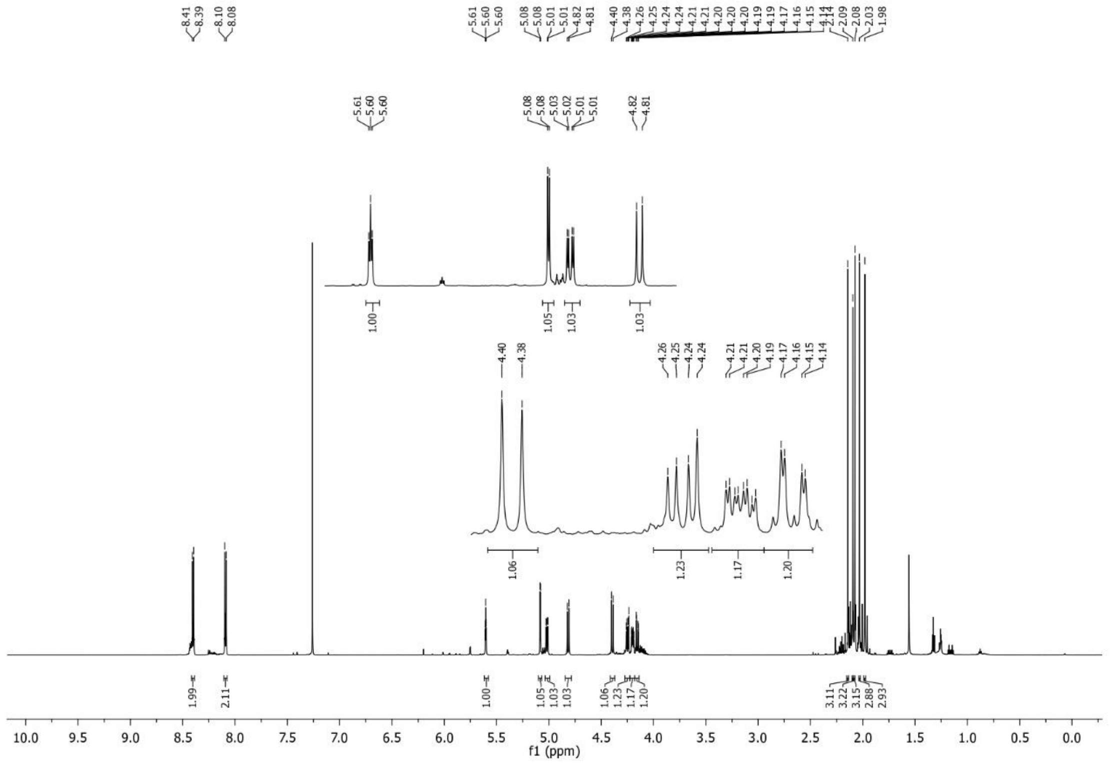

Figure S131: ^{1}H NMR (700 MHz, $CDCl_3$) of 1,2,4,5,6-penta-*O*-acetyl-3-*O*-4-nitrobenzenesulfonyl-D-*glycero*-β-D-*ribo*-hept-2-ulopyranose (**S13**).

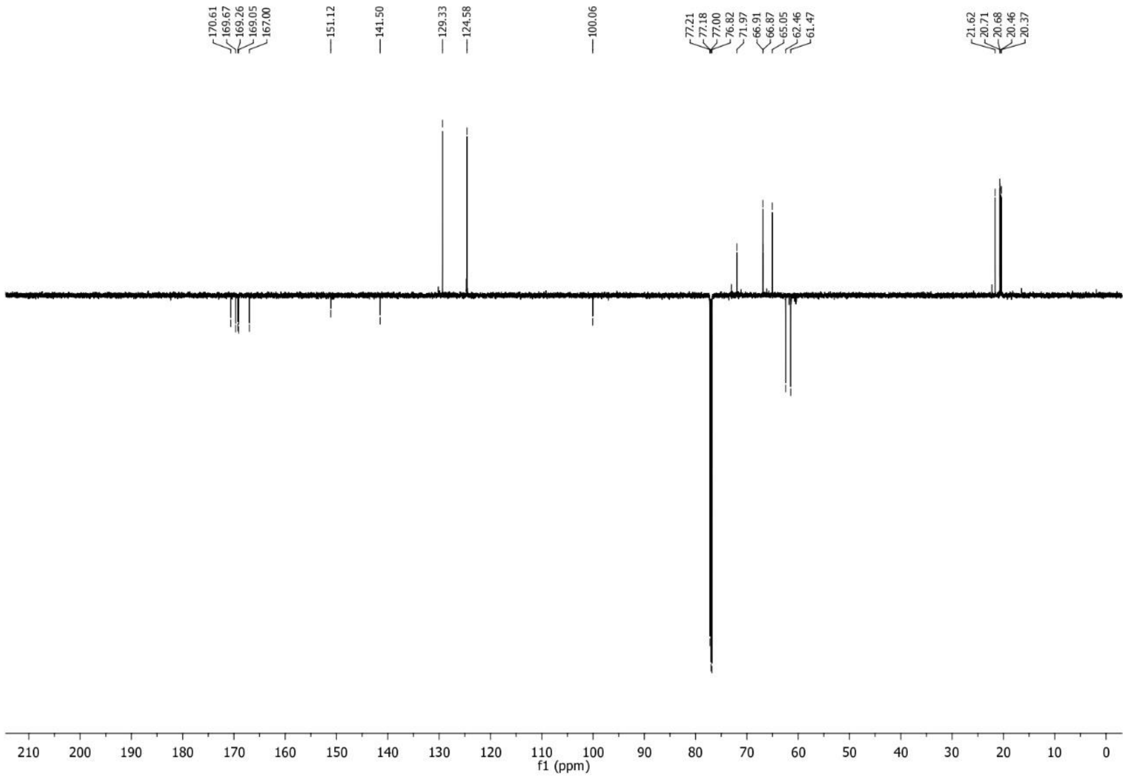

Figure S132: ^{13}C NMR (176 MHz, $CDCl_3$) of 1,2,4,5,6-penta-*O*-acetyl-3-*O*-4-nitrobenzenesulfonyl-D-*glycero*-β-D-*ribo*-hept-2-ulopyranose (**S13**).

4 References

1 C. Loukou, P. Changenet-Barret, M.-N. Rager, P. Plaza, M. M. Martin and J.-M. Mallet, *Org. Biomol. Chem.*, 2011, **9**, 2209.

2 D. R. Hicks and B. Fraser-Reid, *Synthesis*, 1974, **1974**, 203–203.

3 M. B. Comba, M. I. Mangione, A. G. Suárez, A. M. Sarotti and R. A. Spanevello, *Eur. J. Org. Chem.*, 2018, **2018**, 6848–6856.

4 A. P. Rauter, O. Oliveira, T. Canda, E. Leroi, H. Ferreira, M. J. Ferreira and J. A. Ascenso, *Journal of Carbohydrate Chemistry*, 2002, **21**, 257–273.

5 N. Yan, Z.-W. Lei, J.-K. Su, W.-L. Liao and X.-G. Hu, *Chinese Chemical Letters*, 2017, **28**, 467–470.

6 C.-R. Shie, Z.-H. Tzeng, S. S. Kulkarni, B.-J. Uang, C.-Y. Hsu and S.-C. Hung, *Angew. Chem. Int. Ed.*, 2005, **44**, 1665–1668.

7 M. Matwiejuk and J. Thiem, *Eur. J. Org. Chem.*, 2011, **2011**, 5860–5878.

8 N. A. Petasis and E. I. Bzowej, *J. Am. Chem. Soc.*, 1990, **112**, 6392–6394.

9 R. Csuk and B. I. Glänzer, *Tetrahedron*, 1991, **47**, 1655–1664.

10 D. Waschke, Y. Leshch, J. Thimm, U. Himmelreich and J. Thiem, *Eur. J. Org. Chem.*, 2012, **2012**, 948–959.

11 Y. Leshch, A. Jacobsen, J. Thimm and J. Thiem, *Org. Lett.*, 2013, **15**, 4948–4951.

12 M. L. Hofferberth and R. Brückner, *Angew. Chem. Int. Ed.*, 2014, **53**, 7328–7334.

13 Y.-H. Shi, Y.-F. Xie, Y.-Q. Liu, Q.-C. Wei, W.-R. Xu, L.-D. Tang and G.-L. Zhao, *Chinese Chemical Letters*, 2014, **25**, 561–566.

14 W. T. Haskins, R. M. Hann and C. S. Hudson, *J. Am. Chem. Soc.*, 1952, **74**, 2198–2200.

15 K. Heyns, W. Soldat and P. Köll, *Chem. Ber.*, 1973, **106**, 1668–1677.

16 P. Köll and T. Schultek, *Chem. Ber.*, 1977, **110**, 1994–2004.

17 K. Heyns, H. Neste and J. Thiem, *Chem. Ber.*, 1981, **114**, 891–908.

18 M. Adinolfi, G. Barone, L. Guariniello and A. Iadonisi, *Tetrahedron Letters*, 1999, **40**, 8439–8441.

19 M. Zottola, B. V. Rao and B. Fraser-Reid, *J. Chem. Soc., Chem. Commun.*, 1991, 969.

20 C. A. A. Van Boeckel and T. Beetz, *Tetrahedron Letters*, 1983, **24**, 3775–3778.

21 A. Chiotellis, F. Sladojevich, L. Mu, A. Müller Herde, I. E. Valverde, V. Tolmachev, R. Schibli, S. M. Ametamey and T. L. Mindt, *Chem. Commun.*, 2016, **52**, 6083–6086.

22 A. Haschemi, P. Kosma, L. Gille, C. R. Evans, C. F. Burant, P. Starkl, B. Knapp, R. Haas, J. A. Schmid, C. Jandl, S. Amir, G. Lubec, J. Park, H. Esterbauer, M. Bilban, L. Brizuela, J. A. Pospisilik, L. E. Otterbein and O. Wagner, *Cell Metabolism*, 2012, **15**, 813–826.

23 P. M. Clark, G. Flores, N. M. Evdokimov, M. N. McCracken, T. Chai, E. Nair-Gill, F. O'Mahony, S. W. Beaven, K. F. Faull, M. E. Phelps, M. E. Jung and O. N. Witte, *Proc. Natl. Acad. Sci. U.S.A.*, 2014, **111**, E2866–E2874.
